# Musculoskeletal Impairments and Dysfunction in Individuals with Head and Neck Cancer Following Surgery with Neck Dissection—A Systematic Review

**DOI:** 10.3390/life15050800

**Published:** 2025-05-17

**Authors:** Norazlin Mohamad, Ana Izabela Sobral de Oliveira-Souza, Stephanie M. Ntoukas, Ester Moreira de Castro-Carletti, Munayati Munajat, Liz Dennett, Kerry S. Courneya, Susan Armijo-Olivo, Margaret L. McNeely

**Affiliations:** 1 Physical Therapy Department, Faculty of Rehabilitation Medicine, University of Alberta, Edmonton, AB T6G 2G4, Canada; susanarmijo@gmail.com; 2 Centre of Physiotherapy, Faculty of Health Sciences, Universiti Teknologi MARA, Puncak Alam Campus, Puncak Alam 42300, Malaysia; 3 Faculty of Business and Social Sciences, University of Applied Sciences, 49076 Osnabrück, Germany; anaizabela.oliveira@hotmail.com (A.I.S.d.O.-S.); estermdcastro@gmail.com (E.M.d.C.-C.); 4 Faculty of Kinesiology, Sport, and Recreation, University of Alberta, Edmonton, AB T6G 2R3, Canada; ntoukas@ualberta.ca (S.M.N.); kerry.courneya@ualberta.ca (K.S.C.); 5 Department of Physical Rehabilitation Sciences, Kulliyyah of Allied Health Sciences, International Islamic University Malaysia (IIUM), Kuantan 25200, Malaysia; muna@iium.edu.my; 6 Geoffrey and Robyn Sperber Health Sciences Library, University of Alberta, Edmonton, AB T6G 1C9, Canada; liz.dennett@ualberta.ca; 7 Department of Dentistry, Faculty of Medicine and Dentistry, University of Alberta, Edmonton, AB T6G 2R3, Canada; 8 Supportive Care and Patient Experience, Cancer Care Alberta, Edmonton, AB T6G 2G4, Canada

**Keywords:** head and neck neoplasms, neck dissections, postoperative complications, functional disability, musculoskeletal impairments, systematic review

## Abstract

*Background*: Various forms of head and neck cancer (HNC) surgery that include a neck dissection procedure have been shown to negatively influence the neuromusculoskeletal function of the structures affected. This review aimed to identify the neuromusculoskeletal impairments experienced by individuals with HNC following surgery involving different types of neck dissection procedures. *Methods:* The search was conducted in four databases, encompassing randomized control trials (RCTs), cross-sectional studies, and cohort studies that explored neuromusculoskeletal impairments and dysfunction following HNC surgery. The risk of bias in the included studies was assessed using the ROB 2 tool for RCTs and the ROBINS-I tool for non-RCTs. *Results:* Sixty-seven studies were included (prospective cohort studies *n* = 29; cross-sectional studies *n* = 21; retrospective studies *n* = 13; and RCTs *n* = 4). This review revealed diverse neuromusculoskeletal impairments and disabilities in individuals with HNC after undergoing various types of neck dissection. The overall quality of evidence was low due to methodological limitations and variability in assessment tools. *Conclusions*: The extent and type of neuromusculoskeletal impairment resulting from surgery varied depending on the type of surgery and the outcome measures used. Further high-quality studies with standardized assessment, consistent outcome measures, and long-term follow-up are needed to improve the credibility of research in this area.

## 1. Introduction

Head and neck cancers (HNCs) originate in the oral cavity, nasal cavity, sinuses, lips, mouth, salivary glands, throat, or larynx [1]. Worldwide, more than 660,000 cases are diagnosed, and 325,000 deaths are reported annually. Recently, HNCs have become the seventh most common cancer in the world [2,3]. Oral cancers are the most common type of head and neck cancer in India, Pakistan, and other Southeast Asian countries [4]; in contrast, oropharyngeal cancers are prevalent in Western countries. HNCs affect males more than females, with a ratio of 4:1 [4,5].

The majority of HNCs are squamous cell carcinomas, and the primary risk factors for HNCs are excessive tobacco and alcohol exposure [6,7]. Over recent decades, there has been a significant rise in the number of cases of oropharyngeal squamous cell carcinoma associated with human papillomavirus (HPV). This increase is mainly observed among younger men in North America and Europe, highlighting the growing concern surrounding this issue [8]. A recent comprehensive meta-analysis revealed a significantly higher prevalence of HPV-positive cases in oropharyngeal squamous cell carcinoma compared to oral cavity squamous cell carcinoma, further reinforcing the HPV role as a significant risk factor for developing oropharyngeal cancer [8]. Although HPV-positive HNC in younger patients results in significantly better survival outcomes [9], there are significant impacts on the health and well-being of these individuals [8].

HNCs are often treated with surgery, with or without postoperative radiation therapy or concurrent chemoradiotherapy. Neck dissection is a standard surgical procedure in head and neck cancer management, which involves the removal of lymph nodes from the neck [10]. This procedure is crucial for eliminating metastasis-suspected lymph nodes, with nodal status as a key prognostic factor for patient survival [11]. The extent of neck dissection, including the level and number of lymph nodes removed, can vary depending on the stage and location of the primary tumor [11]. Various forms of HNC surgery, including neck dissection, have been shown to influence anatomical and physiological functions, physical features, and the psychosocial well-being of patients [12]. Although intensive treatment regimens enhance survival, long-term deficits such as swallowing and eating difficulties, speaking impairments, as well as regional neuromusculoskeletal impairments such as restricted jaw opening, neck and shoulder dysfunction, postural changes, balance, and gait problems have been reported to have a significant impact on the quality of life (QOL) of individuals with HNC [13,14].

In the last fifteen years, six systematic reviews related to neuromusculoskeletal impairments and dysfunction in individuals with HNC after surgery have been conducted [10,11,15,16,17,18], with one review performing a meta-analysis specifically looking at nerve injury outcomes following neck dissections [11]. Additionally, two reviews examined outcomes related to trismus [16,17], one review evaluated outcomes related to oral function [18], one review involved outcomes related to neck dissection (ND) [10], and one review examined outcomes following surgical reconstruction procedures [15].

Despite the need to understand how surgeries affect neuromusculoskeletal function and body structures in patients with HNC, none of the published reviews have examined all potential impairments nor considered these from a broad rehabilitation perspective. Given the potential for multiple complications related to neuromusculoskeletal issues following HNC neck surgeries, this review aimed to conduct a comprehensive systematic review to identify, summarize, and assess the quality of existing evidence concerning neuromusculoskeletal impairments such as pain, limited range of motion, decreased muscle strength, and disability in individuals with HNC who have undergone surgery including a neck dissection procedure. The overall aim of this review is to better understand the type, extent, and complexity of common impairments and dysfunction in this population in order to inform the development of pre- and post-operative rehabilitation programs for individuals with HNC and provide recommendations for future research.

## 2. Materials and Methods

### 2.1. Protocol and Registration

This systematic review was registered in the PROSPERO database (https://www.crd.york.ac.uk/prospero/) under the registration number CRD42020210544 and reported based on the recommendations of the Preferred Reporting Items for Systematic Reviews and Meta-Analyses (PRISMA) statement [19].

### 2.2. Search Strategy

This systematic review examined the impact of various neck surgeries, including neck dissection with or without reconstruction, on musculoskeletal impairments and dysfunction in individuals with head and neck cancer. A comprehensive literature search strategy was conducted in Ovid MEDLINE (R), Embase (OVID interface), CINAHL, and SCOPUS. A professional librarian from the University of Alberta and the research team incorporated relevant keywords associated with neck dissection surgeries (e.g., radical neck dissection, modified radical neck dissection, selective neck dissection), head and neck cancer (e.g., oral, oropharynx, larynx), and musculoskeletal impairments and functions (e.g., pain, range of motion, muscle strength). The searches were limited to cohort studies (prospective and retrospective), cross-sectional studies, controlled trials, and randomized controlled trials. There were no restrictions on the date of publication. The electronic searches were performed on 15th February 2024 in the health science databases, with an updated search performed on 26th October 2024. A manual search of reference lists and forward citation tracking from each included study using the Web of Science database was also carried out on 26th October 2024. The full search strings for each database are provided in Supplementary Materials A.

### 2.3. Criteria for Studies

The eligibility criteria for this review were based on the PICOS format (population, intervention, comparison, outcome, and study design), as described below:

Population: This review included all studies involving individuals diagnosed with various types of HNC (e.g., nasal and paranasal sinus cancer, nasopharyngeal cancer, mouth and oropharyngeal cancer, larynx or laryngeal cancer, esophageal cancer, or salivary gland cancer) without any restrictions on age or gender. Individuals without head and neck cancer, lacked on precise diagnoses, or focused on animal studies were excluded from this review.

Intervention(s)/Exposure(s): Different types of HNC surgeries that included a neck dissection (e.g., radical neck dissection (RND), modified radical neck dissection (MRND), selective neck dissection (SND), supraomohyoid neck dissection (SOND), extended neck dissection (END), functional neck dissection (FND), and others), with or without reconstruction, were included in this review.

Comparator(s)/Control: Studies that examined diverse types of cancer therapies, including various surgical techniques, chemotherapy, radiotherapy, chemoradiotherapy, waitlist control, and studies without surgical intervention. Additionally, we considered studies that employed the primary intervention, regardless of the presence of a comparison group.

Outcomes: This study was open to any outcome measures related to neuromusculoskeletal impairments and dysfunction. Pain intensity was considered the primary measure for this review. Other outcomes, such as functional assessment (e.g., range of motion, muscle strength, and muscle activation) and dysfunction assessment, measured using specific questionnaires related to neuromusculoskeletal function (e.g., Gothenburg trismus questionnaire, Neck Dissection Impairment Index (NDII), Neck Disability Index (NDI), Shoulder Pain and Disability Index (SPADI), The Constant–Murley score (CS), or any related questionnaires) were considered as secondary outcomes.

Study designs: Due to the specific nature of our research question, we focused not only on randomized controlled trials (RCTs) but also included prospective cohort studies, retrospective studies, and cross-sectional studies published in English that reported neuromusculoskeletal impairments and dysfunction in participants diagnosed with HNC following surgical intervention. By incorporating diverse study designs, we aimed to achieve a more comprehensive clinical understanding of the impairments. All other types of studies were excluded from this review. The summary of the eligibility criteria for this review is presented in Table 1.

### 2.4. Selection of the Studies

Search results were imported into Covidence (www.covidence.org) to conduct the screening process. Two independent reviewers from a team of five (NM, AISOS, SN, EMCC, and MM) screened the titles, abstracts, and full texts of all potentially relevant studies for this review, adhering to the inclusion and exclusion criteria developed above. Disagreement in included studies was resolved by consensus between the reviewers, and the senior authors (MM and SAO) were consulted when consensus was not achieved.

### 2.5. Data Extraction

Data extraction was first performed independently by one reviewer. A second reviewer checked and verified the extracted data. Disagreements in data extraction were resolved by consensus. Relevant information from each study was extracted and organized in the following domains: article information such as the objective of the study, study design, types of HNC intervention characteristics, outcome measures, data analysis, results, authors’ conclusion, limitations of the study, and recommendations. Quantitative data (e.g., mean, median, standard deviations, 95% confidence intervals) were extracted from the studies when possible.

### 2.6. Risk of Bias Assessment

This review included randomized controlled trials (RCTs) and non-randomized studies (prospective cohort, retrospective, and cross-sectional studies).

Randomized controlled trials

The Cochrane Risk of Bias Tool (RoB 2) was used to analyze the risk of bias for RCTs, as recommended by the Cochrane Collaboration [20]. The risk of bias for each study was rated as follows: *high risk of bias, some concerns*, and *low risk of bias* based on established guidelines [20]. Disagreements in risk assessment ratings were resolved by consensus between reviewers.

Non-randomized studies

To analyze the risk of bias for the non-randomized study, all the independent reviewers used the Non-randomized Studies of Interventions (ROBIN-I) tool, as recommended by Cochrane [21]. The risk of bias for each study was rated as follows: low risk of bias, moderate risk of bias, serious risk of bias, critical risk of bias, and no information [21]. The guidelines established by the Cochrane Collaboration to score each of the domains [21] were used during the assessment. *Robvis* software was used to create the risk of bias plots for the non-randomized study [22]. The software is available at https://mcguinlu.shinyapps.io/robvis/ [22].

### 2.7. Evaluation of the Overall Evidence

In this review, the Grading of Recommendations, Assessment, Development, and Evaluations (GRADE) system was used to rate the overall quality of the evidence of the included studies based on the outcomes of interest [23]. The analysis was performed using GRADEpro. The level of evidence for these outcomes was categorized as high, moderate, low, and very low quality based on the guidelines provided by GRADE [24,25].

### 2.8. Data Synthesis

Pooling quantitative data for meta-analysis was not feasible in this review due to several factors, such as the heterogeneity of study cohorts, incomplete pre-, and post-quantitative data in most of the trials, variations in the types of HNC, and different time frames of post-operative assessment in the included studies. Considering these limitations, a narrative synthesis of the findings was employed to present the results instead of conducting a meta-analysis. The findings were summarized based on types of neck dissection surgeries and reconstruction techniques performed (e.g., radical neck dissection, modified radical neck dissection, selective neck dissection, head and neck reconstruction, and flap procedure) and the musculoskeletal impairment and dysfunction outcome (e.g., pain intensity, range of motion (jaw, shoulder, and neck), muscle strength, disability, and other outcomes).

## 3. Results

### 3.1. Study Selection

Our electronic searches found 3475 articles after duplicate removal, and 2953 studies were excluded during the title and abstract screening phase (e.g., studies not involving HNC, not focused on musculoskeletal impairment, and non-human studies). Subsequently, 522 reports were sought for retrieval, but 34 could not be accessed in full text. A total of 488 full-text articles were assessed for eligibility in Covidence. Of these, 422 were excluded based on the eligibility criteria. Only 66 articles related to neuromusculoskeletal impairments were included following thorough screening. In addition, manual searches were performed to identify other potential studies related to this review’s objective. From these searches, 78 studies were found on the Web of Science database. All studies were analyzed by title and abstract; 77 were excluded since they did not meet the inclusion criteria. Only one study [26] was included during the manual search.

**Figure 1:**
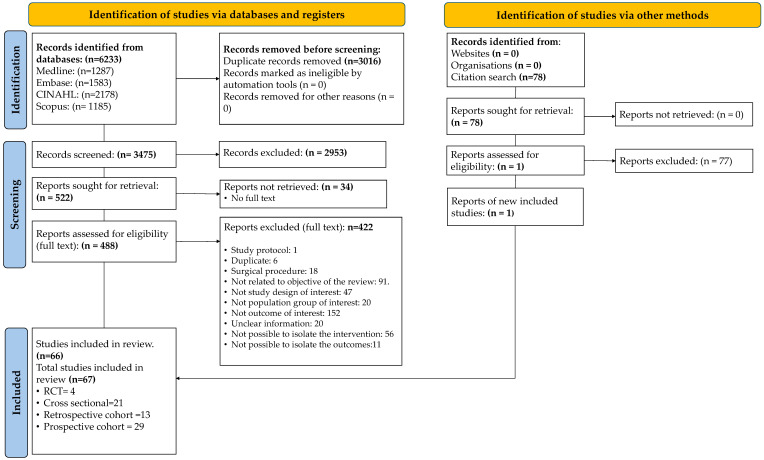
PRISMA flowchart [94].

In total, sixty-seven (67) studies published from 1981 to 2024 fulfilled the eligibility criteria and were included in this review. Among these, prospective cohort studies were the most frequent study designs (*n* = 29) [26,27,28,29,30,31,32,33,34,35,36,37,38,39,40,41,42,43,44,45,46,47,48,49,50,51,52,53,54], followed by cross-sectional studies (*n* = 21) [55,56,57,58,59,60,61,62,63,64,65,66,67,68,69,70,71,72,73,74,75,76], retrospective studies (*n* = 13) [77,78,79,80,81,82,83,84,85,86,87,88,89], and randomized control trials (*n* = 4) [90,91,92,93]. The selection process of the included studies is illustrated in the PRISMA flowchart (Figure 1). Additional details regarding the reasons for exclusion can be found in Supplementary Materials B.

### 3.2. Study Characteristics

Characteristics of the included studies are presented in Table 2. The studies were published between 1984 and 2024 (prospective cohort), 2000 and 2023 (retrospective cohort), 1981 and 2023 (cross-sectional), and 2012 and 2019 (RCTs). Various types of neck dissections such as RND, MRND, SND, END, FND, Mixed ND with preserved/removed cervical nerve root, and mixed ND with reconstruction were found in this review. Most of the studies included individuals experiencing various types of HNC and a wide range of durations from surgery (up to 12 years post-surgery). The summary of the studies included can be found in Supplementary Materials C.

### 3.3. Risk of Bias Within and Across the Studies

**Figure 2:**
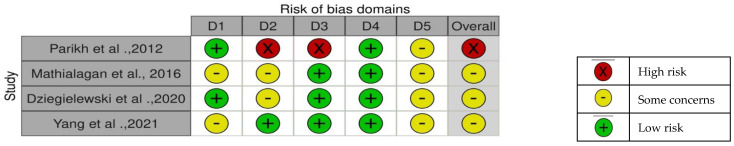
Risk of bias assessed by Cochrane Risk of Bias 2 for randomized controlled trials. **Domains: D1:** randomization process, **D2**: deviation from intended interventions, **D3**: missing outcome data, **D4**: measurement of the outcome, **D5:** selection of reported result.

**Figure 3:**
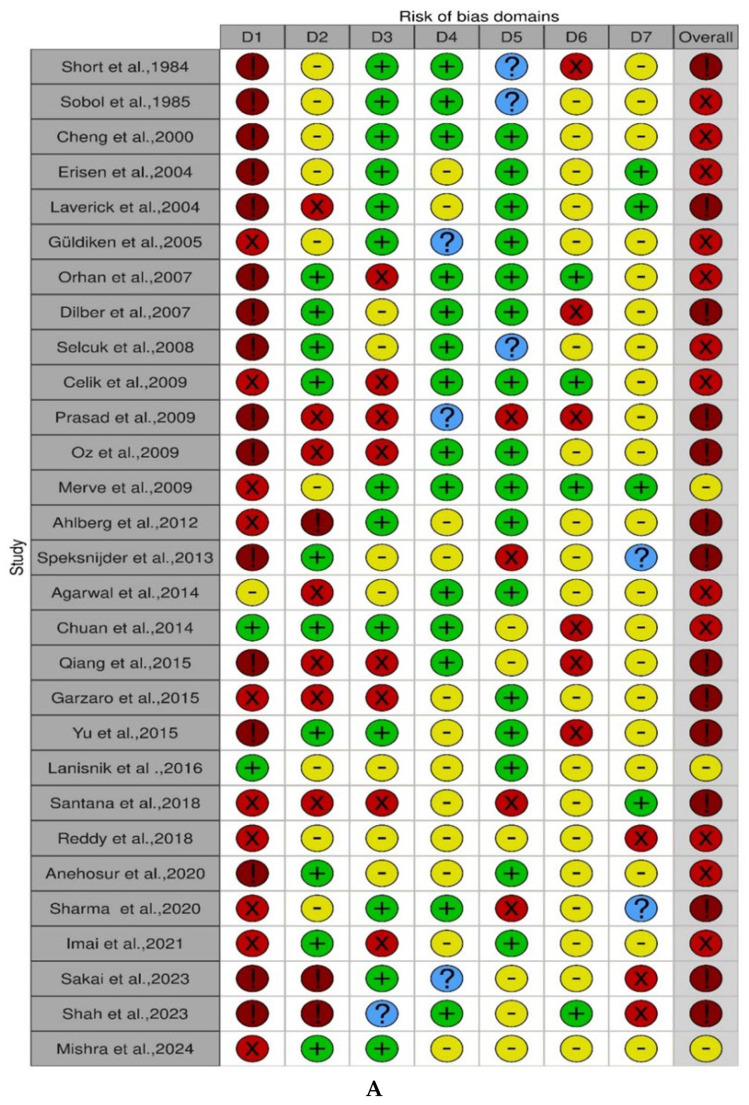

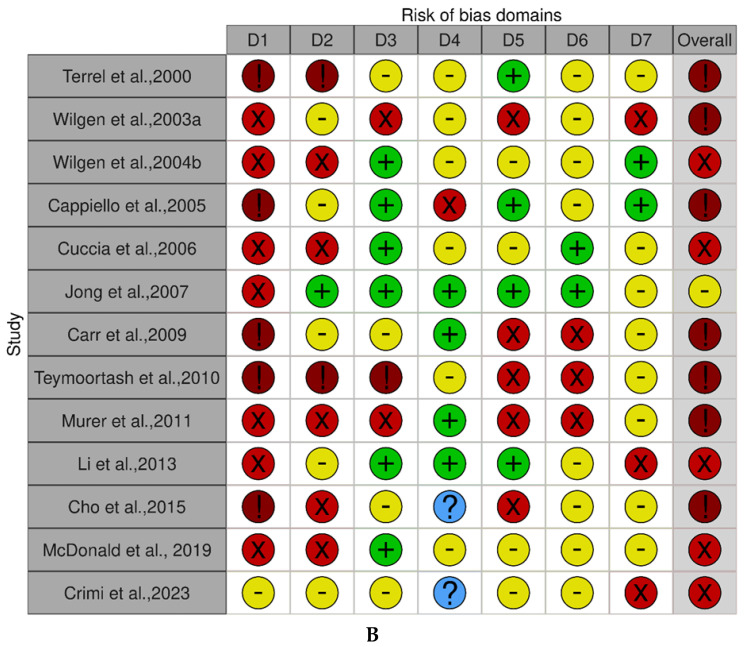

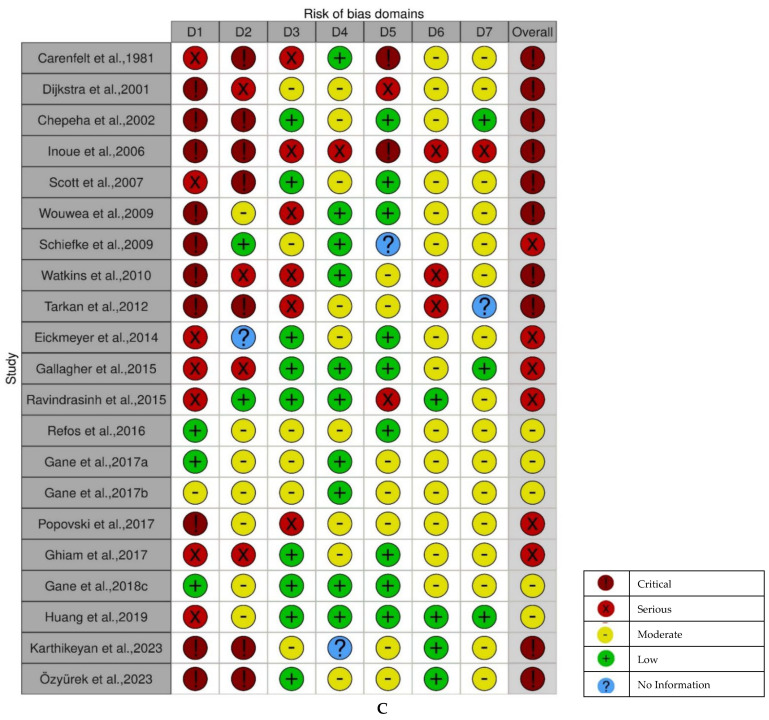
Risk of bias assessed by ROBINS-I tool for non-randomized studies of interventions. (**A**) Risk of bias assessed by ROBINS-I tool for non-randomized studies of interventions (prospective cohort studies). (**B**) Risk of bias assessed by ROBINS-I tool for non-randomized studies of interventions (retrospective cohort studies). (**C**) Risk of bias assessed by ROBINS-I tool for non-randomized studies of interventions (cross-sectional studies) a [60], b [74], c [76]. **Domains**: **D1**: bias due to confounding, **D2:** bias due to selection of participants, **D3:** bias in the classification of interventions, **D4:** bias due to deviations from intended interventions, **D5:** bias due to missing data, **D6:** bias in the measurement of outcomes, **D7:** bias in selection of the reported result. These figures were generated by McGuinness et al. [22].

The ROB assessments for all studies are shown in Figure 2 and Figure 3A–C and Supplementary Materials D.

Randomized controlled trials

The Cochrane Risk of Bias tool demonstrated some bias in each study, with Parikh et al. [92] being the one with the highest risk of bias and the other three studies [90,91,93] with some concerns (Figure 2). Two studies [91,92] employed a random sequence generation method and established explicit inclusion and exclusion criteria for their study populations, thereby ensuring a structured selection process. However, two studies [91,93] had some concerns regarding deviations from intended interventions (D2), which may indicate inconsistencies in protocol adherence or variations in the delivery of interventions across groups. Furthermore, one study [92] exhibited a high risk of bias in both D2 and missing outcome data (D3), suggesting potential deviations that could influence outcomes and a substantial amount of missing data that may compromise the reliability of the findings. These limitations should be considered when interpreting the results of these studies. The summary of ROB across the studies can be found in (see Supplementary Materials D—Figure S1).

Cohort studies

The use of the ROBINS-I tool in the cohort studies (retrospective and prospective) revealed that only three prospective cohort studies [26,51,54] (Figure 3A) and one retrospective cohort study [84] had a moderate risk of bias (Figure 3B). Most studies did not adequately address potential confounding variables. These studies either did not consider confounding factors in their study design or failed to adequately control for confounding factors in the analysis. Consequently, most studies were found to have a serious or critical risk of bias in this domain. Regarding participant selection, some studies in the retrospective and prospective cohorts exhibited serious or critical risk of bias. These studies often lacked clear criteria for selecting participants, potentially introducing selection bias. Furthermore, the participant selection process was not clearly described in certain studies, making it challenging to assess the risk of bias for these studies. The summary of ROBINS-I assessment across the studies for cohort studies can be found in Supplementary Materials D—Figures S2 and S3.

Cross-sectional studies

The ROBIN-I tool for cross-sectional studies demonstrated that only 6 studies [58,60,64,74,75,76] out of 21 studies had a moderate risk of bias (see Figure 3C). Across the studies, due to the nature of the intervention, most of the included studies had a critical risk of bias (high ROB) due to confounding and a critical risk of bias due to the selection of participants. The summary of ROBINS-I assessment across the studies for cross-sectional studies can be found in Supplementary Materials D—Figure S4.

### 3.4. Quality of Evidence

The quality of evidence was assessed using the GRADE approach, which is displayed in Supplementary Materials E. Overall, the quality of evidence of the studies was very low due to the high risk of bias, inconsistency (i.e., heterogeneity of included studies), imprecision of the included studies, and small sample size for some comparisons.

### 3.5. Synthesis of the Results

**The effect of neck dissection by neuromusculoskeletal outcome.**

#### 3.5.1. Pain Intensity Outcomes

The matrix table of the results for pain outcome is displayed in Supplementary Materials F—Table S5.

1. Shoulder pain

Radical Neck Dissection (RND) vs. others.

In this comparison group, three studies [45,69,81] reported on the intensity of shoulder pain in a group of individuals with various types of HNC (mixed HNC). Two studies [45,69] assessed shoulder pain with the VAS, and one study [81] with the health-related quality of life outcome (HRQOL). These studies compared RND with different comparison groups: SND [69] and MRND [45,81]. The details of these studies are reported below.

*RND (sacrificed SAN) vs. MRND (preserved SAN)*

In a retrospective cohort study [81], when comparing RND with sacrificed spinal accessory nerve (SAN) and MRND with preserved SAN in mixed HNC, both groups reported significant shoulder pain in shoulder domains of HRQOL after surgery, and there was no significant difference between RND with sacrificed SAN and MRND with preserved SAN (*p* = 0.07). The certainty of this evidence was considered very low (see Supplementary Materials E—Table S1, comparison E1.1).

However, a prospective cohort study [45] reported that after 6 weeks post-surgery, RND with sacrificed SAN had higher shoulder pain after neck dissection (*p* < 0.05) than the comparison group (MRND with preserved SAN). More than half of the patients with SAN sacrifice had a mean = 2.7/5, while subjects having a preserved SAN had a lower mean = 1.6/5 on a pain score. The certainty of this evidence was considered very low (see Supplementary Materials E—Table S1, comparison E1.2).

*RND vs. SND*

A cross-sectional study [69] evaluated shoulder pain in mixed HNC after undergoing RND and SND. The authors reported that RND had significantly higher shoulder pain intensity than SND (*p* < 0.05) after 10 days of the surgery. The certainty of this evidence was considered very low (see Supplementary Materials E—Table S1, comparison E1.3).

Modified Radical Neck Dissection (MRND) vs. others

Three studies [38,60,69] investigated shoulder pain for this comparison group in individuals with various types of HNC diagnoses (mixed HNC) [60,69] and oropharyngeal carcinoma [38]. The details of these studies are reported below.

*MRND vs. SND*

Two cross-sectional studies [60,69] investigated shoulder pain in mixed HNC for this comparison group. Both studies identified that patients who underwent MRND reported more shoulder pain, as quantified by the Visual Analog Scale (VAS), in comparison to those who underwent SND after 10 days [69] and more than one year post-surgery [60]. Gane et al. [60] also reported that patients who underwent unilateral MRND exhibited a higher VAS score (Mean = 19, SD = 28) than the unilateral SND group (Mean = 12, SD = 16) after the surgery. The certainty of this evidence was considered very low (see Supplementary Materials E—Table S1, comparison E1.4 and E1.5).

*MRND vs. SOND*

A prospective cohort study [38] examined shoulder pain in patients with oropharyngeal carcinoma, comparing MRND with SOND. Both groups reported having shoulder pain after surgery; however, individuals in the SOND group had lower pain scores on the University of Washington Quality of Life questionnaire (UW-QOL) when compared to subjects receiving MRND (*p* < 0.013). The certainty of this evidence was considered very low (see Supplementary Materials E—Table S1, comparison E1.6).

Selective Neck Dissection (SND) vs. others

Two prospective cohort [37,49], two retrospective cohort [79,85], one cross-sectional [75] study, and one RCT [93] evaluated shoulder pain for individuals with mixed HNC [49,75,77,79,85]. These studies compared SND with other interventions in individuals with oropharyngeal carcinoma [93] and oral cavity carcinoma [37]. The details of these studies are reported below.

*SND vs. non-surgical side*

A cross-sectional [75] compared SND with the unaffected shoulder (non-surgical) in mixed HNC. The authors reported that the median (Mdn) VAS score for shoulder pain on the surgical side of SND was Mdn = 4, significantly higher than on the non-surgical side, which was Mdn = 0, (*p* = 0.001) after more than two months of the surgery. The certainty of this evidence was considered very low (see Supplementary Materials E—Table S1, comparison E1.7).

*SND vs. SND (with or without other therapies)*

*SND with radiotherapy* *vs. SND without radiotherapy*

One retrospective cohort study [85] reported that most patients who underwent SND had no persistent shoulder pain after surgery. No significant difference was found in patients who received radiotherapy or no radiotherapy (*p* > 0.05) after more than one year of surgery. The certainty of this evidence was considered very low (see Supplementary Materials E—Table S1, comparison E1.8).

*SND with sacrificed cervical plexus* *vs. SND without sacrificed cervical plexus*

Similarly, in a prospective cohort study [49], no difference was found in VAS evaluation in individuals who underwent SND with or without sacrificed cervical plexus (*p* > 0.05). All patients in both groups reported mild to moderate shoulder pain at two and six weeks after surgery. The certainty of this evidence was considered very low (see Supplementary Materials E—Table S1, comparison E1.9).

*SND (with electrocautery (EC)* *vs. SND (with harmonic scalpel (HS)*

A randomized control trial [93] compared SND (with electrocautery (EC)) with SND (with harmonic scalpel (HS)) in individuals with oral cavity carcinoma. They reported that shoulder pain was still present six months after surgery in the SND group with electrocautery compared to the SND with harmonic scalpel group (*p* = 0.00). The certainty of this evidence was considered very low (see Supplementary Materials E—Table S1, comparison E1.10).

*SND vs. Functional neck dissection (FND)*

A prospective cohort study [37] reported that, when comparing SND with FND in patients with oral cavity carcinoma, shoulder pain was noted in both groups, with the highest mean score being 5.92 after surgery, and no significant difference was found between the groups. The certainty of this evidence was considered very low (see Supplementary Materials E—Table S1, comparison E1.11).

*Supraomohyoid neck dissection (SOND) alone*

A retrospective cohort study [79] evaluated shoulder pain in mixed HNC after more than 1 year of SOND post-surgery. The authors reported that 14 (28%) out of 52 patients complained of ipsilateral shoulder pain following the SOND surgery. The certainty of this evidence was considered very low (see Supplementary Materials E—Table S1, comparison E1.12).

Mixed Neck Dissection (ND): SND and MRND (preserved SAN) alone

A prospective cohort study [28] examined shoulder pain in individuals with oropharyngeal carcinoma who underwent MRND or SND with preserved spinal accessory nerve (SAN). On the 10th postoperative day, 47% of patients reported a pain score of 6 out of 10, while 13% reported a pain score of 8 out of 10. At the six-month follow-up, 35 out of 45 patients had reduced their pain to a score of 2, and two patients had a score of 0. The authors also noted that the improvement could be attributed to physiotherapy treatment. The certainty of this evidence was considered very low (see Supplementary Materials E—Table S1, comparison E1.13).

2. Neck pain

Radical Neck Dissection (sacrificed SAN) vs. Modified Radical Neck Dissection (preserved SAN).

A retrospective cohort study [81] examined neck pain in this comparison group. It compared RND with sacrificed SAN to MRND with preserved SAN among patients with mixed HNC. Patients from both groups reported having neck pain after surgery (mean = 50 points) for RND and (mean= 59.3 points) for MRND-sparing SAN. No significant difference between groups (*p* = 0.16) was found in the study. The certainty of this evidence was considered very low (see Supplementary Materials E—Table S1, comparison E1.14).

Modified Radical Neck Dissection (MRND) vs. Selective Neck Dissection (SND)

Two studies [53,60] investigated neck pain among individuals with mixed HNC in this comparison group. A prospective cohort study [53] reported that shortly after surgery, patients in the SND and MRND groups had significantly higher scores (*p* <0.05) for pain in neck movement compared to healthy controls. There was no significant difference between MRND and SND. The certainty of this evidence was considered very low (see Supplementary Materials E—Table S1, comparison E1.15).

After six months of the surgery, the authors [53] reported no significant difference in the SND group when compared with the healthy group; however, significant differences in pain remained in the MRND group (*p* < 0.05) compared both to the SND group and the group without ND. Additionally, a cross-sectional study [60] reported that patients in the MRND group had a higher score in VAS MD = 25 mm (SD = 31) than the SND group MD = 16 mm (SD = 18) one year after surgery. The certainty of this evidence was considered very low (see Supplementary Materials E—Table S1, comparison E1.16).

Selective Neck Dissection (SND) vs. others

Two studies [53,85] investigated neck pain in this comparison group. One study investigated neck pain in SND with and without radiotherapy [85], and one study compared SND with the non-surgical group [53]. The details of these studies are reported below.

*SND (with radiotherapy) vs. SND (without radiotherapy)*

In a retrospective cohort study [85], no difference was found between SND with and without radiotherapy. No persistent pain was found in patients from either group within a time range of 0.5–9.1 years after the surgery. Out of forty-nine patients, only three in the group receiving radiotherapy reported neck pain. The certainty of this evidence was considered very low (see Supplementary Materials E—Table S1, comparison E1.17).

*SND vs. non-surgical group*

When the SND group was compared with the non-surgery group in a prospective study [53], the SND group scored significantly higher VAS than the non-surgery group immediately after surgery. However, no significant difference was found in the SND group at six months post-surgery. The certainty of this evidence was considered very low (see Supplementary Materials E—Table S1, comparison E1.18 and E1.19).

Mixed Neck Dissection: SND and MRND (sacrificed cervical branches) vs. preserved cervical branches

A retrospective study [84] compared SND/MRND (with cervical root branches removed) with SND/MRND (with cervical root branches preserved). It was found that the frequency and severity of neck pain were significantly higher in the group with the cervical root branches removed than in the group with the cervical root branches preserved (*p* < 0.02), and 30 of the 53 eligible patients (57%) had neck pain after more than 12 months of the surgery. The certainty of this evidence was considered very low (see Supplementary Materials E—Table S1, comparison E1.20).

#### 3.5.2. Range of Motion Outcomes

The matrix table of the results for the range of motion outcome is displayed in Supplementary Materials F—Table S6.

1. Shoulder range of motion.

Radical Neck Dissection (RND) vs. others

Four cross-sectional [64,67,69,72] and two prospective studies [32,41] investigated shoulder range of motion in this comparison group. The details of these studies are reported below.

*RND vs. MRND*

Five cross-sectional studies [64,67,69,70,72] and two prospective studies [32,41] compared RND with MRND in oral carcinoma [67] and mixed HNC [32,41,64,69,70,72] in this comparison group.

One prospective cohort study [32] reported that shoulder abduction and flexion in the RND group were not statistically different from the MRND group at 16 weeks postoperatively. Both groups had significantly reduced shoulder abduction and flexion in patients with mixed HNC. The certainty of this evidence was considered very low (see Supplementary Materials E—Table S2, comparison E2.1).

However, in a long-term follow-up, a prospective study [41] and four cross-sectional studies [64,67,69,72] found that shoulder flexion and abduction were more restricted in the RND group compared to the MRND group after 6–12 months from surgery [41,69,72] and even after 2–7 years from surgery [64,67]. One cross-sectional study [70] evaluated shoulder abduction using the arm abduction test (AAT) after MRND (1 to 23 years post-op) in individuals with mixed HNC. The authors reported that the MRND with sacrificed SAN group scored lower in the arm abduction test (AAT) than those MRND with preserved SAN (*p* = 0.001).

A study [64] also reported that shoulder ROM (abduction and flexion) did not correlate with the presence or absence of radiation. Furthermore, the ability to perform upper limb activities of daily living (ADLs) varied depending on the type of neck dissection. Greater limitations were observed in the group where the nerve was sacrificed compared to the group where it was preserved. The certainty of this evidence was considered very low (see Supplementary Materials E—Table S2, comparison E2.2).

*RND vs. SOND*

A prospective study [32] evaluated shoulder abduction and flexion in mixed HNC after undergoing RND and SOND. The authors reported that individuals who underwent RND significantly had limited shoulder abduction and flexion at 16 weeks for measures of shoulder abduction and flexion (*p* < 0.05) compared to those undergoing SOND. The certainty of this evidence was considered very low (see Supplementary Materials E—Table S2, comparison E2.3).

*RND vs. SND*

A prospective study [41] and two cross-sectional studies [69,72] investigated the shoulder abduction and flexion between RND and SND groups in patients with mixed HNC. All studies reported that shoulder flexion and abduction were significantly more restricted in the RND group compared to the SND group 6–12 months after surgery. The certainty of this evidence was considered very low (see Supplementary Materials E—Table S2, comparison E2.4).

Modified Radical Neck Dissection (MRND) vs. Others

One cross-sectional study [59] and four prospective studies [26,29,30,34] evaluated shoulder range of motion in this comparison group. The details of these studies are reported below.

*MRND vs. non-surgical side*

A cross-sectional study [59] evaluated shoulder abduction and flexion in mixed HNC after undergoing MRND. This study compared the MRND side with the non-surgical side. The authors reported that the MRND group showed a reduction in shoulder abduction and flexion (*p* < 0.002) compared to the non-surgical side shoulder after 6–12 months of post-surgical in individuals with mixed HNC. The certainty of this evidence was considered very low (see Supplementary Materials E—Table S2, comparison E2.5).

*MRND alone*

A prospective cohort study [30] evaluated shoulder abduction at 1 and 6 months after MRND in mixed HNC. They reported that, on goniometric analysis, the mean (±SD) pre-operative and post-operative scores were 4.9 (±0.04) and 3.23 (±0.53), respectively, indicating a significant decrease in arm abduction post-operatively (*p* < 0.001). The certainty of this evidence was considered very low (see Supplementary Materials E—Table S2, comparison E2.6).

*MRND vs. SND*

Two prospective cohort studies [26,29] evaluated shoulder abduction in this comparison group. A prospective study [26] looking at shoulder abduction in mixed HNC reported that the MRND group has significantly decreased shoulder abduction until 3 months post-operatively compared to baseline (*p* = 0.01). Meanwhile, the same finding was noted in the SND group until 1 month of follow-up. The authors also reported a significant decrease in the arm abduction angle in MRND compared to SND at 1 week, 1 month, and 6 months post-operatively (*p* = 0.01, 0.02, and 0.01, respectively). The proportion of patients with active shoulder abduction angle up to 180° without pain at 6 months of the post-operative month was significantly higher in the SND group (90.6%) vs. (63.3%) in the MRND group.

In addition, a prospective cohort study [29] looked at shoulder abduction after MRND and SND in individuals with oral carcinoma after six months of surgery. The author reported a slight reduction in shoulder abduction (<140 degrees) in the MRND group compared to SND after six months; however, no statistical significance between the two groups was detected. The certainty of this evidence was considered very low (see Supplementary Materials E—Table S2, comparison E2.7 and E2.8).

*MRND with pectoralis major myocutaneous flap (PMMC) vs. without PMMC*

A prospective cohort study [34] investigated shoulder abduction and flexion after MRND with pectoralis major myocutaneous flap (PMMC) and without PMMC in individuals with oral carcinoma. The authors reported that both groups showed limited ROM in shoulder abduction and flexion at 3 and 6 months post-operatively (flexion range: 102–113 degrees), (abduction range: 80–95 degrees). No significant difference was found between groups. The certainty of this evidence was considered very low (see Supplementary Materials E—Table S2, comparison E2.9).

Selective Neck Dissection vs. others

Five prospective cohort studies [27,33,40,48], two retrospective [82,85], three cross-sectional studies [56,71,75], and two RCTs [91,92] examined the shoulder ROM in this comparison group. The details of these studies are reported below.

*SND vs. non-surgical side*

Three studies [71,75,85] compared SND with the unaffected side in patients with mixed HNC [75,85] and oral and oropharynx carcinoma [71].

A cross-sectional study [75] evaluated shoulder abduction in mixed HNC after 2 months of SND. The authors reported that shoulder flexion and abduction were significantly better on the unaffected side (*p* < 0.05) after at least 2.6 months post-surgery.

Additionally, a cross-sectional study [71] examined shoulder flexion and abduction in individuals with oral and oropharynx cancer after 1 to 12 years of surgery. The authors [71] reported that shoulder flexion and abduction in individuals with oral and oropharynx cancer significantly differed between the SND and unaffected sides. The unaffected sides had better shoulder flexion and abduction when compared to the SND sides. The mean difference was up to 10 degrees in both ranges.

However, the results were contradicted by a retrospective cohort study [85]. The authors reported no significant difference between the two groups in mixed HNC after 6 months to 9 years of surgery. The average mean of active arm abduction was M = 159.5 degrees on the operated side and M = 162.2 degrees on the non-operated side. The certainty of this evidence was considered very low (see Supplementary Materials E—Table S2, comparison E2.10 and E2.11).

*SND alone*

A cross-sectional study [56] evaluated shoulder ROM in individuals with oral cancer at least after 6 months of SND. The study reported that out of 128 participants, only 51 (39.84%) participants were able to abduct their shoulder to or more than 150° but less than 180° (score 3 of AAT), followed by 31 (24.22%) participants who could abduct up to or more than 90° but not less than 150° (score 2 of AAT). The certainty of this evidence was considered very low (see Supplementary Materials E—Table S2, comparison E2.12).

*SND IIb vs. others*

In this comparison group, two RCT studies [91,92] and one prospective cohort study [33] evaluated shoulder ROM between SND IIa and SND IIb in patients with mixed HNC [33,91] and oral carcinoma [92].

*SND IIb vs. SND IIa*

An RCT [92] evaluated shoulder abduction and flexion among individuals with oral carcinoma after undergoing SND IIb and SND IIa. The authors reported no significant differences in shoulder abduction and flexion for both groups after 6 weeks and 6 months of surgery. Both groups had similar ROM when compared with the unoperated side.

In contrast, another RCT study [91] showed a significant reduction in shoulder motion at 4 and 6 months after the surgery for active abduction and 4 months of active external rotation in the SND IIb group (*p* < 0.05) when compared with SND IIa in mixed HNC. The certainty of this evidence was considered very low (see Supplementary Materials E—Table S2, comparison E2.13 and E2.14).

*SND IIb spared bilaterally vs. SND IIb spared unilaterally*

A prospective cohort study [33] evaluated shoulder abduction and flexion in mixed HNC after undergoing SND (level IIb spared bilaterally) and SND (level IIb spared unilaterally). The authors reported that when SND (level IIb spared bilaterally) was compared to SND (level IIb spared unilaterally), the results showed no significant differences in shoulder abduction after 21 days and 6 months of surgery (*p* > 0.05). Both groups have similar ROMs when compared with pre-operative measurements. The certainty of this evidence was considered very low (see Supplementary Materials E—Table S2, comparison E2.15 and E2.16).

*SND IIb and V dissection* *vs. SND IIb preserved*

Additionally, a prospective cohort study [27] evaluated shoulder abduction and flexion in individuals with mixed HNC after undergoing SND. This study compared SND IIb preserved with SND IIb dissection and V dissection. Both groups showed a slight reduction in shoulder flexion and abduction at 3 months follow-up after surgery. SND IIb preserved showed less shoulder dysfunction than the other group. However, no significant differences were found between the groups. The authors also reported that, over time, all groups showed improvement after 6 months of surgery. The certainty of this evidence was considered very low (see Supplementary Materials E—Table S2, comparison E2.17).

*SND (level V) vs. SND (level II–IV)*

A retrospective cohort study [82] investigated shoulder abduction among individuals with mixed HNC who underwent SND (level II–IV) and SND (level V). They reported that ninety-five percent (95%) of patients in the SND group (level II–IV) had upper limb abduction test results within normal with slight impairment (5%), as compared to the SND group (level V) with only seventy-five percent (75%) of the patients having normal shoulder abduction and with 25% of the participants in this group having reduced shoulder abduction. However, no significant differences were found between the groups. The certainty of this evidence was considered very low (see Supplementary Materials E—Table S2, comparison E2.18).

*SND vs. FND*

A prospective cohort study [48] looked at the effects of two different types of nerve-sparing neck dissection on shoulder function (SND—anterolateral ND (dissection of neck regions 1 through 4 bilaterally) with FND (dissection of neck regions 1 through 5, sparing the accessory nerve, sternocleidomastoid muscle, and internal jugular vein)) in individuals with laryngeal carcinoma. The authors reported that individuals who underwent anterolateral neck dissection (SND) had significantly better shoulder abduction and flexion compared to the FND group (*p* < 0.05) after 6 months of the surgery. The certainty of this evidence was considered very low (see Supplementary Materials E—Table S2, comparison E2.19).

Mixed Neck Dissection vs. others

One retrospective cohort study [84] and three prospective cohort studies [28,35,42] looked for shoulder ROM among mixed HNC [35,42,84] and oropharyngeal cancer [28] in this comparison group. The details of these studies are reported below.

*MRND and SND with cervical root branches removed vs. MRND and SND with preserved cervical root branches*

A prospective cohort study [42] evaluated shoulder abduction among mixed HNCs. The authors reported that the MRND and SND group with the preservation of the cervical root branch had greater shoulder abduction when compared to the group with the sacrificed cervical root branch after 6 months of the surgery (*p* = 0.023).

However, a retrospective cohort study [84] reported that both groups showed a decrease in shoulder abduction after more than 12 months of the surgery. No statistical difference between the groups was found. The certainty of this evidence was considered very low (see Supplementary Materials E—Table S2, comparison E2.20).

*MRND and SND with preserved SAN alone*

A recent prospective cohort study [28] evaluated shoulder ROM among individuals with oropharyngeal cancer after undergoing MRND or SND with preserved SAN. They reported that, at post-operative day 10, 40 out of 45 patients had an arm abduction score of 1 (arm abduction less than 90), and 5 had a score of 2 (less than 150). The certainty of this evidence was considered very low (see Supplementary Materials E—Table S2, comparison E2.21)

At the end of 6 months, of those patients who underwent shoulder physiotherapy as rehabilitation, 27 patients improved to score 4, which is abduction up to 180° with pain or effort, and 15 patients improved to score 3, which is abduction between 150° to 180°, and one patient had score 5, which is abduction above 180° without pain or effort. The study showed a statistically significant difference between time points. The certainty of this evidence was considered very low (see Supplementary Materials E—Table S2, comparison E2.22).

*MRND and SND alone*

A prospective cohort study [35] evaluated shoulder abduction in a group of individuals with various types of cancer (mixed HNC) after going through MRND or SND. The authors reported that pre-operative shoulder abduction decreased one-month post-operative, with an average mean from 165.6 ± 0.98 to 96.5 ± 4.3, respectively (*p* = 0.0001) in mixed HNC. Additionally, they also reported that shoulder abduction was still significantly worse among mixed HNC after 12 months of SND, with an average mean of 157.8 ± 3.9 (*p* = 0.042). The certainty of this evidence was considered very low (see Supplementary Materials E—Table S2, comparison E2.23).

2. Cervical range of motion

Modified Radical Neck Dissection vs. others

One cross-sectional study [76] and two prospective cohort studies [52,53] examined the range of motion of the neck in this comparison group. All studies evaluated cervical ROM in individuals with mixed HNC. The details of these studies are reported below.

*MRND vs. SND*

Flexion and extension

A cross-sectional study [76] evaluated cervical flexion and extension after 0.5–5 years post-surgery in mixed HNC. The authors reported no differences between MRND and SND with nerve preserved in cervical flexion and extension movement. The median was 53 degrees (flexion) and 45 degrees (extension) for SND and 55 degrees (flexion) and 49 degrees (extension) for MRND. However, when compared to healthy subjects, healthy subjects had greater cervical extension compared with the surgical groups (MRND or SND) [76]. The certainty of this evidence was considered very low (see Supplementary Materials E—Table S2, comparison E2.24).

Lateral flexion

Additionally, a prospective cohort study [53] evaluated cervical lateral ROM in individuals with mixed HNC after undergoing MRND or SND. The authors reported that after six months to one year of the MRND procedure, lateral flexion to the contralateral side of the neck in the MRND group was significantly lower (*p* < 0.01) than the lateral flexion of the SND group and a healthy control group. The certainty of this evidence was considered very low (see Supplementary Materials E—Table S2, comparison E2.25).

*MRND vs. SOND*

One prospective cohort study [52] compared the MRND group with SND (supramohyoid) in individuals with mixed HNC. This study reported that all cervical ranges of motion were significantly reduced in MRND combined with external beam radiotherapy two months after treatment. However, after 12 months, all movements have improved except for cervical rotation. On the other hand, SOND did not affect the cervical range of motion (CROM) at any time during the first year after treatment. The certainty of this evidence was considered very low (see Supplementary Materials E—Table S2, comparison E2.26 and E2.27).

Selective Neck Dissection vs. others

Two cross-sectional studies [62,71], one retrospective [85], and one prospective cohort study [33] investigated the range of motion of the cervical spine in this comparison group. The details of these studies are reported below.

*SND vs. non-surgical side*

A retrospective cohort study [85] and cross-sectional [71] evaluated cervical ROM in a group of individuals with mixed HNC [85] and oral/oropharyngeal carcinoma [71] after undergoing SND. Both studies reported no significant differences in all cervical ROM between SND and the non-surgical side after 6 months–9 years [85] and 1–12 years [71] of the surgery. The certainty of this evidence was considered very low (see Supplementary Materials E—Table S2, comparison E2.28).

*SND (level 2b spared bilaterally) vs. SND (level 2b spared unilaterally)*

A prospective cohort study [33] compared SND (level 2b spared bilaterally) with SND (level 2b spared unilaterally) among individuals with laryngeal carcinoma. The study reported that neck flexion, extension, and rotation ROMs were significantly worse in the early post-operative period (day 21 of follow-up) for both groups but became better in the post-operative 6 months. There was no significant difference between the groups for both time points. The certainty of this evidence was considered very low (see Supplementary Materials E—Table S2, comparison E2.29 and E2.30).

*SOND vs. sentinel node biopsy (SNB)*

A cross-sectional study [62] compared SOND with sentinel node biopsy (SNB) in individuals with oral and oropharynx carcinoma. The authors reported that individuals who went through SND or SNB had a similar neck ROM in all directions. Neither group experienced a reduction in neck range of motion. The certainty of this evidence was considered very low (see Supplementary Materials E Table S2, comparison E2.31).

Mixed Neck Dissection vs. Others

*MRND and SND with removed cervical root branches vs. preserved cervical root branches*

A retrospective cohort study [84] compared MRND and SND with removed cervical root branches with MRND and SND with preserved cervical root in mixed HNC. The authors reported that individuals with MRND and SND who had the nerve removed had significantly reduced lateral neck flexion movement compared to the group with the nerve preserved (*p* < 0.05) even after more than 12 months of surgery. The certainty of this evidence was considered very low (see Supplementary Materials E—Table S2, comparison E2.32).

3. Jaw range of motion

Modified Radical Neck Dissection vs. Selective Neck Dissection

One prospective cohort study [52] investigated jaw movement in individuals with mixed HNC after undergoing SND or MRND with external beam radiation therapy (EBRT). This study reported that mouth opening was reduced among individuals with MRND combined with EBRT two months after surgery. No significant changes were observed in the SND group. The certainty of this evidence was considered very low (see Supplementary Materials E—Table S2, comparison E2.33).

#### 3.5.3. Muscle Strength Outcomes

The matrix table of the results for the muscle strength outcome is displayed in Supplementary Materials F—Table S7.

1. Shoulder muscle strength

Radical Neck Dissection vs. others

A cross-sectional study [67] and two prospective cohort studies [32,41] investigated shoulder muscle strength in this comparison group. The details of these studies are reported below.

*RND with sacrificed SAN vs. RND with preserved SAN*

One cross-sectional study [67] compared RND with the sacrificed SAN with RND with the preserved SAN in individuals with oral cancer. The authors reported that shoulder abductor strength using manual muscle testing (MMT) was significantly reduced in the sacrificed RND group compared with the preserved group even after 2 to 7 years of surgery. Eighteen patients in the sacrificed group had greater paresis, while only six patients in the preserved group had greater paresis in the study. The certainty of this evidence was considered very low (see Supplementary Materials E—Table S3, comparison E3.1).

*RND vs. MRND*

Two prospective cohort studies [32,41] evaluated shoulder muscle strength after undergoing RND and MRND in individuals with mixed HNC.

One study [32] reported no significant difference between RND and MRND in shoulder abductor and flexor muscle strength using MMT at 16 weeks post-operatively. Both groups showed reduced shoulder abductor and flexor strength at 16 weeks post-surgery. The certainty of this evidence was considered very low (see Supplementary Materials E—Table S3, comparison E3.2).

Additionally, Erisen et al. [41] reported that MMT for elevator and abductor muscles were significantly weaker in RND than MRND at 6 months follow-up (*p* < 0.01). However, the flexor muscle strength was similar between RND and MRND surgery (*p* > 0.05). Both groups had reduced their MMT score. The certainty of this evidence was considered very low (see Supplementary Materials E—Table S3, comparison E3.3).

*RND vs. SND*

Two prospective cohort studies [32,41] evaluated shoulder muscle strength in individuals with mixed head and neck cancer (HNC) within this comparison group. One study [32] reported that the results significantly differed (*p* < 0.05) between the RND and SND for abductor and flexor muscle strength. The SND group had better shoulder functions than RND at 16 weeks post-surgery. The certainty of this evidence was considered very low (see Supplementary Materials E—Table S3, comparison E3.4).

In addition, another prospective study [41] found that the shoulder joint’s elevator, flexor, and abductor muscles were weaker after RND than SND (*p* < 0.01) at 6 months of follow-up. The certainty of this evidence was considered very low (see Supplementary Materials E—Table S3, comparison E3.5).

MRND with pectoralis major myocutaneous flap (PMMF) vs. MRND without PMMF

A prospective cohort study [34] investigated MRND with and without a pectoralis major myocutaneous flap (PMMF) in individuals with oral carcinoma. They reported that MMT in flexion–extension, abduction–adduction, and internal and external rotation decreased in both groups at three months post-operatively. There was no statistically significant difference in muscle strength between the groups (*p* = 0.096). Both groups had a significant reduction in shoulder muscle strength. The certainty of this evidence was considered very low (see Supplementary Materials E—Table S3, comparison E3.6).

Selective Neck Dissection vs. others

A retrospective cohort study [82] and two prospective studies [27,33] investigated shoulder muscle strength in this comparison group. The details of these studies are reported below.

*SND (level IIb dissected) vs. SND (level IIb preserved)*

In a prospective cohort study [27], when comparing dynamometer scores between SND IIb dissected and SND IIb preserved in mixed HNC, the authors reported that both groups showed a reduction in shoulder elevators, flexors, and abductors at an early stage of follow-up (less than 3 months). No statistically significant differences between groups were found. The certainty of this evidence was considered very low (see Supplementary Materials E—Table S3, comparison E3.7).

*SND (level 2b spared bilaterally) vs. SND (level 2b spared unilaterally)*

A prospective cohort study [33] reported on comparing MMT of the shoulder muscles between SND (level 2b spared bilaterally) and SND (level 2b spared unilaterally) among individuals with laryngeal carcinoma. There were no statistically significant differences in scapular elevation, depression, and adduction muscle strength on day 21 and 6 months after surgery in both groups compared to the baseline measurements. The certainty of this evidence was considered very low (see Supplementary Materials E—Table S3, comparison E3.9).

*SND (level II–V) vs. SND (level II–IV)*

A retrospective cohort study [82] compared SND (level II–V) and SND (level II–IV) in mixed HNC. The authors reported that after at least one year of the surgery, decreased MMT and arm movement impairment were found in 25% of patients, with 25% showing a reduction in shoulder flexor muscle strength and 50% reduced the strength of shoulder abduction in the SND (level II–V) group. In contrast, only one patient (5%) in group SND (level II–IV) presented slight arm abduction impairment. The certainty of this evidence was considered very low (see Supplementary Materials E—Table S3, comparison E3.10).

Mixed Neck Dissection vs. Others

*Mixed ND (SND and MRND) with preserved SAN alone*

A cross-sectional study [55] evaluated shoulder strength and scapular muscle endurance using a dynamometer in mixed HNC after undergoing mixed ND (SND or MRND) with preserved SAN alone. They reported a decrease in strength for trapezius muscles (*p* = 0.001), serratus anterior (*p* = 0.001), infraspinatus/teres minor (*p* = 0.030), and supraspinatus (*p* = 0.001) at three months post-operatively compared to pre-operative measurements. The authors also reported that this group had decreased scapular muscle endurance (*p* = 0.008) at three months of follow-up compared to pre-operative measurement. The certainty of this evidence was considered very low (see Supplementary Materials E—Table S3, comparison E3.11).

2. Neck muscle strength

Selective Neck Dissection vs. others

Only two studies [33,76] investigated the strength of the neck muscles in HNC patients after neck dissection in this comparison group. The details of these studies are reported below.

*SND (level 2b spared bilaterally) vs. SND (level 2b spared unilaterally)*

A prospective cohort study [33] investigated neck flexors and extensors using MMT after undergoing SND (level 2b spared bilaterally) and SND (level 2b spared unilaterally) in mixed HNC. The authors reported that neck flexors and extensors muscle strength significantly decreased at 21 days and six months of follow-up for both groups, with no differences between them. The certainty of this evidence was considered very low (see Supplementary Materials E—Table S3, comparison E3.12 and E3.13).

*SND (preserved SAN) vs. MRND (preserved SAN)*

A cross-sectional study by Gane et al. [76] also examined the isometric strength of the cervical extensors and flexors using a dynamometer in mixed HNC. The authors found that both groups (SND and MRND with accessory nerve preservation) had decreased cervical flexors and extensor strength after more than 6 months of the surgery. There were no significant differences between SND and MRND in cervical spine muscles strength. The certainty of this evidence was considered very low (see Supplementary Materials E—Table S3, comparison E3.14).

3. Respiratory muscle strength

Mixed Neck Dissection: RND, MRND, and SND (stage I–VI neck dissection)

One prospective cohort study [44] evaluated the strength of the inspiratory muscles in mixed HNC after a stage I–VI neck dissection. The authors found that maximum inspiratory pressure (MIP) and nasal inspiratory pressure (SNIP) decreased significantly after dissection surgery (48 and 72 h post-surgery). However, after one month of the surgeries, MIP and SNIP returned to their pre-surgery levels. The certainty of this evidence was considered very low (see Supplementary Materials E—Table S3, comparison E3.15).

#### 3.5.4. Disability Questionnaire Outcomes

The matrix table of the results for the disability questionnaire outcome is illustrated in Supplementary Materials F—Table S8.

1. Shoulder disability

Radical Neck Dissection vs. others

Two cross-sectional studies [65,72], two retrospective cohort studies [87,88], and one prospective cohort study [45] investigated shoulder disability in this comparison group. Various outcomes were used to measure shoulder disability in this comparison group, such as the Shoulder Disability Questionnaire (SDQ) [72,88], the Shoulder Pain and Disability Index (SPADI) [65], The Clinical Assessment Score (CAS) [87], and the Shoulder Function and Performance Score (SFPS) [45]. The details of these studies are reported below.

*RND vs. MRND*

One cross-sectional [65], one retrospective cohort study [87], and one prospective cohort study [45] compared shoulder disability between RND and MRND in individuals with mixed HNC.

A prospective study [45] reported that the RND group showed a significantly larger reduction in Shoulder Function and Performance Score (SFPS) immediately after surgery when compared to the MRND group (*p* < 0.001). The certainty of this evidence was considered very low (see Supplementary Materials E—Table S4, comparison E4.1).

Additionally, in a retrospective cohort study [87], the authors reported that RND patients had significantly worse shoulder function in The Clinical Assessment Score (CAS) than the MRND patients after more than 6 months post-surgery. However, a cross-sectional [65] study reported no significant differences in SPADI scores between the two groups after more than 6 months of the surgery. The authors reported that approximately 35% of patients in the RND group (10/29) and 20.7% of patients in the MRND group (6/29) had high levels of shoulder pain and shoulder disability as determined by their SPADI scores after more than 6 months of surgery. The certainty of this evidence was considered very low (see Supplementary Materials E—Table S4, comparison E4.2).

*RND vs. MRND/SND*

*A* cross-sectional [72] and a retrospective cohort study [88] evaluated shoulder disability using the SDQ in individuals with mixed HNC.

A cross-sectional study [72] reported that no difference in SDQ score was found between the different types of neck dissections. However, the SDQ score in SND was lower than that of MRND and RND. RND showed the highest shoulder disability compared to the other two groups, even after more than 6 months of surgery.

Additionally, a retrospective cohort study [88] reported that SND/MRND had significantly lower disability scores in SDQ than the RND group, with an average mean of 10.90 ± 4.75 (*p* = 0.008) at 12 months of follow-up. The certainty of this evidence was considered very low (see Supplementary Materials E—Table S4, comparison E4.3).

Modified Radical Neck Dissection vs. Others

Three cross-sectional studies [60,66,68], one retrospective cohort study [79], and four prospective cohort studies [26,34,38,54] investigated shoulder disability in this comparison group. Two prospective cohort studies [34,38] investigated shoulder disability in patients with oral cavity cancer, and the remaining studies included patients with mixed HNC. Various outcomes were used to measure shoulder disability in this comparison group, such as the Shoulder Disability Questionnaire (SDQ) [79], the Shoulder Pain and Disability Index (SPADI) [26,54], the Constant–Murley Score (CSM) [54,66,68], the Disability of the Arm, Shoulder and Hand (DASH) [60] and the University of Washington Quality-of-Life (UW-QOL) [38]. The details of these studies are reported below.

*MRND (nerve monitored) vs. (MRND non-monitored)*

A prospective cohort study [54] evaluated shoulder disability using CSM and SPADI in this comparison group. The authors reported no significant differences when comparing the MRND (monitored) with the MRND (non-monitored) side in individuals with mixed HNC. Both groups had decreased (worse) CSM and increased (worse) SPADI scores six weeks after surgery and further worsening of shoulder symptoms at six months post-surgery. The certainty of this evidence was considered very low (see Supplementary Materials E—Table S4, comparison E4.4).

*MRND vs. SND*

A prospective cohort study [26] and three cross-sectional studies [60,66,68] investigated shoulder disability after MRND and SND in individuals with mixed HNC.

A prospective cohort study [26] reported that the SPADI scores for both groups were significantly worse at 1 week, 1 month, and 3 months post-operatively compared to baseline values in both the MRND and SND groups. However, when compared between the groups, the MRND group demonstrated significantly worse SPADI scores at 1 week, 1 month, and 3 months post-operatively compared to the SND group (*p* = 0.01). The certainty of this evidence was considered very low (see Supplementary Materials E—Table S4, comparison E4.5).

Two cross-sectional studies [66,68] indicated that the MRND group exhibited a lower CMS score at 12 months post-surgery (MRND; mean = 68 [10–88], SND; mean = 85 [12–100], *p* = 0.004) [66] and at 22–44 months post-surgery (MRND; mean= 62.8; SND; mean = 80.1) [68] in comparison to the SND group. Furthermore, another cross-sectional study [60] reported that patients in the MRND group demonstrated a worse DASH score (higher scores indicating worse outcomes) one year after surgery compared to the SND group (MRND; MD = 22; SND; MD = 17). The certainty of this evidence was considered very low (see Supplementary Materials E—Table S4, comparison E4.6).

*MRND vs. SOND*

A retrospective cohort study [78] and a prospective cohort study [38] examined shoulder disability in patients who underwent MRND compared to SOND.

A prospective cohort study [38] revealed that individuals undergoing MRND exhibited lower shoulder function scores immediately following surgery for the oral cavity, as assessed by the UW-QOL tool, compared to those undergoing SOND (MRND; M = 68.1, SOND; M = 81.1) with *p* < 0.001. The certainty of this evidence was considered very low (see Supplementary Materials E—Table S4, comparison E4.7).

Furthermore, a retrospective cohort study [78] indicated that the MRND group had a lower SDQ score than the SOND group in mixed HNC (MRND; MD = 22.2, SD = 28.6; SOND MD = 11.6, SD = 26.1) (*p* < 0.01) at more than one year post-surgery. The certainty of this evidence was considered very low (see Supplementary Materials E—Table S4, comparison E4.8).

*MRND with a pectoralis major myocutaneous (PMMC) flap vs. MRND without PMMC flap*

A prospective cohort study [34] compared MRND with a PMMC flap and MRND without a PMMC flap in individuals diagnosed with oral cancer. Both groups exhibited lower SDQ scores at three months post-surgery. The authors reported that, in the MRND with PMMC flap group, 57.5% of patients experienced shoulder disability according to the SDQ scores in the third month, with this figure decreasing to 33.8% in the sixth month following physiotherapy intervention. In contrast, among patients undergoing MRND without the PMMC flap, 54.8% reported shoulder disability at three months, which decreased to 28.1% at six months after physiotherapy intervention. No significant differences were found between the groups. The certainty of this evidence was considered very low (see Supplementary Materials E—Table S4, comparison E4.9).

Selective Neck Dissection alone or vs. others

*SND alone*

Four studies [31,35,43,77] were conducted to investigate shoulder disability in this group. Two are from a retrospective cohort study [77], and three are from a prospective cohort study [31,35,43]. These studies encompassed a diverse range of individuals with HNC, including studies with various types of HNC (mixed HNC) [35], nasopharyngeal carcinoma [31], tongue cancer [43], and oral cancer [77].

A recent retrospective cohort study [77] reported that individuals with oral cancer who underwent SND had lower scores in the SDQ and SPADI after 1 and 6 months of surgery. They also highlighted that younger patients undergoing SND scored better on the SDQ and SPADI compared to older patients with oral cancer after 1 and 6 months of surgery. Furthermore, a prospective cohort study [35] revealed that patients who underwent SND achieved a moderate score of 60.4 on the Western Ontario Rotator Cuff (WORC) questionnaire one month post-surgery. Over time, these patients demonstrated improvement, scoring 72.4 at nine months post-operatively.

Additionally, another prospective cohort study [31] reported that the majority of patients with nasopharyngeal carcinoma who underwent SND experienced moderate shoulder disability on the Disability of the arm, shoulder, and hand (DASH) at both one and two years following the procedure. However, contradicting findings were reported by a prospective cohort study [43], where the authors reported no significant difference in the shoulder component of UW-QOL among patients with tongue cancer at more than one year post-surgery when compared to the pre-measurement with MD = 6.92 (CI 95% 0.29 to 14.13), *p*= 0.06. The certainty of this evidence was considered very low (see Supplementary Materials E—Table S4, comparison E4.10 and E4.11).

*SND vs. non-surgery*

One study [89] evaluated the shoulder domain of UWQOL in individuals with mixed HNC with no surgery group. The authors reported that SND showed a lower score (worse score) in the shoulder domain of UW-QOL compared to the non-surgery group after two years of surgery. The certainty of this evidence was considered very low (Supplementary Materials E—Table S4, comparison E4.12)

*SND vs. FND*

Two prospective cohort studies [37,48] investigated shoulder disability in laryngeal [48] and tongue cancers [37] within this comparison group. Selcuk et al. [48] reported significant differences in SPADI scores, indicating better outcomes in the SND (anterolateral) group compared to the FND group among individuals with laryngeal cancers 6 months after the surgery. Additionally, another prospective study [37] revealed that individuals with tongue cancers who underwent FND exhibited significantly greater severity in SPADI and Groningen Activity Restriction Scale (GARS) scores compared to those who underwent SND (levels I, II, III) after more than 6 months post-surgery, particularly during activities such as dressing, hair washing, performing heavy household tasks, and washing dishes and clothes. The certainty of this evidence was considered very low (see Supplementary Materials E—Table S4, comparison E4.13).

*SND vs. SND with radiotherapy/chemoradiation*

A cross-sectional study [63] was conducted to investigate shoulder disability in individuals diagnosed with mixed HNC, specifically comparing shoulder function across three treatment groups (SND alone or in combination with radiotherapy or chemoradiation) after more than 6 months of surgery. The authors reported no statistically significant differences in the total Constant–Murley Score (CMS) among the groups (*p* = 0.16). The mean CMS values were (84 ± 5) for patients who underwent SND, (71 ± 4) for those who received SND in conjunction with radiotherapy, and (77 ± 4) for those treated with SND and chemoradiation therapy. Notably, all groups exhibited a decline in shoulder function. The certainty of this evidence was considered very low (see Supplementary Materials E—Table S4, comparison E4.14).

*SND unilateral level V vs. bilateral level III to IV dissections*

A prospective cohort study [39] examined shoulder disability in individuals diagnosed with oropharyngeal carcinoma in this comparison group. The authors reported that individuals who underwent SND involving unilateral level V and bilateral levels III to IV dissections exhibited worse scores in the shoulder disability domain of the UW-QOL questionnaire even after more than six months post-surgery. Nonetheless, the differences observed between the groups were not statistically significant (*p* = 0.66). The certainty of this evidence was considered very low (see Supplementary Materials E—Table S4, comparison E4.15).

Supraomohyoid Neck Dissection (SOND) vs. others

Four studies investigated shoulder disability in individuals with mixed HNC [62,80] and oral cancers [61,90] within the comparison group. This comparison group included two cross-sectional studies [61,62], one retrospective cohort study [80], and one randomized controlled trial (RCT) [90]. The details of the studies are described below:

*SOND vs. Sentinel Node Biopsy (SNB)*

A cross-sectional study [62] was conducted to compare SOND and SNB in individuals with mixed HNC. The authors reported that patients in the SNB group demonstrated significantly higher scores on the Constant–Murley Score (CMS) (Mean = 90.3) compared to those in the SND group (Mean = 82.47; *p* = 0.043). These findings indicate better patient-reported symptom scores, active shoulder function scores, and improved post-operative shoulder function in the SNB cohort after the surgery. The certainty of this evidence was considered very low (see Supplementary Materials E—Table S4, comparison E4.16).

*SOND vs. MISOND (minimally invasive supraomohyoid neck dissection)*

A cross-sectional study [61] was conducted to compare open supraomohyoid neck dissection (SOND) with minimally invasive supraomohyoid neck dissection (MISOND) in individuals diagnosed with mixed HNC. The authors reported that the mean SPADI score at six weeks post-operatively was significantly more favorable in the MISOND group (Mean = 14.35 ± 0.71%) compared to the SOND group (Mean = 44.14 ± 1.18%) (*p* = 0.001). The certainty of this evidence was considered very low (see Supplementary Materials E—Table S4, comparison E4.17).

*SOND vs. extended SOND and lateral neck dissection*

A retrospective cohort study [80] was conducted to evaluate DASH in the SOND group and extended SOND and lateral neck dissection group within mixed HNC. The authors reported that the mean DASH scores were 25.1–25.9 (range 0–97.5) for the SOND group, 15.7–16.2 (range 0–46.4) for the extended SOND group, and 11.9–15.0 (range 0–45.3) for the lateral neck dissection group. These scores suggest that all groups experienced minor disability after more than one year post-surgery. The certainty of this evidence was considered very low (see Supplementary Materials E—Table S4, comparison E4.18).

*SOND vs. mixed ND (SND/MRND/RND)*

An RCT study [90] was conducted to compare SOND with a mixed neck dissection (ND) group in individuals diagnosed with oral cancer. The authors reported that, at both one- and three-months post-surgery, individuals in the SOND group exhibited significantly superior scores in the Constant–Murley Score (CMS) and the shoulder domain of the UW-QOL (*p* < 0.05) than the mixed ND (SND/MRND/RND). The certainty of this evidence was considered very low (see Supplementary Materials E—Table S4, comparison E4.19).

Mixed Neck Dissection vs. others

*Mixed ND (SND and MRND (preserved SAN) alone*

A cross-sectional study [55] evaluated shoulder disability using CMS after SND or MRND with preserved SAN in individuals diagnosed with mixed HNC. The authors reported that most of the individuals undergoing this surgery showed lower CMS scores at three months post-operatively when compared to pre-operative measurement (*p* < 0.05). The certainty of this evidence was considered very low (see Supplementary Materials E—Table S4, comparison E4.20).

*Mixed ND with PMMC vs. without PMMC*

Two prospective cohort studies [36,51] were conducted to compare ND with PMMC and ND without PMMC in individuals with oropharyngeal cancer [36] and mixed HNC [51]. Both groups had a significantly higher DASH score (*p* < 0.001) [36] and a low CMS six months after surgery [51] than the pre-operative score. The certainty of this evidence was considered very low (see Supplementary Materials E—Table S4, comparison E4.21).

2. Shoulder and neck disability

Radical Neck Dissection vs. Functional Neck Dissection

A prospective cohort study [47] was conducted to evaluate shoulder and neck disability in this comparison group using the Neck Dissection Impairment Index (NDII) among individuals with mixed HNC. The authors reported that at three and nine months post-surgery, the FND group exhibited significantly lower scores on pain, neck and shoulder stiffness, and disability in lifting heavy objects, light objects, and reaching overhead compared to the RND group (*p* < 0.001). The certainty of this evidence was considered very low (see Supplementary Materials E—Table S4, comparison E4.22).

Modified Radical Neck Dissection vs. Selective Neck Dissection

Two cross-sectional studies [60,66] evaluated shoulder and neck function utilizing the Neck Dissection Impairment Index (NDII) in individuals with mixed HNC. Both studies revealed that patients who underwent MRND significantly exhibited lower NDII scores in comparison to those who underwent SND after more than one year following surgery. The certainty of this evidence was considered very low (see Supplementary Materials E—Table S4, comparison E4.23).

Selective Neck Dissection (levels 2a–4 included dissection of level 2b) vs. Selective Neck Dissection (levels 2a–4 without dissection of level 2b)

An RCT [91] assessed NDII scores among individuals with mixed HNC who underwent SND that included dissection of level 2b, in comparison to SND (levels 2a–4 without dissection of level 2b). The difference between six-month post-operative and pre-operative scores was statistically significant for both groups (SND with spared 2b, *p* = 0.002; SND with 2b dissected, *p* = 0.001). Both groups had lower (worse) scores in NDII. Furthermore, the SND group that included level 2b dissection showed significantly lower (worse) NDII scores than the group that did not include level 2b dissection (*p* = 0.008). The certainty of this evidence was considered very low (see Supplementary Materials E—Table S4, comparison E4.24).

3. Neck disability

Modified Radical Neck Dissection vs. Selective Neck Dissection

A cross-sectional study [60] examined neck disability in mixed HNC using the Neck Disability Index (NDI). The authors found that patients who underwent MRND exhibited slightly higher NDI scores (worse) (Mean = 21, SD = 26) (not significantly different) than those in the unilateral SND group (Mean = 16, SD = 13) after more than one year post-surgery. The certainty of this evidence was considered very low (see Supplementary Materials E—Table S4, comparison E4.25).

Mixed Neck Dissection (spared CN XI) vs. without ND

A prospective cohort study [50] was conducted to compare individuals with laryngeal who underwent neck dissection with cranial nerve XI preservation (MRND/SND) with those who did not undergo neck dissection. The authors reported that, after one to four years post-surgery, the neck dissection group exhibited significantly poorer scores on the Neck Pain and Disability Scale (NPDS) (*p* = 0.00) and the Northwick Park Neck Pain Questionnaire (NPNPQ) (*p* < 0.05) in comparison to the non-neck dissection group (see Supplementary Materials E—Table S4, comparison E4.26).

## 4. Discussion

This comprehensive systematic review examined and synthesized the results from 67 studies focusing on neuromusculoskeletal impairments after neck dissection surgery in patients with head and neck cancer. This review has identified a substantial degree of heterogeneity in the studies pertaining to the neuromusculoskeletal impairments seen in individuals after HNC surgery involving a neck dissection procedure. The observed variability can be attributed to several factors, encompassing the inclusion of different patient cohorts, different types of surgical techniques and protocols, variations in the durations of post-operative follow-up, a wide range in tumor types, differences in the chosen outcome assessments, as well as disparities in the range of co-interventions administered after surgery (such as chemotherapy, radiotherapy, chemoradiation, and rehabilitation).

Although there is substantial variability among the studies included in this review, a reliable association has been established between neck dissection surgeries and neuromusculoskeletal impairments, particularly concerning the shoulder. Shoulder-related outcomes such as pain, loss of active ROM, and shoulder disability have emerged as the most frequently reported outcomes across the included studies. Moreover, this review highlights additional musculoskeletal concerns after neck dissection procedures, including reduced strength in both shoulder and neck muscles, decreased neck ROM, increased neck disability, and respiratory dysfunction.

### 4.1. Shoulder Dysfunction

Individuals with head and neck cancer who underwent surgery with RND exhibit elevated neuromusculoskeletal dysfunction, including shoulder pain and decreased shoulder and neck ROM. Additionally, their scores on disability/dysfunction questionnaires were notably worse when compared to patients who underwent other neck dissections, such as MRND or SND. Following RND, patients frequently experience prolonged and substantial limitations in their ability to engage in daily activities, with severity ranging from moderate to severe, significantly impeding their daily functioning. These results have similar findings to a previous systematic review [10] reporting that the prevalence of shoulder pain was slightly higher after RND when compared to MRND and markedly higher when compared to SND.

Following neck dissection, shoulder dysfunction in the head and neck cancer patients often results from nerve damage during the surgical procedure. As stated in a recent review [11], the prevalence of spinal accessory nerve (SAN) injuries varies depending on the type of neck dissection performed. RND is known to have the highest estimated prevalence of SAN injuries at 94.8%, followed by MRND at 33.0% and SND at 26.7% [11]. Distinguishing between these types of neck dissections is critical to understanding the different risks associated with each procedure. RND involves the removal of lymph nodes and surrounding tissue in cancers of the head and neck and the sacrifice of the SAN, internal jugular vein, and sternocleidomastoid muscle [11,87]. Although MRND and SND are less extensive than RND, they still pose a significant risk to the SAN [64,76,91]. It is worth noting that although the prevalence of injury is lower in SND procedures, SAN damage can still occur due to manipulation of the nerve during the lymph node dissection procedure [62].

The high prevalence of SAN injuries in the context of neck dissection (ND) has important implications for rehabilitation. The consequences of SAN injuries can go beyond immediate post-operative complications. Since the SAN is responsible for the motor innervation of the sternocleidomastoid and trapezius muscles, any injury to this nerve can influence the positioning and movement of the scapula, potentially leading to shoulder impingement, decreased strength when raising the arm, having difficulties with tasks involving lifting or reaching, and limiting shoulder abduction and flexion range of motion [10,75,95]. Moreover, the changes in biomechanical function between the scapula and shoulder joint contribute to the pain and dysfunction of the shoulder joint, significantly affecting their daily activities and overall QoL in HNC survivors [60,75,95].

Surgical interventions can have adverse effects on muscles such as the sternocleidomastoid and scalene muscles, thereby impacting inspiratory strength and reducing lung volumes, ultimately leading to hypoxemia and insufficient cough strength [44]. The deterioration of respiratory function in patients with neck dissection can increase the risk of pulmonary complications and overall mortality [44,96]. These factors warrant careful consideration in the context of patient care and recovery.

This review also highlighted the presence of variability in the existing literature concerning shoulder impairments and function after MRND and SND. Such variability could be attributed to the heterogeneity of the included studies, including variations in HNC criteria, diverse comparative groups, different surgery time frames, and the use of various measures to assess the same outcomes [97]. These factors may contribute to the diverse findings reported across the studies. However, even among patients undergoing MRND and SND, impairments frequently arise, potentially leading to the development of neck stiffness, abnormal head posture, and excessive kyphosis of the upper trunk. These conditions may subsequently result in musculoskeletal pain and interference with respiratory function [65].

It is also crucial to acknowledge the functional constraints linked to local flap repair, especially the PMMF, because of their potential long-term effects on patient well-being [98]. A recent review [99] indicated that patients receiving routine PMMF frequently encounter diminished shoulder range of motion and strength, leading to significant functional disability in performing daily tasks such as lifting, dressing, and doing overhead activities. These physical impairments not only diminish independence but may also exacerbate psychological distress and psychosocial challenges, particularly among individuals who depend significantly on upper extremity function for their livelihood [98,99,100].

### 4.2. Neck Dysfunction

Numerous studies have investigated various aspects of neck outcomes, including cervical joint ROM, cervical muscle strength, and neck disability following neck dissection. While the available evidence is still limited and primarily focused on SND, it still provides valuable insights into the significance of post-operative care for the neck region. In a previous review [10], it was highlighted that a notable percentage of patients (ranging from 1% to 13%) experience limited neck active ROM following neck dissection. This could result from the injury or extraction of various soft tissues, including nerves, muscles, fascia, lymphatic vessels, and veins within the neck during the surgery [76].

Additionally, the incidence of neck pain was found to be higher when the cervical plexus was sacrificed during the neck dissection procedure. Furthermore, research has demonstrated that patients who underwent SND and MRND with preserved SAN still show decreased cervical extensor and flexor muscle strength [76]. The surgical interference with structures like the sternocleidomastoid muscle and nerves during neck dissection can contribute to this dysfunction, resulting in long-term consequences for patients [10]. Furthermore, individuals with HNC who encounter neck dysfunction after neck surgeries and dissection may also have challenges with swallowing, speech, eating, and respiratory functions [12,101,102], and the changes may negatively impact body image and overall well-being [101]. All of these can contribute to further physical, psychological, and socioeconomic burdens [12,103]. Moreover, these factors play a crucial role in individuals’ comprehensive recovery and rehabilitation after the surgeries [12,101,102]. Thus, by addressing these issues early on and implementing appropriate rehabilitation interventions, healthcare professionals can significantly improve patients’ post-operative outcomes and overall well-being after neck dissection surgery.

### 4.3. Other Neuromusculoskeletal Dysfunctions

Among the studies reviewed, only one study [52] addressed the issue of reduced jaw movement and mouth opening (i.e., trismus) after MRND combined with EBRT at two months post-surgery. The consequences of jaw stiffness and mouth opening reduction are considerable, significantly affecting jaw function and oral functions such as eating and talking. Patients may experience reduced food intake, difficulties maintaining oral hygiene, and challenges undergoing necessary dental procedures [16]. These limitations can lead to increased social isolation and, ultimately, a decline in the overall quality of life and mental well-being of HNC survivors [16]. It is essential to acknowledge that the available evidence related to jaw functions, specifically after the surgery with neck dissection, remains limited. Although the chances of developing trismus after neck dissection are lower, the risk is still there [52].

Individuals with head and neck cancer often experience compromised respiratory function due to tumor burden, surgical interventions, or radiation therapy, which can affect diaphragm integrity and function [104]. A prospective study [44] was conducted to investigate the impact of stage I–VI neck dissection surgery (mixed ND) on individuals with head and neck cancer. The study revealed that these patients experienced a decline in maximal inspiratory pressure (MIP) after surgery, and the atrophy of the diaphragm was found one month after the procedure. This finding suggests that diaphragm atrophy in these individuals could potentially result in respiratory complications, diminished exercise tolerance, and a higher risk for pneumonia [105]. Ultimately, these complications can profoundly influence the overall prognosis and quality of life of affected individuals [105]. However, it is important to acknowledge that the existing evidence on this subject is still limited, and the findings of the study remain inconclusive.

### 4.4. Methodological and Quality of Evidence

The risk of bias assessment employed the ROB 2 tool for randomized controlled trials (RCTs) and the ROBIN-I tool for non-randomized studies. To the best of our knowledge, this study represents the first instance where the ROBIN-I tool has been utilized to evaluate the risk of bias in non-randomized studies within a systematic review pertaining to neck dissection. Regarding RCTs, one study [92] exhibited a high risk of bias in terms of allocation concealment (performance bias) and blinding of participants and personnel (detection bias). The absence of blinding during the assessment could potentially impact the findings of this study. Trials lacking sufficient randomization, allocation concealment, and blinding tend to report treatment effects that may deviate from reality compared to trials incorporating these features [106,107]. However, considering that the neck dissection intervention was being investigated, blinding was not feasible. Moreover, this systematic review encompasses only four RCTs that specifically examined neuromusculoskeletal outcomes after the neck dissection. Given the limited number of trials, it is essential to interpret the results cautiously for objective measures and subjective self-reported outcomes. In non-randomized studies, only two cross-sectional studies [75,76] exhibited a moderate overall bias risk, primarily attributable to confounding factors and deviations from the intended intervention. Many studies failed to provide detailed descriptions of their interventions and did not adequately control co-interventions and confounding variables. These issues are particularly relevant to this study since it remains unclear whether the observed effects on selected outcomes were solely due to the intervention itself or influenced by other factors. Although controlling confounding or co-intervention is crucial, it is challenging to achieve in cohort studies, especially among head and neck cancer patients undergoing neck dissection.

Various studies reveal significant variability in the definitions and measurements of neuromusculoskeletal impairments. Commonly reported issues include shoulder and neck dysfunctions, pain, and muscle weakness; however, the outcome measures used and the timing of assessments differ widely. This inconsistency hinders the ability to compare findings and draw definitive conclusions about the prevalence and progression of impairments following neck dissection in individuals with head and neck cancer. To strengthen the evidence base related to this field of research, future studies should adopt standardized definitions and validated outcome measures, utilize rigorous methodologies regardless of study design, and comply with appropriate reporting standards (e.g., STROBE for observational studies, CONSORT for randomized controlled trials) [108,109]. High-quality prospective studies are particularly crucial for accurately characterizing the trajectory and clinical impact of neuromusculoskeletal impairments in this population.

### 4.5. Strengths and Limitations of This Review

As mentioned previously, the studies analyzed in this review exhibited substantial heterogeneity in terms of their design, study population, primary cancer site, stage of cancer, the types of cancer treatments employed, and the group comparison. Furthermore, the methods and timelines utilized to measure neuromusculoskeletal impairments demonstrated inconsistencies, with varying instruments for each outcome measurement. Additionally, the included studies encompassed a mixture of patients with diverse types of neck and head cancer, including nasopharynx, oral cavity, oropharynx, hypopharynx, and larynx cancer. Furthermore, the lack of clear descriptions and inconsistencies in the use of terminologies for the types of neck dissections across different studies posed challenges in generating pooled effect estimates through meta-analyses. Despite these limitations, this review comprehensively synthesizes the available evidence on neuromusculoskeletal impairments and dysfunctions following neck dissection surgeries. Thus, it offers valuable insight for the clinicians and researchers in this specific research area.

### 4.6. Clinical Relevance and Rehabilitation

This review reveals a significant prevalence of musculoskeletal impairments in individuals with HNC following the surgery with ND, particularly affecting shoulder and neck regions. Despite the variability in surgery, cancer group types, and outcome measures, the evidence consistently indicates a reduction in range of motion, muscle weakness, and postural dysfunction, which adversely affect activities of daily living and overall quality of life in individuals with HNC [13,75,95]. These findings highlight the necessity of incorporating targeted rehabilitation programs into the pre and postoperative care plan.

Most rehabilitation interventions for individuals with HNC following the surgery with ND have primarily focused on shoulder function, restorative swallowing therapy, and whole-body conditioning [110,111,112]. Although trismus is a frequently reported complication in individuals with HNC after the surgery, the involvement of physical therapy in high-quality research regarding its management remains limited [113,114]. Similarly, despite the high prevalence of neck dysfunction following neck dissection, rehabilitation strategies specifically targeting neck function are insufficiently studied [110,115,116].

Given the complexity of musculoskeletal impairments within individuals with HNC following the surgery, a comprehensive rehabilitative strategy may provide the most significant benefits in this population. Interventions such as structured physiotherapy, manual therapy techniques, and minimally invasive modalities (e.g., transcutaneous electrical nerve stimulation dry needling) could enhance therapeutic outcomes [116]. Furthermore, to mitigate the long-term morbidity associated with HNC, interdisciplinary collaboration within the rehabilitation field is needed to provide more comprehensive and patient-centered care by addressing the complex and functional limitations that emerge throughout the cancer journey.

### 4.7. Clinical and Research Implications

This review provides insight into the existing literature and highlights the need for high-quality research on the impact of neck dissection on neuromusculoskeletal function. The findings indicate the need for specialized pre- and post-operative rehabilitation programs for patients with HNC to address and minimize any potential negative consequences resulting from the surgery following neck dissection. A significant evidence gap was identified regarding the methodological quality of studies. As patients may experience one or more impairments, there is a need for agreement on a core outcome set to facilitate data collection and better characterize impairments across multiple upper body regions.

## 5. Conclusions

The studies included in this review exhibit a lack of methodological consistency, primarily due to their retrospective nature and the inherent discrepancies in pre-operative and post-operative characteristics between the groups in certain studies. These limitations should be acknowledged when considering the results of this review. Furthermore, it is crucial to note that studies focusing on neuromusculoskeletal outcomes such as shoulder and neck muscle strength are still limited, and most existing studies carry a significant risk of bias. While the conclusions should be approached with caution, it is reasonable to infer that neck dissection procedures contribute to neuromusculoskeletal impairment and dysfunction in patients with HNC. Moreover, advancing research in this field requires optimizing study designs, standardizing assessment methods, and establishing consistent outcome measures for neuromusculoskeletal impairments and dysfunctions. Future research is needed to address these limitations and provide more comprehensive insights into the long-term neuromusculoskeletal effects of neck dissection surgeries in individuals with head and neck cancer.

## Figures and Tables

**Table 1:** Summary of eligibility criteria based on the PICOS framework.

Component	Details
Population (P)	Individuals diagnosed with head and neck cancer who underwent surgical treatment, including neck dissection.
Intervention (I)	Studies reporting musculoskeletal impairments and dysfunctions following surgery with neck dissection
Comparison (C)	Not mandatory; some studies may include comparisons (e.g., affected vs. unaffected side, pre- vs. post-surgery, or control group), but comparison was not required for inclusion
Outcomes (O)	Musculoskeletal impairments and dysfunctions such as shoulder pain and dysfunction (e.g., limited range of motion, weakness), neck pain and dysfunction (e.g., limited range of motion, weakness), functional limitations related to activities of daily living
Study Design (S)	RCTs, observational studies, including cohort and cross-sectional); published in English.

**Table 2:** Descriptions of included studies (N = 67).

Country	n (%)	Publication Date		n(%)
India Turkey USA United Kingdom Netherland China Italy Canada Japan Australia German Brazil Korea Taiwan Sweden Others (Switzerland, Hongkong, Ireland, Macedonia, Slovenia	9 (13) 8 (12) 6 (9) 6 (9) 6 (9) 4 (6) 4 (6) 3 (4.5) 3 (4.5) 3 (4.5) 2 (3) 2 (3) 2 (3) 2 (3) 2 (3) 5 (7.5)	After 2016 2010–2015 2000–2009 1985–1999 **Study Design** RCT Cross-sectional Retrospective Cohort Prospective Cohort **Gender** Mixed		23 (34) 17 (26) 24 (36) 3 (4) **n (%)** 4 (6) 21 (32) 13 (19) 29 (43) 67 (100)
**Head and Neck Cancer (HNC) Diagnosis**				
Mixed HNC Oral, Tongue, and Oropharynx Larynx	43(65) 14 (21) 4 (6)	Nasopharyngeal Not reported		1(2) 4(6)
**Types of Neck Dissection**				
Radical Neck Dissection (RND) Modified Radical Neck Dissection (MRND) Selective Neck Dissection (SND) Elective/Functional Neck Dissection (END/FND)	10 17 27 3	Mixed Neck Dissection (preserved/removed Cervical nerve root) SND/MRND with Reconstruction		8 1
**Musculoskeletal Impairments**				
**Pain** Shoulder Neck Myofascial muscle pain **Range of Motion** Shoulder Joint Cervical Joint Jaw **Strength** Shoulder Muscles Neck Muscles Respiratory Muscles	11/67 5/67 1/67 26/67 9/67 1/67 10/67 2/67 1/67	**Muscle Activation** Trapezius Muscle Sternocleidomastoid **(SCM)** **Muscle volume** Trapezius **Musculoskeletal disability** Shoulder disability Shoulder and neck disability Neck disability **Other** Posture		6/67 2/67 1/67 32/67 5/67 3/67 1/67
**Outcome measure tools**				
**Pain** VAS (11/67) HNQOL (2/67) UWQOL (1/67) **Range of Motion** Goniometer (22/67) Inclinometer (7/67) Tape measurement (1/67) Ruler (1/67) **Posture (1/67)** **Muscle Activation** EMG (6/67)	**Muscle Volume** CT scan (1/67) Ultrasound (1/67) **Strength** Dynamometer (4/67) Manual muscle test (7/67) Isokinetic (1/67) Micro RPM (1/67) **Musculoskeletal disability** Neck Disability - NPNP (1/67) - NPDS (1/67) - NDI (2/67)		Shoulder disability - CMS (8/67) - SDQ (8/67) - UWQOL (5/67) - DASH (4/67) - SPADI (6/67) - GARS (1/67) - SFPS (1/67) - WORC (1/67) Shoulder and Neck Disability - NDII (5/67)	

NDII: Neck Dissection Impairment Index, VAS: Visual Analogue Scale, Micro RPM: Micro direct respiratory pressure meter, CMS: Constant–Murley Score, SDQ: Shoulder Disability Score, UWQOL: University of Washington Quality of Life, DASH: The Disability of Arm, Shoulder, and Hand, SPADI: Shoulder Pain and Disability Index, NPNPQ: Northwick Park Neck Pain Questionnaire, NPDS: Neck Pain and Disability, HNQOL: Head and neck quality of life instrument, GARS: Groningen activity restriction scale, NDI: Neck Disability Index, SFPS: Shoulder Function and Performance Score, WORC: Western Ontario Rotator Cuff questionnaire, EMG: Electromyography, CT scan: Computed Tomography (CT) Scan.

## Data Availability

Data can be available upon request to the authors.

## Supplementary Materials

The following supporting information can be downloaded at: https://www.mdpi.com/article/10.3390/life15050800/s1, Supplementary A: Search strategy of the studies. Supplementary B: List of the excluded studies. Supplementary C: The summary of the included studies. Supplementary D: Summary of the risk of bias across the studies. Supplementary E: GRADE table for included studies. Supplementary F: Matrix table of the results.

## Abbreviations

HNC	Head and neck cancer
HPV	Human papillomavirus
QOL	Quality of life
ND	Neck dissection
RND	Radical neck dissection
MRND	Modified radical neck dissection
SND	Selective neck dissection
SOND	Supraomohyoid neck dissection
END	Extended neck dissection
FND	Functional neck dissection
MISOND	Minimally invasive supraomohyoid neck dissection
SAN	Spinal accessory nerve
HRQOL	Health related quality of life
VAS	Visual analogue scale
UW-QOL	University of Washington Quality of Life questionnaire
EC	Electrocautery
HS	Harmonic scalpel
AAT	Arm abduction test
ADLs	Activities of daily living
PMMC	Pectoralis major myocutaneous flap
ROM	Range of motion
MMT	Manual muscle testing
SDQ	Shoulder Disability Questionnaire
SPADI	Shoulder Pain and Disability Index
CAS	Clinical Assessment Score
SFPS	Shoulder Function and Performance Score
DASH	Disability of the Arm, Shoulder and Hand
WORC	Western Ontario Rotator Cuff
GARS	Groningen Activity Restriction Scale
NDI	Neck Disability Index
NPDS	Neck Pain and Disability Scale
NPNPQ	Northwick Park Neck Pain Questionnaire
PMMF	Pectoralis major myocutaneous flap
