# Supplementary material for: Musculoskeletal Impairments and Dysfunction in Individuals with Head and Neck Cancer Following Surgery with Neck Dissection—A Systematic Review
Source: Life (Basel). 2025 May 17;15(5):800. doi: 10.3390/life15050800 (PMC12112850; doi:10.3390/life15050800)
Supplement: Supplementary file 1 [file life-15-00800-s001.zip › Supplementary_Materials_A.pdf]

SUPPLEMENTARY MATERIALS A

CINAHL Plus with Full Text (EBSCO Interface)
From 1946 to October 2024
Number of results: 2178
Search saved as: HNC systematic review

S1 (MH "Head and Neck Neoplasms") OR (MH "Squamous Cell Carcinoma of Head and Neck") OR (MH "Mouth Neoplasms+") OR (MH "Neoplasms, Squamous Cell+") OR (MH "Nose Neoplasms+") OR (MH "Tongue Neoplasms") OR (MH "Oropharyngeal Neoplasms+")
S2 (MH "Otorhinolaryngologic Neoplasms+")
S3 ((head or neck or mouth or gingival or lip or lips or palatal or palate* or salivary or tongue or oral or otorhinolaryngol* or throat or ear or nose or nasal or sinus or paranasal or pharyng* or oropharyng* or tonsil* or nasopharyng* or hypopharyng* or laryng*) N5 (cancer* or neoplasm* or carcinoma* or tumor* or tumour* or malignan*))
S4 S1 OR S2 OR S3
S5 (MH "Jaw Diseases") OR (MH "Jaw Abnormalities") OR (MH "Joint Diseases") OR (MH "Arthralgia") OR (MH "Shoulder Pain") OR (MH "Contracture") OR (MH "Joint Instability+") OR (MH "Shoulder Impingement Syndrome") OR (MH "Temporomandibular Joint Diseases+") OR (MH "Musculoskeletal Abnormalities") OR (MH "Craniofacial Abnormalities")
S6 (MH "Shoulder Pain") OR (MH "Nociceptive Pain+") OR (MH "Neck Pain") OR (MH "Postoperative Pain") OR (MH "Referred Pain") OR (MH "Muscle Pain") OR (MH "Myofascial Pain Syndromes+") OR (MH "Facial Pain+") OR (MH "Temporomandibular Joint Syndrome") OR (MH "Shoulder Dislocation") OR (MH "Arm Injuries+") OR (MH "Adhesive Capsulitis+") OR (MH "Musculoskeletal Abnormalities+") OR (MH "Shoulder Instability, Posterior") OR (MH "Shoulder Instability, Multidirectional") OR (MH "Shoulder Injuries+") OR (MH "Musculoskeletal Diseases+") OR (MH "Shoulder Instability, Anterior") OR (MH "Shoulder Impingement Syndrome") OR (MH "Scapula+") OR (MH "Rotator Cuff Injuries") OR (MH "Pectoralis Muscles") OR (MH "Neuralgia+") OR (MH "Headache+") OR (MH "Arthralgia+")
S7 (MH "Temporomandibular Joint Syndrome") OR (MH "Temporomandibular Joint") OR (MH "Temporomandibular Joint Diseases+") OR (MH "Jaw Abnormalities+") OR (MH "Trismus")
S8 (MH "Muscle Weakness+") OR (MH "Muscular Disorders, Atrophic+") OR (MH "Masticatory Muscles+") OR (MH "Muscular Atrophy+") OR (MH "Muscle Pain") OR (MH "Muscle Strength+") OR (MH "Muscle Hypotonia+") OR (MH "Muscle Fatigue") OR (MH "Neuromuscular Manifestations+") (MH "Posture+") OR (MH "Balance, Postural") OR (MH "Musculoskeletal Abnormalities+") OR (frozen-shoulder or joint-mobility or myofascial-pain or trismus or neuropath*) OR ((Pain* or ache* or discomfort* or injur* or sore* or tender* or agony or agonies or excruciat* or tear or tears or sprain* or strain* or dislocat* or cramp* or impingement* or instabilit* or stiff* or hypomobility or fibrosis or impair* or dysfunction*) N8 (jaw or facial or orofacial or face or neck or cervical or cervicothoracic or cervico-thoracic or cranial or mandibular or craniomandibular or temporomandibular or TMJ or axial or appendicular or musc* or MSK or postur* or spine or spinal or vertebral or forwardshoulder or thora* or Tx or shoulder* or rotator cuff or arm or arms or elbow* or lumbar or knee* or calf or calves or joint or joints or lower extremit* or lower limb* or upper extremit* or upper limb* or leg or legs or thigh*)) OR (muscle N3 (strength or activation* or fatigu*))
S10 S5 OR S6 OR S7 OR S8 OR S9
S11 (MH "Neck Dissection") OR (MH "Surgical Flaps+") OR (MH "Surgery, Reconstructive+") OR (MH "Perforator Flap") OR (MH "Robotic Surgical Procedures") OR (MH "Dissection")
S12 (MH "Radiosurgery") OR ((surger* N4 (neck or laryngeal)) or radiosurgery or neck dissection or neck reconstruction or surgical flap)
S13 S11 OR S12
S14 S4 AND S10 AND S13

Ovid MEDLINE(R) ALL 1946 to October2024
Number of results: 1287
Search saved as: HNC systematic review.
ovidweb(9).ovd

1. "head and neck neoplasms"/ or "squamous cell carcinoma of head and neck"/ or exp esophageal neoplasms/ or exp facial neoplasms/ or exp mouth neoplasms/ or exp otorhinolaryngologic neoplasms/
2. ((head or neck or esophageal or esophagus or facial or face or eyelid or mouth or gingival or lip or lips or palatal or palate* or salivary or tongue or oral or otorhinolaryngol* or throat or ear or nose or nasal or sinus or paranasal or pharyng* or oropharyng* or tonsil* or nasopharyng* or hypopharyng* or laryng*) adj5 (cancer* or neoplasm* or carcinoma* or tumor* or tumour* or malignan* or leukoplakia)).mp.
3. 1 or 2
4. jaw diseases/ or jaw abnormalities/ or joint diseases/ or arthralgia/ or shoulder pain/ or contracture/ or joint deformities, acquired/ or joint dislocations/ or shoulder dislocation/ or joint instability/ or joint loose bodies/ or shoulder impingement syndrome/ or temporomandibular joint disorders/ or exp craniomandibular disorders/ or musculoskeletal abnormalities/ or craniofacial abnormalities/
5. arm injuries/ or shoulder dislocation/ or leg injuries/ or neck injuries/ or abnormalities, radiation-induced/ or shoulder injuries/ or rotator cuff injuries/ or shoulder impingement syndrome/
6. neuromuscular manifestations/ or muscle cramp/ or muscle hypertonia/ or muscle rigidity/ or muscle hypotonia/ or muscle weakness/ or muscular atrophy/ or sarcopenia/ or spasm/ or hemifacial spasm/ or trismus/
7. musculoskeletal pain/ or myalgia/ or myofascial pain syndromes/
8. facial pain/ or neck pain/ or neuralgia/ or nociceptive pain/ or visceral pain/ or pain, postoperative/ or pain, referred/
9. (frozen shoulder or joint mobility or myofascial pain or trismus or neuropath*).mp.
10. ((Pain* or ache* or discomfort* or injur* or sore* or tender* or agony or agonies or excruciat* or tear or tears or sprain* or strain* or dislocat* or cramp* or impingement* or instabilit* or stiff* or hypomobility or fibrosis or impair* or dysfunction*) adj8 (jaw or facial or orofacial or face or neck or cervical or cervicothoracic or cervico-thoracic or cranial or mandibular or craniomandibular or temporomandibular or TMJ or axial or appendicular or musc* or MSK or postur* or spine or spinal or vertebral or forwardshoulder or thora* or Tx or shoulder* or rotator cuff or arm or arms or elbow* or lumbar or knee* or calf or calves or joint or joints or lower extremit* or lower limb* or upper extremit* or upper limb* or leg or legs or thigh*)).mp.
11. (muscle adj3 (strength or activation* or fatigu*)).mp.
12. 4 or 5 or 6 or 7 or 8 or 9 or 10 or 11
13. radiosurgery/
14. lymph node excision/ or neck dissection/ or sentinel lymph node biopsy/
15.
16. ((surger* adj4 (neck or laryngeal)) or radiosurgery or neck dissection or neck reconstruction or surgical flap).mp.
17. radiotherap*.mp.
18. 13 or 14 or 15 or 16
19. 3 and 12 and 18
20. limit 19 to animals
21. 19 not 20
22. case reports/ or (case-stud* or case-report*).jw. or (case-study or case-report).mp.
23. 21 not 22
24. metastas*.ti.
25. 23 not 24

Scopus (Scopus Interface)
From 1946 to October 2024
Number of results: 1,185
Search saved as: HNC systematic review.

((head OR neck OR mouth OR gingival OR palatal OR palate* OR tongue OR oral OR otorhinolaryn gol* OR nose OR nasal OR sinus OR paranasal OR pharyng* OR oropharyng* OR tonsil* OR nasopha ryng* OR hypopharyng* OR laryng*) W/5 (cancer* OR neoplasm* OR carcinoma* OR tumor* OR tu mour* OR malignan*))) AND (TITLE-ABS-
KEY ((surger* W/4 (neck OR laryngeal)) OR radiosurgery OR neck-dissection OR neck-
reconstruction OR surgical-flap OR sentinel-lymph-node-biopsy OR lymph-node-
excision OR surgical-flaps OR perforator-flap OR myocutaneous)) AND ((TITLE-ABS-
KEY (frozen AND shoulder OR joint AND mobility OR contracture OR myofascial OR neck OR fibro sis OR stiffness OR trismus OR neuropath*)) OR (TITLE-ABS-
KEY ((pain* OR ache* OR discomfort* OR injur* OR sore* OR tender* OR tear OR tears OR sprain* O R strain* OR dislocat* OR cramp* OR impingement* OR instabilit* OR stiff* OR hypomobility OR fibr osis OR impair* OR dysfunction*) (jaw OR facial OR orofacial OR face OR neck OR cervical OR cervi cothoracic OR cervico-
thoracic OR cranial OR mandibular OR craniomandibular OR temporomandibular OR tmj OR axial O R appendicular OR musc* OR msk OR postur* OR spine OR spinal OR vertebral OR forward AND sh oulder OR thora* OR tx OR shoulder* OR rotator-
cuff OR scapula OR arm OR arms AND joint OR joints OR lower-extremit* OR lower-
limb* OR upper-extremit* OR upper-limb* OR leg OR legs OR thigh*))) OR (#4 TITLE-ABS-
KEY (muscle W/3 (strength OR weakness OR hypotonia OR atrophy OR tightness OR activation* OR fatigu*))))

Embase (OVID interface)
From 1946 to October 2024
Number of results: 1,583
Search saved as: HNC systematic review.

1. "head and neck neoplasms"/ or "squamous cell carcinoma of head and neck"/ or exp esophageal neoplasms/ or exp facial neoplasms/ or exp mouth neoplasms/ or exp otorhinolaryngologic neoplasms/
2. ((head or neck or esophageal or esophagus or facial or face or eyelid or mouth or gingival or lip or lips or palatal or palate* or salivary or tongue or oral or otorhinolaryngol* or throat or ear or nose or nasal or sinus or paranasal or pharyng* or oropharyng* or tonsil* or nasopharyng* or hypopharyng* or laryng*) adj5 (cancer* or neoplasm* or carcinoma* or tumor* or tumour* or malignan* or leukoplakia)).mp.
3. 1 or 2
4. jaw diseases/ or jaw abnormalities/ or joint diseases/ or arthralgia/ or shoulder pain/ or contracture/ or joint deformities, acquired/ or joint dislocations/ or shoulder dislocation/ or joint instability/ or joint loose bodies/ or shoulder impingement syndrome/ or temporomandibular joint disorders/ or exp craniomandibular disorders/ or musculoskeletal abnormalities/ or craniofacial abnormalities/
5. arm injuries/ or shoulder dislocation/ or leg injuries/ or neck injuries/ or abnormalities, radiation-induced/ or shoulder injuries/ or rotator cuff injuries/ or shoulder impingement syndrome/
6. neuromuscular manifestations/ or muscle cramp/ or muscle hypertonia/ or muscle rigidity/ or muscle hypotonia/ or muscle weakness/ or muscular atrophy/ or sarcopenia/ or spasm/ or hemifacial spasm/ or trismus/
7. musculoskeletal pain/ or myalgia/ or myofascial pain syndromes/

8. facial pain/ or neck pain/ or neuralgia/ or nociceptive pain/ or visceral pain/ or pain, postoperative/ or pain, referred/
9. (frozen shoulder or joint mobility or myofascial pain or trismus or neuropath*).mp.
10. ((Pain* or ache* or discomfort* or injur* or sore* or tender* or agony or agonies or excruciat* or tear or tears or sprain* or strain* or dislocat* or cramp* or impingement* or instabilit* or stiff* or hypomobility or fibrosis or impair* or dysfunction*) adj8 (jaw or facial or orofacial or face or neck or cervical or cervicothoracic or cervico-thoracic or cranial or mandibular or craniomandibular or temporomandibular or TMJ or axial or appendicular or musc* or MSK or postur* or spine or spinal or vertebral or forwardshoulder or thora* or Tx or shoulder* or rotator cuff or arm or arms or elbow* or lumbar or knee* or calf or calves or joint or joints or lower extremit* or lower limb* or upper extremit* or upper limb* or leg or legs or thigh*)).mp.
11. (muscle adj3 (strength or activation* or fatigu*)).mp.
12. 4 or 5 or 6 or 7 or 8 or 9 or 10 or 11
13. radiosurgery/
14. lymph node excision/ or neck dissection/ or sentinel lymph node biopsy/
15.
16. ((surger* adj4 (neck or laryngeal)) or radiosurgery or neck dissection or neck reconstruction or surgical flap).mp.
17. radiotherap*.mp.
18. 13 or 14 or 15 or 16
19. 3 and 12 and 18
20. limit 19 to animals
21. 19 not 20
22. case reports/ or (case-stud* or case-report*).jw. or (case-study or case-report).mp.
23. 21 not 22
24. metastas*.ti.
25. 23 not 24
