# Supplementary material for: Musculoskeletal Impairments and Dysfunction in Individuals with Head and Neck Cancer Following Surgery with Neck Dissection—A Systematic Review
Source: Life (Basel). 2025 May 17;15(5):800. doi: 10.3390/life15050800 (PMC12112850; doi:10.3390/life15050800)
Supplement: Supplementary file 1 [file life-15-00800-s001.zip › Supplementary_Materials_B.pdf]

SUPPLEMENTARY MATERIALS B: List of Excluded studies

No	Title	Authors	Published Year	Journal
**Study protocol**				
1.	Intraoperative brief electrical stimulation (BES) for prevention of shoulder dysfunction after oncologic neck dissection: study protocol for a randomized controlled trial.	Barber, Brittany; McNeely, Margaret; Chan, K Ming; Beaudry, Rhys; Olson, Jaret; Harris, Jeffrey; Seikaly, Hadi; O'Connell, Daniel	2015	Trials
**Duplicate**				
2.	Risk of marginal mandibular nerve injury in neck dissection.	Moller, Martin Nue; Sorensen, Christian Hjort	2012	Journal of the European Federation of OtoRhino-Laryngological Societies (EUFOS)
3.	Neck and shoulder function in patients treated for oral malignancies: a 1-year prospective cohort study.	Speksnijder, Caroline M; van der Bilt, Andries; Slappendel, Margot; de Wijer, Anton; Merkx, Matthias A W; Koole, Ron	2013	Head & neck
4.	Shoulder Dysfunction after Selective Neck Dissection in Recurrent Nasopharyngeal Carcinoma.	Chan, Jimmy Yu Wai; Wong, Stanley Thian Sze; Chan, Richie Chiu Lung; Wei, William Ignace	2015	Journal of American Academy of Otolaryngology Head and Neck Surgery
5.	Quality of life after neck dissection	Nibu, K.-I.; Inoue, H.; Kawabata, K.; Ebihara, Y.; Onitsuka, T.; Fujii, T.; Saikawa, M.	2005	Toukeibu Gan
6.	Shoulder pain after neck dissection among head and neck cancer patients.	Wang, Hsiao-Lan	2009	Shoulder Pain After Neck Dissection Among Head & Neck Cancer Patients

7.	Does Pectoralis Major Myocutaneous Flap Cause the Shoulder Morbidity: A Clinical Comparative Study.	Anehosur, Venkatesh; Vadera, Hitesh; Bhat, Adithi; Satyanarayana, S; Kumar, Niranjan	2022	Indian journal of otolaryngology and head and neck surgery
**Surgical Procedure**				
8.	Preservation of the external jugular venous drainage system in neck dissection.	Chung, Man Ki; Choi, Jeesun; Lee, Jae-Kwon; Jeong, Jong In; Lee, Won Yong; Jeong, Han-Sin	2009	Journal of American Academy of Otolaryngology-Head and Neck Surgery
9.	Retrospective Outcome Analysis of Buccal Mucosal and Lower Alveolar Squamous Cell Carcinoma from a High-Volume Tertiary Cancer Centre.	Kapali, Aravind S; George, N A; Iype, E M; Thomas, S; Varghese, B T; Balagopal, P G; Sebastian, P	2019	Indian journal of surgical oncology
10.	Anterior belly of digastric muscle transfer: a useful technique in head and neck surgery.	Tan, Swee T	2002	Head & neck
11.	Utilization of intestinal clamps for precise cutting of the pectoralis major muscle while raising a myocutaneous flap.	Chaturvedi, P	2004	The Journal of laryngology and otology
12.	The transverse cervical vessels as recipient vessels for previously treated head and neck cancer patients.	Yu, Peirong	2005	Plastic and reconstructive surgery
13.	Clinical anatomy measurement of accessory nerve in neck dissection	Chen, Fei; Wang, Li-hong; Liang, Chuan-yu; Chen, Jian-chao; Li, Bin; Wang, Shao-xin	2006	Chinese journal of otorhinolaryngology head and neck surgery
14.	Laryngeal nerve morbidity in 1.273 central node dissections for thyroid cancer.	Liu, Xiaoli; Zhang, Daqi; Zhang, Guang; Zhao, Lina; Zhou, Le; Fu, Yantao; Li, Shijie; Zhao, Yishen; Li, Changlin; Wu,	2018	Surgical oncology

		Che-Wei; Chiang, Feng-Yu; Dionigi, Gianlorenzo; Sun, Hui		
15.	Comparison of flap outcomes between single- and multiple-perforator-based free anterolateral thigh flap in head and neck reconstruction.	Wang, Kuan-Chih; Tsai, Chia-Chin; Chang, Chih-Hao; Tseng, Wan-Ling; Hung, Kuo-Shu; Chang, Tzu-Yen; Chen, Szu-Han; Lee, Yao-Chou	2019	Microsurgery
16.	Surgical management of parapharyngeal space masses	Cohen S.M.; Burkey B.B.; Netterville J.L.	2005	Head and Neck
17.	Reconstruction of large defects of the neck using an extended vertical lower trapezius island myocutaneous flap following salvage surgery for neck recurrence of oral carcinoma	Chen W.-L.; Zhang B.; Wang J.-G.; Yang Z.-H.; Huang Z.-Q.; Zhang D.-M.	2011	Journal of Plastic, Reconstructive and Aesthetic Surgery
18.	The necessity of dissection of level IIb in laryngeal squamous cell carcinoma: A clinical study	Dundar R.; Aslan H.; Ozbay C.; Basoglu S.; Guvenc I.A.; Ogredik E.A.; Ozturkcan S.; Tayfun M.A.; Katilmis H.	2012	Otolaryngology - Head and Neck Surgery
19.	Use of extended vertical lower trapezius island myocutaneous flaps to cover exposed reconstructive plates	Fang S.-L.; Wang Y.-Y.; Chen W.-L.; Zhang D.-M.	2014	Journal of Oral and Maxillofacial Surgery
20.	Three-dimensional printing of patient-specific surgical plates in head and neck reconstruction: A prospective pilot study	Yang W.-F.; Choi W.S.; Leung Y.Y.; Curtin J.P.; Du R.; Zhang C.-Y.; Chen X.-S.; Su Y.-X.	2018	Oral Oncology
21.	Microvascular Free Flaps in Head and Neck Reconstruction	Reis, J.; Amarante, J.; Malheiro, E.; Santa-Comba, A.; Costa-Ferreira, A.; Luz Barroso, M.	1998	Acta Medica Portuguesa

22.	The forearm flap: assessment of functional and aesthetic outcomes and quality of life.	Ciuman R; Mohr C; KrÃ¶ger K; Dost P	2007	American Journal of Otolaryngology
23.	Pectoralis major myocutaneous flap-still a workhorse for maxillofacial reconstruction in developing countries.	Gadre, Kiran Shrikrishna; Gadre, Pushkar; Sane, Vikrant Dilip; Halli, Rajshekhar; Doshi, Pankaj; Modi, Sachin	2013	Journal of Oral & Maxillofacial Surgery
24.	Combined Anterolateral Thigh and Tensor Fasciae Latae Flaps: An Option for Reconstruction of Large Head and Neck Defects.	Tursun, Ramzey; Marwan, Hisham; IIIGreen, J. Marshall; Alotaibi, Fawaz; LeDoux, Andre; Green, J Marshall 3rd	2017	Journal of Oral & Maxillofacial Surgery
25.	Local Anesthetic Infusion for Improving Pain From Head and Neck Cancer Surgery.	Rahman, Siamak; Mendelsohn, Abie	2022	JAMA otolaryngology head & neck surgery
**Not related to the objective of the review**				
26.	Screening for dysfunction to promote multidisciplinary intervention by using the University of Washington Quality of Life Questionnaire.	Rogers, Simon N; Lowe, Derek	2009	Archives of otolaryngology--head & neck surgery
27.	Italian cross-cultural adaptation and validation of three different scales for the evaluation of shoulder pain and dysfunction after neck dissection: University of California - Los Angeles (UCLA) Shoulder Scale, Shoulder Pain and Disability Index (SPADI)	Marchese, C; Cristalli, G; Pichi, B; Manciocco, V; Mercante, G; Pellini, R; Marchesi, P; Sperduti, I; Ruscito, P; Spriano, G	2012	Acta otorhinolaryngologica Italica
28.	Evaluation of shoulder disability questionnaires used for the	Goldstein, David P; Ringash, Jolie; Bissada, Eric; Jaquet, Yves; Irish,	2014	Head & neck

	assessment of shoulder disability after neck dissection for head and neck cancer.	Jonathan; Chepeha, Douglas; Davis, Aileen M		
29.	Functional outcomes and quality of life after a 6-month early intervention program for oral cancer survivors: a single-arm clinical trial.	Chen, Yueh-Hsia; Liang, Wei-An; Hsu, Chung-Yin; Guo, Siang-Lan; Lien, Shwu-Huei; Tseng, Hsiao-Jung; Chao, Yuan-Hung	2018	PeerJ
30.	Psychometric properties of 3 patient-reported outcome measures for the assessment of shoulder disability after neck dissection.	Stuiver, Martijn M; ten Tusscher, Marieke R; van Opzeeland, Anita; Brendeke, Wim; Lindeboom, Robert; Dijkstra, Pieter U; Aaronson, Neil K	2016	Head & neck
31.	A new orthosis for trapezius palsy following radical neck dissection in patients with head and neck cancer	Akman M.N.; Sari Z.; Ersoy Y.; Saydam L.	1995	Fizik Tedavi Rehabilitasyon Dergisi
32.	Cost effectiveness of a free forearm flap in reconstruction of the oral cavity and pharynx - The donor site complications	Pabiszczak M.; Banaszewski J.; Balcerowiak A.; Szyfter W.	2012	Otolaryngologia Polska
33.	The efficacy of the Harmonic scalpel in neck dissection: A prospective randomized study	Shin Y.S.; Koh Y.W.; Kim S.-H.; Choi E.C.	2013	Laryngoscope
34.	Evaluation of retromolar space size for intubation feasibility	Truong A.; Truong D.-T.; Cata J.; Martin J.; Ahmad B.	2014	Canadian Journal of Anesthesia
35.	Selective neck dissection for neck residue of nasopharyngeal carcinoma: A prospective study	Chen J.Y.; Zhang L.; Ji Q.H.; Li D.S.; Shen Q.; Wang Z.Y.; Huang C.P.; Wang Y.; Zhu Y.X.	2015	Journal of Cranio-Maxillofacial Surgery
36.	Diagnostic and therapeutic features associated with modification of quality-of-life's outcomes between	Gobbo M.; Bullo F.; Perinetti G.; Gatto A.; Ottaviani G.; Biasotto M.; Tirelli G.	2016	Brazilian Journal of Otorhinolaryngology

	one and six months after major surgery for head and neck cancer			
37.	Postoperative pain management in head and cancer patients	Dort J.; Hinther A.V.; Chandarana S.; Matthews W.; Schrag C.; Nakoneshny S.	2017	Otolaryngology - Head and Neck Surgery (United States)
38.	Clinical and histopathological results of central compartment lymph nodes' dissection in papillary thyroid carcinoma	Diaz Y.C.; Bermudez C.R.; Sanmillan D.F.; Fernandez E.L.-T.; Hernandez J.R.H.	2013	European Surgical Research
39.	Perioperative pain management after fibular free flap harvesting for head-and-neck reconstruction using mini-catheters to inject local anesthetic: A pilot study	Ferri A.; Varazzani A.; Valente A.; Pedrazzi G.; Bianchi B.; Ferrari S.; Sesenna E.	2018	Microsurgery
40.	Accuracy of administrative and clinical registry data in reporting postoperative complications after surgery for oral cavity squamous cell carcinoma	Awad M.I.; Shuman A.G.; Montero P.H.; Palmer F.L.; Shah J.P.; Patel S.G.	2015	Head and Neck
41.	Utility of Superiorly Based Masseter Muscle Flap for Postablative Retromaxillary Reconstruction	Bande, C.R.; Mishra, A.; Gupta, M.K.; Goel, M.; Gawande, M.J.	2017	Journal of Oral and Maxillofacial Surgery
42.	The Role of IONM in Reducing the Occurrence of Shoulder Syndrome Following Lateral Neck Dissection for Thyroid Cancer.	Polistena, Andrea; Ranalli, Monia; Avenia, Stefano; Lucchini, Roberta; Sanguinetti, Alessandro; Galasse, Sergio; Rondelli, Fabio; Vannucci, Jacopo; Patrone, Renato; Velotti, Nunzio; Conzo, Giovanni; Avenia, Nicola	2021	Journal of clinical medicine
43.	The lateral cervical stria approach to selective neck dissection: a preliminary study.	Qian, Y; Tian, Z; Li, B; Xu, Y; Wang, Y; Du, Y; Bian, Y	2023	Medicina oral, patologia oral y cirugia bucal

44.	Assessment of quality of life after soft tissue resection of head and neck carcinoma and reconstruction with double-paddle peroneal artery perforator free flap.	Song, Peijun; Li, Jiancheng; Yang, Dongkun; Hu, Kai; Zhao, Tianlan	2023	The British journal of oral & maxillofacial surgery
**Not study design of interest**				
45.	Spinal accessory nerve preservation during neck dissection.	Eisele, D W; Weymuller, E A Jr; Price, J C	1991	The Laryngoscope
46.	Neck muscle atrophy and soft-tissue fibrosis after neck dissection and postoperative radiotherapy for oral cancer.	Kim, Jinu; Shin, Eun Seow; Kim, Jeong Eon; Yoon, Sang Pil; Kim, Young Suk	2015	Radiation oncology journal
47.	Head drop syndrome secondary to multimodality treatments for head and neck cancer	Smillie I.; Ellul D.; Townsley R.; James A.; Clark B.; Petty R.K.H.; Clark L.	2013	Laryngoscope
48.	A Study Comparing Free-Flap Reconstruction via the Retroauricular Approach and the Traditional Transcervical Approach for Head and Neck Cancer: A Matched Case-Control Study	Kim W.S.; Park J.H.; Byeon H.K.; Chang J.W.; Ban M.J.; Koh Y.W.; Choi E.C.	2015	Annals of Surgical Oncology
49.	Radical neck dissection. Preservation of function of the shoulder	Stell, P.M.; Jones, T.A.	1983	Journal of Laryngology and Otology
50.	Head and neck reconstruction using lateral thigh free flap: Flap design	Ha, B.; Baek, C.-H.	1999	Microsurgery
51.	Radical neck dissection after vascular reconstruction of the carotid artery	Ishiyama, T.; Sakaguchi, M.; Otsuka, A.; Miyashita, K.; Taguchi, K.; Katsuno, S.	1999	Practica Otologica, Supplement

52.	Leg compartment syndrome after fibula free flap.	Berzofsky C; Shin E; Mashkevich G	2013	Otolaryngology-Head & Neck Surgery
53.	Submental Osteocutaneous Perforator Flap for Maxillary and Mandibular Reconstruction Following Tumor Resection.	GarcÃa-de Marcos, Jose A.; Arroyo-RodrÃguez, Susana; Rey-Biel, Juan	2016	Journal of Oral & Maxillofacial Surgery (02782391)
54.	Pre-treatment quality of life as a predictor of distant metastasis-free survival and overall survival in patients with head and neck cancer who underwent free flap reconstruction.	Chen, M.N.; Ho, K.Y.; Hung, Y.N.; Su, C.C.; Kuan, C.H.; Tai, H.C.; Cheng, N.C.; Lin, C.C.	2019	European Journal of Oncology Nursing
55.	Use of the Teres Major Muscle in Chimeric Subscapular System Free Flaps for Head and Neck Reconstruction.	Tomlinson, Andrew R.; Jameson, Mark J.; Pagedar, Nitin A.; Schoeff, Stephen S.; Shearer, A. Eliot; Boyd, Nathan H.	2015	JAMA Otolaryngology-Head & Neck Surgery
56.	Neck stiffness after transoral pharyngolaryngeal surgery for squamous cell carcinoma	Bartier, S.; Mazzaschi, O.; Sauvaget, E.	2020	Annales Francaises d'Oto-Rhino-Laryngologie et de Pathologie Cervico-Faciale
**Not population group of interest**				
57.	The radial forearm flap: reconstructive applications and donor-site defects in 35 consecutive patients.	Swanson, E; Boyd, J B; Manktelow, R T	1990	Plastic and reconstructive surgery
58.	The effect of neck dissection on quality of life after chemoradiation.	Donatelli-Lassig, Amy Anne; Duffy, Sonia A; Fowler, Karen E; Ronis, David L; Chepeha, Douglas B; Terrell, Jeffrey E	2008	Otolaryngology--head and neck surgery : official journal of American Academy of Otolaryngology-Head and Neck Surgery

59.	Electrophysiologic activity of the vestibular fold.	Ricz, Hilton; Bastos, Patricia; Aguiar-Ricz, Lilian; Marques, Wilson Jr; Mamede, Rui Celso Martins	2010	Archives of otolaryngology--head & neck surgery
60.	Anterolateral thigh free flap for "head-to-toe" reconstruction.	Nasajpour, Hossein; Steele, Matthew H	2011	Annals of plastic surgery
61.	Free flap reconstruction of lateral mandibular defects: indications and outcomes.	Dean, Nichole R; Wax, Mark K; Virgin, Frank W; Magnuson, J Scott; Carroll, William R; Rosenthal, Eben L	2012	Otolaryngology--head and neck surgery : official journal of American Academy of Otolaryngology-Head and Neck Surgery
62.	Versatile use of submental tissue for reconstruction of perioral soft tissue defects.	Jeong, Seong-Ho; Lee, Byung-il	2012	The Journal of craniofacial surgery
63.	Free flaps in the reconstruction of head and neck. Clinical experience	Reis, J; Amarante, J; Malheiro, E; Santa-Comba, A; Costa-Ferreira, A; Barroso, M L	1998	Acta medica portuguesa
64.	Glomus jugulare tumor: tumor control and complications after stereotactic radiosurgery.	Foote, Robert L; Pollock, Bruce E; Gorman, Deborah A; Schomberg, Paula J; Stafford, Scott L; Link, Michael J; Kline, Robert W; Strome, Scott E; Kasperbauer, Jan L; Olsen, Kerry D	2002	Head & neck
65.	Immediate and long-term results of plastic reconstruction of soft tissues of the head and neck in areas previously treated with surgery for tumor and radiation injury]	Isaev, P A; Medvedev, V S; Pasov, V V; Semin, D Iu; Plichko, V I; Derbugov, D N	2005	Voprosy onkologii
66.	Reconstruction of Through and Through Oromandibular Defects With Combined Fibula Flap and Anterolateral Thigh Flap.	Gong, Zhao-Jian; Zhang, Shuai; Zhang, Sheng; Liu, Jiang; Xu, Yu-Ming; Wu, Han-Jiang	2017	Journal of oral and maxillofacial surgery

67.	Musculoskeletal neck disorders in thyroid cancer patients after thyroidectomy.	Rodriguez-Torres, Janet; Lopez-Lopez, Laura; Cabrera-Martos, Irene; Torres-Sanchez, Irene; Ortiz-Rubio, Araceli; Valenza, Marie C	2019	European journal of cancer care
68.	Functional neck dissection. III. Functional lateral cervical stripping	Staffieri A.	1976	Nuovo Archivio Italiano di Otologia Rinologia e Laringologia
69.	Definition of quality indicators in microsurgery in head and neck reconstruction based on a 5-year follow-up without a loss	Kessler P.; Poort L.; Bockmann R.; Lethaus B.	2013	Journal of Cranio-Maxillofacial Surgery
70.	Comparision of the outcome of suprafascial &subfascial dissection of radial forearm in head &neck reconstruction	Desai K.A.; Kumar N.; Singhania V.; Prabhu A.	2017	Head and Neck
71.	Thoracic duct injury during thyroid cancer operation	Gyory F.; Andrasi M.; Kovacs D.; Fedor R.	2017	Langenbeck's Archives of Surgery
72.	Total thyroidectomy for malignancy - is central neck dissection a risk factor for recurrent nerve injury and postoperative hypocalcemia? A tertiary center experience in romania	Giulea C.; Enciu O.; Toma E.A.; Martin S.; Fica S.; Miron A.	2019	Acta Endocrinologica
73.	Donor site morbidity of the fasciocutaneous radial forearm flap: What does the patient really bother?	De Witt, C.A.; De Bree, R.; Verdonck-De Leeuw, I.M.; Quak, J.J.; Leemans, C.R.	2007	European Archives of Oto-Rhino-Laryngology
74.	Degenerative and protective reactions of the rat trigeminal motor	Seki Y; Suzuki SO; Nakamura S; Iwaki T; Seki, Yoshihiro; Suzuki, Satoshi O; Nakamura, Seiji; Iwaki, Toru	2009	Journal of Oral Pathology & Medicine

	nucleus after removal of the masseter and temporal muscles.			
75.	Iliac crest internal oblique osteomusculocutaneous free flap reconstruction of the postablative palatomaxillary defect.	Genden EM; Wallace D; Buchbinder D; Okay D; Urken ML	2001	Archives of Otolaryngology - Head & Neck Surgery
76.	Endoscopic-assisted lateral neck dissection and open lateral neck dissection in the treatment of lateral neck lymph node metastasis in papillary thyroid carcinoma: A comparison of therapeutic effect.	Tao Ma; Shuai Zhang; Dongmei Huang; Gang Zhang; Boyi Chen; Ning Zhang	2022	Pakistan Journal of Medical Sciences
**Review (Not study design of interest)**				
77.	Care of defects in the facio cervical region with pedicle flaps and free transplants	Haas, e	1962	Archiv fur Ohren-, Nasen- und Kehlkopfheilkunde
78.	Radical Head And Neck Surgery In Irradiated Patients: Complications And Safeguards.	King, g d	1965	The Surgical clinics of North America
79.	Scar contracture after neck dissection. Causes, prevention, and treatment.	Futrell, J W; Chretien, P B	1976	American journal of surgery
80.	Reanimation of the long-standing partial facial paralysis.	Rubin, L R; Lee, G W; Simpson, R L	1986	Plastic and reconstructive surgery
81.	The spinal accessory nerve plexus, the trapezius muscle, and shoulder stabilization after radical neck cancer surgery.	Brown, H; Burns, S; Kaiser, C W	1988	Annals of surgery
82.	Free flaps in reconstructive surgery.	Soutar, D S	1989	Annals of the Royal College of Surgeons of England

83.	Elective supraomohyoid neck dissection for oral cavity squamous cell carcinoma: is dissection of sublevel IIB necessary?.	Elsheikh, Mohamed N; Rinaldo, Alessandra; Ferlito, Alfio; Fagan, Johannes J; Suarez, Carlos; Lowry, John; Paleri, Vinidh; Khafif, Avi; Olofsson, Jan	2008	Oral oncology
84.	Questionable necessity to remove the submandibular gland in neck dissection.	Takes, Robert P; Robbins, K Thomas; Woolgar, Julia A; Rinaldo, Alessandra; Silver, Carl E; Olofsson, Jan; Ferlito, Alfio	2011	Head & neck
85.	Exercise interventions for shoulder dysfunction in patients treated for head and neck cancer.	Carvalho, Alan P V; Vital, Flavia Mr; Soares, Bernardo G O	2012	The Cochrane database of systematic reviews
86.	Level V Clearance in Neck Dissection for Papillary Thyroid Carcinoma: A Need for Homogeneous Studies.	Battoo, Azhar Jan; Sheikh, Zahoor Ahmad; Thankappan, Krishnakumar; Mir, Abdul Wahid; Haji, Altaf Gowhar	2018	International archives of otorhinolaryngology
87.	Laser utilization in the oral pharynx.	Rathfoot, C J; Coleman, J A	1996	Otolaryngologic clinics of North America
88.	Disfigurement and dysfunction with head and neck cancer surgery.	Dropkin, M J	1998	ORL-head and neck nursing : official journal of the Society of Otorhinolaryngology and Head-Neck Nurses
89.	Avoiding pitfalls in surgery of the neck, parapharyngeal space, and infratemporal fossa.	Sharma, Pramod K; Massey, Becky L	2005	Otolaryngologic clinics of North America
90.	Trismus Secondary Release Surgery and Microsurgical Free Flap Reconstruction After Surgical Treatment of Head and Neck Cancer.	Chang, Yang-Ming; Deek, Nidal Farhan Al; Wei, Fu-Chan	2016	Clinics in plastic surgery
91.	Physical rehabilitation after myocutaneuous flaps	Har-El G.; Krespi Y.P.; Har-El R.	1990	Head and Neck

92.	Preliminary multi-institutional prospective pathologic and molecular studies support preservation of sublevel IIB and level IV for laryngeal squamous carcinoma with clinically negative neck	Ferlito A.; Silver C.E.; Suarez C.; Rinaldo A.	2007	European Archives of Oto-Rhino-Laryngology
93.	Otolaryngological cancer pain at the after-effects stage	Navez M.	2009	Douleurs
94.	Upper quadrant impairments associated with cancer treatment	Stout N.L.; Levy E.; Pfalzer L.	2011	Topics in Geriatric Rehabilitation
95.	Pectoralis major myocutaneous flap	Carlson E.R.	2003	Oral and Maxillofacial Surgery Clinics of North America
96.	Complications of neck dissection	Genden E.M.; Ferlito A.; Shaha A.R.; Talmi Y.P.; Robbins K.T.; Rhys-Evans P.H.; Rinaldo A.	2003	Acta Oto-Laryngologica
97.	Evolution of neck dissection for improved functional outcome	Samant S.; Robbins K.T.	2003	World Journal of Surgery
98.	Lateral neck dissection	Khafif A.	2004	Operative Techniques in Otolaryngology - Head and Neck Surgery
99.	Quality of life in patients with cancer of the oral cavity and oropharynx	Cernea C.R.; Morais-Besteiro J.	2004	Operative Techniques in Otolaryngology - Head and Neck Surgery
100.	Quality of life following neck dissections	Rogers S.N.; Ferlito A.; Pellitteri P.K.; Shaha A.R.; Rinaldo A.	2004	Acta Oto-Laryngologica
101.	Robot-assisted neck dissection through a modified facelift or retroauricular approach	Koh Y.W.; Duvvuri U.; Choi E.C.	2013	Operative Techniques in Otolaryngology - Head and Neck Surgery

102.	Physical functioning and rehabilitation for the cancer survivor	Stubblefield M.D.; Schmitz K.H.; Ness K.K.	2013	Seminars in Oncology
103.	Robotic surgery in ear nose and throat	Parmar A.; Grant D.G.; Loizou P.	2010	European Archives of Oto-Rhino-Laryngology
104.	Surgery Versus Radiotherapy for Early Oropharyngeal Tumors: a Never-Ending Debate	Monnier Y.; Simon C.	2015	Current Treatment Options in Oncology
105.	Prophylactic central compartment neck dissection for papillary thyroid cancer: The search for justification continues	McHenry C.R.	2011	Surgery
106.	Shoulder Pain and Dysfunction after Head and Neck Cancer and Thyroid Cancer Treatment	Spinelli B.A.	2017	Rehabilitation Oncology
107.	Radical neck dissection	McCammon, S.D.; Shah, J.P.	2004	Operative Techniques in Otolaryngology - Head and Neck Surgery
108.	Cancer of the superior aerodigestive tract: postsurgical biomechanics of the cervicobrachial complex. Physiotherapeutical treatment of the sequelae.	Pardo Carballido C	2006	Fisioterapia
109.	Minimally invasive esophagectomy for esophageal squamous cell carcinoma--Shanghai Chest Hospital experience.	Bin Li; Yu Yang; Yifeng Sun; Rong Hua; Xiaobin Zhang; Xufeng Guo; Haiyong Gu; Bo Ye; Zhigang Li; Teng Mao	2018	Journal of Thoracic Disease
110.	Neck dissection for head and neck cancer functional considerations -- the cervical spine.	Gudas S	2001	Rehabilitation Oncology

111.	Delayed anterior ischemic optic neuropathy after neck dissection.	GÃtte K; Riedel F; Knorz MC; HÃrmann K	2000	Archives of Otolaryngology - Head & Neck Surgery
**Not outcome of interest**				
112.	Radical or modified neck dissection: a therapeutic dilemma.	Jesse, R H; Ballantyne, A J; Larson, D	1978	American journal of surgery
113.	Functional radical neck dissection.	Ariyan, S	1980	Plastic and reconstructive surgery
114.	The platysma musculocutaneous flap: experience with 24 cases.	Coleman, J J 3rd; Jurkiewicz, M J; Nahai, F; Mathes, S J	1983	Plastic and reconstructive surgery
115.	Scaling of disfigurement and dysfunction in postoperative head and neck patients.	Dropkin, M J; Malgady, R G; Scott, D W; Oberst, M T; Strong, E W	1983	Head & neck surgery
116.	[Scar neuroma following tumor operations of the head and neck. Immunohistologic studies, differential diagnosis, therapy].	Rauchfuss, A; Caselitz, J	1987	HNO
117.	Coping with disfigurement and dysfunction after head and neck cancer surgery: a conceptual framework.	Dropkin, M J	1989	Seminars in oncology nursing
118.	Adaptation to surgery for head and neck cancer.	Krouse, J H; Krouse, H J; Fabian, R L	1989	The Laryngoscope
119.	Reconstruction of defects in the head and neck with free flaps: 20 years experience.	Eckardt, A; Meyer, A; Laas, U; Hausamen, J-E	2007	The British journal of oral & maxillofacial surgery
120.	[Head and neck reconstruction with antebrachial flap concerning 100 patients].	Ricard, A-S; Zwetyenga, N; Laurentjoye, M; Siberchicot, F; Majoufre-Lefebvre, C	2008	Annales de chirurgie plastique et esthetique

121.	IIb or not IIb: oncologic role of submuscular recess inclusion in selective neck dissections.	Hoyt, Benjamin J A; Smith, Rachel; Smith, Anita; Trites, Jonathan; Taylor, S Mark	2008	Journal of otolaryngology
122.	Marginal mandibular nerve injury during neck dissection and its impact on patient perception of appearance.	Batstone, Martin D; Scott, Barry; Lowe, Derek; Rogers, Simon N	2009	Head & neck
123.	Postoperative myocardial injury after major head and neck cancer surgery.	Nagele, Peter; Rao, Lesley K; Penta, Mrudula; Kallogjeri, Dorina; Spitznagel, Edward L; Cavallone, Laura F; Nussenbaum, Brian; Piccirillo, Jay F	2011	Head & neck
124.	Neck dissection complications	Dedivitis, Rogerio Aparecido; Guimaraes, Andre Vicente; Pfuetzenreiter, Elio Gilberto Jr; Castro, Mario Augusto Ferrari de	2011	Brazilian journal of otorhinolaryngology
125.	Healing time of radial forearm free flap donor sites after preoperative tissue expansion: randomized controlled trial.	Bonaparte, James P; Corsten, Martin J; Allen, Murray	2011	Journal of otolaryngology - head & neck surgery
126.	Free vascularized flaps for reconstruction of the mandible: complications, success, and dental rehabilitation.	van Gemert, Johannes T M; van Es, Robert J J; Rosenberg, Antoine J W P; van der Bilt, Andries; Koole, Ron; Van Cann, Ellen M	2012	Journal of oral and maxillofacial surgery
127.	Abdominal compression: a new intraoperative maneuver to detect chyle fistulas during left neck dissections that include level IV.	Cernea, Claudio R; Hojaij, Flavio C; De Carlucci, Dorival Jr; Tavares, Marcos R; Araujo-Filho, Vergilius J; Silva-Filho, Gilberto Britto E; Brandao, Lenine G	2012	Head & neck
128.	Complications of head and neck skin expansion	Belghith, A; Jeblaoui, Y; Njah, H; Zairi, I; Zitouni, K; Adouani, A	2012	Revue de stomatologie et de chirurgie maxillo-faciale

129.	Functional implications of radical neck dissection and the impact on the quality of life for patients with head and neck neoplasia.	Popescu, B; Bertesteanu, S V G; Grigore, R; Scaunasu, R; Popescu, C R	2012	Journal of medicine and life
130.	Scapular free flap harvest site: recognising the spectrum of radiographic post-operative appearance.	Powell, D K; Nwoke, F; Urken, M L; Buchbinder, D; Jacobson, A S; Silberzweig, J E; Khorsandi, A S	2013	The British journal of radiology
131.	Harmonic scalpel versus conventional haemostasis in neck dissection: a prospective randomized study.	Ferri, Emanuele; Armato, Enrico; Spinato, Giacomo; Lunghi, Marcello; Tirelli, Giancarlo; Spinato, Roberto	2013	International journal of surgical oncology
132.	Elective neck dissection for primary oral cavity squamous cell carcinoma involving the tongue should include sublevel IIb.	Maher, Nigel Gordon; Hoffman, Gary Russell	2014	Journal of oral and maxillofacial surgery
133.	Pain on the first postoperative day after head and neck cancer surgery.	Inhestern, Johanna; Schuerer, Jenny; Illge, Christina; Thanos, Ira; Meissner, Winfried; Volk, Gerd Fabian; Guntinas-Lichius, Orlando	2015	European archives of otorhinolaryngology
134.	Modified pectoralis major myocutaneous flap in reconstruction of head and neck defects.	Chen, Jie; Huang, Wenxiao; Li, Zan; Zhou, Xiao; Yu, Jianjun; Bao, Ronghua; Zhang, Hailin; Ling, Hang	2015	Chinese journal of otorhinolaryngology head and neck surgery
135.	Institutional experience with lateral neck dissections for thyroid cancer.	Glenn, Jason A; Yen, Tina W F; Fareau, Gilbert G; Carr, Azadeh A; Evans, Douglas B; Wang, Tracy S	2015	Surgery
136.	Protection and Dissection of Recurrent Laryngeal Nerve in Salvage Thyroid Cancer Surgery to	Yu, Wen-Bin; Zhang, Nai-Song	2015	Asian Pacific journal of cancer prevention : APJCP

	Patients with Insufficient Primary Operation Extent and Suspicious Residual Tumor.			
137.	Coping with disfigurement/dysfunction and length of hospital stay after head and neck cancer surgery.	Dropkin, M J	1997	ORL-head and neck nursing Nurses
138.	Long-term regional control after radiation therapy and neck dissection for base of tongue carcinoma.	Lee, H J; Zelefsky, M J; Kraus, D H; Pfister, D G; Strong, E W; Raben, A; Shah, J P; Harrison, L B	1997	International journal of radiation oncology, biology, physics
139.	Assessment of postoperative pain after laryngeal surgery for cancer.	Mom, T; Bazin, J E; Commun, F; Dubray, C; Eschalier, A; Derbal, C; Avan, P; Gilain, L	1998	Archives of otolaryngology--head & neck surgery
140.	Body image and quality of life after head and neck cancer surgery.	Dropkin, M J	1999	Cancer practice
141.	Technical modifications of the latissimus dorsi pedicled flap to increase versatility and viability.	Hayden, R E; Kirby, S D; Deschler, D G	2000	The Laryngoscope
142.	The role of postradiotherapy neck dissection in supraglottic carcinoma.	Chan, A W; Ancukiewicz, M; Carballo, N; Montgomery, W; Wang, C C	2001	International journal of radiation oncology, biology, physics
143.	Omental free flap reconstruction in complex head and neck deformities.	Losken, Albert; Carlson, Grant W; Culbertson, John H; Scott Hultman, C; Kumar, Ajay V; Jones, Glyn E; Bostwick, John 3rd; Jurkiewicz, Maurice J	2002	Head & neck
144.	Perioperative complications, comorbidities, and survival in oral or oropharyngeal cancer.	de Cassia Braga Ribeiro, Karina; Kowalski, Luiz Paulo; Latorre, Maria do Rosario Dias de Oliveira	2003	Archives of otolaryngology--head & neck surgery

145.	Extended vertical trapezius myocutaneous flap in head and neck reconstruction as a salvage procedure.	Ugurlu, Kemal; Ozcelik, Derya; Huthut, Ilkay; Yildiz, Kemalettin; Kilinc, Leyla; Bas, Lutfu	2004	Plastic and reconstructive surgery
146.	Selective neck dissection for clinically N0 neck in laryngeal cancer: is dissection of level IIb necessary?.	Coskun, H Hakan; Erisen, Levent; Basut, Oguz	2004	Otolaryngology head and neck surgery :
147.	Repair and reconstruction of oral and maxillofacial defect--clinical analysis of 1973 cases	Zhang, Chenping; Zhang, Zhiyuan; Qiu, Weiliu; Lin, Guochu; Lan, Hanguang; Tang, Yousheng; Sun, Jian; Shen, Guofang; Hu, Yongjie; Ye, Weimin; Li, Jun; Ji, Tong; Xu, Liqun	2005	Chinese journal of reparative and reconstructive surgery
148.	Neurological complications following functional neck dissection.	Prim, M P; De Diego, J I; Verdaguer, J M; Sastre, N; Rabanal, I	2006	European archives of otorhino-laryngology
149.	Free tensor fasciae latae musculofasciocutaneous flap in reconstructive surgery: a series of 85 cases.	Bulstrode, N W; Kotronakis, I; Baldwin, M A R	2006	Journal of plastic, reconstructive & aesthetic surgery : JPRAS
150.	Level V lymph node dissection in oral and oropharyngeal carcinoma patients with clinically node-positive neck: is it absolutely necessary?.	Lim, Young Chang; Koo, Bon Seok; Lee, Jin Seok; Choi, Eun Chang	2006	The Laryngoscope
151.	The tunneled supraclavicular island flap-a fasciocutaneous flap for head and neck reconstructions.	Jeremic, J; Nikolic, Z; Colic, M; Jeremic, K	2006	Acta chirurgica Iugoslavica

152.	Level IIB Neck Dissection in Oral Squamous Cell Carcinoma: Science or Myth?.	Ghantous, Yasmine; Akrish, Sharon; Abd-Elraziq, Morad; El-Naaj, Imad Abu	2016	The Journal of craniofacial surgery
153.	Endoscopic-assisted selective neck dissection via small lateral neck incision for early-stage (T1-2N0M0) head and neck squamous cell carcinoma: 3-year follow-up results.	Liang, Faya; Fan, Song; Han, Ping; Cai, Qian; Lin, Peiliang; Chen, Renhui; Yu, Shitong; Huang, Xiaoming	2017	Surgical endoscopy
154.	Complex Orofacial Reconstruction with the Intrinsic Chimeric Flap.	Maldonado, Andres A; Silva, Amanda K; Humphries, Laura S; Gottlieb, Lawrence J	2017	Journal of reconstructive microsurgery
155.	The utility of the musculocutaneous anterolateral thigh flap in pharyngolaryngeal reconstruction in the high-risk patient.	Ooi, Adrian S H; Teven, Chad M; Inbal, Amir; Chang, David W	2017	Journal of surgical oncology
156.	Lymphangiomatous Tumors of the Neck: Tips to Avoid Iatrogenic Nerve Injuries in the Brachial Plexus Region.	Lin, Jerry Tsung-Kai; Lu, Johnny Chuieng-Yi; Huang, Yenlin; Chang, Tommy Nai-Jen; Chuang, David Chwei-Chin	2018	Annals of plastic surgery
157.	Transoral Robotic Surgery With Transoral Retropharyngeal Lymph Node Dissection in Patients With Tonsillar Cancer: Anatomical Points, Surgical Techniques, and Clinical Usefulness.	Park, Young Min; Cha, Dongchul; Koh, Yoon Woo; Choi, Eun Chang; Kim, Se-Heon	2019	The Journal of craniofacial surgery
158.	Longitudinal Perioperative Pain Assessment in Head and Neck Cancer Surgery.	Buchakjian, Marisa R; Davis, Andrew B; Sciegienka, Sebastian J; Pagedar, Nitin A; Sperry, Steven M	2017	The Annals of otology, rhinology, and laryngology
159.	Impact of skeletal muscle mass volume on surgical site infection in	Makiguchi, Takaya; Yamaguchi, Takahiro; Nakamura, Hideharu; Suzuki, Keisuke;	2019	Microsurgery

	free flap reconstruction for oral cancer.	Harimoto, Norifumi; Shirabe, Ken; Yokoo, Satoshi		
160.	The necessity of level IIb dissection for clinically negative neck oral squamous cell carcinoma.	Garreau, B; Dubreuil, P-A; Bondaz, M; Majoufre, C; Etchebarne, M	2020	Journal of stomatology, oral and maxillofacial surgery
161.	The latissimus dorsi donor site. Current use in head and neck reconstruction	Haughey B.H.; Fredrickson J.M.	1991	Archives of otolaryngology head & neck surgery
162.	Restoration of anterior mandible with the free fibula osseocutaneous flap	Cheung S.W.; Anthony J.P.; Singer M.I.	1994	Laryngoscope
163.	Osteomyocutaneous flap reconstruction for major mandibular defects	Savant D.N.; Kavarana N.M.; Bhathena H.M.; Salkar S.; Ghosh S.	1994	Journal of Surgical Oncology
164.	The radial forearm flap: A reconstructive chameleon	Niazi Z.B.M.; McLean N.R.; Black M.J.M.	1994	Journal of Reconstructive Microsurgery
165.	The ultimate modification in the modified neck dissection	Porter G.A.; Temple W.J.; Huchcroft S.	1995	American Journal of Surgery
166.	Accessory nerve damages and impaired shoulder movements after neck dissections	Miyata K.; Kitamura H.	1997	American Journal of Otolaryngology - Head and Neck Medicine and Surgery
167.	Pectoralis major miocutaneous flap in reconstruction of ablative cervicofacial defects: Our experience	Mevio E.; Sbrocca M.; Gorini E.; Artesi L.; Mullace M.; Mevio N.	2006	Otorinolaringologia
168.	Clinical observations of the anatomy and function of the marginal mandibular nerve	Nason R.W.; Binahmed A.; Torchia M.G.; Thliversis J.	2007	International Journal of Oral and Maxillofacial Surgery
169.	A combined anterolateral thigh flap and vascularized iliac crest flap in	Gaggl A.; Burger H.; Muller E.; Chiari F.M.	2007	International Journal of Oral and Maxillofacial Surgery

	the reconstruction of extended composite defects of the anterior mandible			
170.	The trapezius osteomyocutaneous island flap for reconstructing hemimandibular and oral defects following the ablation of advanced oral malignant tumours	Chen W.-l.; Chen Z.-w.; Yang Z.-h.; Huang Z.-q.; Li J.-s.; Zhang B.; Wang J.-g.	2009	Journal of Cranio-Maxillofacial Surgery
171.	Transoral laser microsurgery (TLM) +/- adjuvant therapy for advanced stage oropharyngeal Cancer: Outcomes and prognostic factors	Rich J.T.; Milov S.; Lewis Jr. J.S.; Thorstad W.L.; Adkins D.R.; Haughey B.H.	2009	Laryngoscope
172.	A comparison of free transfer of radial forearm and anterolateral thigh flaps for head and neck reconstruction	Tamimy M.S.; Rashid M.; Islam M.Z.; Sarwar S.-U.-R.; Aman S.; Aslam A.	2009	European Journal of Plastic Surgery
173.	Timing of radiotherapy in head and neck free flap reconstruction - a study of postoperative complications	Halle M.; Bodin I.; Tornvall P.; Wickman M.; Farnebo F.; Arnander C.	2009	Journal of Plastic, Reconstructive and Aesthetic Surgery
174.	Dysphagia in head and neck cancer	Manikantan K.; Khode S.; Sayed S.I.; Roe J.; Nutting C.M.; Rhys-Evans P.; Harrington K.J.; Kazi R.	2009	Cancer Treatment Reviews
175.	Reconstruction by lateral cervical flap of perioral and oral cavity: Clinical and experimental studies	Kummoona R.	2010	Journal of Craniofacial Surgery
176.	Transaxillary-subclavian transfer of pedicled latissimus dorsi musculocutaneous flap to head and neck region	Demirtas Y.; Yagmur C.; Kelahmetoglu O.; Demir A.; Guneren E.	2010	Journal of Craniofacial Surgery

177.	The utility of the multi-island vertical rectus abdominis myocutaneous (VRAM) flap in head and neck reconstruction	Matros E.; Patel N.P.; Cordeiro P.G.	2011	Oral Oncology
178.	Outcome of neck dissections in a rural tertiary University Hospital in Malaysia	Ramli R.R.; Hassan S.; Adil A.R.S.; Abdullah B.	2011	Pakistan Journal of Medical Sciences
179.	Healing time of radial forearm free flap donor sites after preoperative tissue expansion: Randomized controlled trial	Bonaparte J.P.; Corsten M.J.; Allen M.	2011	Journal of Otolaryngology - Head and Neck Surgery
180.	Anterolateral thigh cutaneous flap vs radial forearm free flap: Our experience in oral, oropharyngeal and hypopharyngeal reconstruction	Camaionia A.; Loreti A.; Damiani V.; Simone M.; Sinopoli I.	2012	European Archives of Oto-Rhino-Laryngology
181.	Microvascular reconstruction of the mouth, jaws, and face: Experience of an australian oral and maxillofacial surgery unit	Hoffman G.R.; Islam S.; Eisenberg R.L.	2012	Journal of Oral and Maxillofacial Surgery
182.	Postoperative and Postradiation Changes on Imaging	Lobert P.; Srinivasan A.; Shah G.V.; Mukherji S.K.	2012	Otolaryngologic Clinics of North America
183.	Free-flap reconstruction of large full-thickness lip and chin defects	Godefroy W.P.; Klop W.M.C.; Smeele L.E.; Lohuis P.J.F.M.	2012	Annals of Otology, Rhinology and Laryngology
184.	Retrospective analysis of pectoralis major myocutaneous flap surgeries performed under war conditions	Brkic F.; Dedic S.D.	2000	Croatian Medical Journal
185.	Anterior belly of digastric muscle transfer: A useful technique in head and neck surgery	Tan S.T.	2002	Head and Neck

186.	Analytic review of 2372 free flap transfers for head and neck reconstruction following cancer resection	Nakatsuka T.; Harii K.; Asato H.; Takushima A.; Ebihara S.; Kimata Y.; Yamada A.; Ueda K.; Ichioka S.	2003	Journal of Reconstructive Microsurgery
187.	Traumatic neuroma and recurrent lymphadenopathy after neck dissection: Comparison of radiologic features	Yabuuchi H.; Kuroiwa T.; Fukuya T.; Tomita K.; Hachitanda Y.	2004	Radiology
188.	Treatment results of post-operative radiotherapy in patients with salivary duct carcinoma of the major salivary glands	Kim J.Y.; Lee S.-W.; Cho K.-J.; Kim S.Y.; Nam S.Y.; Choi S.-H.; Roh J.-L.; Choi E.K.; Kim J.H.; Song S.Y.; Shin H.S.; Chang S.-K.; Ahn S.D.	2012	British Journal of Radiology
189.	The "Reconstruction of head and neck defects with the submental island flap	Xuwei D.; Jian X.; Xueqin L.; Xianjie Z.; Jianbo Y.; Wei L.; Ligen M.	2013	Head and Neck Oncology
190.	Mohs Micrographic surgery for head and neck lesions: A six years single centre experience	Jayaram R.; Craggs L.; Ah-Weng A.; Manisali M.	2014	British Journal of Oral and Maxillofacial Surgery
191.	The free scapular/parascapular flap as a reliable method of reconstruction in the head and neck region: A retrospective analysis of 130 reconstructions performed over a period of 5 years in a single Department	Mitsimponas K.T.; Iliopoulos C.; Stockmann P.; Bumiller L.; Nkenke E.; Neukam F.W.; Schlegel K.-A.	2014	Journal of Cranio-Maxillofacial Surgery
192.	Indications and outcomes for 100 patients managed with a pectoralis major flap within a UK maxillofacial unit	Avery C.M.E.; Gandhi N.; Peel D.; Neal C.P.	2014	International Journal of Oral and Maxillofacial Surgery

193.	Surgical management of head and neck cancers, with consideration of cosmetic and functional problems	Hatano A.	2014	Tokyo Jikeikai Medical Journal
194.	Primary surgical therapy for locally limited oral tongue cancer	Mantsopoulos K.; Psychogios G.; Kunzel J.; Waldfahrer F.; Zenk J.; Iro H.	2014	BioMed Research International
195.	Pressure injury can occur in patients undergoing prolonged head and neck surgery	Wright K.M.; Van Netten Y.; Dorrington C.A.; Hoffman G.R.	2014	Journal of Oral and Maxillofacial Surgery
196.	Outcomes after resection of post chemotherapy residual neck mass in patients with germ cell tumor - An update	Gupta A.; Feifer A.; Gotto G.; Kraus D.; Carver B.; Sheinfeld J.	2010	Journal of Urology
197.	The scapular free flap: When versatility is needed in head and neck reconstruction	Moukarbel R.V.; White J.B.; Fung K.; Franklin J.H.; Yoo J.H.	2010	Journal of Otolaryngology - Head and Neck Surgery
198.	Microsurgical free flap in head and neck reconstruction	Wong C.-H.; Wei F.-C.	2010	Head and Neck
199.	Pectoralis major myocutaneous flaps for head and neck reconstruction: Factors influencing occurrences of complications and the final outcome	Pinto F.R.; Malena C.R.; Vanni C.M.R.S.; de Aquino Capelli F.; de Matos L.L.; Kanda J.L.	2010	Sao Paulo Medical Journal
200.	Treatment outcomes of T4 locally advanced head and neck cancers with soft tissue invasion or bone and cartilage invasion	Do L.; Puthawala A.; Syed N.; Azawi S.; Williams R.; Vora N.	2009	American Journal of Clinical Oncology: Cancer Clinical Trials
201.	Neck incision planning for total laryngectomy: A finite element analysis	Feng A.L.; Clark J.H.; Agrawal N.; Moussa W.; Richmon J.D.	2015	Journal of Biomechanics

202.	Therapeutic robot-assisted neck dissection via a retroauricular or modified facelift approach in head and neck cancer: A comparative study with conventional transcervical neck dissection	Kim W.S.; Byeon H.K.; Park Y.M.; Ha J.G.; Kim E.S.; Koh Y.W.; Choi E.C.	2015	Head and Neck
203.	Level IIB neck dissection in oral squamous cell carcinoma: Science or myth	Ghantousa Y.; Akrish S.; Abdelraziq M.; El-Naaj I.A.	2017	Head and Neck
204.	Nasal ala pressure sores following head and neck reconstructive surgery: A retrospective analysis from a tertiary cancer hospital	Rastogi S.; Bhutia T.; Singh A.; Arun P.	2017	Indian Journal of Anaesthesia
205.	Remote peripheral tissue oxygenation does not predict postoperative free flap complications in complex head and neck cancer surgery: A prospective cohort study	Guye M.-L.; Motamed C.; Chemam S.; Leymarie N.; Suria S.; Weil G.	2017	Anaesthesia Critical Care and Pain Medicine
206.	Primary transoral robotic surgery with concurrent neck dissection for early stage oropharyngeal squamous cell carcinoma implemented at a Danish head and neck cancer center: a phase II trial on feasibility and tumour margin status	Rubek N.; Channir H.I.; Charabi B.W.; Lajer C.B.; Kiss K.; Nielsen H.U.; Bentzen J.; Friborg J.; von Buchwald C.	2017	European Archives of Oto-Rhino-Laryngology
207.	Quality of life in survivors of squamous cell carcinoma of oral	Naqvi S.U.; Zia S.; Farrukh M.S.; Begum K.; Shaikh S.M.; Maqbool S.A.; Aslam R.	2017	Rawal Medical Journal

	and oropharyngeal patients in Karachi, Pakistan			
208.	Neck dissection for head and neck malignancies: A Malaysian 13 years review	Nik Hishamuddin N.H.A.; Azman M.; Kong M.H.; Baki M.M.; Athar P.P.S.H.; Yunus M.R.M.	2017	Bangladesh Journal of Medical Science
209.	Predictors of adverse events after neck dissection: An analysis of the 2006-2011 National Surgical Quality Improvement Program (NSQIP) database	Jain U.; Somerville J.; Saha S.; Ver Halen J.P.; Antony A.K.; Samant S.; Kim J.Y.	2017	Ear, Nose and Throat Journal
210.	Predicting complications of major head and neck oncological surgery: An evaluation of the ACS NSQIP surgical risk calculator	Vosler P.S.; Orsini M.; Enepekides D.J.; Higgins K.M.	2018	Journal of Otolaryngology - Head and Neck Surgery
211.	Marginal mandibular nerve injury during neck dissection level IIa, and influence of different types of dissection: Diathermy versus cold knife	Estomba C.M.C.; Suarez J.A.S.; Garcia J.A.G.; Sarasola E.L.; Arrizabalaga I.T.; Mariezcurrena X.A.	2018	Otolaryngologia Polska
212.	Prognostic indication of sarcopenia for wound complication after total laryngectomy	Achim V.; Bash J.; Mowery A.; Guimaraes A.R.; Li R.; Schindler J.; Wax M.; Andersen P.; Clayburgh D.	2017	JAMA Otolaryngology - Head and Neck Surgery
213.	Staged inset of free flaps for complex microsurgical head and neck reconstruction to ensure total flap survival	Huang T.C.T.; Ciudad P.; Manrique O.J.; Agko M.; Chen S.-H.; Tang P.Y.-B.; Sabbagh M.D.; Chen H.-C.	2018	Microsurgery
214.	Secondary free tissue transfer in head and neck reconstruction	Vamadeva S.V.; Henry F.P.; Mace A.; Clarke P.M.; Wood S.H.; Jallali N.	2019	Journal of Plastic, Reconstructive and Aesthetic Surgery
215.	Association between Sarcopenia and Mortality in Patients	Stone L.; Olson B.; Mowery A.; Krasnow S.; Jiang A.; Li R.; Schindler J.; Wax M.K.;	2019	JAMA Otolaryngology - Head and Neck Surgery

	Undergoing Surgical Excision of Head and Neck Cancer	Andersen P.; Marks D.; Achim V.; Clayburgh D.		
216.	Oncologic outcomes of extended neck dissections in human papillomavirus-related oropharyngeal squamous cell carcinoma	Zenga J.; Pipkorn P.; Graboyes E.M.; Martin E.J.; Rich J.T.; Moore E.J.; Haughey B.H.; Jackson R.S.	2018	Head and Neck
217.	Resection of recurrent neck cancers with replacement of the carotid artery	Ricco J.-B.; Illuminati G.; Belmonte R.	2017	JMV-Journal de Medecine Vasculaire
218.	Head and neck soft tissue reconstruction with anterolateral thigh flaps with various components: Development of an algorithm for flap selection in different clinical scenarios	De Virgilio A.; Iocca O.; Di Maio P.; Malvezzi L.; Pellini R.; Mercante G.; Spriano G.	2019	Microsurgery
219.	Nationwide randomised trial evaluating elective neck dissection for early stage oral cancer (SEND study) with meta-analysis and concurrent real-world cohort	Hutchison I.L.; Ridout F.; Cheung S.M.Y.; Shah N.; Hardee P.; Surwald C.; Thiruchelvam J.; Cheng L.; Mellor T.K.; Brennan P.A.; Baldwin A.J.; Shaw R.J.; Halfpenny W.; Danford M.; Whitley S.; Smith G.; Bailey M.W.; Woodwards B.; Patel M.; McManners J.; Chan C.-H.; Burns A.; Praveen P.; Camilleri A.C.; Avery C.; Putnam G.; Jones K.; Webster K.; Smith W.P.; Edge C.; McVicar I.; Grew N.; Hislop S.; Kalavrezos N.; Martin I.C.; Hackshaw A.	2019	British Journal of Cancer

220.	Long-term follow-up of stereotactic radiosurgery for head and neck malignancies	Owen D.; Iqbal F.; Pollock B.E.; Link M.J.; Stien K.; Garces Y.I.; Brown P.D.; Foote R.L.	2015	Head and Neck
221.	Is immediate bony microsurgical reconstruction after head and neck tumor ablation associated with a higher rate of local recurrence?	Hanken H.; Wilkens R.; Riecke B.; Al-Dam A.; Tribius S.; Kluwe L.; Smeets R.; Heiland M.; Eichhorn W.; Grobe A.	2015	Journal of Cranio-Maxillofacial Surgery
222.	Transaxillary robotic modified radical neck dissection: a 5-year assessment of operative and oncologic outcomes	Kim M.J.; Lee J.; Lee S.G.; Choi J.B.; Kim T.H.; Ban E.J.; Lee C.R.; Kang S.-W.; Jeong J.J.; Nam K.-H.; Jo Y.S.; Chung W.Y.	2017	Surgical Endoscopy
223.	Impact of skeletal muscle mass on postoperative delirium in patients undergoing free flap repair after oral cancer resection	Makiguchi T.; Yamaguchi T.; Nakamura H.; Ogawa M.; Harimoto N.; Shirabe K.; Yokoo S.	2020	Journal of plastic surgery and hand surgery
224.	Impact of Sarcopenia on Outcomes of Autologous Head and Neck Free Tissue Reconstruction	Alwani M.M.; Jones A.J.; Novinger L.J.; Pittelkow E.; Bonetto A.; Sim M.W.; Moore M.G.; Mantravadi A.V.	2020	Journal of reconstructive microsurgery
225.	Microvascular anastomosis in reconstructive head and neck surgery	Remmert, S.	1995	Laryngo- Rhino- Otologie
226.	One-stage reconstruction of complex pharyngoesophageal, tracheal, and anterior neck defects	Yu, P.	2005	Plastic and Reconstructive Surgery
227.	Advantages of perforator flaps in reconstruction of complex defects of the head and neck	Guerra, A.B.; Lyons, G.D.; Dupin, C.L.; Metzinger, S.E.	2005	Ear, Nose and Throat Journal
228.	Functional and aesthetic outcome and survival after double free flap	Posch, N.A.S.; Mureau, M.A.M.; Dumans, A.G.; Hofer, S.O.P.	2007	Plastic and Reconstructive Surgery

	reconstruction in advanced head and neck cancer patients			
229.	Free-flap head and neck reconstruction and quality of life: A 2-year prospective study	Bozec, A.; Poissonnet, G.; Chamorey, E.; Casanova, C.; Vallicioni, J.; Demard, F.; Mahdyoun, P.; Peyrade, F.; Follana, P.; Bensadoun, R.-J.; Benezery, K.; Thariat, J.; Marcy, P.-Y.; Sudaka, A.; Dassonville, O.	2008	Laryngoscope
230.	Free flap reconstruction after previous neck dissection	Nakamura, R.; Hyodo, I.; Okumura, S.; Sawamoto, N.; Kuwata, T.; Kamei, Y.	2015	Japanese Journal of Head and Neck Cancer
231.	Reconstruction of Pharyngeal Defects Using AlloDerm and Sternocleidomastoid Muscle Flap	Sinha, U.K.; Chang, K.E.; Shih, C.W.	2001	Laryngoscope
232.	Use of couplers for vascular anastomoses in 601 free flaps for reconstruction of defects of the head and neck: technique and two-year retrospective clinical study	Assoumane, A.; Wang, L.; Liu, K.; Shang, Z.-J.	2017	British Journal of Oral and Maxillofacial Surgery
233.	Perforator based rectus free tissue transfer for head and neck reconstruction: New reconstructive advantages from an old friend	Kang, S.Y.; Spector, M.E.; Chepeha, D.B.	2017	Oral Oncology
234.	Comparison of standard modified shrobingers incision versus transverse cervical incision for neck dissection â€“ our experience	Agrawal, G.; Gupta, A.; Choraria, A.; Tiwari, S.; Chaudhary, V.	2018	Otolaryngology Case Reports
235.	The subcutaneous cervicofacial flap revisited	Austen Jr., W.G.; Parrett, B.M.; Taghinia, A.; Wolfort, S.F.; Upton, J.	2009	Annals of Plastic Surgery
236.	QOL after head and neck reconstruction: Evaluation of	Hikosaka, M.; Ochiai, H.; Fujii, M.; Habu, N.; Yajima, Y.; Sakurai, T.; Bito, S.	2011	Auris Nasus Larynx

	Japanese patients using SF-36 and GOHAI			
237.	Functional outcomes of fibula and osteocutaneous forearm free flap reconstruction for segmental mandibular defects.	Virgin FW; Iseli TA; Iseli CE; Sunde J; Carroll WR; Magnuson JS; Rosenthal EL	2010	Laryngoscope
238.	Enhanced morbidity of pectoralis major myocutaneous flap used for salvage after previously failed oncological treatment and unsuccessful reconstructive head and neck surgery.	Ribeiro Salles Vanni, Christiana Maria; Matos, Leandro Luongo de; Faro Junior, MÃ¡rio Paulo; Ledo Kanda, Jossi; Cernea, ClÃ¡udio Roberto; Garcia Brandao, Lenine; Pinto, FÃ¡bio Roberto	2012	Scientific World Journal
239.	Results after u-shaped pectoralis major myocutaneous flap reconstruction of circumferential pharyngeal defects.	Espitalier F; Ferron C; Leux C; JÃ©goux F; Durand N; Beauvillain de Montreuil C; Malard O	2012	Laryngoscope
240.	Sternoclavicular osteoradionecrosis following treatment for head and neck cancer.	Gehani, Neal; Ludin, Adir; Baskin, Jonathan Z	2013	American Journal of Otolaryngology
241.	Etiologic causes of late osteocutaneous free flap failures in oral cavity cancer reconstruction.	Zender CA; Mehta V; Pittman AL; Feustel PJ; Jaber JJ	2012	Laryngoscope
242.	The role of pectoralis major muscle flap in salvage total laryngectomy.	Gil Z; Gupta A; Kummer B; Cordeiro PG; Kraus DH; Shah JP; Patel SG	2009	Archives of Otolaryngology - Head & Neck Surgery
243.	Pectoralis myofascial flap during salvage laryngectomy prevents pharyngocutaneous fistula.	Patel UA; Keni SP	2009	Otolaryngology-Head & Neck Surgery
244.	Radial Forearm Free Flap Donor Site Morbidity: Ulnar-Based	Jaquet Y; Enepekides DJ; Torgerson C; Higgins KM	2012	Archives of Otolaryngology - Head & Neck Surgery

	Transposition Flap vs Split-Thickness Skin Graft.			
245.	The pectoralis major myofascial flap: clinical applications in head and neck reconstruction.	Righi RD; Weisberger EC; Slakes SR; Wilson JL; Kesler KA; Yaw PB; Righi, P D; Weisberger, E C; Slakes, S R; Wilson, J L; Kesler, K A; Yaw, P B	1998	American Journal of Otolaryngology
246.	The angular branch: maximizing the scapular pedicle in head and neck reconstruction.	Wagner AJ; Bayles SW	2008	Archives of Otolaryngology - Head & Neck Surgery
247.	Surgical techniques and results of lateral thoracic cutaneous, myocutaneous, and conjoint flaps for head and neck reconstruction.	Yuen AP; Ng RW	2007	Laryngoscope
248.	Salvage surgery for recurrent carcinoma of the hypopharynx and reconstruction using jejunal free tissue transfer and pectoralis major muscle pedicled flap.	Dubsky PC; Stift A; Rath T; Kornfehl J	2007	Archives of Otolaryngology - Head & Neck Surgery
249.	The use of the pectoralis major flap for advanced and recurrent head and neck malignancy in the medically compromised patient.	Avery CM; Crank ST; Neal CP; Hayter JP; Elton C	2010	Oral Oncology
250.	The Pectoralis Major Island Flap: Short Scar Modified Muscle-Sparing Harvesting Technique Improves Aesthetic Outcome in Reconstructive Head and Neck Surgery.	Rauchenwald, Tina; Dejaco, Daniel; Morandi, EviÂ M.; Djedovic, Gabriel; Wolfram, Dolores; Riechelmann, Herbert; Pierer, Gerhard; Morandi, Evi M	2019	ORL

251.	Role of craniofacial surgery in oral and maxillofacial tumors involving the skull base: A retrospective analysis of 126 patients.	Yang, Le; Li, Jing-Yuan; Xiao, Yu-Dong; Deng, Wei; Qiao, Yong-Jie; Liang, Yu-Jie; Liao, Gui-Qing	2020	Oral Surgery, Oral Medicine, Oral Pathology & Oral Radiology
252.	Donor-Site Morbidity after Fibula Transplantation in Head and Neck Tumor Patients: A Split-Leg Retrospective Study with Focus on Leg Stability and Quality of Life.	Attia, Sameh; Diefenbach, Jonas; Schmermund, Daniel; BÃttger, Sebastian; Pons-KÃ¼hnemann, JÃrn; Scheibelhut, Christine; Heiss, Christian; Howaldt, Hans-Peter	2020	Cancers
253.	Disease relapse after segmental resection and free flap reconstruction for mandibular osteoradionecrosis.	Suh JD; Blackwell KE; Sercarz JA; Cohen M; Liu JH; Tang CG; Abemayor E; Nabili V	2010	Otolaryngology-Head & Neck Surgery
254.	Application of chimerical ALT perforator flap with vastus lateralis muscle mass for the reconstruction of oral and submandibular defects after radical resection of tongue carcinoma: a retrospective cohort study.	Yang, Rong; Wu, Xiaoshan; Kumar, Pathak Ajit; Xiong, Yafei; Jiang, Canhua; Jian, Xinchun; Guo, Feng	2020	BMC Oral Health
255.	The effects of adjuvant chemoradiotherapy on survival in patients with primary laryngeal cancer with close surgical margins and positive surgical margins	Ä°nanÃ§, B.; Ä°nanÃ§, K.; Bilici, S.; Ã‡akir, M.; YiÄŸit, Ã–.	2020	Turk Onkoloji Dergisi
256.	Incidence and Clinical Analysis of Complications of Neck Dissection	Agrawal, S.M.; Anehosur, V.	2021	Indian Journal of Otolaryngology and Head and Neck Surgery
257.	Postoperative Pain Treatment with Continuous Local Anesthetic Wound Infusion in Patients with	Gostian, M.; Loeser, J.; Albert, C.; Wolber, P.; Schwarz, D.; Grosheva, M.; Veith, S.; Goerg, C.; Balk, M.; Gostian, A.-O.	2021	JAMA Otolaryngology - Head and Neck Surgery

	Head and Neck Cancer: A Nonrandomized Clinical Trial			
258.	The X-pointer: A forgotten anatomical relationship of spinal accessory nerve and great auricular nerve	Rao, V.; Subash, A.; Sinha, P.; Chatterjee, S.; Nayar, R.C.	2021	Surgical Oncology
259.	Supraclavicular artery island flap for head and neck reconstruction.	Sahin, Bayram; Ulusan, Murat; Basaran, Bora; Gunes, Selcuk; Oymak, Emre; Genc, Selahattin	2021	Acta chirurgiae plasticae
260.	Complications after Functional Neck Dissection in Head and Neck Cancer Patients: An Observational, Retrospective, Single-Centre Study.	Chiesa-Estomba, Carlos M; Soriano-Reixach, Maria; Thomas-Arrizabalaga, Izaskun; Sistiaga-Suarez, Jon A; Gonzalez-Garcia, Jose A; Larruscain, Ekhine; Altuna, Xabier	2021	Journal for otorhino laryngology
261.	Computed tomography-defined sarcopenia as a risk factor for short-term postoperative complications in oral cancer patients with free flap reconstruction: A retrospective population-based cohort study.	Lin, Bo; Lin, Jianlin; Wang, Feng; Wang, Yufan; Shen, Shiyue; Hong, Xia; Yang, HuiJun; Wang, Shunji; Yang, Hongyu	2023	Head & neck
262.	Resection and reconstruction of giant cervical metastatic cancer using a pectoralis major muscular flap transfer: A prospective study of 16 patients.	Xiangmin Zhang; Folin Liu; Xiaolin Lan; Jing Huang; Keqing Luo; Shaojin Li	2015	Oncology Letters
263.	Head and Neck Malignancies and Neck Dissection Complications: A Cohort Study from a Tertiary Care Centre in Telangana, India.	Rao, M. Mallikarjun; Jena, Shubranshu; Panigrahi, Rudra Prakash; Vashist, S. Kumar	2023	Journal of Clinical & Diagnostic Research
**Unclear Information**				

264.	Involvement of peripheral nerves in radical neck dissection.	Swift, T R	1970	American journal of surgery
265.	Considering the spinal accessory nerve in head and neck surgery.	Saunders, J R Jr; Hirata, R M; Jaques, D A	1985	American journal of surgery
266.	Shoulder mobility after spinal accessory nerve-sparing modified radical neck dissection in oral cancer patients.	Umeda, Masahiro; Shigeta, Takashi; Takahashi, Hidenori; Oguni, Akiko; Kataoka, Tomoko; Minamikawa, Tsutomu; Shibuya, Yasuyuki; Komori, Takahide	2010	Oral surgery, oral medicine, oral pathology, oral radiology, and endodontics
267.	Scapular muscle exercises following neck dissection surgery for head and neck cancer: a comparative electromyographic study.	McGarvey, Aoife C; Osmotherly, Peter Grant; Hoffman, Gary R; Chiarelli, Pauline E	2013	Physical therapy
268.	Shoulder function following reconstruction with the supraclavicular artery island flap.	Herr, Marc W; Bonanno, Andrea; Montalbano, Lisa A; Deschler, Daniel G; Emerick, Kevin S	2014	The Laryngoscope
269.	Motor and functional recovery after neck dissection: comparison of two early physical rehabilitation programmes.	Baggi, F; Santoro, L; Grosso, E; Zanetti, C; Bonacossa, E; Sandrin, F; Massaro, M A; Tradati, N; Simoncini, M C	2014	Acta otorhinolaryngologica Italica : organo ufficiale della Societa italiana di otorinolaringologia e chirurgia cervico-facciale
270.	Myofascial pain syndrome after head and neck cancer treatment: Prevalence, risk factors, and influence on quality of life.	Cardoso, Leticia Rodrigues; Rizzo, Claudia Carvalho; de Oliveira, Cleyton Zanardo; dos Santos, Carlos Roberto; Carvalho, Andre Lopes	2015	Head & neck
271.	Shoulder Dysfunction After Radiotherapy in Surgically and Nonsurgically Treated Necks: A Prospective Study.	Sun, Qiang; Guo, Shu; Wang, Di; Xu, Nan; Fang, Qi-gen	2015	Medicine

272.	Painful shoulder syndrome with drooping after cervical lymphatic dissection	Campos, A; Zapater, E; Barona, R; Montalt, J; Basterra, J	1996	Acta otorrinolaringologica espanola
273.	Radical neck dissection and the possibility of complications: surgical technique	Kurnatowski, P; Latkowski, B; Lukomski, M; Piotrowski, S	1996	Otolaryngologia polska = The Polish otolaryngology
274.	Morbidity of the neck after head and neck cancer therapy.	van Wilgen, C Paul; Dijkstra, Pieter U; van der Laan, Berend F A M; Plukker, John T; Roodenburg, Jan L N	2004	Head & neck
275.	Shoulder and neck morbidity in quality of life after surgery for head and neck cancer.	van Wilgen, C P; Dijkstra, P U; van der Laan, B F A M; Plukker, J Th; Roodenburg, J L N	2004	Head & neck
276.	Impact of Neck Disability on Health-Related Quality of Life among Head and Neck Cancer Survivors.	Nilsen, Marci L; Lyu, Lingyun; Belsky, Michael A; Mady, Leila J; Zandberg, Dan P; Clump, David A 2nd; Skinner, Heath D; Peddada, Shyamal Das; George, Susan; Johnson, Jonas T	2020	Otolaryngology--head and neck surgery : official journal of American Academy of Otolaryngology-Head and Neck Surgery
277.	Preservation of spinal accessory nerve during radical neck dissection	Nilakantan A.	2006	Medical Journal Armed Forces India
278.	Risk of marginal mandibular nerve injury in neck dissection	Moller M.N.; Sorensen C.H.	2011	European Archives of Oto-Rhino-Laryngology
279.	Removal of level IIB nodes during extended supraomohyoid neck dissection (I-IV) for oral tongue carcinoma	Prabu N.P.; Muthulingam V.; Swarapriya R.; Sargunar B.; Shamugapriyan	2017	Research Journal of Pharmaceutical, Biological and Chemical Sciences
280.	Functional and morphological findings following pedicled and microanastomosed tissue transfer	Esser, D.; Motsch, C.; Begall, K.; Buhtz, P.	1993	Laryngo- Rhino- Otologie

281.	RetroRESEARCH triangle. Disfigurement and dysfunction with head and neck cancer surgery.	Dropkin MJ	1998	ORL-Head & Neck Nursing
282.	Dissection of Levels II Through V Is Required for Optimal Outcomes in Patients with Lateral Neck Lymph Node Metastasis from Papillary Thyroid Carcinoma.	Javid, Mahsa; Graham, Emma; Malinowski, Jennifer; Quinn, Courtney E.; Carling, Tobias; Udelsman, Robert; Callender, Glenda G.	2016	Journal of the American College of Surgeons
283.	Level V cervical lymph node involvement in patients with stage N1b papillary thyroid carcinoma: a prospective study.	Alwagih, Hatem; Hamza, Yasser; Hamza, Alaa; Hamed, Ahmed; Gabr, Essam	2022	Egyptian Journal of Surgery
**Not possible to isolate the intervention**				
284.	Disability and rehabilitation in head and neck cancer patients after treatment.	Olson, M L; Shedd, D P	1978	Head & neck surgery
285.	Parascapular free flaps for head and neck reconstruction.	Chandrasekhar, B; Lorant, J A; Terz, J J	1990	American journal of surgery
286.	Accessory nerve conduction in neck dissection subjects.	Shankar, K; Means, K M	1990	Archives of physical medicine and rehabilitation
287.	Predictors of functional decline in locally advanced head and neck cancer patients from south Brazil.	Silver, Heidi J; de Campos Graf Guimaraes, Christine; Pedruzzi, Paola; Badia, Maraisa; Spuldaro de Carvalho, Adriana; Oliveira, Benedito V; Ramos, Gyl H A; Dietrich, Mary S; Pietrobon, Ricardo	2010	Head & neck
288.	A prospective cohort study of fibula free flap donor-site morbidity in 157 consecutive patients.	Momoh, Adeyiza O; Yu, Peirong; Skoracki, Roman J; Liu, Suyu; Feng, Lei; Hanasono, Matthew M	2011	Plastic and reconstructive surgery

289.	Minimizing shoulder syndrome with intra-operative spinal accessory nerve monitoring for neck dissection.	Lee, C-H; Huang, N-C; Chen, H-C; Chen, M-K	2013	Acta otorhinolaryngologica Italica : organo ufficiale della Societa italiana di otorinolaringologia e chirurgia cervico-facciale
290.	Complications of neck dissection at a tertiary level hospital: study of 30 cases.	Masud, M K; Ahmad, S M; Karim, M A; Ferdouse, F; Fakir, A Y; Hanif, M A; Abdullah, M; Roy, A S	2014	Mymensingh medical journal : MMJ
291.	Pectoralis Major Myocutaneous Flap in Primary and Salvage Head and Neck Cancer Surgery.	Anicin, Aleksandar; Sifrer, Robert; Strojan, Primoz	2015	Journal of oral and maxillofacial surgery
292.	Patient-reported outcomes for dental health, shoulder-neck dysfunction, and overall quality of life after treatment with radiation for head and neck cancer.	Verma, Neha; Tan, Xianming; Knowles, Mary; Bernard, Stephen; Chera, Bhishamjit	2019	Laryngoscope investigative otolaryngology
293.	Pedicled galeal flap in the reconstruction of head and neck tumor defects	Zhang, B; Tang, P; Qi, Y; Xu, Z; Wu, Y	2000	Zhonghua er bi yan hou ke za zhi
294.	Early use of a mechanical stretching device to improve mandibular mobility after composite resection: a pilot study.	Cohen, Erik G; Deschler, Daniel G; Walsh, Kathleen; Hayden, Richard E	2005	Archives of physical medicine and rehabilitation
295.	Transpositional anastomosis of C7 posterior root and spinal accessory nerve to reconstruct the trapezius muscle function.	Sun, Jian; Li, Jun; Jiang, Jidang	2005	Chinese journal of reparative and reconstructive surgery
296.	Treatment of pain after head and neck surgeries: control of acute pain	Gil, Ziv; Smith, Darryl B; Marouani, Nissim; Khafif, Avi; Fliss, Dan M	2006	Journal of American Academy of Otolaryngology-Head and Neck Surgery

	after head and neck oncological surgeries.			
297.	The rate of facial nerve dysfunction and time to recovery after intraparotid and extraparotid facial nerve exposure and protection in head and neck cutaneous tumor surgery.	Nakamura, Yasuhiro; Teramoto, Yukiko; Asami, Yuri; Imamura, Taichi; Sato, Sayuri; Tanaka, Ryota; Maruyama, Hiroshi; Nakamura, Yoshiyuki; Fujisawa, Yasuhiro; Fujimoto, Manabu; Yamamoto, Akifumi	2017	International journal of clinical oncology
298.	Long-term donor site morbidity in head and neck cancer patients and its impact on quality of life: a cross-sectional study.	Kansy, K; Hoffmann, J; Alhalabi, O; Mistele, N; Freier, K; Shavlokhova, V; Mertens, C; Freudlsperger, C; Engel, M	2019	International journal of oral and maxillofacial surgery
299.	Performance status scale for head and neck scores for oral cancer survivors: predictors and factors for improving quality of life.	Kondo, Takahide; Sugauchi, Akinari; Yabuno, Yusuke; Kobashi, Hironobu; Amano, Katsuhiko; Aikawa, Tomonao; Kogo, Mikihiko; Okura, Masaya	2019	Clinical oral investigations
300.	Presurgery and Postsurgery Quality of Life and Associated Factors in Patients With Malignant Neoplasms of the Head and Neck: A 6-Month Follow-up Study.	Hu, Zhiping; Zhou, Xiaoling; Duan, Weiwei; Lei, Wenge; Zhang, Min; Zeng, Lingxia	2019	Cancer nursing
301.	Treatment modality impact on quality of life for human papillomavirus-associated oropharynx cancer.	Xu, Mary Jue; Plonowska, Karolina A; Gurman, Zev R; Humphrey, Amanda K; Ha, Patrick K; Wang, Steven J; El-Sayed, Ivan H; Heaton, Chase M; George, Jonathan R; Yom, Sue S; Algazi, Alain P; Ryan, William R	2020	The Laryngoscope
302.	Surgical rescue for persistent head and neck cancer after first-line treatment.	Steinbichler, Teresa Bernadette; Golm, L; Dejaco, D; Riedl, D; Kofler, B; Url, C; Wolfram, D; Riechelmann, H	2020	European archives of otorhino-laryngology

303.	Radial forearm free flap donor site outcomes comparison by closure methods	Ho T.; Couch M.; Carson K.; Schimberg A.; Manley K.; Byrne P.J.	2006	Otolaryngology - Head and Neck Surgery
304.	Randomized trial of postoperative reirradiation combined with chemotherapy after salvage surgery compared with salvage surgery alone in head and neck carcinoma	Janot F.; De Raucourt D.; Benhamou E.; Ferron C.; Dolivet G.; Bensadoun R.-J.; Hamoir M.; Gery B.; Julieron M.; Castaing M.; Bardet E.; Gregoire V.; Bourhis J.	2008	Journal of Clinical Oncology
305.	Trismus following treatment of head and neck cancer	Jeremic G.; Venkatesan V.; Hallock A.; Scott D.; Hammond A.; Read N.; Franklin J.; Yoo J.; Fung K.	2011	Journal of Otolaryngology - Head and Neck Surgery
306.	Effect of fibula free flap harvest on the gait of head and neck cancer patients: Preliminary results	Macdonald K.I.; Taylor S.M.; Trites J.R.B.; Fung E.W.; Barnsley P.G.; Dunbar M.J.; Leahey J.L.; Hart R.D.	2011	Journal of Otolaryngology - Head and Neck Surgery
307.	Quality of life and swelling of the head, neck and arm afterneck dissection	Maune S.; Heissenberg M.C.; Schmidt C.; Rzehak P.; Kuchler T.	1999	European Journal of Lymphology and Related Problems
308.	National trends in surgery for sinonasal malignancy and the effect of hospital volume on short-term outcomes	Ouyang D.; El-Sayed I.H.; Yom S.S.	2014	Laryngoscope
309.	Exercise intervention for the treatment of trismus in head and neck cancer	Pauli N.; Fagerberg-Mohlin B.; Andrell P.; Finizia C.	2014	Acta Oncologica
310.	Prevalence and prediction of trismus in patients with head and neck cancer: A cross-sectional study	van der Geer S.J.; van Rijn P.V.; Kamstra J.I.; Langendijk J.A.; van der Laan B.F.A.M.; Roodenburg J.L.N.; Dijkstra P.U.	2019	Head and Neck

311.	Neck dissection following concurrent chemoradiation for advanced head and neck carcinoma: Pathologic findings and complications	Christopoulos A.; Nguyen-Tan P.F.; Tabet J.-C.; Fortin B.; Soulieres D.; Charpentier D.; Guertin L.	2008	Journal of Otolaryngology - Head and Neck Surgery
312.	The assessment of the disability of upper extremities and the quality of life (QOL) in patients who underwent neck dissection, using with the Japanese version of the DASH questionnaire	Kimura, S.; Ogino, M.; Kamura, Y.; Fujioka, S.; Tatehara, S.; Iritani, K.; Hayashi, T.; Matsui, H.; Yonezawa, K.; Hirayama, Y.; Iwae, S.	2016	Japanese Journal of Head and Neck Cancer
313.	Pain in Head and Neck Cancer Survivors: Prevalence, Predictors, and Quality-of-Life Impact	Cramer, J.D.; Johnson, J.T.; Nilsen, M.L.	2018	Otolaryngology - Head and Neck Surgery (United States)
314.	Regional flaps in head and neck reconstruction: a reappraisal.	Colletti, Giacomo; Tewfik, Karim; Bardazzi, Alessandro; Allevi, Fabiana; Chiapasco, Matteo; MandalÃ , Marco; Rabbiosi, Dimitri	2015	Journal of Oral & Maxillofacial Surgery
315.	Dynamics of quality of life of head and neck cancer patients after treatment. Clinical significance	Karpenko, A.V.; Sibgatullin, R.R.; Boyko, A.A.; Kostova, M.G.; Nikolayeva, O.M.; Trunin, E.M.	2019	Opuholi Golovy i Sei
316.	Assessment of Quality of Life of Free Anterolateral Thigh Flap for Reconstruction of Tissue Defects of Total or Near-Total Glossectomy	Zhang, S.; Wu, S.; Liu, L.; Zhu, D.; Zhu, Q.; Li, W.	2020	Journal of Oncology
317.	Motor control integrated into muscle strengthening exercises has more effects on scapular muscle activities and joint range of motion before initiation of radiotherapy in	Chen, Y.H.; Lin, C.R.; Liang, W.A.; Huang, C.Y.	2020	PLoS ONE

	oral cancer survivors with neck dissection: A randomized controlled trial			
318.	Reproducibility of measurements on physical performance in head and neck cancer survivors; measurements on maximum mouth opening, shoulder and neck function, upper and lower body strength, level of physical mobility, and walking ability	van Hinte, G.; Leijendekkers, R.A.; te Molder, B.; Jansen, L.; Bol, C.; Merkx, M.A.W.; Takes, R.; Nijhuis-Van der Sanden, M.W.G.; Speksnijder, C.M.	2020	PLoS ONE
319.	Shoulder function after neck dissection: Assessment via a shoulder-specific quality-of-life questionnaire and active shoulder abduction	Imai, T.; Sato, Y.; Abe, J.; Kumagai, J.; Morita, S.; Saijo, S.; Yamazaki, T.; Asada, Y.; Matsuura, K.	2021	Auris Nasus Larynx
320.	ERAS for Head and Neck Tissue Transfer Reduces Opioid Usage, Peak Pain Scores, and Blood Utilization	Clark, B.S.; Swanson, M.; Widjaja, W.; Cameron, B.; Yu, V.; Ershova, K.; Wu, F.M.; Vanstrum, E.B.; Ulloa, R.; Heng, A.; Nurimba, M.; Kokot, N.; Kochhar, A.; Sinha, U.K.; Kim, M.P.; Dickerson, S.	2021	Laryngoscope
321.	Patient-focused management of shoulder disability post-neck dissection: A pilot study	Neralla, M.; Rajan, J.; George, R.	2021	International Journal of Dentistry and Oral Science
322.	Management and prevention of brachial plexus injury caused by surgical suture of neck dissection induced chylous fistula.	Wang, Mei; Yuan, Xiaohui; Chen, Yongzheng; Wu, Jianfang; Tian, Shu; Wu, Chunping	2022	American journal of otolaryngology
323.	Effect of progressive muscle relaxation on postoperative pain,	Loh, El-Wui; Shih, Huei-Fen; Lin, Chung-Kwei; Huang, Tsai-Wei	2022	Patient education and counseling

	fatigue, and vital signs in patients with head and neck cancers: A randomized controlled trial.			
324.	Persistent Postsurgical Pain in Oral Cancer Patients Reconstructed with Anterolateral Thigh Free Flap.	Shen, Ya-Chun; Liao, Kuei-Lin; Cheng, Kuang-I; Tseng, Kuang-Yi; Su, Miao-Pei	2022	Medicina (Kaunas, Lithuania)
325.	Motor control integrated into muscle strengthening exercises has more effects on scapular muscle activities and joint range of motion before initiation of radiotherapy in oral cancer survivors with neck dissection: A randomized controlled trial.	Chen, Yueh-Hsia; Lin, Chi-Rung; Liang, Wei-An; Huang, Cheng-Ya	2020	PLoS ONE
326.	Factors influencing neck and shoulder function after oral oncology treatment: a five-year prospective cohort study in 113 patients.	van Hinte, Gerben; Merkx, Matthias A. W.; Wetzels, Jan-Willem G. H.; Speksnijder, Caroline M.; Koole, Ron; de Haan, Anton F. J.	2019	Supportive Care in Cancer
327.	Radiotherapy versus transoral robotic surgery and neck dissection for oropharyngeal squamous cell carcinoma (ORATOR): an open-label, phase 2, randomised trial.	Nichols, Anthony C; Theurer, Julie; Prisman, Eitan; Read, Nancy; Berthelet, Eric; Tran, Eric; Fung, Kevin; de Almeida, John R; Bayley, Andrew; Goldstein, David P; Hier, Michael; Sultanem, Khalil; Richardson, Keith; Mlynarek, Alex; Krishnan, Suren; Le, Hien; Yoo, John; MacNeil, S Danielle; Winquist, Eric; Hammond, J Alex	2019	Lancet Oncology

328.	Assessment of post-surgical donor site morbidity in vastus lateralis free flap for head and neck reconstructive surgery: an observational study.	Seth, Ishith; Hewitt, Lyndel; Yabe, Takako; Dunn, Masako; Wykes, James; Clark, Jonathan R.; Ashford, Bruce	2021	ANZ Journal of Surgery
329.	Neck Disability and Swallowing Function in Posttreatment Head and Neck Cancer Patients	Harris, A.; Lyu, L.; Wasserman-Winko, T.; George, S.; Johnson, J.T.; Nilsen, M.L.	2020	Otolaryngology - Head and Neck Surgery (United States)
330.	A randomized controlled trial of scapular exercises with electromyography biofeedback in oral cancer patients with accessory nerve dysfunction.	Chen, Yueh-Hsia; Liang, Wei-An; Lin, Chi-Rung; Huang, Cheng-Ya	2022	Supportive care in cancer : official journal of the Multinational Association of Supportive Care in Cancer
331.	Effects of Conscious Control of Scapular Orientation in Oral Cancer Survivors With Scapular Dyskinesis: A Randomized Controlled Trial.	Chen, Yueh-Hsia; Huang, Cheng-Ya; Liang, Wei-An; Lin, Chi-Rung; Chao, Yuan-Hung	2021	Integrative cancer therapies
332.	Radiotherapy versus transoral robotic surgery and neck dissection for oropharyngeal squamous cell carcinoma (ORATOR): an open-label, phase 2, randomised trial.	Nichols, Anthony C; Theurer, Julie; Prisman, Eitan; Read, Nancy; Berthelet, Eric; Tran, Eric; Fung, Kevin; de Almeida, John R; Bayley, Andrew; Goldstein, David P; Hier, Michael; Sultanem, Khalil; Richardson, Keith; Mlynarek, Alex; Krishnan, Suren; Le, Hien; Yoo, John; MacNeil, S Danielle; Winquist, Eric; Hammond, J Alex	2019	Lancet Oncology
333.	Pain management with popliteal block for fibular graft harvesting in head and neck reconstruction; a	Persson, Karolina; SjÃ¶vall, Johanna; Kander, Thomas; Walther-Sturesson, Louise	2022	Oral Oncology

	randomised double-blind placebo-controlled study.			
334.	A prospective evaluation of neck and shoulder function following treatments of early-stage human papillomavirus-associated oropharynx cancer.	Gulati, Arushi; Plonowska-Hirschfeld, Karolina; Stephens, Erika M; Kansara, Sagar; Zebolsky, Aaron L; Ochoa, Edgar; Xu, Mary J; Ha, Patrick K; Heaton, Chase M; Yom, Sue S; Chan, Jason W; Algazi, Alain P; Kang, Hyunseok; Ryan, William R	2023	Clinical otolaryngology : official journal of ENT-UK ; official journal of Netherlands Society for Oto-Rhino-Laryngology & Cervico-Facial Surgery
335.	Functional Outcomes in Head and Neck Cancer Patients.	Riechelmann, Herbert; Dejaco, Daniel; Steinbichler, Teresa Bernadette; Lettenbichler-Haug, Anna; Anegg, Maria; Ganswindt, Ute; Gamerith, Gabriele; Riedl, David	2022	Cancers
336.	Prospective quality of life outcomes for human papillomavirus associated oropharynx cancer patients after surgery alone.	Stephens, Erika M; Plonowska-Hirschfeld, Karolina; Gulati, Arushi; Kansara, Sagar; Qualliotine, Jesse; Zelbolsky, Aaron L; van Zante, Annemieke; Ha, Patrick K; Heaton, Chase M; Ryan, William R	2023	American journal of otolaryngology
337.	Long-term patient-reported donor-site morbidity after free peroneal fasciocutaneous flap in head and neck reconstruction	Lee, S.-Y.; Yang, K.-C.; Lin, C.-T.; Ho, Y.-Y.; Chen, L.-W.; Liu, W.-C.	2023	Journal of International Medical Research
338.	Dynamometric outcomes of the donor site leg after vastus lateralis free flap harvest	Mihovilovic, A.; Martinovic, D.; Martinic, J.; Markovic, D.; Tarle, M.; Jerkovic, D.; Vuk, S.; Dediol, E.	2023	Journal of Cranio-Maxillofacial Surgery
339.	Effect of Manual Pressure Release and Scapular Stabilization Exercises	Mohammed Attia, Alzahraa Mohammed; Aboelnour, Nancy Hassan; Sherif, Ragab Ali; Saafaan, Karim Ibrahim	2022	Egyptian Journal of Hospital Medicine

	on Myofascial Pain Syndrome Following Neck Dissection Surgery.			
**Not related to neck dissection (not related to objective of the review)**				
340.	Microsurgery for macrodefects: microvascular free-tissue transfer for massive defects of the head and neck.	Hardesty, R A; Jones, N F; Swartz, W M; Ramasastry, S S; Heckler, F D; Newton, E D; Schramm, V L	1987	American journal of surgery
341.	Mandibular reconstruction with composite microvascular tissue transfer.	Coleman, J J 3rd; Wooden, W A	1990	American journal of surgery
342.	Assessing quality of life in patients treated for advanced head and neck cancer.	Rathmell, A J; Ash, D V; Howes, M; Nicholls, J	1991	Clinical oncology (Royal College of Radiologists (Great Britain))
343.	Types and causes of pain in cancer of the head and neck.	Vecht, C J; Hoff, A M; Kansen, P J; de Boer, M F; Bosch, D A	1992	Cancer
344.	Anterolateral thigh cutaneous flap vs. radial forearm free-flap in oral and oropharyngeal reconstruction: an analysis of 48 flaps.	Camaioni, A; Loreti, A; Damiani, V; Bellioni, M; Passali, F M; Viti, C	2008	Acta otorhinolaryngologica Italica
345.	Immediate impact of primary surgery on health-related quality of life of hospitalized patients with oral and oropharyngeal cancer.	Biazevic, Maria G H; Antunes, Jose Leopoldo Ferreira; Togni, Janina; de Andrade, Fabiana P; de Carvalho, Marcos B; Wunsch-Filho, Victor	2008	Journal of oral and maxillofacial surgery : official journal of the American Association of Oral and Maxillofacial Surgeons
346.	Assessment of donor-site morbidity using balance and gait tests after bilateral fibula osteoseptocutaneous free flap transfer.	Lin, Jeng-Yee; Djohan, Risal; Dobryansky, Michael; Chou, Shih-Wei; Hou, Wen-Hsuan; Chen, Ming-Huei; Wei, Fu-Chan	2009	Annals of plastic surgery
347.	Maxillary reconstruction with the scapular angle osteomyogenous free flap.	Miles, Brett A; Gilbert, Ralph W	2011	Archives of otolaryngology--head & neck surgery

348.	Donor site morbidity of free ulnar forearm flap.	Tan, Swee T; James, Dylan W; Moaveni, Zachary	2012	Head & neck
349.	Cranial nerve dysfunction following Gamma Knife surgery for pituitary adenomas: long-term incidence and risk factors.	Cifarelli, Christopher P; Schlesinger, David J; Sheehan, Jason P	2012	Journal of neurosurgery
350.	Simultaneous reconstruction of head and neck defects following tumor resection and trismus release with a single anterolateral thigh donor site utilizing a lateral approach to flap harvest.	Lin, Pao-Yuan; Chen, Chien-Chung; Kuo, Yur-Ren; Jeng, Seng-Feng	2012	Microsurgery
351.	Outcome, general, and symptom-specific quality of life after various types of parotid resection.	Ciuman, Raphael Richard; Oels, Wolfgang; Jaussi, Rolf; Dost, Philipp	2012	The Laryngoscope
352.	Assessment of quality of life of patients with oral cavity cancer who have had defects reconstructed with free anterolateral thigh perforator flaps.	Li, Wenlu; Yang, Yanjie; Xu, Zhongfei; Liu, Fayu; Cheng, Yusheng; Xu, Lei; Sun, Changfu	2013	The British journal of oral & maxillofacial surgery
353.	Home-based exercise on functional outcome of the donor lower extremity in oral cancer patients after fibula flap harvest.	Liu, Ting-Yuan; Huang, Yu-Chi; Leong, Chau-Peng; Tseng, Chiung-Yi; Kuo, Yur-Ren	2013	Biomedical journal
354.	The use of local flaps in the one-step nose reconstruction after cancer resection.	Chiummariello, Stefano; Del Torto, Giuseppe; Iera, Marco; Alfano, Carmine	2013	Annali italiani di chirurgia
355.	Reconstructive techniques of the parotid region.	Dell'aversana Orabona, Giovanni; Salzano, Giovanni; Petrocelli, Marzia; Iaconetta, Giorgio; Califano, Luigi	2014	The Journal of craniofacial surgery

356.	Long-term quality of life in survivors of head and neck cancer who have had defects reconstructed with radial forearm free flaps.	Li, Peng; Zhang, Xu; Luo, Rui-Hua; Zhao, Ming; Liu, Shan-Ting; Du, Wei; Qi, Jin-Xing	2015	The Journal of craniofacial surgery
357.	The use of health related quality of life data to produce information sheets for patients with head and neck cancer.	Rogers, S N; Hogg, E S; Cheung, W K A; Lai, L K L; Jassal, P; Lowe, D	2015	Annals of the Royal College of Surgeons of England
358.	The sequellae of donor sites. Functional results of the treatment of cancer of the oral cavity	Siberchicot, F	1997	Revue de stomatologie et de chirurgie maxillo-faciale
359.	Electromyography of the infrahyoid muscles - part 2: pathological findings	Remmert, S; Klostermann, W; Wessel, K; Gehrking, E	2001	Laryngo- rhino-otologie
360.	Donor site morbidity after microvascular fibula transfer.	Zimmermann, C E; Borner, B I; Hasse, A; Sieg, P	2001	Clinical oral investigations
361.	The temporalis muscle flap for reconstruction after head and neck oncologic surgery.	Hanasono, M M; Utley, D S; Goode, R L	2001	The Laryngoscope
362.	Disability in patients with head and neck cancer.	Taylor, Joseph C; Terrell, Jeffrey E; Ronis, David L; Fowler, Karen E; Bishop, Carol; Lambert, Michael T; Myers, Larry L; Duffy, Sonia A; Bradford, Carol R; Chepeha, Douglas B; Hogikyan, Norman D; Prince, Mark E; Teknos, Theodoos N; Wolf, Gregory T; University of Michigan Head and Neck Cancer Team	2004	Archives of otolaryngology--head & neck surgery
363.	Superiorly based and island masseter muscle flaps for repairing oropharyngeal defects.	Antoniades, Kostas; Lasaridis, Nikos; Vahtsevanos, Kostas; Hadjipetrou,	2005	Journal of cranio-maxillo-facial surgery

		Loukia; Antoniades, Vasilis; Karakasis, Dimitris		
364.	Donor site choice for free flaps in head and neck reconstruction after tumor surgery	Zhang, Bi; Yamada, Atsushi	2005	Chinese journal of reparative and reconstructive surgery
365.	Donor site morbidity of the anterolateral thigh flap.	Weise, Hannes; Naros, Andreas; Blumenstock, Gunnar; Krimmel, Michael; Hoefert, Sebastian; Kluba, Susanne; Hofer, Stefan; Reinert, Siegmar	2017	Journal of cranio-maxillo-facial surgery
366.	Free Flap Head and Neck Reconstruction with an Emphasis on Postoperative Care.	van Gijn, Daniel Richard; D'Souza, Jacob; King, Wendy; Bater, Michael	2018	Facial plastic surgery: FPS
367.	Application of robot-assisted surgery in the surgical treatment of head and neck cancer	Du, Y F; Chen, N; Li, D Q	2019	Chinese journal of stomatology
368.	Myofascial pain, widespread pressure hypersensitivity, and hyperalgesia in the face, neck, and shoulder regions, in survivors of head and neck cancer.	Ortiz-Comino, L; Fernandez-Lao, C; Castro-Martin, E; Lozano-Lozano, M; Cantarero-Villanueva, I; Arroyo-Morales, M; Martin-Martin, L	2020	Journal of the Multinational Association of Supportive Care in Cancer
369.	Achieving mandibular continuity with vascular bone flaps: A comparison of primary and secondary reconstruction	Markowitz B.; Taleisnik A.; Calcaterra T.; Shaw W.	1994	Journal of Oral and Maxillofacial Surgery
370.	Comparison of quality of life outcomes in laryngeal cancer patients following chemoradiation vs. total laryngectomy	Lotempio M.M.; Wang K.H.; Sadeghi A.; Delacure M.D.; Juillard G.F.; Wang M.B.	2005	Otolaryngology - Head and Neck Surgery

371.	Evaluation of the donor site in patients who underwent reconstruction with a free radial forearm flap	Ito O.; Igawa H.H.; Suzuki S.; Muneuchi G.; Kawazoe T.; Saso Y.; Onodera M.; Park S.; Hata Y.	2005	Journal of Reconstructive Microsurgery
372.	Quality of Life Evaluation for Patients Receiving Vascularized Versus Nonvascularized Bone Graft Reconstruction of Segmental Mandibular Defects	Vu D.D.; Schmidt B.L.	2008	Journal of Oral and Maxillofacial Surgery
373.	Reconstruction of Oromandibular Defects by Vascularized Free Flaps: The Radial Forearm Free Flap and Fibular Free Flap as Major Donor Sites	Gonzalez-Garcia R.; Naval-Gias L.; Rodriguez-Campo F.J.; Roman-Romero L.	2009	Journal of Oral and Maxillofacial Surgery
374.	Transoral resection for squamous cell carcinoma of the base of the tongue	Henstrom D.K.; Moore E.J.; Olsen K.D.; Kasperbauer J.L.; McGree M.E.	2009	Archives of Otolaryngology - Head and Neck Surgery
375.	Postoperative functional investigation of donor site with free anterolateral thigh flaps to reconstruct head and neck defects	Liu W.-W.; Guo Z.-M.; Zhang Q.; Yang A.-K.; Li H.; Liu X.-K.; Song M.	2011	Chinese Journal of Cancer Prevention and Treatment
376.	Suitability of the anterolateral thigh perforator flap and the soleus perforator flap for intraoral reconstruction: A retrospective study	Wolff K.-D.; Holzle F.; Kolk A.; Hohlweg-Majert B.; Kesting M.R.	2011	Journal of Reconstructive Microsurgery
377.	Longitudinal evaluation of restricted mouth opening (trismus) in patients following primary	Scott B.; D'Souza J.; Perinparajah N.; Lowe D.; Rogers S.N.	2011	British Journal of Oral and Maxillofacial Surgery

	surgery for oral and oropharyngeal squamous cell carcinoma			
378.	The submental flap for oral cavity reconstruction: Extended indications and technical refinements	Amin A.A.; Sakkary M.A.; Khalil A.A.; Rifaat M.A.; Zayed S.B.	2011	Head and Neck Oncology
379.	Incidence of trismus in head and neck cancer	Finizia C.A.	2012	Otolaryngology - Head and Neck Surgery (United States)
380.	Oromandibular reconstruction	Cannon T.Y.; Strub G.M.; Yawn R.J.; Day T.A.	2012	Clinical Anatomy
381.	Early effectiveness of posterior radial collateral artery perforator compound flap for reconstruction of tongue defects after tumor excision	Chen Y.; Yang X.; Li W.; Hu Q.	2012	Chinese journal of reparative and reconstructive surgery
382.	Functional outcomes of the retromaxillary-infratemporal fossa dissection for advanced head and neck/skull base lesions	Shibuya T.Y.; Doerr T.D.; Mathog R.H.; Burgio D.L.; Meleca R.J.; Yoo G.H.; Guthikonda M.	2000	Skull Base Surgery
383.	Assessment of quality of life in patients treated by supracricoid partial laryngectomy with cricohyoidoepiglottopexy (CHEP)	Marquez Moyano J.A.; Sanchez Gutierrez R.; Roldan Nogueras J.; Ostos Aumente P.; Lopez Villarejo P.	2004	Acta Otorrinolaringologica Espanola
384.	Functional outcome after different oncological interventions in head and neck cancer patients	Mucke T.; Koschinski J.; Wagenpfeil S.; Wolff K.-D.; Kanatas A.; Mitchell D.A.; Deppe H.; Kesting M.R.	2012	Journal of Cancer Research and Clinical Oncology
385.	Chimaeric subscapular system free flap for complex oro-facial defects	L'Heureux-Lebeau B.; Odobescu A.; Harris P.G.; Guertin L.; Danino A.M.	2013	Journal of Plastic, Reconstructive and Aesthetic Surgery
386.	Comparison between anterolateral thigh perforator free flaps and	Xiao Y.; Zhu J.; Cai X.; Wang J.; Liu F.; Wang H.	2013	Medicina Oral, Patologia Oral y Cirugia Bucal

	pectoralis major pedicled flap for reconstruction in oral cancer patients-A quality of life analysis			
387.	Carcinoma of the nasal cavity and paranasal sinuses	Mendenhall W.M.; Amdur R.J.; Morris C.G.; Kirwan J.; Malyapa R.S.; Vaysberg M.; Werning J.W.; Mendenhall N.P.	2009	Laryngoscope
388.	Reconstruction techniques for hypopharyngeal and cervical esophageal carcinoma	Jiang M.; He X.; Wu D.; Han Y.; Zhang H.; Wang M.	2015	Journal of Thoracic Disease
389.	Comprehensive Analysis of Functional Outcomes and Survival After Microvascular Reconstruction of Glossectomy Defects	Chang E.I.; Yu P.; Skoracki R.J.; Liu J.; Hanasono M.M.	2015	Annals of Surgical Oncology
390.	Reverse Superior Labial Artery Flap in Reconstruction of Nose and Medial Cheek Large Defects	Brunetti B.; Campa S.; Tenna S.; Persichetti P.	2016	Annals of plastic surgery
391.	Reducing Trismus after Surgery and Radiotherapy in Oral Cancer Patients: Results of Alternative Operation Versus Traditional Operation	Tsai C.-C.; Wu S.-L.; Lin S.-L.; Ko S.-Y.; Chiang W.-F.; Yang J.-W.	2016	Journal of Oral and Maxillofacial Surgery
392.	Surgical management of osteoradionecrosisand their outcomes	Murthya S.P.; Anand A.; Balasubramaniana D.; Rathoda V.P.; Limbachiyaa S.; Thankappana K.; Iyer S.	2017	Head and Neck
393.	Facial mimetic, cosmetic, and functional standardized assessment of the facial artery musculomucosal (FAMM) flap	Jowett N.; Hadlock T.A.; Sela E.; Toth M.; Knecht R.; Lorincz B.B.	2017	Auris Nasus Larynx
394.	Postoperative pain management in head and neck cancer surgery	Garnier F.; Bouroche G.; Sitbon P.; Bourgain J.L.	2017	Praticien en Anesthesie Reanimation

395.	Functional outcomes and quality of life after a 6-month early intervention program for oral cancer survivors: A single-arm clinical trial	Chen Y.-H.; Liang W.-A.; Hsu C.-Y.; Guo S.-L.; Lien S.-H.; Tseng H.-J.; Chao Y.-H.	2018	PeerJ
396.	Quality of Life after Free Fibula Flap Reconstruction of Segmental Mandibular Defects	Lofstrand J.; Nyberg M.; Karlsson T.; Thorarinsson A.; Kjeller G.; Liden M.; Frojd V.	2018	Journal of Reconstructive Microsurgery
397.	Surgical management of severe osteoradionecrosis of the mandibular bone by using double free flap reconstruction	Rommel N.; Kesting M.R.; Rohleder N.H.; Wolff K.-D.; Weitz J.	2018	Journal of Cranio-Maxillofacial Surgery
398.	Long-term quality of life after ablative intraoral tumour surgery	Schliephake, H.; Neukam, F.W.; Schmelzeisen, R.; Varoga, B.; Schneller, H.	1995	Journal of Cranio-Maxillofacial Surgery
399.	The suprafascially elevated radial forearm flap - Indications, technique, and donor-site morbidity	Lutz, B.S.; Chang, S.C.-N.; Chuang, S.-S.; Wei, F.-C.	1999	Handchirurgie Mikrochirurgie Plastische Chirurgie
400.	Comparison of radial forearm free flap, pedicled buccal fat pad flap and split-thickness skin graft in reconstruction of buccal mucosal defect	Chien, C.-Y.; Hwang, C.-F.; Chuang, H.-C.; Jeng, S.-F.; Su, C.-Y.	2005	Oral Oncology
401.	The versatility of the free lateral arm flap in head and neck soft tissue reconstruction: clinical experience of 210 cases	Marques Faria, J.C.; Rodrigues, M.L.; Scopel, G.P.; Kowalski, L.P.; Ferreira, M.C.	2008	Journal of Plastic, Reconstructive and Aesthetic Surgery
402.	Pain, function, and psychologic outcome before, during, and after intraoral tumor resection.	Gellrich N; Schimming R; Schramm A; Schmalohr D; Bremerich A; Kugler J; Gellrich, Nils-Clauidus; Schimming, Ronald; Schramm, Alexander; Schmalohr,	2002	Journal of Oral & Maxillofacial Surgery (02782391)

		Dorothea; Bremerich, Andreas; Kugler, Joachim		
403.	The uvulopalatal flap for reconstruction of the soft palate.	Gillespie MB; Eisele DW	2000	Laryngoscope
404.	Assessment of fibula flap with flexor hallucis longus's effect on head & neck tumor patients' quality of life and function of donor site.	Ni, Youkang; Zhang, Xuedi; Zhang, Zhiqiang; Liang, Weidi; Zhao, Lina; Li, Zijia; Li, Siqi; Lu, Ping; Xu, Zhongfei; Dai, Wei; Duan, Weiyi; Tan, Xuexin; Sun, Changfu; Liu, Fayu	2020	Oral Oncology
405.	Chimeric Reverse Temporal Muscle and Pericranial Flap for Double-Layer Closure of Deep Facial Defects.	Algan, Said; Tan, Onder; Kara, Murat; Inaloz, Akin; Cakmak, Mehmet Akif; Aydin, Osman Enver	2018	Journal of Oral & Maxillofacial Surgery
406.	Cranial nerve outcomes in regionally recurrent head & neck melanoma after sentinel lymph node biopsy	Hanks, J.E.; Yalamanchi, P.; Kovatch, K.J.; Ali, S.A.; Smith, J.D.; Durham, A.B.; Bradford, C.R.; Malloy, K.M.; McLean, S.A.	2020	Laryngoscope
407.	Myxoma of the nasal bone.	Al-Qahtani, Khalid; Islam, Tahera; AlOulah, Mohammad; Bafaqeeh, Sameer; Faqeeh, Yasser	2017	Auris Nasus Larynx
408.	Long-term quality of life & functional outcomes after treatment of oropharyngeal cancer	Scott, S.I.; Kathrine Ã˜. Madsen, A.; Rubek, N.; Charabi, B.W.; Wessel, I.; Fredslund HadjÃº, S.; Jensen, C.V.; Stephen, S.; Patterson, J.M.; Friborg, J.; Hutcheson, K.A.; Kehlet, H.; von Buchwald, C.	2021	Cancer Medicine
409.	Masseter muscle flap for reconstruction of intra-oral defects in patients with early cancer of	Rajani, B C; Nadimul, Hoda; Subhabrata, Ghosh; Sabitha, K S; Vinitha, Annavarjula; Vasantha Dhara, B	2022	Brazilian journal of otorhinolaryngology

	posterior-inferior parts of the oral cavity.			
410.	The CO2 waveguide laser with flexible fiber in transoral resection of oral and oropharyngeal cancers: a retrospective cohort study on postoperative and quality of life outcomes	Gardenal, N.; Rigo, S.; Boscolo Nata, F.; FernÃ¡ndez-FernÃ¡ndez, M.M.; Boscolo-Rizzo, P.; Gatto, A.; Tirelli, G.	2022	Lasers in Medical Science
411.	Estimation of Temporomandibular Joint Dysfunction in Oral Cancer Survivors	Shinde S.; Kadam I.; Patil S.; Bhore P.; Gudur A.	2022	Asian Pacific journal of cancer prevention: APJCP
**Not possible to isolate the outcomes**				
412.	Functional evaluation of the spinal accessory nerve after neck dissection.	Leipzig, B; Suen, J Y; English, J L; Barnes, J; Hooper, M	1983	American journal of surgery
413.	Shoulder function after selective and superselective neck dissections: clinical and functional outcomes.	Giordano, L; Sarandria, D; Fabiano, B; Del Carro, U; Bussi, M	2012	Acta otorhinolaryngologica Italica
414.	Pain in the neck after neck dissection.	Talmi, Y P; Horowitz, Z; Pfeffer, M R; Stolik-Dollberg, O C; Shoshani, Y; Peleg, M; Kronenberg, J	2000	Otolaryngology head and neck surgery
415.	Transmandibular approaches to the oral cavity and oropharynx: A functional assessment	Christopoulos E.; Carrau R.; Segas J.; Johnson J.T.; Myers E.N.; Wagner R.L.	1992	Archives of Otolaryngology - Head and Neck Surgery
416.	Comparison of postoperative complications in advanced head and neck cancer patients receiving neoadjuvant chemotherapy followed by surgery versus surgery alone	Joshi P.; Joshi A.; Prabhash K.; Noronha V.; Chaturvedi P.	2015	Indian Journal of Medical and Paediatric Oncology

417.	Unilateral hyperslucent thorax on plain chest radiographs after neck dissection: Importance of atrophy of the trapezius muscle	Sugimoto, H.; Ohsawa, T.	1994	American Journal of Roentgenology
418.	Functional Involvement Of The Shoulder After Laryngectomy; A Lesion Of The Sterno-Clavicular Joint].	Chantraine, A; Sverdlik, S	1964	Revue du rhumatisme et des maladies osteo-articulaires
419.	Psychosocial considerations of the post-treatment of head and neck cancer patients.	Argerakis, G P	1990	Dental clinics of North America
420.	Free-flap reconstruction of the head and neck	Talmi, Y P; Gapany, M; Hoffman, H T	1992	Harefuah
421.	Application of stereotactic radiosurgery to the head and neck region.	Bajada, C; Selch, M; De Salles, A; Goetsch, S; Juillard, G; Solberg, T; Parker, R	1994	Acta neurochirurgica
422.	Quality of life after oral and oropharyngeal reconstruction with a radial forearm free flap: prospective study.	Bozec, Alexandre; Poissonnet, Gilles; Chamorey, Emmanuel; Casanova, Cedric; Laout, Claire; Vallicioni, Jacques; Demard, Francois; Peyrade, Frederic; Follana, Philippe; Bensadoun, Rene-Jean; Benezery, Karen; Thariat, Juliette; Marcy, Pierre-Yves; Sudaka, Anne; Weber, Patrice; Dassonville, Olivier	2009	Journal of otolaryngology
