# Supplementary material for: Musculoskeletal Impairments and Dysfunction in Individuals with Head and Neck Cancer Following Surgery with Neck Dissection—A Systematic Review
Source: Life (Basel). 2025 May 17;15(5):800. doi: 10.3390/life15050800 (PMC12112850; doi:10.3390/life15050800)
Supplement: Supplementary file 1 [file life-15-00800-s001.zip › Supplementary_Materials_C.pdf]

**SUPPLEMENTARY MATERIALS C:** The Summary of the included studies

| No | Study | Populations | Interventions | Other/comparators | Musculoskeletal Outcomes | Result | Conclusion |
|---|---|---|---|---|---|---|---|
| **1.** | **Authors:** Gallagher et al., 2015 [66]<br>**Title:** Association between multimodality neck treatment and work and leisure impairment: A disease-specific measure to assess both impairment and rehabilitation after neck dissection<br>**Country:** Canada<br>**Objective:** To explore the association between treatment outcome and shoulder-related on critical daily life functions such as employment and recreation.<br>**Study Design:** Cross-sectional.<br>**Groups:** 2 (SND vs MRND)<br>**Funding:** Unclear<br>**Setting:** Department of Otolaryngology– Head and Neck Surgery at the University of Michigan Health System | **Type of cancer:** Mixed HNC<br>**Stage:** Mixed stage (1-4)<br>**Age:** 57 (29-85) years.<br>**Gender:** M: 127 F: 40<br>**Duration post-surgery:** at least 12 months following neck dissection.<br>**Total sample size:** 167 | **Intervention 1:** Selective neck dissection (SND)<br>**Description:** Not reported.<br>**ND Surgery area:** Unclear.<br>**Reconstruction surgery:** Not reported.<br>**Other cancer treatments:** Radiotherapy or/and Chemotherapy<br>**Total sample:** 121<br>**Follow – up:** 1 (post-operative follow-up) | **Intervention 2:** Modified radical neck dissection (MRND)<br>**Description:** Not reported.<br>**ND surgery area:** Unclear.<br>**Reconstruction surgery:** Not reported.<br>**Other cancer treatments:** Radiotherapy or/and chemotherapy<br>**Total sample:** 46<br>**Follow – up:** 1 (post-operative follow-up) | **Outcome 1:** Neck and shoulder disability<br>**Outcome Tool:** Neck dissection impairment index (NDII)<br>**Outcome 2:** Shoulder disability<br>**Outcome Tool:** Constant Murley Score. | **Outcome 1:**<br>• Lower NDI scores were reported for patients who underwent MRND compared to patients who underwent SND (p =0.01).<br><br>**Outcome 2:** Not reported. | **Conclusion:**<br>• Shoulder impairment was significantly worse in patients who underwent MRND when compared to SND.<br>• Those who received radiation therapy or chemotherapy have been shown to have more significant shoulder disability. |
| **2.** | **Authors:** Wouwe et al., 2009 [72]<br>**Title:** Shoulder morbidity after non-surgical treatment of the neck<br>**Country:** Netherlands.<br>**Objective:** To determine the shoulder morbidity after | **Type of cancer:** Mixed HNC (oral cavity, oropharynx and larynx).<br>**Stage:** Mixed stage<br>**Age:** 60. 4 years (range 21–80 years).<br>**Gender:** M: 69 F: 31 | **Intervention 1:** Radical neck dissection (RND)<br>**Description:** Not reported.<br>**ND surgery area:** Unclear. | **Intervention 2:** Modified radical neck dissection (MRND).<br>**Description:** Not reported.<br>**ND surgery area:** Unclear. | **Outcome 1:** Shoulder Disability<br>**Outcome Tool:** Shoulder Disability questionnaire (SDQ)<br><br>**Outcome 2:** | **Outcome 1:**<br>• No difference in SDQ score was found between the different types of neck dissections. However, the score in SND is lower than MRND and RND. | **Conclusion:**<br>• The SDQ, stiffness, and pain scores were significantly higher in the surgical group than the non-surgical group ($p < 0.01$). |

| | | | | | | | |
|---|---|---|---|---|---|---|---|
| | non-surgical neck and neck dissection treatment.<br>**Study Design:** Cross-sectional.,<br>**Groups:** 3 (RND vs MRND vs SND)<br>**Funding:** Not reported<br>**Setting:** Unclear | **Duration post-surgery:** (Range 6–122 months)<br>**Total sample size:** 43 | **Reconstruction surgery:** Not reported.<br>**Other cancer treatments:** Radiotherapy and/or Chemotherapy<br>**Total sample:** 12<br>**Follow – up:** 1 (post-operative follow-up) | **Reconstruction surgery:** Not reported.<br>**Other cancer treatments:** Radiotherapy and/or chemotherapy<br>**Total sample:** 22<br>**Follow – up:** 1 (post-operative follow-up)<br><br>**Intervention 3:** Selective neck dissection (SND)<br>**Description:** Not reported.<br>**ND surgery area:** Unclear.<br>**Reconstruction surgery:** Not reported.<br>**Other cancer treatments:** Radiotherapy and/or chemotherapy<br>**Total sample:** 9<br>**Follow-up:** 1 (post operative follow-up) | Range of motion (ROM) - Shoulder (Flex and Abd)<br>**Outcome tool:** Inclinometer | • RND showed highest shoulder disability compared to the other two groups.<br><br>**Outcome 2:**<br>• The range of abduction and flexion of the shoulder was lower after RND than after MRND and SND. | • The RND group showed the highest shoulder dysfunction when compared to MRND and SND. |
| **3.** | **Authors:** Schiefke et al.,2009 [62]<br>**Title:** Function, postoperative morbidity, and quality of life after cervical sentinel node biopsy and after selective neck dissection.<br>**Country:** Germany | **Type of cancer:** Mixed HNC (Lip, floor of mouth, Palate, Buccal plane, Tongue, Tonsil, Hypopharynx )<br>**Stage:** mixed stage (I-IV)<br>**Age:** 32–89 | **Intervention 1:** Selective neck dissection (SND) levels I–III (supraomohyoid neck dissection).<br>**Description:** Unclear. | **Intervention 2:** SNB, sentinel node biopsy.<br>**Description:** Unclear.<br>**ND Surgery area:** SCC of the lips and the oral cavity | **Outcome 1:** Neck ROM<br>**Outcome tool:** Unclear.<br><br>**Outcome 2:** Constant Murley Score (CMS). | **Outcome 1:**<br>• SND and SNB had similar neck ROM in all directions.<br><br>**Outcome 2:**<br>• The Constant score of SNB patients (90. 3) was significantly higher | **Conclusion:**<br>• No significant differences exist in neck function (ROM) and CMS.<br>• However, the SNB group showed better function than SND. |

| | | | | | | | |
|---|---|---|---|---|---|---|---|
| | **Objective:** To compare postoperative morbidity and QOL between SNB and SND.<br>**Study Design:** Cross-sectional<br>**Groups:** 2<br>(Sentinel Biopsy (SNB) vs. SND levels I–III (supraomohyoid neck dissection))<br>**Funding:** Not reported<br>**Setting**: Department of Oral and Maxillofacial Surgery and Department of Otolaryngology of the University of Leipzig and Department of Otolaryngology, Charite, Humboldt University, Berlin | **Gender:** Mixed<br>M: 37; F: 12<br>**Duration post-surgery:** Not reported.<br>**Total sample size:** 49 | **ND Surgery Area:** levels I–III (supraomohyoid neck dissection).<br>**Reconstruction surgery:** Not reported.<br>**Other cancer treatments:** Alone<br>**Total Sample:** 25<br>**Follow-up:** 1 (post-operative follow-up) | **Reconstruction surgery:**<br>Not reported.<br>**Other cancer treatments:** Alone<br>**Total Sample:** 24<br>**Follow-up:** 1 (post-operative follow-up) | | (p=0.043) than in the SND group (82. 47) in patient symptom score and active shoulder function score.<br>• Patients treated by SNB received better scores in global shoulder active mobility. | |
| **4.** | **Authors:** Watkins et al., 2010 [63]<br>**Title:** Shoulder function in patients undergoing selective neck dissection with or without Radiation and chemotherapy<br>**Country:** USA<br>**Objective:** To compare patients who underwent selective neck dissection with those who underwent selective neck dissection plus radiotherapy or selective neck dissection plus chemoradiation therapy. | **Type of cancer:** Mixed HNC (upper aerodigestive tract carcinoma)<br>**Stage:** Not reported.<br>**Age mean:** 58<br>**Gender:** Mixed<br>M: 21F: 13<br>**Duration post-surgery:** 19–110 months<br>**Total sample size:** 34 | **Intervention 1:** Selective neck dissection (SND)<br>**Description:** Unclear.<br>**ND Surgery Area:** Unclear.<br>**Reconstruction surgery:** No<br>**Other cancer treatments:**<br>Alone<br>**Total sample:** 7<br>**Follow-up:** 1 (post-operative follow-up) | **Intervention 2:** selective neck dissection (SND) plus radiotherapy<br>**Description:** Unclear.<br>**ND Surgery area:** Unclear.<br>**Reconstruction surgery:**<br>No<br>**Other cancer treatments:**<br>radiotherapy<br>**Total sample:** 13<br>**Follow-up:** 1 (post-operative follow-up) | **Outcome 1:** Constant Murley Score (CMS). | **Outcome 1:**<br>• There was no significant difference (p =0. 16) in shoulder function when comparing total CMS scores among patients in the three treatment groups: SND (84±5), SND plus radiotherapy (71 ± 4), and SND plus chemoradiation therapy (77 ± 4). | **Conclusion:**<br>• SND can have a negative effect on shoulder function despite the preservation of the spinal accessory nerve.<br>• Radiotherapy or chemoradiation therapy added to selective neck dissection does not contribute additional detriment to shoulder function. |

| | | | | | | | |
|---|---|---|---|---|---|---|---|
| | **Study Design:** Cross-sectional<br>**Groups:** 3( SND alone, SND with Radiotherapy and SND with chemotherapy)<br>**Funding:** Not reported<br>**Setting**: Unclear | | | **Intervention 3:**<br>**Description:** Selective neck dissection With chemoradiation therapy:<br>**ND Surgery Area:** Unclear.<br>**Reconstruction surgery:** No<br>**Other cancer treatments:** Chemoradiation therapy:<br>**Total sample:** 14<br>**Follow-up:** 1 (post-operative follow-up) | | | |
| **5.** | **Authors:** Chepeha et al., 2002 [68].<br>**Title:** Functional assessment using constant´s shoulder scale after modified radical and selective neck dissection<br>**Country:** Canada<br>**Objective:** To determine which clinical and demographic determinants were important to long-term impairment.<br>**Study Design:** Cross-sectional<br>**Groups:** 2 (SND vs MRND)<br>**Funding:** Not reported<br>**Setting**: Outpatient clinic at the University of Michigan, | **Type of cancer:** Mixed HNC (Oral cavity, Oropharynx, Larynx/hypopharynx)<br>**Stage:** Mixed stage<br>**Age mean:** 56. 9-57. 6<br>**Gender:** Mixed. M:74% F:26%<br>**Duration post-surgery:** SND: 22. 4 months MRND: 42. 9 months<br>**Total sample size:** 64 | **Intervention 1:** Selective neck dissection (SND).<br>**Description:** All SND procedures were performed as described by Medina, and none included dissections of level V or excluded dissection of levels II and III.<br>**ND Surgery Area:** Unclear.<br>**Reconstruction surgery:** Not reported.<br>**Other cancer treatments:** | **Intervention 2:** Modified radical neck dissection (MRND)<br>**ND Surgery Area:** Unclear.<br>**Reconstruction surgery:** Not reported.<br>**Other cancer treatments:** Not reported.<br>**Total sample:** 32<br>**Follow-up:** 1 (post-operative follow-up) | **Outcome 1:** Constant Murley Score (CMS) | **Outcome 1:**<br>• The score showed MRND patients have lower scores on CMS when compared to SND: 80. 1; MRND: 62. 8 | **Conclusion:**<br>• The critical factors contributing to shoulder dysfunction after neck dissection were weight, radiation therapy, and neck dissection type. |

| | | | | | | | |
|---|---|---|---|---|---|---|---|
| | Department of Otolaryngology | | Not reported.<br>**Total sample:** 32<br>**Follow-up:** 1 (post-operative follow-up) | | | | |
| **6.** | **Authors:** Gane et al., 2018 [76]<br>**Title:** Neck and shoulder motor function following neck dissection: a comparison with healthy control subjects<br>**Country:** Australia<br>**Objective:** To compare the neck and shoulder motor function of patients following neck dissection, including comparison with a group of healthy volunteers.<br>**Study Design:** Cross-sectional<br>**Randomized Groups:** 3 (SND, MRND, Control)<br>**Funding:** Physiotherapy Research Foundation, Australia.<br>**Setting**: Two tertiary hospitals in Brisbane, Australia. | **Type of cancer:** Mixed HNC (Squamous cell, thyroid, melanoma, other)<br>**Stage:** mixed stage<br>**Age:** 51. 5<br>**Gender:** Mixed (ND group)<br>M: 39, F: 18<br><br>Healthy Controls:<br>M: 16, F: 18<br>**Duration post-surgery:** 0. 5-5 years post-surgery<br>**Total sample size:** 91 | **Intervention 1:**<br>Selective neck dissection (SND).<br>**Description:** Unclear.<br>**ND Surgery area:** Unclear.<br>**Reconstruction surgery:** Not reported.<br>**Other cancer treatments:**<br>Radiotherapy or/and Chemotherapy<br>**Total Sample:** n=37<br>**Follow-up:** 1 (post-operative follow-up) | **Intervention 2:**<br>Modified radical neck dissection (MRND)<br>**Description:** Unclear.<br>**ND Surgery Area:** Unclear.<br>**Reconstruction surgery:** Not reported.<br>**Other cancer treatments:**<br>Radiotherapy or/and Chemotherapy<br>**Total Sample:** 20<br><br>**Intervention 3:**<br>Control group (No surgery)<br>**Description:**<br>Healthy controls<br>**Total Sample:** 34 | **Outcome 1:**<br>ROM for neck and shoulder<br>**Outcome tool:**<br>Inclinometer<br><br>**Outcome 2:** Cervical and shoulder Muscle strength<br>**Outcome tool:**<br>Dynamometer | **Outcome 1:**<br>• In the neck dissection group, participants demonstrated significantly less flexion AROM (p = 0. 001) and abduction AROM (p =0. 0001) of the affected shoulder than the unaffected one.<br>• Healthy volunteers had significantly greater AROM in cervical extension and rotation to the affected and unaffected sides than those undergoing surgery.<br><br>**Outcome 2:**<br>• Healthy volunteers had significantly greater isometric strength of the shoulder flexors of the affected arm (women and men and cervical flexors (women and men) and a larger ratio of cervical flexion to extension strength (men only) | **Conclusion:**<br>• Muscular strength and AROM of the neck and shoulder are impaired in patients following neck dissection compared to healthy control subjects. |
| **7.** | **Authors:** Yu-Chi Huang et al., 2019 [75]. | **Type of cancer:** Mixed HNC (oral cavity, pharynx) | **Intervention 1:**<br>Selective neck dissection (SND) | **Intervention 2:**<br>Non-operated side | **Outcome 1:**<br>Pain<br>**Outcome tool:** | **Outcome 1:**<br>• The median VAS score of shoulder pain on the | **Conclusion:**<br>• Moderate shoulder pain and shoulder |

| | | | | | | | |
|---|---|---|---|---|---|---|---|
| | **Title:** The sonography and physical findings on shoulder after selective neck dissection in patients with head and neck cancer: a pilot study<br>**Country:** Taiwan<br>**Objective:** To investigate soft tissue disorders of affected shoulders after nerve-sparing selective neck dissection (SND) in patients with head and neck cancers (HNCs) by sonography.<br>**Study Design:** Cross-sectional<br>**Group:** 2 (SND (operated vs non-operated))<br>**Funding:** Chang Gung Memorial Hospital<br>**Setting**: Chang Gung Memorial Hospital | **Stage:** Unclear.<br>**Age:** 53<br>**Gender:** Mixed<br>M: 17, F: 1<br>**Duration post-surgery:**<br>Less than 6 months duration since nerve-sparing SND.<br>M: 2.6 months<br>**Total sample size:** 18 | **Description:** Unclear.<br>**ND Surgery Area:** Unclear.<br>**Reconstruction surgery:** Not reported.<br>**Other cancer treatments:** Radiotherapy<br>**Total Sample:** 18<br>**Follow-up:** 1 (post-operative follow-up) | | VAS<br><br>**Outcome 2:**<br>ROM for shoulder<br>**Outcome tool:**<br>Goniometer | surgical side was 4, snd non surgical Mdn = 0 , significantly higher than that on the nonsurgical side (p = 0.001).<br><br>**Outcome 2:**<br>• Lower shoulder flexion and abduction in operated shoulder | motion limitations occur in patients with HNC after SND. |
| **8.** | **Authors:** Scott et al., 2007 [71].<br>**Title:** The impact of selective neck dissection on shoulder and cervical spine movements<br>**Country:** United Kingdom<br>**Objective:** To investigate the deficits in function following a function-preserving neck dissection by comparing operative versus non-operative sides for cervical spine and shoulder movements in | **Type of cancer:** Mixed HNC, oral cavity, and oropharynx.<br>**Stage:** Advanced T stage 3-4 tumours<br>**Age:** 61<br>**Gender:** Mixed<br>M: 33; F: 30<br>**Duration post-surgery:** <1 month to 12 years postop.<br>**Total sample size:** 63 | **Intervention 1:**<br>Selective neck dissection (SND)<br>Operated side<br>**Description:**<br>Unilateral SND<br>**ND Surgery Area:** Unclear.<br>**Reconstruction surgery:** Not reported.<br>**Other cancer treatments:** Radiotherapy<br>**Total Sample:** n=63 | **Intervention 2:**<br>Non-operated side | **Outcome 1:** ROM for shoulder and neck<br>**Outcome tool:**<br>Goniometer (shoulder)<br>Tape measure (cervical)<br><br>**Outcome 2:**<br>Shoulder disability & quality of life.<br>**Outcome tool:**<br>University of Washington Quality-of-Life (UW-QOL)<br>NDII and SDQ | **Outcome 1:**<br>• Shoulder flexion and abduction results were significantly reduced on the operated shoulder compared to the non-surgical part. No difference in cervical ROM<br><br>**Outcome 2:**<br>• Cervical spine and shoulder movements on operated and non-operated sides were | **Conclusion:**<br>• Shoulder movements are limited on the operated vs. non-operated side in HNC patients who have received an SND. |

| | | | | | | | |
|---|---|---|---|---|---|---|---|
| | combination with patient self-completed questionnaires.<br>**Study Design:** Cross-sectional<br>**Group:** 2 (SND (operated vs non-operated))<br>**Funding:** Not reported<br>**Setting**: University Hospital Aintree | | **Follow-up:** 1 (post-operative follow-up) | | | correlated with the three patients' rated assessments (UW-QoL shoulder domain, SDQ, and NDII), except for shoulder elevation | |
| **9.** | **Authors:** Cho et al., 2015 [88].<br>**Title:** Measurement of the trapezius muscle volume: A new assessment strategy of shoulder dysfunction after neck dissection for the treatment of head and neck cancers<br>**Country:** Korea<br>**Objective:** to evaluate the influences of SAN injury during different types of neck dissection on postoperative shoulder dysfunction by measuring the trapezius muscle volume from a CT scan and from a questionnaire of shoulder disability.<br>**Study Design:** Retrospective cohort<br>**Groups:** 2 (RND vs Mixed ND)<br>**Funding:** Korea Health technology R&D Project, | **Type of cancer:** Mixed HNC (thyroid, tonsil, tongue, floor of the mouth, larynx, submandibular gland, parotid gland)<br>**Stage:** Not reported.<br>**Age:** 55. 57 (13. 17)<br>**Gender:** Mixed<br>M: 22; F: 20<br>**Duration post-surgery:** 12 months<br>**Total sample size:** 42 | **Intervention 1:** Radical neck dissection. (SAN SACRIFICE GROUP)<br><br>**Description:** Not reported.<br>**ND Surgery Area:** mixed<br>**Reconstruction surgery:** Not reported.<br>**Other cancer treatments:** Radiotherapy<br>**Total Sample:** 10<br>**Follow-up:** 2 (pre and post-operative follow-up) | **Intervention 2:** Mixed Neck Dissection SAN preservation group (MRND/SND)<br><br>**Description:** Not reported.<br>**ND Surgery Area:** mixed<br>**Reconstruction surgery:** Not reported.<br>**Other cancer treatments:** Radiotherapy<br>**Total Sample:** 32<br>MRND: 9<br>SND: 23 | **Outcome 1:** The volume of the trapezius muscle<br>**Outcome tool:** CT scan<br><br>**Outcome 2:** Shoulder disability<br>**Outcome tool:** Shoulder Disability Questionnaire (SDQ) | **Outcome 1:**<br>• The ratio of the trapezius muscle in the SAN preservation group (0. 91+-0. 14) was significantly higher than that of the RND group (0. 37+- 0. 18; p=0. 005).<br>• No difference between MRND and SND<br><br>**Outcome 2:**<br>• The SDQ score for the SAN preserving group (1. 8262. 96) was significantly lower (better) than the RND group (10. 90+-4. 75; p=0. 008) | **Conclusion:**<br>• RND patients have reduced trapezius muscle volume and also have a high disability in SDQ score when compared to MRND and SND. |

| | | | | | | | |
|---|---|---|---|---|---|---|---|
| | Ministry of Health and Welfare, Republic of Korea<br>**Setting**: Guru Hospital Korea University | | | | | | |
| **10.** | **Authors:** Jong-Lyel Roh et al.,2007 [84].<br>**Title:** Cervical sensory preservation during neck dissection<br>**Country:** Korea<br>**Objective:** compared the pain and motion of the neck and shoulder and the depression and QOL scores between groups undergoing CN XI-preserving neck dissections<br>**Study Design:** Retrospective cohort<br>**Randomized Groups:** 2 (SND and MND with cervical root branches preserved and SND and MND with cervical root branches removed)<br>**Funding:** Not reported<br>**Setting**: Unclear | **Type of cancer:** Mixed HNC (thyroid, larynx, oral cavity, Parotid/submandibular gland, hypopharynx.)<br>**Stage:** Mixed stage<br>**Age**: 27–78<br>**Gender:** Mixed M: 22 ; F: 31<br>**Duration post-surgery:** after 12-month surgery<br>**Total sample size:** 53 | **Intervention 1:** SND and MND with cervical root branches preserved<br>**Description:** During neck dissection, including level V, the CN XI and cervical sensory branches were identified and preserved after careful separation from the fibrofatty tissue of the posterior cervical triangle.<br>**ND Surgery Area:** Unclear.<br>**Reconstruction surgery:** No<br>**Other cancer treatments:** Radiotherapy<br>**Total Sample:** 24<br>**Follow-up:** 1 (post-operative follow-up) | **Intervention 2:** SND and MND with cervical root branches removed<br>**Description:** Unclear.<br>**ND Surgery Area:** Mixed (depending on the area of the tumour)<br>**Reconstruction surgery:** No/<br>**Other cancer treatments:** Radiotherapy<br>**Total Sample:** 29 | **Outcome 1:** Neck pain<br>**Outcome tool:** VAS<br><br>**Outcome 2:** Sensation<br>**Outcome tool:** Palpation/pinprick<br><br>**Outcome 3:** Myofascial pain<br>**Outcome tool:** VAS<br>**Outcome 4:** ROM for neck and shoulder<br>**Outcome tool:** Goniometer: shoulder inclinometer: neck<br><br>**Outcome 5:** Depression<br>**Outcome tool:** Beck Depression Inventory (BDI) | **Outcome 1:**<br>• The incidence and severity of neck pain were higher in the nerve-removed group than in the nerve-preserved group ($p = 0.02$). 30 of the 53 eligible patients (57%) experienced neck pain.<br><br>**Outcome 2:**<br>• 11 (37%) of these patients had allodynia in the neck, and 21 (70%) had hyperpathia. The incidence of allodynia and hyperpathia were also higher in the nerve-removed group ($p < 0.05$)<br><br>**Outcome 3:**<br>• Myofascial pain was most frequently present on the operated side: levator scapulae (43%), trapezius (32%), and rhomboid (15%) muscles. Incidences of myofascial pain and joint pain were not statistically different between the groups.<br><br>**Outcome 4:** | **Conclusion:**<br>• The removal of cervical root branches resulted in increased neck and shoulder pain as well as loss of sensation on the dissected neck after neck dissection preserving CN XI, related to depression and poor QOL.<br><br>` |

| | | | | | | | |
|---|---|---|---|---|---|---|---|
| | | | | | | • The mean active range of motion of the cervical spine or shoulder was not statistically different between groups (p > 0.1)<br><br>**Outcome 5**<br>• Thirty patients (57%) were estimated to have depression, which was higher in the nerve-removed group compared to the nerve-preserved group (p = 0.045) | |
| **11.** | **Authors:** Simon D. Carr et al., 2009 [80].<br>**Title:** Upper limb dysfunction following selective neck dissection: a retrospective questionnaire study<br>**Country:** United Kingdom<br>**Objective:** To determine total upper limb function following selective neck dissection over a mean follow-up of 1.6 years<br><br>**Study Design:** Retrospective cohort<br>**Groups:** 3 (Supramohyoid neck dissection (SOND) vs extended SOND (adds level IV and level V vs lateral | **Type of cancer:** Mixed HNC (did not report the origin of HNC)<br>**Stage:** Not reported.<br>**Age:** 45–77<br>**Gender:** Mixed: M: 50; F: 15<br>**Duration post-surgery:** 1. 6 years (range, 0. 5–4 years)<br>**Total sample size:** 56 | **Intervention 1:** Selective neck dissection (SND) supraomohyoid neck dissection (SOND)<br>**Description:** lymph node levels I–III)<br>**ND Surgery Area:** mixed<br>**Reconstruction surgery:** No<br>**Other cancer treatments:** Not reported.<br>**Total Sample:** 42<br>**Follow-up:** 1 (post-operative follow-up) | **Intervention 2:** Selective neck dissection (SND) extended SOND,<br>**Description:** (adds level IV and level V superior to the inferior belly of the omohyoid).<br>**ND Surgery Area:** mixed<br>**Reconstruction surgery:** No<br>**Other cancer treatments:** Unclear.<br>**Total Sample:** 12<br>**Follow-up:** 1 (post-operative follow-up)<br><br>**Intervention 3:** Selective neck | **Outcome 1:** Shoulder disability<br><br>**Outcome tool:** Disability of the arm, shoulder and hand (DASH) | **Outcome 1:**<br>• There is no statistical difference between groups. | **Conclusion:**<br>• Each type of selective neck dissection showed mild shoulder disability using DASH. |

| | | | | | | | |
|---|---|---|---|---|---|---|---|
| | neck dissection (levels II II IV))<br>**Funding:** Not reported<br>**Setting:** At the study centre (a tertiary head and neck cancer unit) | | | dissection (SND) , Lateral neck dissection<br>**ND Surgery Area:** mixed<br>**Reconstruction surgery:** No<br>**Other cancer treatments:** Unclear.<br>**Total Sample:** 11<br>**Follow-up:** 1 (post-operative follow-up) | | | |
| **12.** | **Authors:** Karin Murer et al., 2011 [86].<br>**Title:** Comparison of morbidity between sentinel node biopsy and elective neck dissection for treatment of the no neck in patients with oral squamous cell carcinoma<br>**Country:** Switzerland<br>**Objective:** To compare the complication rate and postoperative morbidity with special emphasis on shoulder function between patients undergoing SNB and elective neck dissection<br>**Study Design:** Retrospective cohort<br>**Groups:** 2<br>(SNB (sentinel node biopsy) vs.END (elective neck dissection) ) | **Type of cancer:** Mixed Cancer (Oral and Oropharynx)<br>**Stage:** Mixed stage<br>**Age:** 64 years (range, 35. 4–90. 3 years)<br>**Gender:**<br>M: SNB:19 END:21<br>F: SNB:14 END:8<br>**Duration post-surgery:** follow-up time after surgery was 4. 6 years (range, 1–9. 3 years).<br>**Total sample size:** 62 | **Intervention 1:** SNB, sentinel node biopsy<br>**Description:** SNB is a minimally invasive procedure with the excision of a limited number of lymph nodes and reduced tissue Trauma.<br>**ND Surgery Area:** oral cavity and oropharynx<br>**Reconstruction surgery:** No<br>**Other cancer treatments:** Alone<br>**Total Sample:** 33<br>**Follow-up:** 1 (post-operative follow-up) | **Intervention 2:** Elective Neck Dissection (END)<br>**Description:** NR<br>**ND Surgery Area:** Oral cavity and oropharynx<br>**Other cancer treatments:** Alone<br>**Total Sample:** 29<br>**Follow-up:** 1 (post-operative follow-up) | **Outcome 1:** Neck and shoulder disability<br><br>**Outcome tool:** Neck dissection impairment index (NDII)<br>**Outcome 2:** Shoulder disability<br><br>**Outcome tool:** Constant Murley Score | **Outcome 1:**<br>• Almost all patients in the SNB group showed a normal NDII post-operatively.<br>• Postoperative NDII after elective neck dissection also revealed excellent results on average. SND:99. 7 (90–100) END 94. 3 (32. 5–100)<br><br>**Outcome 2:**<br>• After SNB, the shoulder function was significantly better than patients after elective neck dissection (p= 0.018).<br>• However, with a mean score of 99. 87% (97. 3–100%) for the SNB group and 96. 13% (65. 3–100%) for the elective neck | **Conclusion:**<br>• They concluded that SNB is associated with a very low toll of complications and excellent preservation of shoulder function.<br>• Regarding the rate of postoperative complications and shoulder function, SNB is significantly superior to elective neck dissection.<br>• These results strongly support the idea that patients with nodal negative early SCC of the oral cavity should be offered SNB. |

| | | | | | | | |
|---|---|---|---|---|---|---|---|
| | **Funding:** Not reported<br>**Setting:** Department of Otolaryngology of the University Hospital of Zurich, Switzerland | | | | | dissection group, respectively, both groups achieved excellent results. | |
| **13.** | **Authors:** Li Y et al., 2013 [87].<br>**Title:** Evaluation of the efficacy of a novel radical neck dissection preserving the external jugular vein, greater auricular nerve, and deep branches of the cervical nerve<br>**Country:** China<br>**Objective:** Evaluated the efficacy, safety, and complication of NRND.<br>**Study Design:** Retrospective cohort<br>**Groups:** 2 (RDN vs NRDN)<br>**Funding:** Chongqing Medical University, the Natural Science Foundation Project of CQ CSTC and the Chongqing Municipal Health Bureau<br>**Setting:** Chongqing Medical University | **Type of cancer:** Oral cancer (Tongue, Floor of mouth, buccal cancer, lower gum cancer)<br>**Stage:** III: 27 (27%); IV: 73 (73%)<br>**Age:** 51. 1 (31 - 60), mean 52 years<br>**Gender:** Mixed<br>M: 61; F: 39<br>**Duration post-surgery:** 6 months post-surgery<br>**Total sample size:** 100 | **Intervention 1:** Radical neck dissection (RND)<br>**Description:** One group of 48 patients was treated with conventional RND, with dissection of the internal jugular vein, greater auricular nerve, and accessory nerve<br>**ND Surgery area:** Mixed<br>**Reconstruction surgery:** Not reported.<br>**Other cancer treatments:** Alone<br>**Total Sample:** 48<br>**Follow-up:** 1 (6-month post-operative follow-up) | **Intervention 2:** Novel RND (NRND)<br>**Description:** The other group of 52 patients was treated with NRND, with preservation of the external jugular vein, greater auricular nerve, and deep branches of the cervical nerve. A rectangular incision was made to conventional RND.<br>**ND Surgery Area:** Mixed<br>**Reconstruction surgery:** Not reported.<br>**Other cancer treatments:** Alone<br>**Total Sample:** 52<br>**Follow-up:** 1 (6-month post-operative follow-up) | **Outcome 1:** Shoulder disability<br>**Outcome tool:** Clinical assessment of pain and ROM<br><br>**Outcome 2:** Function<br>**Outcome tool:** Sensitivity test<br><br>**Outcome 3:** Facial and intracranial venous reflux<br>**Outcome tool:** Clinical assessment | **Outcome 1:**<br>• The six-month post-surgery follow-up showed that the NRND patients had significantly better shoulder function than the RND patients.<br><br>**Outcome 2:**<br>• The RND patients often reported auricular and posterior ear skin numbness or hypoesthesia, while the NRND patients usually reported normal feeling<br><br>**Outcome 3:**<br>• All patients had Phase I incision healing. | **Conclusion:**<br>• The NRND, preserving the external jugular vein, greater auricular nerve, and deep branches of the cervical nerve, has overcome the limitations of conventional and previously improved RND procedures.<br>• With the radical degree unaffected, the NRND diminished the postsurgical complications of intracranial hypertension, shoulder dysfunction, and auricular numbness. |
| **14.** | **Authors:** Terrel et al., 2000 [81]<br>**Title:** Pain, quality of life, and spinal accessory nerve status after neck dissection<br>**Country:** USA | **Type of cancer:** Mixed HNC (larynx, oral cavity, or oropharynx)<br>**Stage:** mixed stage I, II, III, IV<br>**Age:** 61.4 years | **Intervention 1:** MRND: dissections in which the spinal accessory nerve (CN XI) was resected | **Intervention 2:** SND: Neck dissection spared CN XI.<br>**Description:** Not described | **Outcome 1:** Pain.<br>**Outcome tool:** HRQOL.<br><br>**Outcome 2:** | **Outcome 1:**<br>• Patients who had resecting level V have greater shoulder and neck pain compared to | **Conclusion:**<br>• Modified neck dissections and selective neck dissections may be preferable over |

| | | | | | | | |
|---|---|---|---|---|---|---|---|
| | **Objective:** To assess QOL in patients with head and neck cancer who underwent neck dissection, and to compare QOL scores for patients in whom the spinal accessory nerve (CN XI) was resected or preserved.<br>**Study Design:** Retrospective cohort<br>**Groups:** 3<br>(Resecting CN XI vs Sparing CN XI; Resecting Level V vs Sparing Level V; Radical Neck dissection vs Modified Radical neck dissection sparing CN XI)<br>**Funding:** Not reported<br>**Setting:** Outpatient clinic (University of Michigan, the Ann Arbor Veterans Affairs Hospital, and the Cleveland Clinic Foundation) | **Gender:** Mixed (unclear the number of male and female patients)<br>**Duration post-surgery:** NR<br>**Total sample size:** 236 | **Description:** Not describe<br>**ND Surgery area:** mixed<br>**Reconstruction surgery:** NR<br>**Other cancer treatments:** Radiotherapy and/or chemotherapy<br>**Total Sample:** 46<br>**Follow-up:** Unclear | **ND Surgery Area:** Mixed<br>**Reconstruction surgery:** NR<br>**Other cancer treatments:** Radiotherapy and/or chemotherapy<br>**Total Sample:** 129<br>**Follow-up:** Unclear<br><br>**Intervention 3:** Radical neck dissection (RND)dissection who had the posterior triangle (level V) dissected<br>**ND Surgery area:** mixed<br>**Reconstruction surgery:** NR<br>**Other cancer treatments:** Radiotherapy and/or chemotherapy<br>**Total Sample:** 61<br>**Follow-up:** Unclear | Quality of life.<br>**Outcome tool:** SF 12 | those who are spared level V(p=0.006)<br><br>**Outcome 2:**<br>• Unclear. | traditional radical neck dissection because these less radical operations are associated with better pain-related QOL and less shoulder or neck pain. |
| **15.** | **Authors:** Dijkstra et al., 2001 [69].<br>**Title:** Incidence of shoulder pain after neck dissection: a clinical explorative study for risk factors<br>**Country:** Netherlands | **Type of cancer:** Mixed HNC (Squamous cell carcinoma; salivary gland tumor, melanoma, and others)<br>**Stage:** Mixed stage | **Intervention 1:** RND<br>**Description:** Not reported<br>**ND Surgery area:** Mixed<br>**Reconstruction surgery:** with | **Intervention 1:** MRND (preserving N XI)<br>**Description:** Not reported<br>**ND Surgery area:** Mixed | **Outcome 1:** Pain<br>**Outcome tool:** VAS<br><br>**Outcome 2:** Shoulder disability | **Outcome 1:**<br>• Patients who underwent RND have higher shoulder pain compared to MRND and SND.<br><br>**Outcome 2:** unclear | **Conclusion:**<br>• Pain after neck dissection is clinically present in 70% of the patients.<br>• A risk factor for the development of |

| | | | | | | | |
|---|---|---|---|---|---|---|---|
| | **Objective:** To determine the incidence of shoulder pain and restricted range of motion of the shoulder after neck dissection the day before discharge from the hospital, to analyze the effect of shoulder pain on daily activities in the clinical phase and to identify risk factors for the development of shoulder pain and restricted range of motion of the shoulder.<br>**Study Design:** Cross-sectional<br>**Groups:** 3 (RND vs MRND vs SND)<br>**Funding:** Not reported<br>**Setting:** Multicentre study in seven Dutch hospitals | **Age:** 60. 3 (12)<br>**Gender:** Mixed<br>M: 103: F: 68<br>**Duration post-surgery:** days: 13. 2 (10)<br>**Total sample size:** 172 | pectoral cutaneous flap; radial cutaneous flap.<br>**Other cancer treatments:** Radiotherapy or/and chemotherapy<br>**Total Sample:** 45<br>**Follow-up:** 1 (post-operative follow-up) | **Reconstruction surgery:** Unclear<br>**Other cancer treatments:** Radiotherapy or/and chemotherapy<br>**Total Sample:** 95<br>**Follow-up:** 1 (post-operative follow-up)<br><br>**Intervention 1:** SND<br>**Description:** Not reported<br>**ND Surgery area:** Mixed<br>**Reconstruction surgery:** Unclear<br>**Other cancer treatments:** Radiotherapy or/and chemotherapy<br>**Total Sample:** 32<br>**Follow-up:** 1 (post-operative follow-up) | **Outcome tool:** Clinical questions<br><br>**Outcome 3:** ROM - shoulder<br>**Outcome tool:** Inclinometer | **Outcome 3:**<br>• Patients in the RND group showed less forward flexion, abduction, and external shoulder rotation.<br>• Patients with MRND and SND have better movement than the RND group. | shoulder pain is a nonselective dissection.<br>• The pain has a considerable impact on clinical daily living activities. |
| **16.** | **Authors:** Cappiello et al., 2005 [82]<br>**Title:** Shoulder disability after different selective neck dissections (levels II-IV versus levels II-V): A comparative study<br>**Country:** Italy<br>**Objective:** To compare the results of clinical and electrophysiological investigations of shoulder function in patients affected | **Type of cancer:** Larynx or laryngeal cancer oropharynx<br>**Age:** SND (level II - IV) = 62 and SND (level V) = 61<br>**Gender:** Mixed (did not report the number)<br>**Duration post-surgery:** NR<br>**Total sample size:** 40 | **Intervention 1:** Selective neck dissection (SND)SND level II-IV.<br>**Description:** Patients in this group had undergone unilateral SND, including lymph nodes from levels II to IV.<br>**ND Surgery Area:** Not reported. | **Intervention 2:** Selective neck dissection (SND) SND level V.<br>**Description:** Unilateral SND from levels II to V. In all cases where the neck dissection was extended to level V, the C3-4 rami of the cervical plexus was preserved. | **Outcome 1:** Muscle strength - Shoulder<br>**Outcome tool:** Manual resistance<br><br>**Outcome 2:** ROM - Shoulder<br>**Outcome tool:** Goniometer<br><br>**Outcome 3:** Muscle activation | **Outcome 1:**<br>• None of the 20 patients reported any alteration in muscle strength.<br><br>**Outcome 2:**<br>• Ninety-five percent of upper limb abduction test results were normal in group 1, with slight impairment in only one patient (5%), as compared with only 75% | **Conclusion:**<br>• The study data confirm that clearance of the posterior triangle of the neck increases shoulder morbidity.<br>• However, subclinical nerve impairment can be observed even after selective neck dissection (levels II-IV) if the submuscular recess is dissected. |

| | | | | | | | |
|---|---|---|---|---|---|---|---|
| | by head and neck carcinoma treated with concomitant surgery on the primary and the neck with different selective neck dissections.<br>**Study Design:** Retrospective cohort<br>**Groups:** 2 (SND (level II - IV) and SND (level V))<br>**Funding:** Not reported<br>**Setting:** Department of Otolaryngology, University of Brescia (Brescia, Italy) | | **Reconstruction surgery:** NR.<br>**Other cancer treatments:** Unclear<br>**Total Sample:** 20<br>**Follow-up:** Not reported | **ND Surgery Area:** Not reported.<br>**Reconstruction surgery:** NR.<br>**Other cancer treatments:** Unclear<br>**Total Sample:** 20<br>**Follow-up:** Not reported | **Outcome tool:** EMG activity | of normal results of such a test in group 2<br><br>**Outcome 3:**<br>• Electromyographic abnormalities were less frequent in group 1 than in group 2, with a statistically significant difference (p=0. 003).<br>• The distribution of abnormalities recorded in the SCM muscle was 40% and 45% in the two groups, whereas the distribution of UT muscle alterations was 20% and 85%, respectively. | |
| **17.** | **Authors:** Cuccia et al., 2006 [83].<br><br>**Title:** Evidence of significant sternocleidomastoid atrophy following modified radical neck dissection type III<br>**Country:** Ireland<br>**Objective:** To assess retrospectively the local morphological changes in the sternocleidomastoid muscle in terms of local cross-sectional dimensional area (LCSD) and contractility following modified radical neck dissection type III. | **Type of cancer:** General HNC (Did not report specific type of HNC)<br>**Stage:** Not reported.<br>**Age:** NR<br>**Gender:** Not reported.<br>**Duration post-surgery:** 22.3<br>**Total sample size:** 45 | **Intervention 1:** Modified radical neck dissection (MRND)-unilateral type III<br>**Description:** Unclear<br>**ND Surgery Area:** Not reported.<br>**Reconstruction surgery:** NR.<br>**Other cancer treatments:** Alone.<br>**Total Sample:** 45<br>**Follow-up:** 1 (post-operative follow-up) | | **Outcome 1:** Muscle volume<br><br>**Outcome tool:** Ultrasound | **Outcome 1:**<br>• Type III modified radical neck dissection causes atrophy of the sternocleidomastoid muscle | **Conclusion:**<br>• This study shows that type III modified radical neck dissection causes atrophy of the sternocleidomastoid muscle.<br>• The atrophy is most marked in the middle and distal parts of the muscle. |

| | | | | | | | |
|---|---|---|---|---|---|---|---|
| | **Study Design:**<br>Retrospective cohort<br>**Groups:** 1(MRND)<br>**Funding:** Not reported<br>**Setting:** Not reported | | | | | | |
| **18.** | **Authors:** C. Carenfelt et al., 1981 [67].<br>**Title:** Radical Neck Dissection and Permanent Sequelae Associated with Spinal Accessory Nerve Injuries<br>**Country:** Sweden<br>**Objective:** Shoulder pain and physical and social disability following radical neck dissection have been evaluated in patients with sacrificed spinal accessory nerves and in patients with preserved nerves.<br>**Study Design:** Cross-sectional<br>**Groups:** 2 (Sacrificed SAN vs. Preserved SAN)<br>**Funding:** Not reported<br>**Setting:** Department of Physical Therapy | **Type of cancer:** Mixed HNC (unclear type of cancer)<br>**Stage:** Not reported.<br>**Age:** 53<br>**Gender:** Mixed<br>M: 37; F: 16<br>**Duration post-surgery:** 2-7 years post-op<br>**Total sample size:** 53 | **Intervention 1:**<br>Radical neck dissection (RND)Sacrificed SAN.<br>**Description:** SAN was sacrificed when a tumor involved the nerve or suspicious nodes were discovered adjacent to the nerve.<br>**ND Surgery Area:** Unclear.<br>**Reconstruction surgery:** Not reported<br>**Other cancer treatments:**<br>Radiotherapy<br>**Total Sample:** 18<br>**Follow-up:** 1 (post-operative follow-up) | **Intervention 2:**<br>Radical neck dissection (RND)<br>Preserved SAN<br>**ND Surgery Area:** Unclear.<br>**Reconstruction surgery:** Not reported<br>**Other cancer treatments:**<br>Radiotherapy<br>**Total Sample:** 35 | **Outcome 1:**<br>ROM – Shoulder<br>**Outcome tool:**<br>Goniometer<br><br>**Outcome 2:**<br>Muscle strength - Shoulder Abduction<br>**Outcome tool:**<br>Unclear | **Outcome 1& 2:**<br>• The ROM of shoulder abduction and the strength of abduction were significantly reduced in the sacrificed group.<br>• Eighteen patients in the sacrificed group had major paresis, whereas only six patients in the preserved group had major paresis. | **Conclusion:**<br>• The range of active shoulder abduction and the strength of abduction were significantly reduced in the sacrificed SAN group. |
| **19.** | **Authors:** McDonald et al., 2019 [89]<br>**Title:** Health-related quality of life in patients with T1N0 oral squamous cell carcinoma: selective neck dissection compared with wait and watch surveillance.<br>**Country:** United Kingdom | **Type of cancer:** Mixed HNC (Tongue, floor of mouth, Other)<br>**Stage:** I<br>**Age:** 61 (but not clearly reported)<br>**Gender:** Mixed<br>M: 72; F: 54 | **Intervention 1:**<br>Selective neck dissection (SND)<br>**Description:**<br>Not reported.<br>**ND Surgery Area:**<br>Mixed (oral)<br>**Reconstruction surgery:** | **Intervention 2:** No intervention<br>**Description:**<br>Not reported.<br>**ND Surgery Area:**<br>No surgery in the wait and watch group. | **Outcome 1:**<br>Quality of life<br>**Outcome tool:**<br>University of Washington Quality-of-Life (UW-QOL) | **Outcome 1:**<br>• Though the differences were insignificant, there was a worse HRQoL with SND than with wait-and-watch surveillance. | **Conclusion:**<br>• Worse HRQoL with SND than wait-and-watch surveillance, though the differences were insignificant. |

| | | | | | | | |
|---|---|---|---|---|---|---|---|
| | **Objective:** The aim of this study was to report the health-related quality of life in a consecutive group of patients with stage I disease at a time closest to 2 years after primary surgery.<br>**Study Design:** Retrospective cohort<br>**Randomized Groups:** 2 (Neck Dissection vs. Wait and Watch)<br>**Funding:** Not reported<br>**Setting:** Aintree University Hospital | **Duration post-surgery:** 2 years post-surgery<br>**Total sample size:** 126 | Other.<br>**Other cancer treatments:** Combined Free flap and/or RT in some pts.<br>**Total sample:** 37<br>**Follow-up:** 1 (post-operative follow-up) | **Reconstruction surgery:** Other<br>**Other cancer treatments:** Alone.<br>**Total sample:** 89 | | | |
| **20.** | **Authors:** Teymoortash et al., 2010 [85].<br>**Title:** Postoperative morbidity after different types of selective neck dissection<br>**Country:** Germany<br>**Objective:** To evaluate the type and frequency of complications after Selective neck dissection (SND).<br>**Study Design:** Retrospective cohort<br>**Randomized Groups:** 2 (SND with radiotherapy vs. without Radiotherapy)<br>**Funding:** Not reported<br>**Setting:** Department of Otolaryngology, Head and Neck Surgery, Philipp University, Marburg, Germany | **Type of cancer:** Mixed HNC carcinoma of the head<br>**Stage:** Mixed stage metastatic and non-metastatic<br>**Age:** Mean: 62 years.<br>**Gender:** Mixed M: 59 F: 39<br>**Duration post-surgery:** 0. 5–9. 1 year<br>**Total sample size:** 98 | **Intervention 1:** Selective neck dissection (SND) with radiotherapy<br>**Description:** Unclear<br>**ND Surgery Area:** Not reported.<br>**Reconstruction surgery:** Not reported.<br>**Other cancer treatments:** Radiotherapy.<br>**Total sample:** 37<br>**Follow-up**: 1 (post-operative follow-up) | **Intervention 2:** SND without radiotherapy<br>**Description:** Unclear<br>**ND Surgery Area:** Not reported.<br>**Reconstruction surgery:** Not reported.<br>**Other cancer treatments:** No<br>**Total sample:** 51<br>**Follow-up**: 1 (post-operative follow-up) | **Outcome 1:** Pain<br>**Outcome tool:** Visual rating scale<br><br>**Outcome 2:** ROM - shoulder<br>**Outcome tool:** Goniometer | **Outcome 1:**<br>• Forty-nine (94. 2%) patients did not have any persistent pain in the neck and shoulder area. Three (5. 8%) patients reported pain on a 2, 3, and 5 scale. All of those patients had received radiotherapy.<br><br>**Outcome 2:**<br>• Regarding shoulder and arm function, 31 (81. 6%) of all examined patients showed no side differences in active arm abduction. An impairment of active arm abduction up to 20 on the operated side. | **Conclusion:**<br>• The surgical extension of different kinds of SND seems to be correlated with higher postoperative morbidity compared to SNB in patients. |

| | | | | | | | |
|---|---|---|---|---|---|---|---|
| **21.** | **Authors:** Van Wilgen et al., 2003[79] .<br>**Title:** Shoulder pain and disability in daily life, following supraomohyoid neck dissection: a pilot study<br>**Country:** Netherlands<br>**Objective:** The purpose of this pilot study was to analyse the incidence of shoulder pain and disability following supraomohyoid neck dissection, and to determine which daily activities were disturbed.<br>**Study Design:** Retrospective cohort<br>**Randomized Groups:** 1 (SOND)<br>**Funding:** Not reported<br>**Setting:** Department of Oral and Maxillofacial Surgery (University Hospital Groningen) | **Type of cancer:** Mixed HNC (oral cavity or oropharynx)<br>**Stage:** Not reported.<br>**Age:** Mean: 63 years.<br>**Gender:** Mixed<br>F: 27, M: 23<br>**Duration post-surgery:** 2. 3 years<br>**Total sample size:** 50 | **Intervention 1:** Supraomohyoid neck dissection (SOND)<br>**Description:** NR<br>**ND Surgery Area:** Neck<br>**Reconstruction surgery:** Not reported.<br>**Other cancer treatments:** Combined Neck dissection and radiotherapy<br>**Total sample:** 50<br>**Follow-up**: 1 (post-operative follow-up) | | **Outcome 1:** Pain<br>**Outcome tool:** Not reported.<br><br>**Outcome 2:** Shoulder disability<br>**Outcome tool:** Shoulder Disability questionnaire (SDQ), and Groningen activity restriction scale (GARS) | **Outcome 1:**<br>• Fourteen patients (28%) perceived shoulder pain, of which four experienced this constantly, three often, and seven experienced shoulder pain occasionally. Twenty-seven patients had received radiotherapy, of which 10 (37%) complained of shoulder pain.<br><br>**Outcome 2:**<br>• The correlation between the frequency of shoulder pain and the amount of perceived disability during daily activities was r = 0:89. | **Conclusion:**<br>• Although the supraomohyoid neck dissection was developed to reduce shoulder morbidity, 28% of the patients in this study experienced shoulder pain and disability in daily activities one year or more post-operatively. |
| **22.** | **Authors:** Van Wilgen et al., 2004 [78]<br>**Title:** Shoulder complaints after nerve sparing neck dissections<br>**Country:** Netherlands<br>**Objective:** The aim of this study was to analyze shoulder complaints after nerve-sparing neck dissection and its impact on daily activities, and to | **Type of cancer:** Mixed HNC (Oral, Oropharynx, Larynx, Thyroid)<br>**Stage:** Not reported.<br>**Age:** 61 years (SD: 12. 1)<br>**Gender:** Mixed<br>M:89, F: 48<br>**Duration Post Surgery:** With | **Intervention 1:** Modified radical neck dissection (MRND)<br>**Description:** Not reported.<br>**ND Surgery Area:** Mixed<br>**Reconstruction surgery:** Yes<br>**Other cancer treatments:** Radiotherapy | **Intervention 2:** Postero-lateral neck dissection (PLND)<br>**Description:** Not reported.<br>**ND Surgery Area:** Mixed<br>**Reconstruction surgery:** Yes<br>**Other cancer treatments:** | **Outcome 1:** Shoulder disability<br>**Outcome tool:** Shoulder disability questionnaire (SDQ) | **Outcome 1:**<br>• The mean scores of the SDQ were: 48. 6 (SD: 35. 1) for the PLND, 22. 2 (SD: 28. 6) for the MRND, and 11. 6 (SD: 26. 1) for the SOHND, p <0. 01<br>• The mean scores on the SDQ were significantly higher for the patients with radiation therapy than those without mean | **Conclusion:**<br>• The prevalence of shoulder complaints after SOHND is low and reduces disability in daily life compared to other neck dissection types.<br>• The prevalence of shoulder complaints and disability rates |

| | | | | | | | |
|---|---|---|---|---|---|---|---|
| | analyse the influence of radiation therapy on shoulder complaints.<br>**Study Design:** Retrospective cohort<br>**Randomized Groups:** 3 ( PLND, MRND, and SOHND)<br>**Funding:** Grant from the University Hospital Groningen<br>**Setting:** University Hospital in Groningen | shoulder complaint: Mean: 3. 2 years<br>No shoulder complaint: 2. 9 years.<br>**Total sample size:** 137 | **Total sample:** 51<br>**Follow-up**: 1 (post-operative follow-up) | Radiotherapy<br>**Total sample:** 21<br><br>**Intervention 3:** Supraomohyoid neck dissection (SOND)<br>**Description:** Not reported.<br>**ND Surgery Area:** Mixed<br>**Reconstruction surgery:** Yes<br>**Other cancer treatments:** Radiotherapy<br>**Total Sample:** 65<br>**Follow-up**: 1 (post-operative follow-up) | | difference 12. 2 (95% CI: 2. 1 to 22. 3).<br>• However, in the regression analyses, radiation therapy did not contribute significantly to the prediction of the SDQ score. | after PLND are high and need further investigation.<br>• Radiation therapy does not have a significant effect on shoulder complaints and disability. |
| **23.** | **Authors:** Ravindrasinh et al., 2015 [61].<br>**Title:** Minimally invasive supraomohyoid neck dissection by total endoscopic technique for oral squamous carcinoma<br>**Country:** India<br>**Objective:** To study the feasibility of a total endoscopic technique for selective neck dissection in oral cancers and to compare the technique with conventional open technique with a long cervical scar | **Type of cancer:** Oral squamous carcinoma<br>**Stage:** I<br>**Age:** Not reported.<br>**Gender:** Not reported.<br>**Duration Post Surgery:** MISOND = 53. 7 (29. 8); open SOND = 39. 4 (5) months<br>**Total sample size:** 57 | **Intervention 1:** Open supraomohyoid neck dissection (SOND)<br>**Description:** Not reported.<br>**ND Surgery Area:** Supraomohyoid<br>**Reconstruction surgery:** Not reported.<br>**Other cancer treatments:** Alone.<br>**Total sample:** 35<br>**Follow-up:** 1 (post-operative follow-up) | **Intervention 2:** Minimally invasive supraomohyoid neck dissection (MISOND).<br>**ND Surgery Area:** Supraomohyoid<br>**Reconstruction surgery:** Not reported.<br>**Other cancer treatments:** Alone.<br>**Total Sample:** 22<br>**Follow-up:** 1 (post-operative follow-up) | **Outcome 1:** Shoulder disability<br><br>**Outcome tool:** Shoulder pain and disability index (SPADI) | **Outcome 1:**<br>• The mean SPADI score assessed six weeks postoperatively for the MISOND group was 14. 35 ± 0. 71 %, which was significantly better than 44. 14 ± 1. 18 % for the open technique (p<0. 001). | **Conclusion:**<br>• MISOND is a feasible and safe procedure with immediate oncologic outcomes comparable with those of conventional open SOND and provides better cosmetic and functional outcomes. |

| | | | | | | | |
|---|---|---|---|---|---|---|---|
| | **Study Design:** Cross-sectional.<br>**Randomized Groups:** 2 (open SOND vs MISOND)<br>**Funding:** Not reported<br>**Setting:** Bharat Cancer Hospital and Research Institute | | | | | | |
| **24.** | **Authors:** Refos et al., 2016 [58].<br>**Title:** Shoulder morbidity after pectoralis major flap reconstruction<br>**Country:** Netherlands<br>**Objective:** To examine the additional shoulder morbidity because of PMPF by comparing shoulder function within patients with neck dissections on both sides and PMPF on one side, and shoulder function between patients who underwent PMPF and neck dissections and those who had neck dissection only.<br>**Study Design:** Cross-sectional<br>**Randomized Groups:** 3 ( Mixed Neck dissection + PMPF , Mixed Neck Dissection without PMPF)<br>**Funding:** Not reported<br>**Setting:** VU University Medical Center, Amsterdam | **Type of cancer:** Mixed HNC<br>**Stage:** Mixed stage<br>**Age:** Group 1 (PMPF): 64. 7 (14. 4); Group 2 (neck dissection bilateral): 67. 8 (13. 5); Group 3 (one side): 60. 9 (9. 3)<br><br>**Gender:**<br>Mixed<br>Group 1: M :8, F:1<br>Group 2: M :23 , F:3<br>Group 3: M :33 , F: 14<br>**Duration Post Surgery:**<br>Group 1= 14-95 months. Group 2 = 4-139 months<br>Group 3= 4-226 months<br>**Total sample size:** 82 | **Intervention 1:** Mixed Neck dissection + PMPF<br>**Description:** Group 1 = Patients with a similar type of neck dissection on both sides and on one side PMPF.<br>**ND Surgery Area:** Mixed<br>**Reconstruction surgery:** PMPF = 45.<br>**Other cancer treatments:** Physical therapy or/and radiotherapy<br>**Total sample:** 9<br>**Follow-up:** 1 (post-operative follow-up) | **Intervention 2:** Mixed Neck Dissection<br>**Description:** Group 2 = Patients in whom a neck dissection and PMPF harvest was performed on the same side while no or a different type of neck dissection was performed on the other side.<br>**ND Surgery Area:** Mixed<br>**Reconstruction surgery:** PMPF = 45<br>**Other cancer treatments:** Combined : RT or PT<br>**Total sample:** 26<br>**Follow-up:** 1 (post-operative follow-up)<br><br>**Intervention 3:** Mixed Neck Dissection without PMPF | **Outcome 1:** Shoulder disability<br>**Outcome tool:** Shoulder Disability Questionnaire (SDQ)<br><br>**Outcome 2:** Pain<br>**Outcome tool:** VAS<br><br>**Outcome 3:** Stiffness<br>**Outcome tool:** Questionnaires<br>**Description:** Patients were also asked if they had experienced stiffness of the shoulder during the previous week ("yes" or "no").<br><br>**Outcome 4:** ROM - shoulder<br>**Outcome tool:** Inclinometer | **Outcome 1:**<br>• Within group 1, no difference was found between PMPF and neck dissection side and neck dissection only side. Comparing groups 2 and 3 there was a trend (p =0.065) for higher prevalence of shoulder morbidity for PMPF and neck dissection sides than for neck.<br>**Outcome 2:**<br>• Patients had shoulder pain (VAS >0) in 37 of 111 (33%) evaluable treated sides. Within group 1 and between groups 2 and 3 no difference was found in the presence of pain. Within group 1, no difference in mean VAS score was found between PMPF and neck dissection. | **Conclusion:**<br>• Patients frequently have additional shoulder morbidity after PMPF harvest, particularly after SND. PMPF harvest adds to the impairment of abduction. |

| | | | | | | | |
|---|---|---|---|---|---|---|---|
| | | | | **Description:**<br>Group 3 = patients with only a neck dissection (on either one or both sides).<br>**Reconstruction surgery:**<br>PMPF<br>**Total sample:** 47<br>**Follow-up:** 1 (post-operative follow-up) | | **Outcome 3:**<br>• Patients had shoulder stiffness in 49 of 103 (48%) evaluable treated sides. Within group 1, no difference was found between PMPF and neck dissection side and neck dissection only side. Comparing groups 2 and 3, a significant (p = 0.002) higher prevalence of shoulder stiffness for neck dissection only sides was found than for PMPF and neck dissection sides.<br><br>**Outcome 4:**<br>• A significant (p =. 026) lower ROM of abduction was found for group 2 (PMPF and neck dissection sides) as compared to group 3 (neck dissection only sides).<br>• Shoulder droop was not significantly more present in patients of group 2 (PMPF and neck dissection sides) as compared to group 3 (neck dissection only sides), also not in patients with unilateral neck dissection. | |

| | | | | | | | | |
|---|---|---|---|---|---|---|---|---|
| **25.** | **Authors:** Gane et al., 2017 [60].<br>**Title:** Neck and Upper Limb Dysfunction in Patients following Neck Dissection: Looking beyond the Shoulder<br>**Country:** Australia<br>**Objective:** To evaluate the level of self-reported upper limb and neck function in patients following ND and explore any associations with patient or clinical factors.<br>**Study Design:** Cross-sectional<br>**Randomized Groups:** 3 (SND vs MRND vs Bilateral ND)<br>**Funding:** Physiotherapy Research Foundation<br>**Setting:** 2 tertiary hospitals in<br>Brisbane, Australia | **Type of cancer:** Mixed HNC (SCC, squamous cell carcinoma; PTC, papillary thyroid carcinoma; Melanoma; other)<br>**Stage:** Mixed stage<br>**Age:** 62 (51, 71) years<br>**Gender:** Mixed M:63, F: 26<br>**Duration Post Surgery:** 2. 98 (1. 71, 4. 18) years<br>**Total sample size:** 89 | **Intervention 1:** Selective neck dissection (SND)-Unilateral<br>**Description:** Not reported.<br>**ND Surgery Area:** Mixed<br>**Reconstruction surgery:** Not reported.<br>**Other cancer treatments:** Radiotherapy and/or Chemotherapy<br>**Total sample:** 57<br>**Follow-up:** 1 (post-operative follow-up) | **Intervention 2:** Modified radical neck dissection (MRND): Unilateral<br>**Description:** Not reported.<br>**ND Surgery Area:** Mixed<br>**Reconstruction surgery:** Not reported.<br>**Other cancer treatments:** Radiotherapy and/or Chemotherapy<br>**Total Sample:** 18<br>**Follow-up:** 1 (post-operative follow-up)<br><br>**Intervention 3:** Bilateral ND<br>**Description:** Not reported.<br>**ND Surgery Area:** Mixed<br>**Reconstruction surgery:** Not reported.<br>**Other cancer treatments:** Radiotherapy and/or Chemotherapy<br>**Total sample:** 14<br>**Follow-up**: 1 (post-operative follow-up) | **Outcome 1:** Shoulder disability<br>**Outcome tool:** Quick Disability of the Arm, Shoulder and Hand (Quick DASH)<br><br>**Outcome 2:** Neck disability<br>**Outcome tool:** Neck Disability Index (NDI)<br><br>**Outcome 3:** Pain<br>**Outcome tool:** VAS | **Outcome 1:**<br>• They did not present the between-group comparison, but the results are: Unilateral SND (n = 57): mean 17 (16); median 11 (5.28), Unilateral MRND (n = 18): mean 22 (30); Median 5 (0.34)<br><br>**Outcome 2:**<br>• They did not present the between-group comparison, but the results are: Unilateral SND: mean 16 (13); median 12 (4.26), Unilateral MRND: mean 21 (26); Median 6 (2.34)<br><br>**Outcome 3:**<br>• They did not present a group comparison, but the results are: Unilateral SND: mean 12 (16); median 3 (0.20); Bilateral ND: mean 25 (29); Median 16 (2.47)<br><br>**Outcome 4:**<br>• They did not present a between-group comparison, but the results are: Unilateral SND: mean 16 (18); median 11 (2.24) Bilateral | **Conclusion:**<br>• This study highlights the potential for neck and upper limb dysfunction in patients following ND. |

| | | | | | | | |
|---|---|---|---|---|---|---|---|
| | | | | | | ND: mean 22 (29); Median 9 (0.28) | |
| **26.** | **Authors:** Gane et al., 2017[74].<br>**Title:** Predictors of health-related quality of life in patients treated with neck dissection for head and neck cancer<br>**Country:** Australia<br>**Objective:** To examine the effect of clinical and treatment-related factors on HRQOL in patients who were 6 months to 5 years post neck dissection for HNC.<br>**Study Design:** Cross-sectional<br>**Randomized Groups:** 3 (Unilateral SND vs Unilateral MRND, Unilateral SND vs bilateral ND, Unilateral MRND vs Bilateral ND)<br>**Funding:** Physiotherapy Research Foundation<br>**Setting:** Two tertiaries hospitals in Brisbane, Australia | **Type of cancer:** Mixed HNC (SCC; PTC; Melanoma; Others)<br>**Stage:** Mixed stage (T-stage and N-stage)<br>**Age:** 61 (50, 70) years<br>**Gender:** Mixed M:91, F: 38<br>**Duration Post Surgery:** Days = 1089<br>**Total sample size:** 129 | **Intervention 1:** Selective neck dissection (SND): Unilateral SND<br>**Description:** Not reported.<br>**ND Surgery Area:** Mixed<br>**Reconstruction surgery:** Unclear.<br>**Other cancer treatments:** Radiotherapy and/or Chemotherapy<br>**Total sample:** 74 (57%).<br>**Follow-up:** 1 (post-operative follow-up) | **Intervention 2:** Modified radical neck dissection (MRND): Unilateral MRND<br>**Description:** Not reported.<br>**ND Surgery Area:** Mixed<br>**Reconstruction surgery:** Unclear.<br>**Other cancer treatments:** Combined Chemotherapy<br>**Total sample:** 31 (24%)<br>**Follow-up:** 1 (post-operative follow-up)<br><br>**Intervention 3:** Bilateral ND<br>**Description:** Not reported.<br>**ND Surgery Area:** Mixed<br>**Reconstruction surgery:** Unclear.<br>**Other cancer treatments:** Combined<br>**Total sample:** 24 (19%) | **Outcome 1:** Neck disability<br>**Outcome tool:** Neck dissection impairment index (NDII) | **Outcome 1:**<br>• They did not present between-group comparison, but the results are: Unilateral SND (n = 74): mean 75 (23); median 83 (48, 93), Unilateral MRND (n = 31): mean 66 (31); Median 73 (38, 98) . Indicates MRND had more neck disability compared to SND. | **Conclusion:**<br>• In conclusion, patients following neck dissection demonstrated reduced HRQOL when measured with NDII.<br>• More extensive neck dissection and adjuvant treatment were associated with worse HRQOL, confirming that more treatment (surgery or other modalities) leads to comparatively worse HRQOL. |

| | | | | | | | |
|---|---|---|---|---|---|---|---|
| | | | | **Follow-up**: 1 (post-operative follow-up) | | | |
| **27.** | **Authors:** Popovski et al., 2017 [59].<br>**Title:** Spinal accessory nerve preservation in modified neck dissections: surgical and functional outcomes<br>**Country:** Macedonia<br>**Objective:** To analyse the intra-operative variations of the spinal accessory nerve pathway and to evaluate shoulder dysfunction postoperatively.<br>**Study Design:** Cross-sectional<br>**Randomized Groups:** 2 (MRND and SND)<br>**Funding:** Not reported<br>**Setting:** Clinic for Maxillofacial Surgery in Skopje | **Type of cancer:** Mixed HNC<br>**Stage:** Not reported.<br>**Age:** Not reported.<br>**Gender:**<br>Not reported.<br>**Duration Post Surgery:** Five years<br>**Total sample size:** 60 | **Intervention 1:**<br>Modified radical neck dissection (type I, II, III) -MRND<br>**Description:**<br>Unclear<br>**ND Surgery Area:**<br>Posterior neck triangle<br>**Reconstruction surgery:**<br>Not reported.<br>**Other cancer treatments:**<br>Not reported.<br>**Total sample:** 20<br>**Follow-up:** 1 (post-operative follow-up) | **Intervention 2:**<br>Selective neck dissections - SND (supra-omohyoid, lateral, posterolateral, anterior compartment)<br>**Description:**<br>Unclear.<br>**ND Surgery Area:**<br>Posterior neck triangle<br>**Reconstruction surgery:**<br>Not reported.<br>**Other cancer treatments:** Not reported.<br>**Total sample:** 40<br>**Follow-up:** 1 (post-operative follow-up) | **Outcome 1:**<br>Shoulder disability<br>**Outcome tool:**<br>Shoulder Disability Questionnaire (SDQ)<br><br>**Outcome 2:**<br>ROM- Shoulder<br>**Outcome tool:**<br>Not reported.<br><br>**Outcome 3:**<br>Shoulder pain<br>**Outcome tool:**<br>VAS | **Outcome 1:**<br>• SDQ score was significantly lower in the spinal accessory nerve preservation group compared to the MRND group.<br><br>**Outcome 2:**<br>• Shoulder flexion and abduction on the operated with MRND were significantly lower than on the control sides.<br>**Outcome 3:**<br>• Visual Analog Scale (VAS) was also used, and 28% of the patients had no pain. | **Conclusion:**<br>• On average, neck dissection patients with the spinal accessory nerve preserved have less pain in their shoulders, less functional disability and more substantial results on physical examination than those with the spinal accessory nerve sacrificed without any difference in local control and survival. |
| **28.** | **Authors:** Eickmeyer et al., 2014 [64]<br>**Title:** Quality of Life, Shoulder Range of Motion, and Spinal Accessory Nerve Status in Five-Year Head and Neck Cancer Survivors<br>**Country:** USA<br>**Objective:** To determine the association of neck dissection and radiation treatment for head and neck cancer (HNC) with subsequent shoulder range | **Type of cancer:** Mixed HNC (Larynx, oral cavity, oropharynx, hypopharynx, other)<br>**Stage:** Not reported.<br>**Age:** 65.3<br>**Gender:** Mixed<br>M: 77; F: 28<br>**Duration Post Surgery:** At least five years post-treatment.<br>**Total sample size:** 105 | **Intervention 1:** SAN sacrificing<br>**Description:** Unclear<br>**ND Surgery Area:**<br>Mixed<br>**Reconstruction surgery:**<br>Not reported.<br>**Other cancer treatments:**<br>Radiotherapy<br>**Total sample:** 16<br>**Follow-up:** 1 (post-operative follow-up) | **Intervention 2:**<br>SAN sparing<br>**Description:** Unclear<br>**ND Surgery Area:**<br>Mixed<br>**Reconstruction surgery:**<br>Not reported.<br>**Other cancer treatments:**<br>Radiotherapy<br>**Total sample:** 33<br>**Follow-up:** 1 (post-operative follow-up) | **Outcome 1:**<br>Quality of life<br>**Outcome tool:**<br>University of Washington Quality-of-Life (UW-QOL)<br><br>**Outcome 2:**<br>ROM – Shoulder (Flexion, abduction, external and internal rotation).<br>**Outcome tool:**<br>Goniometer | **Outcome 1:**<br>• Impairment scores for UWQOL subjective disfigurement, level of activity, self-reported recreation/entertainment, speech, and shoulder disability were worst for the nerve sacrifice group.<br><br>**Outcome 2:**<br>• Flexion and abduction are worse for the nerve sacrifice group. | **Conclusion:**<br>• Deficits in QoL for 5-year survivors, with few having received appropriate rehab. Shoulder dysfunction is common among all subjects. Shoulder ROM and QoL were the best in the non-surgical group. |

| | | | | | | | |
|---|---|---|---|---|---|---|---|
| | of motion (ROM) and quality of life (QOL) in 5-year survivors.<br>**Study Design:** Cross-sectional<br>**Randomized Groups:** 3 (SAN sacrificing, SAN sparing and no surgery)<br>**Funding:** Not reported<br>**Setting:** Tertiary care hospital and from an affiliated Veterans Affairs medical centre. | | | **Intervention 3:** No surgery<br>**ND Surgery Area:** Mixed<br>**Reconstruction surgery:** Not reported.<br>**Other cancer treatments:** Radiotherapy<br>**Total sample:** 56<br>**Follow-up:** Post cancer treatment | | • Shoulder ROM is not correlated with the presence or absence of radiation.<br>• The type of dissection is associated with the ability to perform upper extremity ADLs in the nerve sacrifice group compared to nerve-sparing. | |
| **29.** | **Authors:** Ghiam et al., 2017 [65]<br>**Title:** Assessment of musculoskeletal impairment in head and neck cancer patients<br>**Country:** USA<br>**Objective:** To describe the types of musculoskeletal impairment in head and neck cancer survivors and to evaluate objective and subjective measures of musculoskeletal impairment and identify areas of need in future studies.<br>**Study Design:** Cross-sectional<br>**Randomized Groups:** 1 Mixed ND (RND and MRND)<br>**Funding:** Pain and Symptom Management Ingram Foundation Funds | **Type of cancer:** Mixed HNC (Paranasal sinuses, oral cavity, oropharynx, larynx)<br>**Stage:** Mixed stage<br>**Age:** 60. 4 years<br>**Gender:** Mixed<br>M: 19 (65. 5%)<br>F: 10 (34. 5%)<br>**Duration Post Surgery:** mean: 23. 9 months post-treatment<br>**Total sample size:** 28 | **Intervention 1:** Mixed ND (RND and MRND)<br>**ND Surgery Area:** Mixed<br>**Reconstruction surgery:** Flap reconstruction.<br>**Other cancer treatments:** Radiation, chemotherapy.<br>**Total sample:** 28 (RND = 11, MRND= 7<br>**Follow-up**: 1 (post-operative follow-up) | | **Outcome 1:** Neck disability<br>**Outcome tool:** Neck disability index (NDI)<br><br>**Outcome 2:** Shoulder pain and disability<br>**Outcome tool:** Shoulder pain and disability index (SPADI)<br><br>**Outcome 3:** Acute and late symptoms of HNC<br>**Outcome tool:** Vanderbilt Head and Neck Symptom Survey (VHNSS)<br><br>**Outcome 4:** ROM- Cervical<br>**Outcome tool:** | **Outcome 1:**<br>• The majority of the patients (69%, 20/29) suffered from some level of neck disability as described by the NDI. Approximately 10% (3/29) had a severe level of neck disability, while approximately 17% (5/29) had a moderate level of disability.<br><br>**Outcome 2:**<br>• Approximately 35% (10/29) and 20. 7% (6/29) reported high levels of shoulder pain and shoulder disability as determined by their SPADI scores. | **Conclusion:**<br>• Both subjective and objective evidence of musculoskeletal impairments in post-surgical HNC patients.<br>• Musculoskeletal impairment is a significant side effect in head and neck cancer survivors that results in chronic neck pain, shoulder disability, trismus, and postural deficits. |

| | | | | | | | |
|---|---|---|---|---|---|---|---|
| | **Setting:** Vanderbilt University Medical Center | | | | Dynatronics CROM<br><br>**Outcome 5:**<br>ROM- Mouth opening<br>**Outcome tool:**<br>OraStretch | **Outcome 3:**<br>• In terms of symptom clusters, patients reported the highest severity scores for trismus with a median score of 4. 0 out of 10 possible (IQR, 0–8), followed by xerostomia (median = 3. 8; IQR, 1–6), difficulty swallowing solids (median = 3. 6; IQR, 2–7), dysphonia (median = 3. 3; IQR, 1–5), and neck and shoulder impairment (median = 3. 0; IQR, 0–6).<br><br>**Outcome 4:**<br>• Statistically significant inverse associations with self-reports of neck/shoulder impairment (VHNSS) were observed for CROM extension (rs = −0. 46, p = 0. 012), left lateral flexion (rs = −0. 51, p = 0. 005), and right lateral rotation (rs = −0. 50, p = 0. 006).<br><br>**Outcome 5**<br>• The MIO for the patients averaged 33. 4 mm (SD = 11. 2). MIO values were inversely associated with | |

| | | | | | | | |
|---|---|---|---|---|---|---|---|
| | | | | | | self-reported jaw and trismus symptoms on the VHNSS (rs = −0. 58, p = 0. 00) | |
| **30.** | **Authors:** Inoue et al., 2006 [70].<br>**Title:** Quality of Life After Neck Dissection<br>**Country:** Japan<br>**Objective:** To assess the impact of modifications to radical neck dissection on postoperative quality of life.<br>**Study Design:** Cross-sectional<br>**Randomized Groups:** 5<br>No neck dissection (Control group) Level I-III nodes of extension of neck dissection and spinal accessory nerve preserved (Group III) Level II-IV nodes of extension of neck dissection and spinal accessory nerve preserved (Group IV) Level I-V nodes of extension of neck dissection and spinal accessory nerve preserved (Group V) Level I-V nodes of extension of neck dissection and spinal accessory nerve was resected (Group VI)<br>**Funding:** Ministry of Health, Labour and Welfare, Tokyo, Japan | **Type of cancer:** Mixed HNC (Oral cavity, Oropharynx, Larynx Hypopharynx, Other.<br>**Stage:** Metastatic and non-metastatic.<br>**Age:** Mean: 61 (39 to 84 years)<br>**Gender:** Not reported.<br>**Duration Post Surgery:** 36 months, (12 months to 23 years)<br>**Total sample size:** 74 | **Intervention 1:** Mixed Neck Dissection (with different level)<br>**Description:** In general, the cervical nerve, SCM, SAN, and IJV were preserved in necks in which level V nodes were not dissected. Level V nodes were dissected in 74 necks. Both the SCM and cervical nerve were resected in all necks in which level V nodes were dissected.<br>**ND Surgery Area:** Neck and nodes.<br>**Reconstruction surgery:** Not reported.<br>**Other cancer treatments:** Combined 2 neck dissection and radiotherapy.<br>**Total sample:** Unclear.<br>**Follow-up:** 1 (post-operative follow-up) | **Intervention 2:** No neck dissection (control) | **Outcome 1:**<br>ROM - Shoulder<br>**Outcome tool:**<br>Arm Abduction Test (AAT) | **Outcome 1:**<br>• The AAT scores decreased as the extent of dissection increased.<br>• The AAT scores for all four groups were significantly lower than for the control group.<br>• Among the four groups, the score for group VI was significantly lower than for the other three groups (p<0.001). | **Conclusion:**<br>• In terms of shoulder drop, the score in group IV was the same as that in group III and better than that in group V.<br>• Although not statistically significant, these findings indicate that preservation of the SCM and SAN may contribute to preventing shoulder drops. |

| | | | | | | | |
|---|---|---|---|---|---|---|---|
| | **Setting:** Department of Otolaryngology–Head and Neck Surgery, Kobe University Hospital | | | | | | |
| **31.** | **Authors:** Tarkan et al., 2012 [73].<br>**Title:** Clinical and electrophysiological evaluation of shoulder functions in spinal accessory nerve-preserving neck dissection<br>**Country:** Turkey<br>**Objective:** To evaluate SAN functions after neck dissection with preservation of the SAN by objective clinical physical examination, EMG or electroneurography (ENG) findings, and subjective patient complaints, as well as to investigate the effects of neck dissection type.<br>**Study Design:** Cross-sectional<br>**Randomized Groups:**1 (Mixed SND and MRND)<br>**Funding:** Not reported<br>**Setting:** Department of Neurology and Department of Physical Therapy and Rehabilitation | **Type of cancer:** Head and neck cancer and/or neck metastasis<br>**Stage:** Mixed stage<br>**Age:** M: 57. 6 (38-80 years)<br>**Gender:** Mixed<br>M: 22, F:7<br>**Duration Post Surgery:** More than 12 weeks<br>**Total sample size:** 29 | **Intervention 1:** Mixed Neck Dissection (Preserved SAN)<br>**Description:** 29 patients who underwent unilateral SND and MRND due to head and neck cancer and/or neck metastasis.<br>**ND Surgery Area:** Unclear.<br>**Reconstruction surgery:** Not reported.<br>**Other cancer treatments:** Alone.<br>**Total sample:** 29<br>**Follow-up**: 1 (post-operative follow-up) | | **Outcome 1:**<br>ROM- shoulder<br>**Outcome tool:**<br>Goniometer<br><br>**Outcome 2:**<br>Pain<br>**Outcome tool:**<br>VAS | **Outcome 1:**<br>• The shoulder joint's flexion, abduction, and external rotation were significantly affected on the operated side ($p < 0.05$).<br><br>**Outcome 2:**<br>• Mild or moderate pain was observed at the early stage (initial 12 months) with a visual pain scale. The only patient with very severe pain was in the early stages. | **Conclusion:**<br>• Despite the preservation of the SAN during neck dissection, changes and alterations in clinical functions might be seen in all areas of the nerve that innervate the shoulder muscles. |
| **32.** | **Authors:** Cheng et al., 2000 [46].<br>**Title:** Objective comparison of shoulder dysfunction | **Type of cancer:** Not reported.<br>**Stage:** Not reported. | **Intervention 1:**<br>Radical neck dissection (RND). | **Intervention 2:**<br>Modified radical neck dissection (MRND)<br>**Description:** | **Outcome 1:**<br>Muscle strength<br>**Outcome tool:**<br>Isokinetic | **Outcome 1:**<br>• SND: The peak torques of shoulder flexion-extension and abduction- | **Conclusion:**<br>• The SND group was found to have the least damage to the SAN, as |

| | | | | | | | |
|---|---|---|---|---|---|---|---|
| | after three neck dissection technique<br>**Country:** Taiwan<br>**Objective:** To measure shoulder disability and compare the differences in shoulder disability that occurred in patients who underwent different neck dissection procedures<br>**Study Design:** Prospective cohort<br>**Randomized Groups:** 3 (RND vs MRND vs SND)<br>**Funding:** Not reported<br>**Setting:** Unclear | **Age:** Mean: 50 (39 to 70 years)<br>**Gender:** Mixed<br>M: 18, F: 3<br>**Duration Post Surgery:** Not reported.<br>**Total sample size:** 21 | **Description:** Not reported.<br>**ND Surgery Area:** Unclear.<br>**Reconstruction surgery:** Not reported.<br>**Other cancer treatments:**<br>Not reported.<br>**Total sample:** 5<br>**Follow-up:** 3 (pre and post follow-up, 1 and 6 months) | Not reported.<br>**ND Surgery Area:** Unclear.<br>**Reconstruction surgery:**<br>Not reported.<br>**Other cancer treatments:** Not reported.<br>**Total sample:** 9<br>**Follow-up**: 3 (pre and post follow-up, 1 and 6 months)<br><br>**Intervention 3:** Selective neck dissection (SND)<br>**Description:**<br>Not reported.<br>**ND Surgery Area:**<br>Not reported.<br>**Reconstruction surgery:**<br>Not reported.<br>**Other cancer treatments:**<br>Not reported.<br>**Total sample:** 7<br>**Follow-up:** 3 (pre and post follow-up, 1 and 6 months) | **Outcome 2:** Muscle activation - upper trapezius<br>**Outcome tool:** EMG | adduction 1 month after operation were significantly lower than pre-operative measurement. However, 6 months after operation, the peak torque of the operated shoulder had returned to its preoperative value.<br>• MRND: The affected shoulder showed peak torques that were significantly lower 1 month after the operation than before the surgery. Six months after the operation, the peak torques of the affected shoulder were still lower than those seen before the operation. However, statistical significance was only seen in shoulder flexion-extension at 60°/s.<br>• RND: The peak torques of the operated shoulder in all directions were significantly lower 1 month and 6 months after operation compared with the preoperative peak torque ($p < 0.05$). | demonstrated by postoperative ENoG, EMG, and isokinetic evaluation.<br>• Preservation of the SAN, accompanied by rehabilitation, may lessen the development of shoulder disability after neck dissection. |

| | | | | | | | |
|---|---|---|---|---|---|---|---|
| | | | | | | **Outcome 2:**<br>• SND: no significant difference between pre- and post-surgery.<br>• MRND: The amplitude and response area were significantly lower in patients who underwent MND than in those who underwent SND.<br>• RND: patients who underwent RND showed the worst disturbance on EMG examination 5 weeks after the operation. l5 patients who underwent RND had abnormal EMG findings on their operated side. | |
| **33.** | **Authors:** Short et al., 1984 [45].<br>**Title:** Shoulder Pain and Function After Neck Dissection With or Without Preservation of the Spinal Accessory Nerve<br>**Country:** USA<br><br>**Objective:** To compare the difference in degree of pain as well as functional disability of the shoulder in patients who underwent neck dissection with and without spinal | **Type of cancer:** Mixed HNC (larynx, hypopharynx, oropharynx, oral cavity and other locations)<br>**Stage:** Mixed stage<br>**Age:** Mean: 60 (22-86 years)<br>**Gender:** Mixed.<br>M: 27, F: 8.<br>**Duration Post Surgery:** At least after 6 weeks post-operative.<br>**Total sample size:** 43 | **Intervention 1:**<br>Modified radical neck dissection (MRND).<br>**Description:** Not reported.<br>**ND Surgery Area:** Mixed<br>**Reconstruction surgery:** Not reported.<br>**Other cancer treatments:** Radiotherapy and physical therapy.<br>**Total sample:** 23<br>**Follow-up:** 1 (post-operative follow-up) | **Intervention 2:**<br>Radical neck dissection (RND)<br>**Description:**<br>Not reported.<br>**ND Surgery Area:** Not reported.<br>**Reconstruction surgery:**<br>Not reported.<br>**Other cancer treatments:**<br>Radiotherapy and Physical therapy<br>**Total sample:** 12<br>**Follow-up:** 1 (post-operative follow-up) | **Outcome 1:**<br>Pain<br>**Outcome tool:**<br>Unclear pain scale<br><br>**Outcome 2:**<br>Shoulder function<br>**Outcome tool:**<br>Unclear.<br><br>**Outcome 3:**<br>Physical performance<br>**Outcome tool:**<br>Unclear | **Outcome 1:**<br>• All patients who underwent RND had shoulder pain<br>• 9 of the 23 patients (39%) who underwent MRND or conservative neck dissection had none.<br>• Six of the eight patients (75%) who received only neck radiation had no shoulder pain.<br>• Among the 23 patients who had preservation of their spinal accessory nerve, an average of 1. 6 | **Conclusion:**<br>• The results of this study show that, on average, neck dissection patients with their spinal accessory nerve preserved have less pain in their shoulders, less functional disability, and stronger results on their physical examination than those with their spinal accessory nerve sacrificed. |

| | | | | | | | |
|---|---|---|---|---|---|---|---|
| | accessory nerve preservation.<br>**Study Design:** Prospective cohort<br>**Randomized Groups:** 3 MND vs RND vs RT (radiotherapy)<br>**Funding:** Not reported<br>**Setting:** University, Veterans Administration, or Pacific Medical Center Hospitals | | | **Intervention 3:**<br>Radiotherapy<br>**Description:**<br>Of the eight patients who received primary radiation treatment to the neck, none underwent neck dissection. Two of the patients had partial laryngectomy, and one had complete laryngectomy for treatment of their primary tumor. They received neck radiation in doses of 4,500 to 6,600 rads.<br>**ND Surgery Area:**<br>Not reported.<br>**Reconstruction surgery:**<br>Not reported.<br>**Other cancer treatments:**<br>Alone.<br>**Total sample:** 8<br>**Follow-up:** 1 (post-operative follow-up) | | was obtained from a scale of 0 to 5.<br>• Among the 12 patients who had sacrificed of the spinal accessory nerve, an average of 2. 7 was obtained for pain level.<br><br>**Outcome 2:**<br>• The responses to the four questions about the task performance were averaged.<br>• A value of 0. 8 was obtained for patients who preserved their SAN, an average of 2. 1 was obtained for the 12 patients who sacrificed their nerves, and an average of 0. 1 was found for the radiation treatment-only group.<br><br>**Outcome 3:**<br>• The preserved nerve group (MRND) scored better than the sacrificed nerve group.<br>• The radiation treatment patients uniformly scored 5 in the trapezius and abduction tests. | • It was also found that the patients who received whole neck radiation treatment without neck dissection had little pain, infrequent and insignificant functional disability, and normal strength on physical examination. |
| **34.** | **Authors:** Sobol et al., 1985 [32]. | **Type of cancer:** Not reported.<br>**Stage:** Not reported. | **Intervention 1:**<br>Radical neck dissection (RND) | **Intervention 2:**<br>Modified radical neck dissection (MRND)<br>**Description:** | **Outcome 1:**<br>Muscle strength – shoulder (flexor/abductor) | **Outcome 1:**<br>• The RND group was not statistically different from the MRND group | **Conclusion:**<br>• Those who underwent an RND suffered the most significant |

| | | | | | | | |
|---|---|---|---|---|---|---|---|
| | **Title:** Comparison of Physical Dysfunction After Neck Dissection<br>**Country:** USA<br>**Objective:** To compare the differences in functional shoulder disability that occurred in patients who underwent dissection with or without preservation of the spinal accessory nerve, and with or without complete dissection of the nerve.<br>**Study Design:** Prospective cohort<br>**Randomized Groups:** 3 (RND, MRND and SOMND)<br>**Funding:** Not reported<br>**Setting:** University of Oklahoma Teaching Hospitals | **Age:** Mean: 56 (**21**-82 years)<br>**Gender:** Mixed<br>M: 25, F: 10<br>**Duration Post Surgery:** After 16 weeks of surgery (range 11 to 39 weeks)<br>**Total sample size:** 35 | **Description:** A classical radical neck dissection involved a sacrifice of the spinal accessory nerve, the sternocleidomastoid muscle (SCM), and the internal jugular vein. Included in this category were a few patients in whom the sternocleidomastoid muscle and accessory nerve were sacrificed, with the jugular vein preserved.<br>**ND Surgery Area:** Not reported.<br>**Reconstruction surgery:** Not reported.<br>**Other cancer treatments:** Radiotherapy.<br>**Total sample:** 11<br>**Follow-up**: 1 (post-operative follow-up) | Modified radical neck dissection was defined as resectioning all lymphatic contents in the anterior and posterior neck triangles while preserving the spinal accessory nerve. The nerve was dissected from exiting the skull base to inserting into the trapezius muscle. The sternocleidomastoid muscle was sacrificed, and the internal jugular vein was sometimes preserved.<br>**ND Surgery Area:** Not reported.<br>**Reconstruction surgery:** Not reported.<br>**Other cancer treatments:** Radiotherapy<br>**Total sample:** 21<br>**Follow-up:** 1 (post-operative follow-up)<br><br>**Intervention 3:** Supraomohyoid neck dissection (SOND)<br>**Description:** | **Outcome tool:** Using 1 and 5-pound weight<br><br>**Outcome 2:** ROM- Shoulder (Abd/flex)<br>**Outcome tool:** Goniometer<br>.<br>**Outcome 3:** Muscle activation (trapezius)<br>**Outcome tool:** EMG<br><br>**Outcome 4:** Posture<br>**Outcome tool:** Shoulder symmetry was analyzed by measuring shoulder droop, presence or absence of shoulder protraction, and flaring of the scapula. | 16 weeks postoperatively compared based on absolute strength or range of motion changes.<br>• However, the SOND group was statistically different from the RND and MRND when compared at 16 weeks based on absolute change in abduction and flexion measurements using 0,1, and 5-pound weights ($p < 0.05$).<br><br>**Outcome 2:**<br>• RND was not statistically different from the MRND group 16 weeks postoperatively in shoulder ROM<br>• However, the SOND was statistically different from both RND and MRND groups when compared at 16 weeks for shoulder ROM<br><br>**Outcome 3:**<br>• There was a statistically significant difference between the RND and MRND groups when they were compared on the basis of electromyogram data 16 | reduction in shoulder movement and had severely abnormal electromyograms.<br>• Those who underwent MRND with preservation of the SAN suffered less loss of shoulder function than the RND group, but not to a significant degree at 16 weeks.<br>• However, the electromyograms of patients who underwent MRND were significantly better than those of the RND group, which suggests that these patients may improve with time.<br>• Patients who underwent SOND that involved minimal dissection of the spinal accessory nerve had minimal loss of shoulder function and, usually, normal electromyograms at 16 weeks that documented less injury to the SAN. |

| | | | | | | | |
|---|---|---|---|---|---|---|---|
| | | | | The SOND involved dissection and removal of the lymphatic contents from the submandibular triangle and the jugulodigastric, mid jugular, and upper posterior triangle regions of the neck, with preservation of the internal jugular vein, the spinal accessory nerve, and the SCM. In this procedure, the nerve is only minimally dissected.<br>**ND Surgery Area:** Not reported.<br>**Reconstruction surgery:** Not reported.<br>**Other cancer treatments:** Radiotherapy<br>**Total sample:** 6<br>**Follow-up:** 1 (post-operative follow-up) | | weeks postoperatively ($p<0.05$).<br>• The electromyograms of the SOND were significantly different from those of the MRND and RND groups 16 weeks postoperatively ($p<0.05$).<br><br>**Outcome 4:**<br>• The SOND group statistically differed from both the MRND and RND groups when compared on the basis of protraction and droop ($p<0.05$). | |
| **35.** | **Authors:** Orhan et al., 2007 [47].<br>**Title:** Spinal accessory nerve function after neck dissections<br>**Country:** Turkey | **Type of cancer:** Mixed HNC (Oral and larynx)<br>**Stage:** Mixed stage<br>**Age:** Mean: 60.47 (47 to 75 years)<br>**Gender:** Mixed | **Intervention 1:** Radical neck dissection (RND)<br>**Description:** Not reported.<br>**ND Surgery Area:** Mixed (Unclear.). | **Intervention 2:** Functional Neck Dissection (FND)<br>**Description:** Not reported.<br>**ND Surgery Area:** Mixed | **Outcome 1:** Muscle activation: (upper extremities/trapezius)<br>**Outcome tool:** EMG<br><br>**Outcome 2:** | **Outcome 1:**<br>• Both groups had low postoperative EMG scores, compared with preoperative values, and this was statistically | **Conclusion:**<br>• Following the operation, motor amplitudes decreased in both groups. As expected, the decreases in amplitude and EMG |

| | | | | | | | |
|---|---|---|---|---|---|---|---|
| | **Objective:** To use objective techniques to measure shoulder function and its impairment in patients undergoing RND and FND procedures.<br>**Study Design:** Prospective cohort<br>**Randomized Groups:** 2 (RND vs FND)<br>**Funding:** Not reported<br>**Setting:** Departments of Neurology and ENT and Head and Neck Surgery | (M: 20, F: 1).<br>**Duration Post Surgery:** After three weeks of surgery<br>**Total sample size:** 21 | **Reconstruction surgery:** Not reported.<br>**Other cancer treatments:** Radiotherapy.<br>**Total sample:** 10<br>**Follow-up**: 4 (pre and post-operative follow-up) 3 weeks, 3 months and 9 months | **Reconstruction surgery:** Not reported.<br>**Other cancer treatments:** Radiotherapy<br>**Total Sample:** 32<br>**Follow-up**: 4 (pre and post-operative follow-up) 3 weeks, 3 months and 9 months | Shoulder disability neck disability<br>**Outcome tool:** Neck dissection impairment index (NDII) | significant ($p = 0.001$ for FND, $p = 0.006$ for RND).<br>• However, in the RND group, the difference between the pre-operative and post-operative EMG scores was greater than that in the FND group. $P< 0.05$ for all follow-ups.<br><br>**Outcome 2:**<br>• The FND group's questionnaire scores for pain, neck and shoulder stiffness, and disability in lifting heavy objects, light objects and reaching overhead were significantly lower than those of the RND group ($p<0.001$). | score were more prominent in the RND group.<br>• The FND group scores for pain, neck and shoulder stiffness, and disability in heavy object lifting, light object lifting, and reaching overheads were significantly lower than those of the RND group. |
| **36.** | **Authors:** Muhammet Dilber et al., 2007 [49].<br>**Title:** The relationship between shoulder pain and damage to the cervical plexus following neck dissection<br>**Country:** Turkey<br>**Objective:** To evaluate the relationship between cervical plexus damage following neck dissection and shoulder pain.<br>**Study Design:** Prospective cohort | **Type of cancer:** Larynx or laryngeal cancer<br>**Stage:** Mixed stage<br>**Age:** Mean: 57. 4 years (47–72 years)<br>**Gender:** Mixed<br>M: 16; F :1<br>**Duration Post Surgery:** After two weeks postoperative<br>**Total sample size:** 17 | **Intervention 1:** Selective neck dissection (SND)- Spared cervical plexus side<br>**Description:** Bilateral lateral selective neck dissection (II, III, IV) in 10 patients (59%) and bilateral anterolateral selective neck dissection (II, III, IV, VI) in 7 patients. In all patients, the cervical plexus was | **Intervention 2:** Selective neck dissection (SND) - Sacrificed cervical plexus side.<br>**Description:** Bilateral lateral selective neck dissection (II, III, IV) in 10 patients (59%) and bilateral anterolateral selective neck dissection (II, III, IV, VI) in 7 patients. In all patients, the cervical plexus was | **Outcome 1:** Sensation - dermatomes of the occipital nerve, C2, C3, and C4 on both sides of the neck<br>**Outcome tool:** Sensorial perception score<br><br>**Outcome 2:** Pain<br>**Outcome tool:** Visual Analogue Scale (VAS) and Verbal Rating Scale (VRS) | **Outcome 1:**<br>• The scores and degrees of sensorial perception obtained in the postoperative 1st, 3rd, and 6th months were statistically more favorable in the sides of the neck where the cervical plexus was spared ($p < 0.05$).<br><br>**Outcome 2:**<br>• The mean degrees and scores of shoulder pain | **Conclusion:**<br>• Damage to the cervical plexus during neck dissection causes loss of sensorial innervation of the neck, but sacrificing the cervical plexus during selective neck dissection has no adverse effect on shoulder pain. |

| | | | | | | | |
|---|---|---|---|---|---|---|---|
| | **Randomized Groups:** 2 (SND (spared cervical plexus), SND (sacrificed cervical plexus)) **Funding:** Not reported **Setting:** Department of Otorhinolaryngology of Uludag University | | sacrificed on one side of the neck and spared on the other, whereas the accessory nerve was spared on both sides of the neck. **ND Surgery Area:** Larynx **Reconstruction surgery:** No. **Other cancer treatments:** Radiotherapy. **Total sample:** 17 **Follow-up:** 4 (post-operative follow-up): 2 weeks, one month, three months, and six months. | sacrificed on one side of the neck and spared on the other, whereas the accessory nerve was spared on both sides of the neck. **ND Surgery Area:** Larynx **Reconstruction surgery:** No. **Other cancer treatments:** Radiotherapy. **Total sample:** 17 **Follow-up:** 4 (post-operative follow-up): 2 weeks, one month, three months, and six months. | | experienced during rest and movement were not found to differ significantly in either the VRS or the VAS evaluations in the sides with and without sparing of the cervical plexus (p > 0. 05).<br>• All patients scored mild to moderate pain after surgery at follow-up. | |
| **37.** | **Authors:** Adin Selcuk et al., 2008 [48]. **Title:** Shoulder function in various types of neck dissection. Role of spinal accessory nerve and cervical plexus preservation **Country:** Turkey **Objective:** To show the effects of functional ND versus anterolateral ND on shoulder function. **Study Design:** Prospective cohort **Randomized Groups:** 2 (Functional ND and anterolateral ND procedure) | **Type of cancer:** Larynx or laryngeal cancer **Stage:** Mixed stage T2-T4, N0-N2b, M0. **Age:** Not reported. **Gender:** Not reported. **Duration Post Surgery:** Unclear **Total sample size:** 23 | **Intervention 1:** Functional neck dissection (FND) **Description:** Dissection of neck regions 1 through 5, sparing the accessory nerve, SCM, and internal jugular vein. **ND Surgery Area:** Larynx. **Reconstruction surgery:** Not reported. **Other cancer treatments:** | **Intervention 2:** Selective neck dissection (SND) anterolateral neck dissection **Description:** Dissection of the neck (regions 1 through 4). **ND Surgery Area:** Larynx **Reconstruction surgery:** Not reported. **Other cancer treatments:** Radiotherapy **Total sample:** 14 | **Outcome 1:** Shoulder disability **Outcome tool:** Shoulder pain and disability index (SPADI)<br><br>**Outcome 2:** ROM- shoulder (Abd/Add/Flex/Int rotation and Ext rotation) **Outcome tool:** Goniometer<br><br>**Outcome 3:** Accessory nerve function **Outcome tool:** EMG | **Outcome 1:**<br>• Shoulder pain and disability scores were better in group 2 (anterolateral) than in group 1 (functional)<br><br>**Outcome 2:**<br>• ROM of all directions is better in group 2 than in group 1 and showed a significant difference.<br><br>**Outcome 3:**<br>• There was a significant increase in distal motor latencies of all 3 parts of | **Conclusion:**<br>• Preserving the cervical plexus and less disturbance of the spinal accessory nerve are essential to diminish postoperative shoulder disability. The type of neck dissection has a significant influence on shoulder function. |

| | | | | | | | |
|---|---|---|---|---|---|---|---|
| | **Funding:** Not reported<br>**Setting:** Unclear | | Combined: Radiotherapy<br>**Total sample:** 12<br>**Follow-up:** 2 (pre- and post-operative follow-up at 6 month) | **Follow-up**: 1 (post-operative follow-up) | | the trapezius muscles at the sixth week and sixth month postoperatively compared with the preoperative period in both groups.<br>• The two groups had no statistically significant preoperative difference in accessory nerve distal latency.<br>• Distal motor latency of the accessory nerves at postoperative week 6 was significantly lower in group 2 than in group 1 ($p <0. 005$). | |
| **38.** | **Authors:** Oz & Memis 2009 [50].<br>**Title:** Development of musculoskeletal complaints and functional disabilities in patients with laryngeal carcinoma after neck dissection sparing spinal accessory nerve<br>**Country:** Turkey<br>**Objective:** To compare the musculoskeletal complaints and functional disabilities of male subjects who underwent ND sparing the CN XI with healthy age-matched male subjects.<br>**Study Design:** Prospective cohort<br>**Randomized Groups:** 2 | **Type of cancer:** Laryngeal carcinoma<br>**Stage:** Mixed stage<br>**Age:** ND = 60. 05 (9. 78) years and Healthy = 62. 95 (9. 53) years<br>**Gender:** Male only<br>**Duration Post Surgery:** 1. 6 +- 0. 94 years (minimum 1, maximum 4 years).<br>**Total sample size:** 40 | **Intervention 1:** Mixed Neck Dissection SND/ MRND (spare CN XI) FND, functional neck dissection; LND, lateral neck dissection; L/R, left/right; ND, neck dissection; SG, supraglottic.<br>**Description:** Not reported.<br>**ND Surgery Area:** Unclear.<br>**Reconstruction surgery:** Not reported.<br>**Other cancer treatments:** Not reported. | **Intervention 2:** No neck dissection<br>**Total sample:** 20 | **Outcome 1:** ROM - Cervical<br>**Outcome tool:** Goniometer<br><br>**Outcome 2:** PROM-Shoulder<br>**Outcome tool:** Goniometer<br><br>**Outcome 3:** Neck disability<br>**Outcome tool:** Northwick Park Neck Pain Questionnaire (NPNPQ)<br><br>**Outcome 4:** Neck disability<br>**Outcome tool:** | **Outcome 1:**<br>• A significant reduction in cervical ROM was observed in the ND group compared with the control group in each direction of movement except flexion and left rotation ($p < 0. 05$)<br><br>**Outcome 2:**<br>• A decrease in the PROM of the shoulder was also observed more in the ND group compared with the control group (13/20 versus 4/20; $p= 0. 009$). | **Conclusion:**<br>• The morbidity of the neck after ND sparing CN XI consisted of neck and shoulder pain and decreased ROM of these joints. The pain was not severe in most of the patients, but functional disability was more common in the surgery group compared with the healthy population. |

| | | | | | | | |
|---|---|---|---|---|---|---|---|
| | (ND group (spare CN XI) compared patient without ND)<br>**Funding:** Not reported<br>**Setting:** Ear, Nose and Throat Department of Atatürk Training and Research Hospital | | **Total sample:** 20<br>**Follow-up**: 1 (post-operative follow-up) | | Neck Pain and Disability Scale (NPDS) | **Outcome 3:**<br>• No statistically significant difference between the groups.<br><br>**Outcome 4:**<br>• The neck dissection group had statistically significant higher NPDS scores compared with the control group (p= 0.00) | |
| **39.** | **Authors:** Celik et al., 2009 [33].<br>**Title:** Accessory nerve function after level 2B-preserving selective neck dissection<br>**Country:** Turkey<br>**Objective:** To evaluate the relationship between accessory nerve functions and level 2b–preserving selective neck dissection.<br>**Study Design:** Prospective cohort<br>**Randomized Groups:** 2 (Level 2b was spared bilaterally (L2bPSND) vs level 2b was spared unilaterally (16 L2bPSND))<br>**Funding:** Not reported<br>**Setting:** Uludag University, Faculty of Medicine, Department of Otolaryngology-Head and Neck Surgery. | **Type of cancer:** Larynx or laryngeal cancer<br>**Stage:** Not reported.<br>**Age:** 57. 8, ranging from 46 to 73 years.<br>**Gender:** Mixed<br>M: 28, F: 2<br>**Duration Post Surgery:** 21st day and 6th month.<br>**Total sample size:** 41 | **Intervention 1:** Selective neck dissection (SND)level 2b was spared bilaterally (L2bPSND).<br><br>**ND Surgery Area:** Larynx.<br>**Reconstruction surgery:** Not reported.<br>**Other cancer treatments:** Unclear.<br>**Total sample:** 25<br>**Follow-up:** 3 (pre and post-follow-up) 21st day and six months | **Intervention 2:** Selective neck dissection (SND) level 2b was spared unilaterally (16L2bPSND).<br><br>**ND Surgery Area:** Larynx<br>**Reconstruction surgery:** Not reported.<br>**Other cancer treatments:** Unclear.<br>**Total sample:** 16<br>**Follow-up:** 3 (pre and post-follow-up) 21st day and six months | **Outcome 1:** ROM- Shoulder<br>**Outcome tool:** Goniometer<br><br>**Outcome 2:** Scapular muscle strength<br>**Outcome tool:** Daniels and Worthingham 22 scoring system<br><br>**Outcome 3:** ROM - Neck<br>**Outcome tool:** Goniometer<br><br>**Outcome 4:** Neck muscle strength (flexion /extension)<br>**Outcome tool:** Daniels and Worthingham 22 scoring system | **Outcome 1:**<br>• There were no significant differences before and after surgery post-op 21st day and six months (p> 0.05).<br><br>**Outcome 2:**<br>• There were no statistical differences in shoulder muscle strength after 21st day and six months of surgery<br><br>**Outcome 3:**<br>• There were significant differences in neck ROM extension and flexion in group 2 after the 21st day of post-operative, but there were no significant differences after the evaluation of the 6 months. | **Conclusion:**<br>• Results demonstrate that all shoulder functions are protected by preserving level 2b, preventing problems like shoulder syndrome and adhesive capsulitis.<br>• However, neck ROM and strength are generally reduced after SND for both groups. |

| | | | | | | | |
|---|---|---|---|---|---|---|---|
| | | | | | | • Patients have improved their neck ROM after 6 months.<br><br>**Outcome 4:**<br>• Neck muscle strength for flexor showed significant differences in 21 months and six months after surgery.<br>• There were no significant differences in extensor muscle after the 21st and 6 months of surgery for group 1. | |
| **40.** | **Authors:** Guo et al., 2014 [38]<br>**Title:** Supraomohyoid neck dissection and modified radical neck dissection for clinically node-negative oral squamous cell carcinoma: A prospective study of prognosis, complications and quality of life<br>**Country:** China<br>**Objective:** To assess the prognosis and morbidity between supraomohyoid neck dissection (SOND) and modified radical neck dissection (MRND) for oral squamous cell carcinoma (OSCC) in patients with a clinically node-negative neck (cN0). | **Type of cancer:** Oral and oropharyngeal cancer<br>**Stage:** Mixed stage<br>**Age:** SOND: 58. 3 (12. 0) MRND: 57. 3 (11. 5)<br>**Gender:** Mixed<br>M: SOND = 90, MRND = 84.<br>F: SOND = 72 , MRND = 76<br>**Duration Post Surgery:** Post op 1 year<br>**Total sample size:** 332 | **Intervention 1:** Selective neck dissection (SND) Supraomohyoid neck dissection.<br>**Description:** Not reported.<br>**ND Surgery Area:** Mixed<br>**Reconstruction surgery:** Not reported.<br>**Other cancer treatments:** Radiotherapy.<br>**Total sample:** 162<br>**Follow-up:** 1 (post-operative follow-up) | **Intervention 2:** Modified radical neck dissection (MRND).<br>**Description:** Not reported.<br>**ND Surgery Area:** Mixed<br>**Reconstruction surgery:** Not reported.<br>**Other cancer treatments:** Radiotherapy<br>**Total sample:** 160<br>**Follow-up**: 1 (post-operative follow-up) | **Outcome 1:** Shoulder disability<br>**Outcome tool:** The University of Washington Quality of Life (UW-QOL) | **Outcome 1:**<br>• SOND group has better scores than MRND in pain relief (78. 8 ± 12. 6 vs. 75. 2 ± 10. 4, p< 0. 013) and shoulder function (81. 1 ± 16. 6 vs. 68. 1 ± 13. 9, p < 0. 001). | **Conclusion:**<br>• SOND was associated with a lower rate of complication and reduced recovery time in this study.<br>• Importantly, patients who underwent SOND had better performance scores than those who underwent MRND in the pain relief and shoulder function domains of the UW-QOL questionnaire survey at 1-year post-treatment.<br>. |

| | | | | | | | |
|---|---|---|---|---|---|---|---|
| | **Study Design:** Prospective cohort<br>**Randomized Groups:** 2 (SOND vs MRND)<br>**Funding:** Government of China<br>**Setting:** Oral and maxillofacial surgery, Stomatological Hospital, Peking University | | | | | | |
| **41.** | **Authors:** Qiang Sun et al., 2015 [36].<br>**Title:** Does pectoralis major flap harvesting induce upper extremity dysfunction?<br>**Country:** China<br>**Objective:** To investigate the effect of PMM flap surgery on upper extremity function.<br>**Study Design:** Prospective cohort<br>**Randomized Groups:** 2 (Neck dissection with pectoralis major myocutaneous (PMM) flap vs without pectoralis major myocutaneous (PMM) flap))<br>**Funding:** No funding<br>**Setting:** First Affiliated Hospital of China Medical University, Shenyang, China | **Type of cancer:** Oropharyngeal and oral cancer<br>**Stage:** Mixed stage<br>**Age:** Mean: 56. 8 (44–78 years).<br>**Gender:** Male<br>**Duration Post Surgery:** after 1 year<br>**Total sample size:** 92 | **Intervention 1:** SND/MRND with pectoralis major myocutaneous (PMM) flap<br><br>**ND Surgery Area:** Mixed<br>**Reconstruction surgery:** Yes<br>**Other cancer treatments:** Radiotherapy<br>**Total sample:** 46<br>**Follow-up**: 2 (pre and post-operative follow-up after 1 year) | **Intervention 2:** SND/MRND with other reconstruction (non-pectoralis major myocutaneous (PMM) flap<br><br>**ND Surgery Area:** Mixed<br>**Reconstruction surgery:** Yes<br>**Other cancer treatments:** Radiotherapy<br>**Total sample:** 46<br>**Follow up**: After 1 year | **Outcome 1:** Shoulder disability<br>**Outcome tool:** Disability of the arm, shoulder and hand (DASH) | **Outcome 1:**<br>• There was no significant difference between pre- and postoperative DASH scores in the control group.<br>• In the PMM flap group, the postoperative DASH score was significantly higher than the preoperative score (p< 0. 001).<br>• Correlation analyses revealed that flap size was significantly associated with post-operative DASH score in the PMM flap group (r 1⁄4 0. 901, p < 0. 001). | **Conclusion:**<br>• PMM flap reconstruction resulted in significantly higher DASH scores (i.e., more extensive upper extremity disability) compared with preoperative values. ND with PMM had a higher disability than non-PMM reconstruction. |
| **42.** | **Authors:** Garzaro et al., 2015 [42]. | **Type of cancer:** Mixed HNC (Oral cavity , Oropharynx , | **Intervention 1:** Mixed Neck Dissection. SND or MRND with | **Intervention 2:** Mixed Neck Dissection | **Outcome 1:** ROM- Shoulder<br>**Outcome tool:** | **Outcome 1:**<br>• The group with preservation of the | **Conclusion:**<br>• Preserving the cervical root branches during |

| | | | | | | | |
|---|---|---|---|---|---|---|---|
| | **Title:** A study of neck and shoulder morbidity following neck dissection: The benefits of cervical plexus preservation.<br>**Country:** Italy<br>**Objective:** To evaluate the hypothesis that preservation of cervical root branches of the cervical plexus is associated with greater shoulder mobility, less of sensation in the face and neck and quality of life<br>**Study Design:** Prospective cohort<br>**Randomized Groups:** 2 Neck dissection with/without (Group 1: preservation cervical root branches) VS (Group 2: Removal Cervical root branch)<br>**Funding:** Not reported<br>**Setting:** Unclear | Hypopharyx , Larynx , Paranasal ,Nasopharynx and Parotid gland.<br>**Stage:** Not reported.<br>**Age:** Mean = 53. 4 (34-78years)<br>**Gender:** Mixed M:47; F:7<br>**Duration Post Surgery:** Not reported.<br>**Total sample size:** 54 | preservation of cervical root branches<br>**ND Surgery Area:** Mixed<br>**Reconstruction surgery:** Not reported.<br>**Other cancer treatments:** Radiotherapy or /and physiotherapy<br>**Total sample:** 23<br>**Follow-up:** 1 (post-operative follow-up) | SND or MRND with sacrifice cervical root branches.<br>**ND Surgery Area:** Mixed<br>**Reconstruction surgery:** Not reported.<br>**Other cancer treatments:** Radiotherapy or /and physiotherapy<br>**Total sample:** 31<br>**Follow-up:** 1 (post-operative follow-up) | Arm Abduction Test (AAT)<br><br>**Outcome 2:** Quality of life<br>**Outcome tool:** University of Washington Quality-of-Life (UW-QOL) | cervical root branch has greater shoulder abduction when compared to a group with the sacrificed cervical root branch. p=0.023.<br><br>**Outcome 2:**<br>• Patients with preservation of the cervical root branch have higher scores in the shoulder domain in the UWQOL compared to the group that sacrificed the cervical root branch (p=0.042). | neck dissection may improve QOL by providing a better shoulder range of motion, less loss of sensation in the neck, and less pain in the neck and shoulder. |
| **43.** | **Authors:** Prasad et al., 2008 [37].<br>**Title:** Assessment of shoulder function after functional neck dissection and selective neck dissection (Levels I, II, III) in patients with carcinoma of tongue: a comparative study<br>**Country:** India<br>**Objective:** To evaluate and compare shoulder function | **Type of cancer:** Tongue<br>**Stage:** Not reported.<br>**Age:** SND = mean 54. 2 years.<br>FND = mean 53. 13,<br><br>**Gender:** Mixed<br>SND = 24 males, 26 females<br>FND = 27 males, 23 females | **Intervention 1:** Selective neck dissection (SND) (levels I, II, III).<br>**Description:** Not reported.<br>**ND Surgery Area:** Oral cavity.<br>**Reconstruction surgery:** No.<br>**Other cancer treatments:** | **Intervention 2:** Functional Neck Dissection (FND)<br>**Description:** Not reported.<br>**ND Surgery Area:** Oral cavity.<br>**Reconstruction surgery:** No.<br>**Other cancer treatments:** Unclear. | **Outcome 1:** Pain<br>**Outcome tool:** VAS<br><br>**Outcome 2:** Shoulder disability<br>**Outcome tool:** Combination of two questionnaires SDQ and GARS | **Outcome 1:**<br>• Pain is present in both treatment groups. No significant difference in the pain values was found between FND and SND (levels I, II, III). The highest mean score was 5. 92. | **Conclusion:**<br>• The degree of shoulder morbidity is much higher in patients who have undergone FND as compared to SND (levels I, II, III) as a treatment modality for carcinoma tongue, even though both treatment options are nerve-preserving. |

| | | | | | | | |
|---|---|---|---|---|---|---|---|
| | with respect to pain and disability in patients who have undergone nerve-sparing neck dissection i. e. selective neck dissection (levels I, II, III) and functional neck dissection as a part of their treatment modal<br>**Study Design:** Prospective cohort<br>**Randomized Groups:** 2 (SND vs FND)<br>**Funding:** Not reported<br>**Setting:** Head and Neck Division of Surgical Oncology Department at Regional<br>Cancer Centre and Department of Oral and Maxillofacial surgery | **Duration Post Surgery:** At least 6 months after surgery (mean: 8 months)<br>**Total sample size:** 105 | Unclear.<br>**Total sample:** 50<br>**Follow-up:** 1 (post-operative follow-up at least 6 months after surgery) | **Total sample:** 55<br>**Follow-up:** 1 (at least 6 months after surgery) | | **Outcome 2:**<br>• On a comparison, patients who have undergone FND have significantly higher severity of disability when compared to SND (levels I, II, III), especially while dressing, hair washing, doing heavy household chores, and washing dishes/clothes. | |
| **44.** | **Authors:** Chan et al., 2015 [31]<br>**Title:** Shoulder Dysfunction after Selective Neck Dissection in Recurrent Nasopharyngeal Carcinoma<br>**Country:** Hong Kong<br>**Objective:** To investigate the incidence of nodal micrometastasis in patients with recurrent NPC and clinically N0 status. The shoulder function after SND in the same cohort of patients was subsequently assessed using a health- | **Type of cancer:** Nasopharyngeal cancer<br>**Stage:** Mixed stage<br>**Age:** 52. 8<br>**Gender:** Mixed (unclear number)<br>**Duration Post Surgery:** After 1 and 2 years<br>**Total sample size:** 46 | **Intervention 1:** Selective neck dissection (SND)<br>**Description:** All patients underwent nasopharyngectomy via the maxillary swing approach and ipsilateral SND. The spinal accessory nerve (SAN) was carefully preserved,<br>**ND Surgery Area:** Nasopharyngeal.<br>**Reconstruction surgery:** | | **Outcome 1:** Shoulder disability<br>**Outcome tool:** Disability of the arm, shoulder and hand (DASH) | **Outcome 1:**<br>• The mean (SD) DASH score for the first year after treatment was 44. 2 (10. 1; range, 28. 0-66. 5).<br>• The mean (SD) DASH score for the second year after treatment was 46. 3 (12. 4; range, 22. 3-70. 5),<br>• There was no statistically significant change (p = 0. 09) compared with the score obtained 1 year after surgery. | **Conclusion:**<br>• Selective neck dissection causes significant and long-lasting shoulder morbidity that adversely affects the quality of life of patients with recurrent NPC with clinically N0 status. |

| | | | | | | | |
|---|---|---|---|---|---|---|---|
| | related, self-reported questionnaire.<br>**Study Design:** Prospective cohort<br>**Randomized Groups:** 1(SND)<br>**Funding:** Hong Kong UGC Area of Excellence (AoE) scheme.<br>**Setting:** Unclear | | Subplatysmal skin flap.<br>**Other cancer treatments:** Radiotherapy, chemoradiotherapy and physiotherapy<br>**Total sample:** 46<br>**Follow-up:** 2 (post-operative follow-up after 1 and 2 years) | | | | |
| **45.** | **Authors:** Anehosur et al., 2020[34].<br>**Title:** Does Pectoralis Major Myocutaneous Flap Cause the Shoulder Morbidity: A Clinical Comparative Study<br>**Country:** India<br>**Objective:** To compare the morbidity of shoulder function following modified radical neck dissection with and without Pectoralis Major Myocutaneous muscle flap (PMMC) harvest in head and neck cancer patient<br>**Study Design:** Prospective cohort<br>**Randomized Groups:** 2 (MRND with PMMC flap vs MRND with No PMMC flap group)<br>**Funding:** No funding<br>**Setting:** Unclear | **Type of cancer:** Oral<br>**Stage:** Mixed stage Stage III/IV carcinoma<br>**Age:** MRND with PMMC = 51. 7 years, MRND with No PMMC flap Group-2 = 46. 6 years<br>**Gender:** Mixed<br>**Duration Post Surgery:** After 3 months<br>**Total sample size:** 40 | **Intervention 1:** Modified radical neck dissection (MRND) with PMMC flap.<br>**ND Surgery Area:** Mixed<br>**Reconstruction surgery:** Pectoralis Major Myocutaneous muscle flap<br>**Other cancer treatments:** Radiotherapy or chemotherapy.<br>**Total sample:** 20<br>**Follow-up:** Post-operative follow-up (3 and 6 months) | **Intervention 2:** Modified radical neck dissection (MRND) with no PMMC flap (other reconstruction).<br>**ND Surgery Area:** Mixed<br>**Reconstruction surgery:** Other forms of reconstruction methods such as free fibula, radial forearm, anterolateral thigh flaps, and skin graft.<br>**Other cancer treatments:** Radiotherapy or chemotherapy<br>**Total Sample:** 20<br>**Follow-up:** Post-operative follow-up (3 and 6 months) | **Outcome 1:** Shoulder disability<br>**Outcome tool:** Shoulder Disability Questionnaire (SDQ<br><br>**Outcome 2:** ROM-Shoulder<br>**Outcome tool:** Goniometer<br><br>**Outcome 3:** Muscle strength<br>**Outcome tool:** Manual Muscle Testing (MMT) | **Outcome 1:**<br>• In Group-1, 57. 5% of patients had shoulder disability according to SDQ in the third month, and disability reduced to 33. 8% in the sixth month after physiotherapy intervention.<br>• In Group-2, 54. 8% of patients had shoulder disability in the third month, and disability was reduced to 28.1% in the sixth month after physiotherapy intervention.<br><br>**Outcome 2:**<br>• Both groups showed limited ROM in flexion and shoulder Abduction after three months and six months of post-op (with range flexion = 102 | **Conclusion:**<br>• This study's results suggest that harvesting the Pectoralis Major Myocutaneous muscle flap does not intensify the morbidity of the shoulder, which is documented in RND or MRND. |

| | | | | | | | |
|---|---|---|---|---|---|---|---|
| | | | | | | - 113 degrees, abduction = 80 - 95 degrees).<br><br>**Outcome 3:**<br>• In both groups, MMT revealed a decreased strength in all ROM i. e. flexion-extension, abduction–adduction, internal and external rotation.<br>• In the third month postoperatively in Group 1 the muscle strength was 60. 75%, and in Group 2 it was 66. 75%.<br>• There was no statistically significant difference in muscle strength in Group 1 and Group 2 (p = 0. 096). | |
| **46.** | **Authors:** Imai et al., 2021 [35]<br>**Title:** Shoulder function after neck dissection: Assessment via a shoulder-specific quality-of-life questionnaire and active shoulder abduction<br>**Country:** Japan<br>**Objective:** To investigate shoulder function after ND. The shoulder function was as- sessed by measuring the range of active shoulder abduction (ASA), while the qualitative recovery of the | **Type of cancer:** Mixed HNC (Hypopharynx, Larynx, Sinonasal cavity, Thyroid , Salivary gland and others<br>**Stage:** Unclear.<br>**Age:** Mean: 59. 9 years.<br>**Gender:** Mixed M: 53; F: 13<br>**Duration Post Surgery:** At least 1 month after surgery<br>**Total sample size:** 66 | **Intervention 1:** Mixed Neck Dissection (SND and MRND)<br>**Description:** Not reported.<br>**ND Surgery Area:** Mixed<br>**Reconstruction surgery:** Not reported.<br>**Other cancer treatments:** Radiotherapy and physiotherapy<br>**Total sample:** 66 | | **Outcome 1:**<br>ROM- Shoulder<br>**Outcome tool:**<br>Goniometer<br><br>**Outcome 2:**<br>Shoulder disability and QOL<br>**Outcome tool:**<br>Western Ontario Rotator Cuff (WORC) questionnaire | **Outcome 1:**<br>• The ASA angle decreased from preoperatively (165. 6 ±0. 98 °) to 1 month postoperatively (96. 5 ±4. 3 °; p < 0. 0001)<br>• The ASA angle remained significantly worse than preoperatively, even at 12 months postoperatively (165. 6 ±0. 98) preoperatively versus 157. 8 ±3. 9 ) at 12 months postoperatively; p = 0.042). | **Conclusion:**<br>• Results showed that patients had reduced active shoulder abduction after neck dissection (SND and MRND), and ASA angle and shoulder-specific QOL significantly recovered six months after ND. |

| | | | | | | | |
|---|---|---|---|---|---|---|---|
| | shoulder was assessed using a shoulder-specific quality-of-life (QOL) questionnaire<br>**Study Design:** Prospective cohort<br>**Randomized Groups:** 1 Mixed Neck Dissection (SND and MRND)<br>**Funding:** Not reported<br>**Setting:** Department of Head and Neck Surgery, Miyagi Cancer Centre | | **Follow-up:** 5 (1, 3, 6, 9, and 12 months postoperatively) | | | **Outcome 2:**<br>• The WORC score was 60. 4 ±2. 4% at one month postoperatively and recovered to 62. 6 ±2 over time. 3% at three months postoperatively, 67. 9 ±2. 6% at six months postoperatively, and 72. 4 ±2. 6% at nine months postoperatively. | |
| **47.** | **Authors:** Agarwal et al., 2014 [43]<br>**Title:** Prospective evaluation of the quality of life of oral tongue cancer patients before and after the treatment<br>**Country:** India<br>**Objective:** To evaluate the changes in QOL after 12 months post treatment from their pre-treatment levels, using specific questionnaires of well-known acceptability, responsiveness, and validity, with special emphasis on domains such as chewing, swallowing, speech, psychological aspects (mood and anxiety) and pain.<br>**Study Design:** Prospective cohort<br>**Randomized Groups:** 1(SND) | **Type of cancer:** Tongue<br>**Stage:**T1-2N0M0 all patients presented the same stage<br>**Age:** Mean: 51. 62years<br>**Gender:** Mixed<br>M: 34; F: 5<br>**Duration Post Surgery:** 12 months<br>**Total sample size:** 39 | **Intervention 1:** Selective neck dissection (SND).<br>**ND Surgery Area:** Tongue<br>**Reconstruction surgery:** Not reported.<br>**Other cancer treatments:** Radiotherapy<br>**Total Sample:** 39<br>**Follow up**: 1(post-operative follow-up) | | **Outcome 1:** Quality of life<br>**Outcome tool:** QOL questionnaires based on UW-QOL | **Outcome 1:**<br>• Overall, QOL became poor after surgery.<br>• Seven domains were significantly worsened, including the patient's appearance, swallowing, chewing, speech, shoulder pain, discomfort, taste, and saliva production scores. | **Conclusion:**<br>• The study found that the QOL score changes after the treatment in tongue cancer patients. Overall, QOL became poor, but in five domains, it was improved. |

| | | | | | | | |
|---|---|---|---|---|---|---|---|
| | **Funding:** Not reported<br>**Setting:** Department of Oncosurgery and Department of Otorhinolaryngology-Head and Neck Surgery, Sir Ganga Ram Hospital, New Delhi. | | | | | | |
| **48.** | **Authors:** Laverick et al., 2004 [39]<br>**Title:** The Impact of Neck Dissection on Health-Related Quality of Life<br>**Country:** United Kingdom<br>**Objective:** To compare health-related quality of life, particularly shoulder function, by using the University of Washington Quality of Life questionnaire (UW-QOL)12 in patients having no neck dissection and those having a selective dissection.<br>**Study Design:** Prospective cohort<br>**Randomized Groups:** 4 (None vs Unilateral level III - IV vs Unilateral Level V vs Bilateral level III – IV)<br>**Funding:** Not reported<br>**Setting:** Regional Maxillofacial Unit, University Hospital Aintree, Liverpool, England | **Type of cancer:** Oropharyngeal and oral. cancer<br>**Stage:** Mixed stage<br>**Age:** Mean: 62<br>**Gender:** Mixed M: 180; F: 98<br>**Duration Post Surgery:** Not reported.<br>**Total sample size:** 278 | **Intervention 1:** Selective neck dissection (SND).<br>**Description:** Not described.<br>**ND Surgery Area:** Mixed Oral<br>**Reconstruction surgery:** Unclear.<br>**Other cancer treatments:** Alone<br>**Total sample:** No ND:58 Unilateral: 181 Bilateral: 39<br>**Follow-up**: 3 (post-surgery follow-up: 6,12 and 18 months) | | **Outcome 1:** Quality of life<br>**Outcome tool:** University of Washington Quality-of-Life (UW-QOL) | **Outcome 1:**<br>• Pain tended to improve with time, while for all other domains, the trend was for worse scores at 6 months compared with baseline<br>• Patients with bilateral level III to IV dissections had the worst composite UW-QOL profile over time, while those with no neck dissection (most of whom had no flap surgery) had the best profile.<br>• For shoulder disability, patients with no neck dissection and patients with unilateral level III to IV dissections had reasonably similar mean scores.<br>• On the other hand, patients with unilateral level V and bilateral level III to IV dissections recorded much worse scores on average. | **Conclusion:**<br>• There is subjective morbidity associated with shoulder dysfunction after a unilateral level III or IV neck dissection compared with patients undergoing primary surgery without neck dissection.<br>• More extensive surgery in the neck, whether bilaterally removing levels I to III or IV or extending posteriorly to include level V, is associated with statistically significantly worse shoulder dysfunction. |

| | | | | | | | |
|---|---|---|---|---|---|---|---|
| **49.** | **Authors:** Erisen et al., 2004 [41]<br>**Title:** Shoulder Function After Accessory Nerve–Sparing Neck Dissections<br>**Country:** Brazil<br>**Objective:** To observe the effects of preservation of the SAN during ND and postoperative ART on shoulder functions<br>**Study Design:** Prospective cohort<br>**Randomized Groups:** 2 (RND vs MRND)<br>**Funding:** Not reported<br>**Setting:** University of Uludag School of Medicine Department of Otolaryngology | **Type of cancer:** Mixed HNC (didn't report types of HNC)<br>**Stage:** Not reported.<br>**Age:** Study groups: 57 years and control: 50<br>**Gender:** Mixed<br>Study group: M :19; F :4<br>Control group: M:60; F:9<br>**Duration Post Surgery:** 27 months<br>**Total sample size:** 72 | **Intervention 1:**<br>Radical neck dissection (RND).<br>**ND Surgery Area:**<br>Not reported.<br>**Reconstruction surgery:** Not reported.<br>**Other cancer treatments:**<br>Radiotherapy<br>**Total sample:** 23<br>**Follow-up**: 1 (post-operative follow-up) | **Intervention 2:**<br>Modified radical neck dissection (MRND) or SND.<br>**ND Surgery Area:**<br>Not reported.<br>**Reconstruction surgery:**<br>Not reported.<br>**Other cancer treatments:**<br>Radiotherapy<br>**Total sample:** 69<br>**Follow-up**: 1 (post-operative follow-up) | **Outcome 1:**<br>ROM - shoulder (Flex/Abd)<br>**Outcome tool:**<br>Goniometer<br><br>**Outcome 2:**<br>Muscle strength-shoulder (Elevation/Abd/Flex)<br>**Outcome tool:**<br>Manual muscle strength test | **Outcome 1:**<br>• ROM of the shoulder joint, flexion, and abduction were more restricted after RND than after MRND/SND ($p<0.0001$),<br><br>**Outcome 2:**<br>• Elevator and abductor muscles of the shoulder joint became weaker after RND than they did after MRND/SND ($p < 0.01$), but flexor muscle strength was similar after RND and MRND/SND ($p > 0.05$). | **Conclusion:**<br>• Radiotherapy does not have a negative effect on shoulder function after ND.<br>• SAN is always functionally impaired even if we preserve it macroscopically during ND. |
| **50.** | **Authors:** Güldiken et al., 2005 [40]<br>**Title:** Assessment of shoulder impairment after functional neck dissection: Long term results<br>**Country:** Turkey<br>**Objective:** To measure shoulder disability and evaluate patients who underwent FND procedure.<br>**Study Design:** Prospective cohort<br>**Randomized Groups:** 1 (FND)<br>**Funding:** Not reported | **Type of cancer:** Mixed HNC (laryngeal cancer - supraglottic; glottic ; supraglottic + glottic; transglottic)<br>**Stage:** Mixed stage<br>**Age:** Mean: 57. 28<br>**Gender:** Mixed<br>M:24 ; F:1<br>**Duration Post Surgery:** Not reported.<br>**Total sample size:** 25 | **Intervention 1:**<br>Functional ND.<br>**ND Surgery Area:**<br>Mixed<br>**Reconstruction surgery:** No.<br>**Other cancer treatments:**<br>Mixed: Alone or RT<br>**Total sample:** 25<br>**Follow-up**: 5 (pre and post-operative follow-up at the 1st, 3rd, 6th, and 18th months | | **Outcome 1:**<br>ROM - shoulder (Flex/Ext/Abd)<br>**Outcome tool:**<br>Inclinometer<br><br>**Outcome 2:**<br>Shoulder disability<br>**Outcome tool:**<br>Neck dissection impairment index (NDII) | **Outcome 1:**<br>• No significant difference between preoperative and postoperative measurements for most of the movements.<br>• However, the abduction measurements in the first and third months were found to decrease in comparison with preoperative measurements ($p < 0.05$).<br>• No significant difference between preoperative and postoperative 6th and 18th months. | **Conclusion:**<br>• FND is an oncologically safe procedure and gives rise to less shoulder morbidity.<br>• Although ROM improved after 18 months from surgery, pain and stiffness were worse than preoperative values.<br>• The patients with total laryngectomy had lower NDII scores compared to other patients. |

| | | | | | | | |
|---|---|---|---|---|---|---|---|
| | **Setting:** Department of Otorhinolaryngology and Department of Physical Medicine and Rehabilitation | | | | | **Outcome 2:**<br>• We found that NDII scores in patients who underwent total laryngectomy were lower than the patients who underwent partial laryngectomy and glossectomy (p = 0.002 and 0.043, respectively)<br>• The pain and stiffness scores of the final visit were worse than the preoperative scores (p < 0.005) | |
| **51.** | **Authors:** Santana et al., 2018 [44]<br>**Title:** Inspiratory muscle weakness, diaphragm immobility and diaphragm atrophy after neck dissection.<br>**Country:** Brazil<br>**Objective:** Evaluate diaphragm mobility and inspiratory strength after neck dissection.<br>**Study Design:** Prospective cohort<br>**Randomized Groups:** 1 Mixed ND (RND: 6, MRND 1, SND:34, END-2) - level I–VI (unilateral or bilateral neck dissection)<br>**Funding:** FAPESP (Fundação | **Type of cancer:** Mixed HNC (Thyroid, oral cavity and others)<br>**Stage:** Not reported.<br>**Age:** 51<br>**Gender:** Mixed<br>M: 24; F: 19<br>**Duration Post Surgery:** 48 hours preop, 72 hours post-op, and 1-month post-op.<br>**Total sample size:** 43 | **Intervention 1:** Mixed ND (RND: 6, MRND 1, SND:34, END-2) - level I–VI (unilateral or bilateral neck dissection)<br>**ND Surgery Area:** Not applicable.<br>**Reconstruction surgery:** Not reported.<br>**Other cancer treatments:** Not reported<br>**Total sample:** 43<br>**Follow-up**: 3 (pre and post-operative follow-up 72 hours and 1 month) | | **Outcome 1:**<br>Diaphragm function and inspiratory muscle strength.<br><br>**Outcome tool:**<br>Measure maximal inspiratory pressure (MIP) and sniff nasal inspiratory pressure (SNIP) through a digital manometer | **Outcome 1:**<br>• Diaphragm mobility: except for the three patients with diaphragm immobility, mobility did not change immediately after or even 1-month post-op.<br>• Inspiratory strength: MIP and SNIP decreased significantly after dissection surgery. One-month post-op, MIP and SNIP returned to preop levels. | **Conclusion:**<br>• One month after the dissection, inspiratory strength returns to normal, but the diaphragm thickness and thickening fraction decrease, indicating diaphragm atrophy. |

| | | | | | | | |
|---|---|---|---|---|---|---|---|
| | de Amparo e Pesquisa do Estado de Sao Paulo)<br>**Setting:** AC Camargo Cancer Center, an oncological teaching hospital | | | | | | |
| **52.** | **Authors:** Lanisnik et al., 2016 [54]<br>**Title:** The impact on post-operative shoulder function of intraoperative nerve monitoring of cranial nerve XI during modified radical neck dissection<br>**Country:** Slovenia<br>**Objective:** To evaluate the impact of intraoperative nerve monitoring on postoperative shoulder function.<br><br>**Study Design:** Prospective randomized trial<br>**Randomized Groups:** 1 (MRND monitored vs non-monitored nerve)<br>**Funding:** Not reported<br>**Setting:** University Medical Center Maribor, Slovenia. | **Type of cancer:** Mixed HNC (squamous cell carcinoma of the head and neck cancer)<br>**Stage:** Mixed stage<br>**Age:** Mean: 65 (55 -79 years)<br>**Gender:** Mixed<br>M: 18; F: 2<br>**Duration Post Surgery:** 6 weeks and 6 months<br>**Total sample size:** 20 | **Intervention 1:** Modified radical neck dissection (MRND).<br>**ND Surgery Area:** Into the sternocleidomastoid muscle at the same area.<br>**Reconstruction surgery:** Not reported.<br>**Other cancer treatments:** Radiotherapy or /and chemotherapy<br>**Total sample:** 20<br>**Follow-up**: 3 (pre and post-operative follow-up at 6 weeks and 6 months) | | **Outcome 1:** Shoulder disability<br>**Outcome tool:** Constant Murley Score<br><br>**Outcome 2:** Shoulder disability<br>**Outcome tool:** Shoulder pain and disability index (SPADI) | **Outcome 1:**<br>• Pre-operatively, there was no difference in Constant shoulder score between the monitored and non-monitored sides (p = 0. 269).<br>• The Constant shoulder score decreased from the baseline value at 6 weeks on the monitored and non-monitored side (p = 0. 087) and decreased at 6 months on both sides (p = 0. 310).<br><br>**Outcome 2:**<br>• Preoperatively, there was no difference in the SPADI score between the monitored and non-monitored side (p = 0. 243).<br>• At 6 weeks, the SPADI score increased, and thus, the patient's complaints of shoulder symptoms were on the monitored as well as the non-monitored side (p = 0. 326). | **Conclusion:**<br>• Intraoperative monitoring of the CN XI is beneficial at the beginning of the learning curve of neck dissection. It may help in the identification of the motor branch of the CN XI and its anatomical patterns and help develop the surgeon's technique. |

| | | | | | | | |
|---|---|---|---|---|---|---|---|
| | | | | | | • Similar results were also obtained at 6 months on both sides, with further deterioration of the symptoms and an increase in the SPADI score (p=0.290). | |
| **53.** | **Authors:** Ahlberg et al., 2012 [52]<br>**Title:** Morbidity of supraomohyoidal and modified radical neck dissection combined with radiotherapy for head and neck cancer: a prospective longitudinal study<br>**Country:** Sweden<br>**Objective:** To show the investigated impact of supraomohyoidal neck dissection and modified radical neck dissection, both combined with radiotherapy, on cervical range of motion (CROM), mouth opening, swallowing, lymphedema, and shoulder<br>**Study Design:** Prospective cohort<br>**Randomized Groups:** 2<br>SOND vs MRND vs Radiation therapy<br>**Funding:** Not reported<br>**Setting:** Karolinska | **Type of cancer:** Mixed HNC (oral cavity, oropharynx, epipharynx, hypopharynx, larynx, salivary glands, nose and sinus, other)<br>**Stage:** Mixed stage<br>**Age:** 61. 6 years<br>**Gender:** Mixed<br>M: 142<br>F: 64<br>**Duration Post Surgery:** Unclear.<br>**Total sample size:** 206 | **Intervention 1:**<br>Selective neck dissection (SND) with External beam radiation therapy (EBRT).<br>**ND Surgery Area:** Mixed<br>**Reconstruction surgery:** Not reported.<br>**Other cancer treatments:**<br>Radiotherapy or/and chemotherapy<br>**Total sample:** 25<br>**Follow-up**: 3 (post-operative follow-up 2,6 and 12 months) | **Intervention 2:**<br>Modified radical neck dissection (MRND) with EBRT<br>**ND Surgery Area:** Mixed<br>**Reconstruction surgery:**<br>Not reported.<br>**Other cancer treatments:**<br>Radiotherapy or/and chemotherapy<br>**Total sample:** 83<br>**Follow-up**: 3 (post-operative follow-up 2,6 and 12 months)<br><br>**Intervention 3:**<br>External Beam Radiation Therapy<br>**ND Surgery Area:** Mixed<br>**Reconstruction surgery:**<br>Not reported.<br>**Total sample:** 98 | **Outcome 1:**<br>ROM - Jaw<br>**Outcome tool:**<br>Unclear<br><br>**Outcome 2:**<br>ROM – Neck (rotation/flex/ext/lat flex<br>**Outcome tool:**<br>Inclinometer<br><br>**Outcome 3:**<br>Shoulder disability<br>**Outcome tool:**<br>The patient was asked to lift his/her shoulder to the ear and abduct and flex his/her extended arm or, in the case of a noticeable shoulder droop. | **Outcome 1:**<br>• There was a significant reduction in mouth opening in patients who were operated on with an MRND 2 months after treatment (p<0.001), but no significant reduction at 12 months after treatment.<br><br>**Outcome 2: Neck rotation**<br>• There was a significant reduction in cervical rotation in patients who were operated on with an MRND 2 months after treatment (p <0.001).<br>• At 12 months after treatment, cervical rotation was still significantly reduced.<br><br>**Outcome 2: Neck flex/extension**<br>• There was a significant reduction in cervical | **Conclusion:**<br>• CROM and mouth opening were reduced by MRND in combination with EBRT at 2 months after termination of treatment. However, this effect declined to a nonsignificant level after 12 months for all parameters except for cervical rotation.<br>• On the other hand, supraomohyoidal neck dissection did not affect CROM at any point during the first year after treatment.<br>• The significant morbidity in patients who had neck dissection was shoulder disability, which was found in 18% of these patients. |

| | | | | | | | |
|---|---|---|---|---|---|---|---|
| | University Hospital in Stockholm | | | **Follow up**: 3(post-operative follow-up 2,6 and 12 months) | | flexion-extension in patients who were operated on with a MRND 2 months after treatment (p < 0.001)<br>• No significant reduction at 12 months after treatment.<br><br>**Outcome 2: Neck lateral flexion**<br>• There was a significant reduction in cervical lateral flexion in patients who were operated on with an MRND 2 months after treatment (p < 0.001), but no significant reduction at 12 months after treatment.<br><br>**Outcome 3:**<br>• The total incidence of shoulder disability in patients who had neck dissection was 18% (18 of 102.<br>• There was no significant difference between patients who were operated on with MRND (16 of 78; 20%) and those who were operated on with SOND (2 of 24; 8%; p = 0 23). | |

| | | | | | | | | |
|---|---|---|---|---|---|---|---|---|
| **54.** | **Authors:** Speksnijder et al., 2013 [53].<br>**Title:** Neck and shoulder function in patients treated for oral malignancies: A 1-year prospective cohort study<br>**Country:** Netherlands<br>**Objective:** The purpose of this prospective cohort study was to examine and quantify the effect of neck and shoulder function of patients with malignancies in the oral cavity treated with and without a selective, modified, or radical neck dissection.<br>**Study Design:** Prospective cohort<br>**Randomized Groups:** 6 Patient groups: no neck dissection; SND (ipsilateral and contralateral); MRND (ipsilateral and contralateral); bilateral neck dissection<br>**Funding:** Not reported<br>**Setting:** Department of Oral and Maxillofacial Surgery and Special Dental Care, University Medical Center Utrecht | **Type of cancer:** Oral cancer.<br>**Stage:** Mixed stage<br>**Age:** Intervention group: M: 65. 3 years Healthy: M: 60. 3 years<br>**Gender:** Not reported.<br>**Duration Post Surgery:** Before, 4 to 6 weeks after the intervention, 6 months, and 1 year after the intervention<br>**Total sample size:** 145 | **Intervention 1:** Mixed Neck Dissection. (MRND or SND or RND)<br>**ND Surgery Area:** Mixed<br>**Reconstruction surgery:** Yes.<br>**Other cancer treatments:** Radiotherapy.<br>**Total sample:** MRND =16, SND = 55, RND 2, Bilateral ND =10<br>**Follow-up**: 4 (pre- and post-operative follow-up) | **Intervention 2**: Healthy group (N=62) | **Outcome 1:** ROM - Neck<br>**Outcome tool:** Inclinometer<br><br>**Outcome 2:** Function – Shoulder and neck<br>**Outcome tool:** Unclear. | **Outcome 1:**<br>• No significant differences in maximal lateral flexion of the neck were observed between the patients in the four patient groups and the healthy controls.<br>• Shortly after intervention, lateral flexion to the contralateral side of the neck of the MRND group was significantly lower ($p<0.01$) than the lateral flexion of the healthy controls, patients with no neck dissection, and contralateral lateral flexion in the SND group.<br>• One year after intervention, the bilateral neck dissection group showed significantly lower ($p < 0.05$) maximal lateral flexion of the neck than healthy controls and patients without a neck dissection.<br><br>**Outcome 2:**<br>• After the intervention, the patients in the SND, MRND, and bilateral neck dissection groups scored significantly higher ($p <0.05$) on pain | **Conclusion:**<br>• After the intervention, more extended neck dissections induced greater neck and shoulder function deterioration.<br>• The deterioration of the shoulder mobility, from before to shortly after intervention, was not only influenced by the neck dissection but also by the tumor site and extent of reconstructive surgery.<br>• One year after the intervention, patients treated with a bilateral neck dissection still showed deteriorated lateral flexion of the neck. In contrast, patients treated with a unilateral MRND still reported pain while moving the neck. |

| | | | | | | | |
|---|---|---|---|---|---|---|---|
| | | | | | | during neck movement than the healthy controls and those without neck dissection.<br>• Six months after the intervention, the MRND group differed significantly ($p < 0.05$) from the no-neck dissection group. | |
| **55.** | **Authors:** Sharma et al., 2020 [29].<br>**Title:** Neurovascular Complications After Neck Dissection: A Prospective Analysis at a Tertiary Care Centre in South India<br><br>**Country:** India<br>**Objective:** To study various short-term and long-term neurovascular complications following neck dissection.<br>**Study Design:** Prospective<br>**Randomized Groups:** 2 (MRND and SND)<br>**Funding:** Not reported<br>**Setting:** Regional Cancer Centre, Trivandrum | **Type of cancer:** Oral cavity squamous cell carcinoma<br>**Stage:** Mixed stage<br>**Age:** Mean = 56. 5 years<br>**Gender:** Mixed (male to female = 2.5: 1)<br>**Duration Post Surgery:** 6-months<br>**Total sample size:** 105 | **Intervention 1:** Modified radical neck dissection (MRND).<br>**ND Surgery Area:** Oral cavity.<br>**Reconstruction surgery:** Yes.<br>**Other cancer treatments:** Not reported<br>**Total sample:** 29<br>**Follow-up**: 1 (post-operative follow-up at 6 months) | **Intervention 2:** Selective neck dissection (SND).<br>**Description:** All neck dissections were performed via transverse skin crease incision or reverse hockey stick incision. SND: ESOHND (n=70) = removal of levels I–IV, and SND: SOHND (n=8) = removal of levels I–III.<br>**ND Surgery Area:** Oral cavity.<br>**Reconstruction surgery:** Yes.<br>**Other cancer treatments:** Not reported<br>**Total Sample:** SND: ESOHND (n=70) and SND: SOHND (n=8) = removal of levels I–III. | **Outcome 1:** ROM- Shoulder<br>**Outcome tool:** Not reported<br><br>**Outcome 2:** Scapula flaring<br>**Outcome tool:** Not reported.<br>**Description:** Pre-operative. Assessing the scapula flaring<br><br>**Outcome 3:** Shoulder drooping<br>**Outcome tool:** Not reported.<br><br>**Outcome 4:** The angle of mouth deviation<br>**Outcome tool:** Not reported. | **Outcome 1:**<br>• Decreased range of arm abduction <140° in 8 patients.<br>• There was slightly higher shoulder dysfunction noted in the MRND group compared with SND, but no statistical significance.<br><br>**Outcome 2:**<br>• Scapular flaring was present in 7 patients. There was slightly higher shoulder dysfunction noted in the MRND group compared with SND, but the difference could not reach statistical significance.<br><br>**Outcome 3:**<br>• Shoulder droop present in 10 patients. There was slightly higher shoulder dysfunction noted in the | **Conclusion:**<br>• Complications after ND arise due to the complex anatomy of the neck, but a vigilant and proactive approach in the perioperative period can minimize their impact. |

| | | | | | | | |
|---|---|---|---|---|---|---|---|
| | | | | **Follow-up**: 1 (post-operative follow-up at 6 months) | | MRND group compared with SND, but the difference could not reach statistical significance.<br>**Outcome 4:**<br>• On POD1, 32. 5% (n = 28) patients had some angle of deviation.<br>• At 6 months, 13. 4% (n = 11) patients had some angle of deviation.<br>• Marginal mandibular nerve injury was not significantly associated with MRDN and SND (ESOHND and SOHND). | |
| **56.** | **Authors:** Merve et al., 2009 [51].<br>**Title:** Shoulder morbidity after pectoralis major flap reconstruction for head and neck cancer<br>**Country:** United Kingdom<br>**Objective:** To ascertain the effect of PMF harvest on shoulder function while taking into account the confounding effect of neck dissection.<br>**Study Design:** Prospective cohort<br>**Randomized Groups:** 2 (PMF and ND group vs ND-only group.)<br>**Funding:** Not reported<br>**Setting:** Unclear | **Type of cancer:** Mixed HNC (Hypopharynx, tongue, base/supraglottis, oropharynx, larynx and others<br>**Stage:** Mixed stage<br>**Age:** PMF and ND group = 59. 3 years ND group = 60. 5 years<br>**Gender:** Mixed<br>PMF and ND group: M=19, F=3.<br>ND group: M=27, F=8<br>**Duration Post Surgery:** Minimum 6 months<br>**Total sample size:** 57 | **Intervention 1:** PMF and ND group: Patients had different neck dissections (Grade 1, 2, 3).<br>**ND Surgery Area:** Not reported.<br>**Reconstruction surgery:** Yes.<br>**Other cancer treatments:** Radiotherapy<br>**Total sample:** 22<br>**Follow up**: 1(post-operative follow- up, more than 6 months) | **Intervention 2:** Mixed Neck Dissection Patients had different neck dissections (grade 1, 2, 3).<br>**Description:**<br>**ND Surgery Area:** Not reported.<br>**Reconstruction surgery:** Yes.<br>**Other cancer treatments:** Radiotherapy<br>**Total sample:** 35<br>**Follow up**: 1(post-operative follow- up, more than 6 months) | **Outcome 1:** Shoulder disability<br>**Outcome tool:** Constant score method | **Outcome 1:**<br>• The comparison between the two groups shows no difference overall (p = 0.4).<br>• Subgroup analysis according to grade of neck dissection shows no significant difference between the PMF and neck dissection groups. | **Conclusion:**<br>• PMF harvest has little effect on shoulder function, as measured by Constant score, in addition to the effects of neck dissection, especially in patients undergoing RND or ERND.<br>• Therefore, there is minimal or low shoulder morbidity, in additional to neck dissection, caused by PMF reconstruction in head and neck surgery. |

| | | | | | | | |
|---|---|---|---|---|---|---|---|
| 57. | **Authors:** Reddy et al., 2018 [30]<br>**Title:** Nerve and vein preserving neck dissections for oral cancers: a prospective evaluation of spinal accessory nerve function and internal jugular vein patency following treatment<br>**Country:** Italy<br>**Objective:** To study functional outcomes of nerve-sparing with arm abduction test and Neck Dissection Quality of Life questionnaire.<br>**Study Design:** Prospective cohort<br>**Randomized Groups:** 1(MRND)<br>**Funding:** Not reported<br>**Setting:** Unclear | **Type of cancer:** Oral squamous cell cancer<br>**Stage:** Mixed stage<br>**Age:** Mean: 55. 86 years.<br>**Gender:**<br>Not reported.<br>**Duration Post Surgery:** After end of surgery, 1 and 6 months after surgery<br>**Total sample size:** 42 | **Intervention 1:**<br>Modified radical neck dissection (MRND)type II.<br>**ND Surgery Area:**<br>Oral cavity.<br>**Reconstruction surgery:** Yes.<br>**Other cancer treatments:**<br>Radiotherapy and/or chemoradiotherapy<br>**Total sample:** 42<br>**Follow up**: 2 ( pre and post follow-up at 1 month and 6 months) | | **Outcome 1:**<br>ROM-Shoulder<br>**Outcome tool:**<br>Goniometer | **Outcome 1:**<br>• The mean (± SD) preoperative and postoperative scores were 4. 9 (± 0. 04) and 3. 23 (± 0. 53), respectively, indicating a decrease in arm abduction postoperatively (p < 0. 001). | **Conclusion:**<br>• The study highlights nerve and vein dysfunction following MRND type II in patients undergoing surgery for OSCC. |
| 58. | **Authors:** Dziegielewski et al,2019 [91].<br>**Title:** 2b or Not 2b? Shoulder Function After Level 2b Neck Dissection: A Double-Blind Randomized Controlled Clinical Trial<br>**Country:** Canada<br>**Objective:** to determine the impact of level 2b dissection on shoulder function related QOL. | **Type of cancer:** Mixed HNC (Oral cavity, Oropharynx and Larynx)<br>**Stage:** Mixed stage<br>**Age: (**44. 0 – 78. 8)<br>**Gender:** Mixed<br>M: 26, F: 4<br>**Total sample size:** 40<br>**Duration Post Surgery:** pre-post examination | **Intervention 1:**<br>Selective neck dissection (SND): Level 2b was not dissected<br>**Reconstruction surgery:** Not reported.<br>**Other cancer treatments:**<br>Radiotherapy or/and chemotherapy and Physical therapy | **Intervention 2:**<br>Selective neck dissection (SND) Level 2b was dissected (SND [2ab-4]).<br><br>**Reconstruction surgery:**<br>Not reported.<br>**Other cancer treatments:**<br>Radiotherapy or/and chemotherapy and | **Outcome 1:** Shoulder and Neck Disability<br>**Outcome tool:**<br>Neck dissection impairments index (NDII)<br><br>**Outcome 2:** ROM-Shoulder<br>**Outcome tool:**<br>Goniometer<br><br>**Outcome 3:** Muscle strength | **Outcome 1:**<br>• The differences between 6-month postoperative and preoperative scores were statistically significant for both groups (group 1, p= 0.002; group 2, p =0.001). Between-group p=0.008<br><br>**Outcome 2: Shoulder ROM**<br>• Significant differences were observed between | **Conclusion:**<br>• SND, including level 2b, leads to significant long-term deterioration in QOL, active abduction, and nerve conduction amplitude deficiencies. |

| | | | | | | | |
|---|---|---|---|---|---|---|---|
| | Secondary outcomes included objective physical assessments of shoulder function as well as electromyography (EMG) measurements of SAN function.<br>**Study Design:** RCT_ Parallel<br>**Randomized Groups:** 2 (Selective neck dissection (SND): Level 2b was not dissected vs Selective neck dissection (SND) Level 2b was dissected (SND [2ab-4]).<br>**Funding:** Edmonton Civic Employees Research Grant<br>**Setting:** Tertiary-care academic centre (University of Alberta) | | treatment (post-operative)<br>**Total Sample:** 15<br>**Follow-up:** 3 (pre and post follow-up at 4 and 6 months) | Physical therapy treatment (post-operative)<br>**Total Sample:** 15<br>**Follow-up:** 3 (pre and post follow-up at 4 and 6 months) | **Outcome tool:** Seated row test<br>**Description**<br>Participants performed a 1-repetition maximum seated row test to evaluate shoulder retraction muscle group strength changes. | the 4 and 6 months for active abduction and 4 months for active external measurement compared to the 2 groups.<br>• Rotation. Group 2, with 2b removed, has limited ROM when compared to Group 1<br><br>**Outcome 3:**<br>• No significant difference for both group | |
| **59.** | **Authors:** Mathialagan et al., 2016 [93].<br>**Title:** Comparison of spinal accessory dysfunction following neck dissection with harmonic scalpel and electrocautery – A randomized study<br>**Country:** India<br>**Objective:** To study the spinal accessory nerve injury during neck dissection by both harmonic scalpel and electrocautery technique and compared postoperative recovery of | **Type of cancer:** Oral cancer.<br>**Stage:** Mixed stage<br>**Age mean :** 45. 35<br>**Gender:** Mixed<br>M: 31; F: 9<br>**Duration Post Surgery:** postoperative day 1 until 6 months<br>**Total sample size:** 40 | **Intervention 1:**<br>Selective neck dissection (SND)using harmonic scalpel (HS) technique.<br>**Description:** Not reported.<br>**ND Surgery Area:** Unclear.<br>**Reconstruction surgery:**<br>Not reported.<br>**Other cancer treatments:** Alone<br>**Total Sample:** 20 | **Intervention 2:**<br>Selective neck dissection (SND) standard neck dissection technique (sharp dissection and using monopolar and bipolar cautery) (EC)<br>**Description:** Not reported.<br>**ND Surgery Area:** Unclear.<br>**Reconstruction surgery:** Not reported.<br>**Other cancer treatments:** Alone. | **Outcome 1:** Pain<br>**Outcome tool:** VAS<br><br>**Outcome 2:** ROM - Shoulder Abduction<br>**Outcome tool:** Goniometer | **Outcome 1:**<br>• The study shows a significant difference between the two groups. Persisting shoulder pain remains in the electro-cautery group (EC) even after 6 months of SND.<br><br>**Outcome 2:**<br>• The descriptive number shows greater shoulder function improvement occurred in the harmonic scalpel group compared to electrocautery after | **Conclusion:**<br>• Shoulder pain and shoulder abduction recovered almost fully during the follow-up period in patients in whom SND was performed with a harmonic scalpel as compared to electrocautery.<br>• Both techniques have a significant impact on shoulder dysfunction in the postoperative period. |

| | | | | | | | |
|---|---|---|---|---|---|---|---|
| | shoulder function after neck dissection.<br>**Study Design:** RCT - Parallel<br>**Randomized Groups:** 2 (Selective neck dissection (SND)using harmonic scalpel (HS) technique vs Selective neck dissection (SND) standard neck dissection technique (sharp dissection and using mono polar and<br>bipolar cautery) (EC)<br>**Funding:** Not reported<br>**Setting:** Unclear | | **Follow-up:** 5 (pre- and post-operative follow-up at 1 week, 1,3 and 6 months) | **Total Sample:** 20<br>**Follow-up:** 5 (pre- and post-operative follow-up at 1 week, 1,3 and 6 months) | | selective neck dissection at six months. | |
| **60.** | **Authors:** Parikh et al., 2012 [92].<br>**Title:** A double-blind randomised trial of IIb or not IIb neck dissections on electromyography, clinical examination, and questionnaire-based outcomes: a feasibility study<br>**Country:** UK<br>**Objective:** To evaluate the feasibility of comparing differences in EMG or NCS measurements, questionnaires completed by patients, and range of movement after selective supraomohyoid neck dissection with or without level IIb for node-negative oral cancer | **Type of cancer:** Oral cancer (Tongue and floor of the mouth)<br>**Stage:** Mixed stage<br>**Age mean:** 59 - 62<br>**Gender:** Mixed M:22; F:10<br>**Duration Post Surgery:** 6 weeks and 6 months<br>**Total sample size:** 32 | **Intervention 1:** Selective neck dissection (SND) IIa.<br>**Description:** Unclear.<br>**ND Surgery Area:** Oral cavity.<br>**Reconstruction surgery:** Unclear.<br>**Other cancer treatments:** Alone.<br>**Total Sample:** 32<br>**Follow-up:** 3 (pre and post-operative follow-up at 6 weeks and 6 months) | **Intervention 1:** Selective neck dissection (SND)IIb.<br>**Description:** Unclear.<br>**ND Surgery Area:** Oral cavity.<br>**Reconstruction surgery:** Unclear.<br>**Other cancer treatments:** Alone.<br>**Total Sample:** 18<br>**Follow-up:** 3 (pre and post-operative follow-up at 6 weeks and 6 months) | **Outcome 1:** Muscle activation (Trapezius)<br>**Outcome tool:** EMG<br>**Description:** EMG and NC (nerve conduction) studies were done before the operation and at six weeks postoperatively.<br><br>**Outcome 2:** ROM – Shoulder (flex, abd, lateral rotation<br>**Outcome tool:** Goniometer<br><br>**Outcome 3:** Neck and Shoulder disability<br>**Outcome tool:** Neck dissection impairment index (NDII) | **Outcome 1:**<br>• There was a greater mean fall in trapezius M-response amplitude for those who had IIb dissected, which suggested that the inclusion of this level caused additional morbidity.<br>• However, it was insignificant for patients with unilateral dissections, or all necks combined.<br><br>**Outcome 2:**<br>• Both groups have shown a slight reduction in shoulder ROM. No differences were | **Conclusion:**<br>• The data from this feasibility study might show that including IIb makes a clinically meaningful difference, but the numbers were too small to verify this, and a larger multicentre trial is required.<br>• Combining EMG or NCS and questionnaire data before operation and to 6 weeks would suffice and simplify a new study design. |

| | | | | | | | |
|---|---|---|---|---|---|---|---|
| | **Study Design:** RCT - Parallel<br>**Randomized Groups:** 2 (Selective neck dissection (SND) IIa vs Selective neck dissection (SND)IIb.)<br>**Funding:** Not reported<br>**Setting:** Regional Head and Neck Cancer Centre at University Hospital, Aintree,<br>and the Walton Centre for Neurology and Neurosurgery | | | | | identified between groups.<br><br>**Outcome 3:**<br>• Both groups have shown a reduction in shoulder scores. No differences were identified between groups. | |
| **61.** | **Authors:** Yang et al., 2021 [90].<br>**Title:** Application of supraomohyoid neck dissection via retroauricular hairline incision in patients with oral cancer<br>**Country:** China<br>**Objective:** To explore the application of supraomohyoid neck dissection via retroauricular hair-line incision in patients with oral cancer.<br>**Study Design:** RCT.<br>**Randomized Groups:** 2 (Mixed ND vs SOND)<br>**Funding:** Not reported<br>**Setting:** Unclear | **Type of cancer:** Oral cancer<br>**Stage:** Not reported.<br>**Age:** Experimental group = M: 51. 83. Control group = M: 51. 37<br>**Gender:** Mixed M: 41; F: 27<br>**Duration Post Surgery:** 1-3 months<br>**Total sample size:** 68 | **Intervention 1:** Mixed ND<br>**ND Surgery Area:** Unclear.<br>**Reconstruction surgery:** Not reported.<br>**Other cancer treatments:** Not reported.<br>**Total sample:** 34<br>**Follow-up**: 2 (post-operative follow-up at 1 and 3 months after surgery) | **Intervention 2:** Supraomohyoid neck dissection (SOND) via retroauricular hairline incision<br>**ND Surgery Area:** Unclear.<br>**Reconstruction surgery:** Not reported.<br>**Other cancer treatments:** Not reported.<br>**Total sample:** 34<br>**Follow-up:** 2 (post-operative follow-up at 1 and 3 months after surgery) | **Outcome 1:** Shoulder function<br>**Outcome tool:** The Constant-Murley<br><br>**Outcome 2:** Quality of life<br>**Outcome tool:** University of Washington Quality-of-Life (UW-QOL)<br><br>**Outcome 3:** Skin scars<br>**Outcome tool:** Degree of satisfaction to the scars<br>**Description:** Degree of satisfaction to the scars: the patients were scored with their satisfaction on the scar length, size, and visibility. The answer was "satisfactory" or | **Outcome 1:**<br>• The experimental group had statistically significantly better recovery of shoulder joint function (pain, muscle strength, ROM, and daily activities of the shoulder) than the control group.<br>**Outcome 2:**<br>• The experimental group had a significantly better quality of life than the control group.<br><br>**Outcome 3:**<br>• The degree of satisfaction with the scars in the experimental group is statistically significantly better than the control group. | **Conclusion:**<br>• Supraomohyoid neck dissection via retroauricular hairline can reduce the amount of intraoperative blood loss, improve the recovery of shoulder joint function, improve the quality of life, increase the degree of satisfaction to the scars, and reduce the incidence of related complications in patients with oral cancer. |

| | | | | | | | |
|---|---|---|---|---|---|---|---|
| | | | | | "unsatisfactory". The rate of satisfaction was calculated. | | |
| **62.** | **Authors:** Karthikeyan et al.,2023 [56]<br>**Title:** Quality of life and shoulder function among oral cancer patients treated with selective neck dissection<br>**Country:** India<br>**Objective:** To evaluate the ND-related QoL and the shoulder function of the oral cancer patients who underwent SND.<br>**Study Design:** Cross-Sectional<br>**Groups: 1** (SND)<br>**Funding:** No funding<br>**Setting:** Unclear | **Type of cancer:** Oral cancer (Lip, buccal mucosa, upper and lower alveolus ,retro molar trigone, tongue, floor of the mouth, hard palate)<br>**Stage:** Mixed stage<br>Stage II = 13 (10.2%)<br>Stage III = 38 (29.6%)<br>Stage IV = 77 (60.2%)<br>**Age:** 31 and above<br>**Gender:** Mixed<br>M: 85; F: 43<br>**Duration post-surgery:** More than 6 months<br>**Total sample size:** 128 | **Intervention 1:** Selective Neck Dissection (SND) (Level I to Level IV neck nodes)<br>**Description:** Not reported<br>**ND Surgery Area:** Oral<br>**Reconstruction surgery**: Not reported<br>**Other cancer treatments:** Not reported<br>**Total sample:** 128<br>**Follow-up**: 1 (post-operative follow-up) | | **Outcome 1:** ROM - Shoulder<br>**Outcome tool:** Goniometer Arm abduction test (AAT)<br><br>**Outcome 2:** Quality of life<br>**Outcome tool:** ND related quality of life | **Outcome 1:**<br>• The study showed that out of 128 participants, fifty-one (39.84%) participants were able to abduct up to or more than 150° but less than 180° (Score 3 of AAT) followed by 31 (24.22%) participants who could abduct up to or more than 90° but not less than 150° (Score 2 of AAT).<br><br>**Outcome 2**<br>• Out of the 128 patients, 94 (73.8%) had better QoL, and 34 (26.2%) had the worse QoL. | **Conclusion:**<br>• SND, being a more conservative approach sparing the spinal accessory nerve, could still limit the QoL and could affect shoulder function to a certain extent in individuals with oral cancer. |
| **63.** | **Authors:** Ozyrek et al., 2023 [55].<br>**Title:** Decreased muscle strength and scapular muscle endurance associated with shoulder function after neck dissection<br>**Country:** Turkey<br>**Objective:**<br>1. To determine the changes in rotator cuff and scapular muscle strength, and scapular muscle endurance following ND. | **Type of cancer:** Mixed HNC (oral cavity, larynx, thyroid)<br>**Stage:** Not reported<br>**Age:** Median = 51.00 (39.75–65.25)<br>**Gender:** M: 9; F: 5 (35.7%)<br>**Duration post-surgery:** After 3 months<br>**Total sample size:** 14 | **Intervention 1:** Mixed ND: MRND (1) and SND (15) ( preserved SAN).<br>**Description:** Not reported<br>**ND Surgery Area:** Mixed<br>**Reconstruction surgery:** Not reported<br>**Other cancer treatments:** Radiation therapy and/or chemotherapy<br>**Total sample:** 14 | | **Outcome 1:** Shoulder-muscle strength (trapezius, serratus anterior and rotator cuff muscles)<br>**Outcome tool:** Hand-held Dynamometer<br><br>**Outcome 2:** Scapular muscle endurance.<br>**Outcome tool:** Scapular muscle endurance test (SMET) | **Outcome 1:**<br>• A decrease in muscle strength was found at 3 months postoperatively compared to preoperative, except for the subscapularis muscle strength ($p < 0.05$)<br><br>**Outcome 2:**<br>• A decrease in scapular muscle endurance was found at 3 months postoperatively | **Conclusion:**<br>• The study highlights a decrease in both scapular and rotator cuff muscle strength and scapular muscle endurance three months after ND (preserved SAN).<br>• Declines in muscle strength and endurance may be correlated with postoperative deteriorated shoulder |

| | | | | | | | |
|---|---|---|---|---|---|---|---|
| | 2. To investigate the relationship between these changes and postoperative shoulder function.<br>**Study Design:** Cross-sectional<br>**Groups:** 1(Mixed ND: MRND (1) and SND (15) (preserved SAN).<br>**Funding:** Dokuz Eylül University Department of Scientific Research Projects<br>**Setting:** School of Physical Therapy and Rehabilitation, Dokuz Eylül University | | **Follow-up:** 2 (pre- and post-operative follow-up at 3 months) | | **Outcome 3:** Shoulder function<br>**Outcome tool:** Constant Murley Shoulder Score | compared to preoperative ($p < 0.01$)<br><br>**Outcome 3:**<br>• Shoulder function decreased at 3 months postoperatively compared to preoperative ($p < 0.05$). | function in patients with HNC. |
| **64.** | **Authors:** Crimi et al.,2023 [77]<br>**Title:** Does Age Affect the Rate of Spinal Nerve Injury after Selective Neck Dissection? Age as a Prognostic Factor of Spinal Nerve Injury after Selective Neck Dissection<br>**Country:** Italy<br>**Objective:** To investigate whether age is a significant risk factor for spinal nerve injury following selective neck dissection (SND) in patients with head and neck cancer.<br>**Study Design:** Retrospective cohort<br>**Groups: 1** (SND)<br>**Funding:** No funding<br>**Setting:** Maxillo-Facial | **Type of cancer:** Oral Cancer (oral cavity, nasal surface, salivary glands, adenoid cystic carcinoma; and osteosarcoma)<br>**Stage:** Not reported<br>**Age:** M = 62.5 (range: 19–83)<br>**Gender:** Mixed (M:48, F:30)<br>**Duration post-surgery:** After 1 month<br>**Total sample size:** 75 | **Intervention 1:** Selective Neck Dissection (SND)<br>**Description:** Not reported<br>**ND Surgery Area:** Not reported<br>**Reconstruction surgery:** Not reported<br>**Other cancer treatments:** Radiation<br>**Total Sample:** 75<br>**Follow-up:** 2 (post-operative follow-up at 1 month and 6 months | | **Outcome 1:** Shoulder function<br>**Outcome tool:** Shoulder Disability Questionnaire (SDQ)<br>**Follow-up:** 1 and 6 months<br><br>**Outcome 2:** Shoulder pain and function<br>**Outcome tool:** Shoulder pain and disability index (SPADI)<br>**Follow-up:** 2 (post-operative follow-up at 1 month and 6 months | **Outcome 1:**<br>• Group A (younger) has better SDQ (shoulder function) than Group B (older) at 1-month and 6-month follow-up<br><br>**Outcome 2:**<br>• Group A (younger age) has less pain and shoulder function than Group B (older age) at 1 month and 6 months | **Conclusion:**<br>• SAN lesions after SND in HNC patients are one of the most common complications.<br>• This study demonstrated age as a risk factor that predicts either SAN lesion rate or recovery after surgery, which appeared to be better in younger patients. |

| | | | | | | | |
|---|---|---|---|---|---|---|---|
| | Surgery Unit of Policlinico San Marco in Catania, Italy, and the Maxillo-Facial Surgery Unit of Policlinico S. Orsola in Bologna, Italy | | | | | | |
| **65.** | **Authors:** Sakai et al.,2023 [27]<br>**Title:** Shoulder function after neck dissection with level IIb preservation: a prospective observational study<br>**Country:** Japan<br>**Objective:** To investigate whether shoulder syndrome could be reduced by level IIb preservation<br>**Study Design:** Prospective observational study<br>**Groups:** 3 (Group 1, IIb preservation ; Group 2, IIb dissection ; and Group 3, IIb and V dissection group)<br>**Funding:** Not reported<br>**Setting:** Tokai University Hospital, Kanagawa, Japan | **Type of cancer:** Mixed HNC **(**Laryngeal and hypopharyngeal cancer)<br>**Stage:** Mixed stage<br>**Age:** Mean: 66.4- 72.5<br>**Gender:** Not reported<br>**Duration post-surgery:** 3-6 months<br>**Total sample size:** 35<br>Group 1 = 9<br>Group 2 = 16<br>Group 3 = 10 | **Intervention 1:** SND IIb preserving<br>**Description:** The sternocleidomastoid muscle, internal jugular vein, and SAN were all preserved.<br>**ND Surgery Area:** Laryngeal and hypopharyngeal<br>**Reconstruction surgery:** Not reported<br>**Other cancer treatments:** Not reported<br>**Total Sample:** 9<br>**Follow-up:** 3 (Preoperative and at 3 and 6 months) | **Intervention 2:** SND IIb dissection<br>**Description:** Regarding the management of the SAN, in level IIb dissections, the SAN was dissected circumferentially while the fat pad of level IIb was passed underneath.<br>**ND Surgery Area:** Laryngeal and hypopharyngeal<br>**Reconstruction surgery:** Not reported<br>**Other cancer treatments:** Not reported<br>**Total Sample:** 16<br>**Follow-up:** 3 (Preoperative, and at 3 and 6 months)<br><br>**Intervention 3:** SND IIb and V dissection group<br>**Description:** In level IIb dissections, the SAN | **Outcome 1:** ROM - Shoulder (flexion and abduction<br>**Outcome tool:** Goniometer<br><br>**Outcome 2:** Shoulder – strength<br>**Outcome tool:** Hand-held Dynamometer<br><br>**Outcome 3:** Shoulder QOL<br>**Outcome tool:** Neck Dissection Questionnaire and Shoulder QOL | **Outcome 1:**<br>• The early mean reduction in active shoulder ROM was smaller in group 1, but the difference was not statistically significant at 3-month follow-up.<br>• At 6-month follow-up, the values for the three groups differed when compared to pre-operative measurement. Over time, all three groups showed improvement after 6 months.<br><br>**Outcome 2:**<br>• There are no statistically significant differences between groups in muscle strength of shoulder flexion and abduction<br><br>**Outcome 3:**<br>• The three groups had no significant differences for any of the items. However, in terms of | **Conclusion:**<br>• Although the results showed less shoulder dysfunction in the early postoperative period, the difference was insignificant.<br>• Shoulder function tended to improve over time with or without IIb dissection. The QOL in the level IIb preservation group was preserved in the early postoperative period.<br>• Neck dissection with level IIb preservation may help reduce shoulder syndrome and maintain QOL in the early postoperative period. |

| | | | | | | | |
|---|---|---|---|---|---|---|---|
| | | | | was dissected circumferentially while the fat pad of level IIb was passed underneath. The dissection at level V was limited to the anterior part of the trapezius muscle branch of the SAN, and no further posterior dissection was performed.<br>**ND Surgery Area:** Laryngeal and hypopharyngeal<br>**Reconstruction surgery:** Not reported<br>**Other cancer treatments:** Not reported<br>**Total Sample:** 10<br>**Follow-up:** 3 (Pre and post-surgery at 3 and 6 months) | | average scores, group 1 tended to have higher scores and better QOL at follow-up<br>• In contrast, groups 2 and 3 tended to have lower scores but had almost improved at 3 months.<br>• For the NDQ scores, there was no significant difference among the three groups in the average total score at follow-up. | |
| **66.** | **Authors:** Shah et al.,2022[28]<br>**Title:** Shoulder Dysfunction Post Spinal Accessory Nerve Preserving Neck Dissections: Our Experience<br>**Country:** India<br>**Objective:** To analyze shoulder dysfunction in patients who underwent spinal accessory nerve preserving neck dissections. | **Type of cancer:** oropharyngeal cancer (buccal mucosa, tongue, lip)<br>**Stage:** Mixed stage<br>**Age:** 40-60 years<br>**Gender:** M: 80% F :20%<br>**Duration post-surgery:** 10 days<br>**Total sample size:** 45 | **Intervention 1:** Mixed ND (SND = 37 patients MRND = 8 patients) – preserved SAN with bilateral ND<br>**Description:** Unclear<br>**ND Surgery Area:** Unclear<br>**Reconstruction surgery:** Not reported | | **Outcome 1:** Shoulder Pain<br>**Outcome tool:** VAS<br><br>**Outcome 2:** Shoulder ROM<br>**Outcome tool:** Arm Abduction Test | **Outcome 1:**<br>• On post-op day 10, 47% of patients had a pain score of 6 out of 10, whereas 13% had a pain score of 8.<br>• After 3–6 months of physiotherapy, at the end of 6 months, 35 patients out of 45 had improved to score 2, and 2 patients had scored 0. | **Conclusion:**<br>• The incidence of shoulder dysfunction, even in spinal accessory nerve preserving neck dissections, is 100%.<br>• The extent of shoulder dysfunction is variable in this study, where more than 50% of patients scored above 4 |

| | | | | | | | |
|---|---|---|---|---|---|---|---|
| | **Study Design:** A single-centre prospective observational study<br>**Groups:** 1 (Mixed ND – Preserved SAN)<br>**Funding:** No funding<br>**Setting:** A tertiary care centre | | **Other cancer treatments:** Physiotherapy treatment<br>**Total Sample:** 45<br>**Follow-up:** day 10, 1 month and 6 months | | | • There was a statistically significant difference between time points.<br><br>**Outcome 2:**<br>• At postoperative day 10, 40 out of 45 patients had an arm abduction score of 1 (arm abduction less than 90), and 5 had a score of 2(less than 150).<br>• At the end of 6 months, when the patient underwent shoulder physiotherapy as rehabilitation, 27 patients improved to score 4, which is abduction up to 180° with pain or effort, and 15 patients improved to score 3, which is abduction between 150° to 180° and one patient had score 5 which is abduction above 180° without pain or effort.<br>• There was a statistically significant difference between time points. | on the arm abduction test and below 4 on the pain scale at the end of 6 months.<br>• Active rehabilitation in the form of shoulder physiotherapy significantly improved the arm abduction test score and the pain score over 6 months. |
| **67.** | **Authors:** Mishra et al.,2024 [26]<br>**Title:** Shoulder Dysfunction and Quality of Life Following Modified Radical and Selective Neck Dissection: A Prospective Comparative Study | **Type of cancer:** Mixed HNC<br>**Stage:** Mixed<br>**Age:** Group A: 48.18 Group B: 52.16<br>**Gender:** Mixed<br>M: 53<br>F: 12 | **Intervention 1:** MRND<br>**Description:** Intraoperatively, SAN was identified in the anterior triangle and was traced till its insertion into the | **Intervention 2:** SND<br>**Description:** SAN was identified in the anterior triangle. In SND, it was traced till its insertion into the SCM. | **Outcome 1:** Shoulder ROM<br>**Outcome tool:** Arm Abduction Score<br><br>**Outcome 2:** Shoulder disability | **Outcome 1:**<br>• In the MRND group, the decrease in abduction angle was significant till 3 months post-operatively compared to baseline (pre-operative) (p = 0.01). In the SND | **Conclusion:**<br>• It was found that shoulder impairment was significantly greater in individuals who had an MRND due to the involvement of level V dissection. |

| | | | | | | | |
|---|---|---|---|---|---|---|---|
| | **Country:** India<br>**Objective:** To compare the shoulder function in patients undergoing modified radical neck dissection with those undergoing selective neck dissection and to assess the quality of life in both groups.<br>**Study Design:** Prospective longitudinal comparative study<br>**Groups:**2 (MRND vs SND)<br>**Funding:** No funding<br>**Setting:** Department of Otolaryngology and Head and Neck surgery | **Duration post-surgery:** 6 months<br>**Total sample size:** 65 | trapezius in the case of MRND<br>**ND Surgery Area:** Neck<br>**Reconstruction surgery:** Not reported<br>**Other cancer treatments:** No<br>**Total Sample:** 33<br>**Follow-up:** Pre- and post-operative 1 week, 1 month, 3 month and 6 months | **ND Surgery Area:** Neck<br>**Reconstruction surgery:** Not reported<br>**Other cancer treatments:** No<br>**Total Sample:** 32<br>**Follow-up:** Pre- and post-operative 1 week, 1 month, 3 month and 6 months | **Outcome tool:** Shoulder Pain and Disability Index (SPADI)<br><br>**Outcome 3:** Quality of Life<br>**Outcome tool:** Neck Dissection Quality of Life questionnaire (NDQOL) | group, the same finding was noted till 1 month.<br>• There was a significant decrease in the arm abduction angle in MRND compared to SND at 1 week, 1 month, and 6 months postoperatively (p = 0.01, 0.02, and 0.01, respectively).<br>• The proportion of patients having active shoulder abduction angle up to 180° without pain at 6 months was significantly higher in the SND group [29(90.6%)- vs. 21 patients (63.3%)-in the MRND group].<br><br>**Outcome 2:**<br>• SPADI scores were significantly worse at 1 week, 1 month, and 3 months post-operatively than the baseline values in both groups<br>• Intergroup comparison showed SND patients having significantly better SPADI scores at 1 week, 1 month, 3 months, and 6 months post-operatively than MRND (p = 0.01). | • In contrast, those who underwent SND experienced an early and sustained recovery in shoulder function, particularly when they engaged in early rehabilitation.<br>• However, substantial clinical recovery may take several months despite this initial intervention. Therefore, it is essential to inform patients about this timeline during preoperative counseling and to encourage them to continue their therapy through regular follow-up appointments. |

| | | | | | | | |
|---|---|---|---|---|---|---|---|
| | | | | | | **Outcome 3:**<br>• Both groups had lower scores in NDQOL<br>• For NDQOL, the intergroup analysis showed no significant difference in either the 3 months ($p=0.32$) or the 6 months post-operative score ($p=0.44$) between the two groups. | |
