# Supplementary material for: Musculoskeletal Impairments and Dysfunction in Individuals with Head and Neck Cancer Following Surgery with Neck Dissection—A Systematic Review
Source: Life (Basel). 2025 May 17;15(5):800. doi: 10.3390/life15050800 (PMC12112850; doi:10.3390/life15050800)
Supplement: Supplementary file 1 [file life-15-00800-s001.zip › Supplementary_Materials_D.pdf]

**SUPPLEMENTARY MATERIALS D:** Risk of bias assessment across the studies

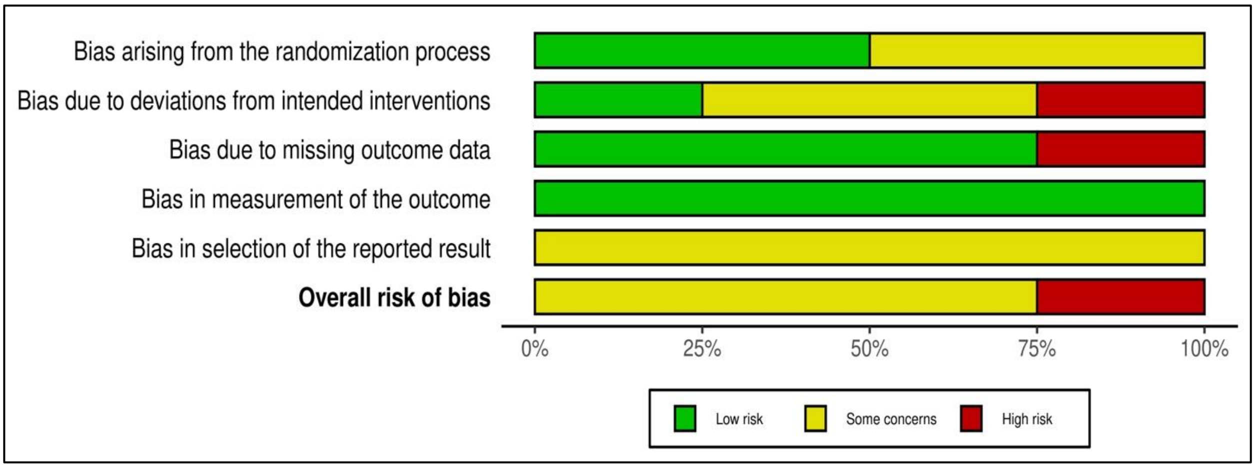


**Figure S1:** Cochrane Risk of Bias assessment across the studies for **RCT**

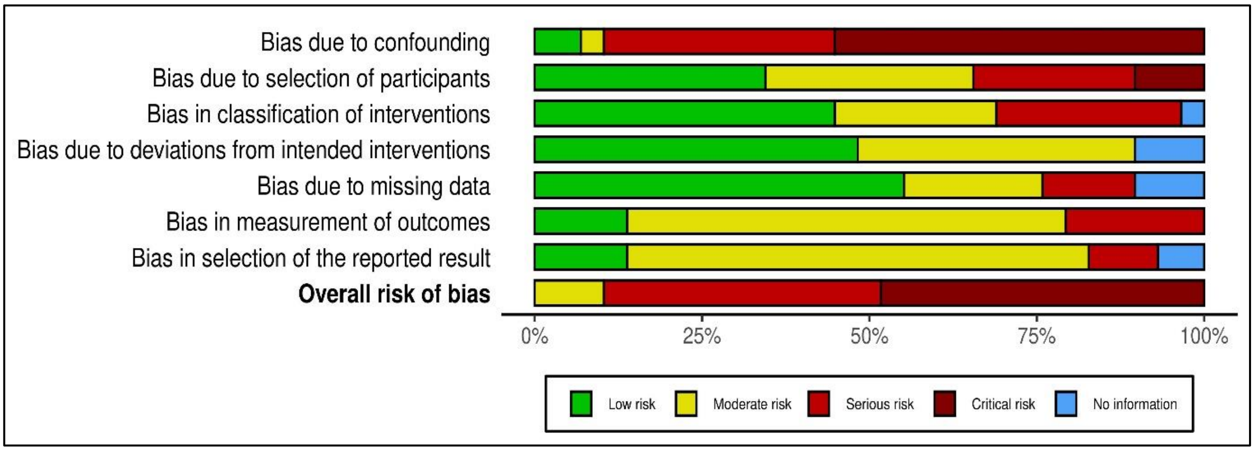


**Figure S2**: ROBINS-I assessment across the studies for **PCS**

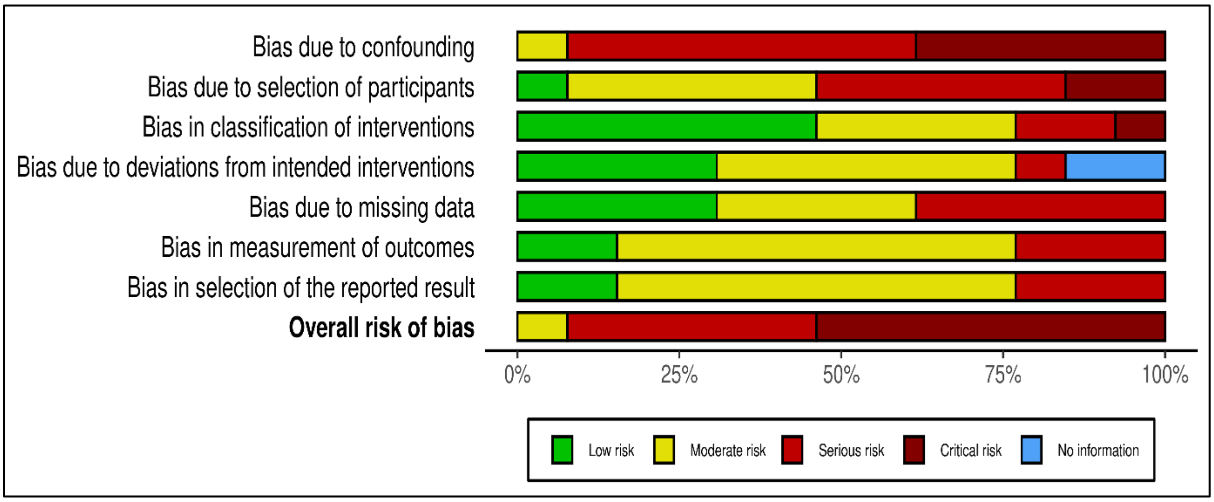


**Figure S3**: ROBINS-I assessment across the studies for **RCS**

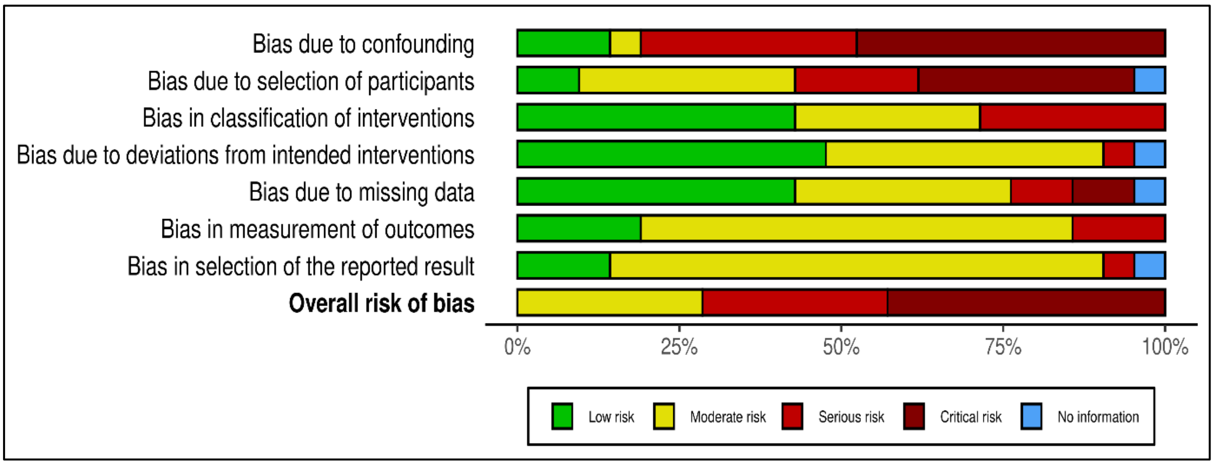


**Figure S4**: ROBINS-I assessment across the studies for **CS studies**
