# Supplementary material for: Musculoskeletal Impairments and Dysfunction in Individuals with Head and Neck Cancer Following Surgery with Neck Dissection—A Systematic Review
Source: Life (Basel). 2025 May 17;15(5):800. doi: 10.3390/life15050800 (PMC12112850; doi:10.3390/life15050800)
Supplement: Supplementary file 1 [file life-15-00800-s001.zip › Supplementary_Materials_E.pdf]

SUPPLEMENTARY MATERIALS E: GRADE table for included studies

Table S1: The overall quality of the evidence with the GRADE approach for **pain intensity** outcome.

Certainty assessment						No of patients		Impact	Certainty	Importance
№ of studies	Study design	Risk of bias	Inconsistency	Indirectness	Imprecision	Intervention	Comparison			
**SHOULDER PAIN**										
**E1.1. RND vs. others (RND sacrificed SAN vs. MRND preserved SAN) (Follow-up: not reported) (Assessed with: HRQOL -Shoulder pain)**										
1	RCS[81]	very serious[a,c]	NA	serious[a,c]	serious[b]	49	129	Both groups have significantly reported higher shoulder pain after the surgery. No differences between procedures	⊕◯◯◯-[a,b,c] Very low	Critical
**E1.2. RND vs. others (RND sacrificed SAN vs. MRND preserved SAN) (Follow-up: 6 weeks post-surgery); (Assessed with: VAS-Shoulder pain)**										
1	PCS[45].	very serious[a,c]	NA	serious[a,c]	serious[b]	12	23	RND with sacrificed SAN significantly had a higher VAS score when compared with MRND with preserved SAN	⊕◯◯◯- [a,b,c] Very low	Critical
**E1.3. RND vs. others (RND sacrificed SAN vs.SND) (Follow-up: 10 days post-surgery) ; (Assessed with: VAS-Shoulder pain)**										
1	CS[69]	very serious[a,c]	NA	serious[a,c]	serious[b]	Unclear		RND statistically significant had a higher VAS score when compared with SND	⊕◯◯◯-[a,b,c] Very low	Critical
**E1.4. MRND vs. others (MRND vs. SND) (Follow-up: range 13.2 days post-surgery); (Assessed with: VAS - Shoulder pain)**										
1	CS [69]	very serious[a,b]	NA	serious[a,c]	serious[b]	Unclear		Participants in MRND scored higher shoulder pain score than those in SND after surgery. However, no significant value was reported	⊕◯◯◯-[a,b,c] Very low	Critical
**E1.5. MRND vs. others (MRND vs. SND) (Follow-up: range more than 1-year post-surgery); (Assessed with: VAS - Shoulder pain)**										
1	CS [60]	very serious[a,b,c]	NA	serious[a,c]	serious[b]	18	57	Participants in MRND scored higher shoulder pain score than those in SND. However, no significant value was reported	⊕◯◯◯-[a,b,c] Very low	Critical
**E1.6. MRND vs. others (MRND vs. SOND) (Follow-up: range more than 1-year post-surgery) ;(Assessed with: UWQOL– Shoulder pain)**										
1	PCS[38]	very serious[a,b,c]	NA	serious[a,c]	serious[b]	160	162	Participants in MRND statistically significant had scored higher shoulder pain scores than those in SND	⊕◯◯◯-[a,b,c] Very low	Critical
**E1.7. SND vs. others (SND vs. non-surgical) (Follow-up: range 2.6 months post-surgery); (Assessed with: VAS - Shoulder pain)**										
1	CS[75]	very serious[a,b,c]	NA	serious[a,c]	serious[b]	18	18	SND statistically significant had caused shoulder pain after the surgery	⊕◯◯◯-[a,b,c] Very low	Critical
**E1.8. SND vs. others (SND with radiotherapy vs. SND without radiotherapy) (Follow-up: range 0.5 month- 9.1 years) ;(Assessed with: VAS- Shoulder pain)**										
1	RCS[85]	very serious[a,b,c]	NA	serious[a,c]	serious[b]	37	51	Both groups significantly did not cause shoulder pain	⊕◯◯◯-[a,b,c] Very low	Critical

Certainty assessment						No of patients		Impact	Certainty	Importance
№ of studies	Study design	Risk of bias	Inconsistency	Indirectness	Imprecision	Intervention	Comparison			
**E1.9. SND vs. others (SND with SND with sacrificed cervical plexus vs. SND with preserved cervical plexus) (Follow-up: range 2-6 weeks); (Assessed with: VAS- Shoulder pain)**										
1	PCS[49]	very serious[a,b,c]	NA	serious[a,c]	serious[b]	17	17	Both groups reported mild to moderate shoulder pain at two and six weeks after surgery.	⊕◯◯◯-[a,b,c] Very low	Critical
**E1.10. SND vs. others (SND with SND with EC vs. SND with HS) (Follow-up: range 1 day – 6 months post-surgery); (Assessed with: VAS- Shoulder pain)**										
1	RCT [93]	very serious[a,b,c]	NA	serious[a,c]	serious[b]	20	20	SND with EC statistically significant had caused shoulder pain after the surgery	⊕◯◯◯-[a,b] Very low	Critical
**E1.11. SND vs. others (SND vs. FND) (Follow-up: range 6 months to 8 months) (Assessed with: VAS - Shoulder pain)**										
1	PCS[37]	very serious[a, c]	NA	serious[c]	serious[c]	50	55	Shoulder pain was noted in both groups, no significant difference was found between the groups	⊕◯◯◯-[a,c] Very low	Critical
**E1.12. SND vs. others (SOND alone) (Follow-up: more than 1 year); (Assessed with: VAS - Shoulder pain)**										
1	RCS[79]	Extremely serious[a,b, c,e]	NA	very serious[c]	serious[b]	50	-	SOND can cause shoulder pain after the surgery	⊕◯◯◯-[a,b,c] Very low	Critical
**E1.13. Mixed ND (SND and MRND - preserved SAN alone) (Follow-up: range 10 days to 6 months) ;(Assessed with: VAS - Shoulder pain)**										
1	PCS[28]	very serious[a,c,e]	NA	serious[c]	serious[c]	45	-	SND and MRND with preserved SAN can cause shoulder pain	⊕◯◯◯-[a,c] Very low	Critical
**NECK PAIN**										
**E1.14. RND (Sacrificed SAN) vs. MRND (Preserved SAN) (Follow-up: not reported) (Assessed with: HRQOL-Neck pain)**										
1	RCS[81]	very serious[a,c,d]	NA	serious[c]	serious[c]	49	129	Neck pain was present in both groups after surgery. No significant difference in neck pain between groups was observed	⊕◯◯◯-[a,c,d] Very low	Critical
**E1.15. MRND vs. SND (Follow-up: after the surgery); (Assessed with: VAS - Neck pain)**										
1	PCS[53]	Very serious[a,b,c]	NA	serious[b]	serious[b]	16	55	Neck pain was present in both groups. No significant difference in neck pain between groups was observed.	⊕◯◯◯ [a,b,c] Very low	Critical
**E1.16. MRND vs. SND (Follow-up: range 6 months to more than 1 year); (Assessed with: VAS - Neck pain)**										
2	CS[60] PCS[53]	serious[a,b]	serious[a,b]	serious[b]	serious[b]	34	112	MRND can significantly cause long-term neck pain and was significantly different from SND	⊕◯◯◯-[a,b] Very low	Critical
**E1.17. SND vs. others (SND with radiotherapy vs. SND without radiotherapy) (Follow-up: range 6 months to 9 years); (assessed with: VAS - Neck pain)**										

Certainty assessment						No of patients		Impact	Certainty	Importance
№ of studies	Study design	Risk of bias	Inconsistency	Indirectness	Imprecision	Intervention	Comparison			
1	RCS [85]	very serious[a,b,c]	NA	serious[c]	serious[c]	37	51	Patients from both groups had no persistent neck pain at range of 6 months to 9 years after surgery	⊕◯◯◯-[a,b,c] Very low	Critical
**E1.18. SND vs. others (SND vs. non-surgical group) (Follow-up: after the surgery); (Assessed with: VAS - Neck pain)**										
1	PCS[53]	very serious[a,b,c]	NA	serious[c]	serious[c]	55	unclear	Neck pain was significantly higher after the SND surgery when compared with the non-surgical group	⊕◯◯◯-[a,b,c] Very low	Critical
**E1.19. SND vs. others (SND vs. non-surgical group) (Follow-up: more than 6 months post-surgery); (Assessed with: VAS - Neck pain)**										
1	PCS[53]	very serious[a,b,c]	NA	serious[c]	serious[c]	55	unclear	No neck pain during the long-term follow-up in the SND group.	⊕◯◯◯-[a,b,c] Very low	Critical
**E1.20. Mixed ND: SND and MRND (sacrificed cervical branches) vs. SND and MRND (preserved cervical branches) (Follow-up: more than 1-year post-surgery) ;(Assessed with: VAS - Neck pain)**										
1	RCS [84]	very serious[a,b,c]	NA	serious[c]	serious[c]	29	24	Neck pain was observed in both groups, but the severity of neck pain was significantly higher in the sacrificed group	⊕◯◯◯-[a,b,c] Very low	Critical

RCS: Retrospective cohort study; **PCS:** Prospective cohort study; **CS**: Cross-sectional study; **RCT**: Randomized controlled trial.

a. Serious or Critical risk of bias due to confounding, co-intervention, selections of participants, or measurement of outcomes; b. Different types of studies, comparison groups, type of HNC, surgery location and follow-up duration; c. Single study; d. Some studies did not mention the duration of follow-up, e. There is no comparison group.

Table S2: The overall quality of the evidence with the GRADE approach for a **range of motion** outcomes.

Certainty assessment						No of patients		Impact	Certainty	Importance
№ of studies	Study design	Risk of bias	Inconsistency	Indirectness	Imprecision	Intervention	Comparison			
**SHOULDER ROM**										
**E2.1. RND vs. others (RND vs. MRND) (Follow-up: 16 weeks post-surgery); (Assessed with: Goniometer - Shoulder abduction and flexion)**										
1	PCS [32]	very serious[a,b,c]	NA	serious[b]	serious[b]	11	21	RND and MRND significantly reduced shoulder abduction and flexion at 16 weeks post-surgery	⊕◯◯◯-[a,b,c] Very low	Critical
**E2.2. RND vs. others (RND vs. MRND) (Follow-up: range 6 months to 23 years post-surgery); (Assessed with: Goniometer - Shoulder abduction and flexion and AAT)**										
5	CS [64], [67], [69], [72] PCS[41]	very serious[a,b]	very serious[a,b,c]	serious[b]	serious[b]	102	215	RND significantly reduced shoulder abduction and flexion in the long term (i.e., **6 months to 23 years post-surgery)**	⊕◯◯◯-[a,b] Very low	Critical
**E2.3. RND vs. others (RND vs. SOND) (Follow-up: 16 weeks post-surgery); (Assessed with: Goniometer - Shoulder abduction and flexion)**										
1	PCS [32]	very serious[a,b,c]	NA	serious[b]	serious[b]	11	6	RND significantly reduced shoulder abduction and flexion in the short term when compared to SOND	⊕◯◯◯-[a,b,c] Very low	Critical
**E2.4. RND vs. others (RND vs. SND) (Follow-up: range 6 months to 12 months post-surgery); (Assessed with: Goniometer - Shoulder abduction and flexion)**										
3	CS[69], [72] PCS [41]	very serious[a,b]	very serious[a,b]	serious[b]	serious[b]	80	64	RND significantly reduced shoulder abduction and flexion in the long term when compared to SND	⊕◯◯◯-[a,b] Very low	Critical
**E2.5. MRND vs. others (MRND vs. non-surgical side) (Follow-up: range 6 months to 12 months post-surgery) ;(Assessed with: Goniometer - Shoulder abduction and flexion)**										
1	CS[59]	very serious[a,b,c]	NA	serious[b]	serious[b]	60	60	MRND significantly reduced shoulder abduction and flexion when compared to the non-surgical side	⊕◯◯◯-[a,b,c] Very low	Critical
**E2.6. MRND vs. others (MRND alone) (Follow-up: range 1 month and 6 months post-surgery) ;(Assessed with: Goniometer - Shoulder abduction and flexion)**										
1	PCS[30]	very serious[a,b,c]	NA	serious[b]	serious[b]	42	-	MRND can significantly reduce shoulder abduction and flexion	⊕◯◯◯-[a,b,c] Very low	Critical
**E2.7. MRND vs. others (MRND vs. SND) (Follow-up: range 1 week and 1-month post-surgery); (Assessed with: Goniometer - Shoulder abduction)**										
1	PCS[26]	very serious[a,c]	NA	serious[c]	serious[c]	33	32	MRND significantly reduced shoulder abduction when compared to SND in 1 week, 1 month after surgery.	⊕◯◯◯-[a,c] Very low	Critical
**E2.8. MRND vs. others (MRND vs. SND) (Follow-up: range 6 months post-surgery); (Assessed with: Goniometer - Shoulder abduction)**										

Certainty assessment						No of patients		Impact	Certainty	Importance
№ of studies	Study design	Risk of bias	Inconsistency	Indirectness	Imprecision	Intervention	Comparison			
2	PCS [26], [29]	very serious[a,b]	NA	serious[a,b]	serious[a,b]	62	110	MRND group showed lower shoulder abduction when compared to the SND. Mixed results when compiling information from both studies regarding statistical significance	⊕◯◯◯-[a,b] Very low	Critical
**E2.9. MRND vs. others (MRND with PMMC vs. MRND without PMMC) (Follow-up: range 3 months and 6 months post-surgery); (Assessed with: Goniometer - Shoulder Abduction and Flexion)**										
1	PCS[34]	very serious[a,c]	NA	serious[c]	serious[c]	20	20	Both groups significantly reduced shoulder flexion and abduction in 3 and 6 months. No significant difference between groups.	⊕◯◯◯-[a,c] Very low	Critical
**E2.10. SND vs others (SND vs. Non-surgical side) (Follow-up: range 2 months post-surgery); (Assessed with AAT and Goniometer: Shoulder Abduction and Flexion)**										
1	CS[75]	very serious[a,b,c]	NA	serious[b,c]	serious[b,c]	18	18	SND significantly reduced shoulder abduction and flexion after the surgery	⊕◯◯◯-[a,b,c] Very low	Critical
**E2.11. SND vs. others (SND vs. Non-surgical side) (follow-up: range 6 months to 12 years; assessed with AAT and Goniometer: Shoulder Abduction/Flexion)**										
2	RCS [85] CS [71]	very serious[a,b]	very serious[a,b,e]	serious[b,e]	serious[b,e]	114	100	A study[71]reported that SND can significantly reduce shoulder abduction and flexion; however, one study [85]did not find any significant differences between the groups.	⊕◯◯◯-[a,b,e] Very low	Critical
**E2.12. SND vs. others (SND alone) (follow-up: range 6 months to 12 years; assessed with AAT and Goniometer: Shoulder Abduction)**										
1	CS [56]	very serious[a,b,c]	NA	serious[b,c]	serious[b,c]	128	-	SND can cause a decrease in shoulder abduction even after more than 6 months of post-surgery when compared with the unaffected side.	⊕◯◯◯-[a,b,c] Very low	Critical
**E2.13. SNDIIb vs. others (SND IIb vs. SND IIa) (Follow-up: range 6 weeks post-surgery); (Assessed with: Goniometer - Shoulder abduction)**										
1	RCT [92]	very serious[a,b,c]	NA	serious[b,c]	serious[b,c]	18	32	Both groups did not have a negative impact on shoulder abduction at 6 weeks post-surgery when compared with the unaffected side	⊕◯◯◯-[a,b,c] Very low	Critical
**E2.14. SND vs. others (SND IIb vs SND IIa) (Follow-up: range 4 months to 6 months post-surgery); (Assessed with: Goniometer - Shoulder abduction)**										
2	RCT [91], [92]	very serious[a,b]	very serious[a,b,e]	serious[b,e]	serious[b,e]	33	47	One study [91] reported that SND IIb significantly reduces shoulder abduction. However, another study[92]did not find any differences	⊕◯◯◯-[a,b,e] Very low	Critical
**E2.15. SND vs. others (SND IIb spared bilaterally vs. SND IIb spared unilaterally) (Follow-up: range 21 days post-surgery); (Assessed with: Goniometer - Shoulder abduction)**										
1	PCS[33]	very serious[a,b,c]	NA	serious[b,c]	very serious[b,c]	25	16	Both groups had similar shoulder ROM when compared with baseline ROM	⊕◯◯◯-[a,b,c] Very low	Critical

Certainty assessment						No of patients		Impact	Certainty	Importance
№ of studies	Study design	Risk of bias	Inconsistency	Indirectness	Imprecision	Intervention	Comparison			
**E2.16. SND vs. others (SND IIb spared bilaterally vs. SND IIb spared unilaterally) (Follow-up: range 6 months post-surgery); (Assessed with: Goniometer - Shoulder abduction)**										
1	PCS[33]	very serious[a,b,c]	NA	serious[b,c]	serious[b,c]	25	16	Both groups had similar shoulder ROM when compared with baseline ROM	⊕◯◯◯-[a,b,c] Very low	Critical
**E2.17. SND vs. others (SND -level IIb-V vs. SND -level IIb preserved) (Follow-up: range 6 months to 0); (Assessed with: Goniometer - Shoulder abduction)**										
1	PCS[27]	very serious[a,b ,c]	NA	serious[b,c]	very serious[b,c]	16	9	No significant differences were found between the groups. However, the SND (level IIb-IV) dissected showed more reduction in shoulder abduction than SND -level IIb preserved	⊕◯◯◯-[a,b,c] Very low	Critical
**E2.18. SND vs. others (SND -level V vs. SND -level IIb-IV) (Follow-up: not reported); (Assessed with: Goniometer - Shoulder abduction)**										
1	RCS [82]	very serious[a,b,c,d]	NA	serious[b,d]	very serious[b,d]	20	20	No significant differences were found between the groups. However, SND (V) dissected showed more reduction in shoulder abduction than SND -level IIb-IV.	⊕◯◯◯-[a,b,c,d] Very low	Critical
**E2.19. SND vs. others (SND vs. FND) (Follow-up: 6 months post-surgery); (Assessed with: Goniometer - Shoulder abduction and flexion)**										
1	PCS [48]	very serious[a,b,c]	NA [c]	serious[b,c]	very serious[c]	14	12	FND significantly reduced shoulder abduction and flexion when compared with SND.	⊕◯◯◯-[a,b,c] Very low	Critical
**E2.20. Mixed ND (MRND and SND -removed cervical root branches vs. preserved cervical root branches) (Follow-up: 6 months to more than 12 months post-surgery); (Assessed with: Goniometer - Shoulder abduction)**										
2	PCS [42]<br>RCS [84]	very serious[a,b]	very serious[a,b]	serious[b,d]	serious[a,b]	60	47	One study [84]reported that both groups significantly reduced shoulder abduction with no significant difference between groups. Another study [42]reported that MRND with removed cervical root branches had worse shoulder abduction than the preserved group.	⊕◯◯◯-[a,b] Very low	Critical
**E2.21. Mixed ND (MRND and SND -preserved SAN alone) (Follow-up: Day 10th post-surgery); (Assessed with: Goniometer and Arm abduction test - Shoulder abduction)**										
1	PCS[28]	very serious[a,c,f]	NA	serious[b,c,f]	serious[a,c,f]	45	-	Mixed ND with preserved SAN can cause limited shoulder abduction	⊕◯◯◯- [a,b,c,f] Very low	Critical
**E2.22. Mixed ND (MRND and SND -preserved SAN alone) (Follow-up: 6 months post-surgery); (Assessed with: Goniometer and Arm abduction test - Shoulder abduction)**										
1	PCS[28]	very serious[a,c,f]	NA	serious[b,c,f]	serious[a,c,f]	45	-	Mixed ND with preserved SAN can cause limited shoulder abduction The study showed a statistically significant difference between time points	⊕◯◯◯- [a,b,c,f] Very low	Critical

Certainty assessment						No of patients		Impact	Certainty	Importance
№ of studies	Study design	Risk of bias	Inconsistency	Indirectness	Imprecision	Intervention	Comparison			
**E2.23. Mixed ND (MRND and SND alone) (Follow-up: range 1 month to 6months post-surgery); (Assessed with AAT and Goniometer: Shoulder Abduction and/or Flexion)**										
1	PS[35]	very serious[a,c,f]	NA	serious[b,c,f]	serious[a,c,f]	66	-	Mixed ND (SND and MRND can significantly reduce shoulder abduction at 1 month and 6 months follow-up when compared to pre-operative measurement	⊕◯◯◯- [a,b,c,f] Very low	Critical
**CERVICAL ROM**										
**E2.24. MRND vs. others (MRND vs. SND) (Follow-up: range 6 months to 5 years) ;(Assessed with: Cervical ROM (flexion and extension) - Inclinometer)**										
1	CS[76]	very serious[a,b,c]	NA	serious[b,c]	serious[a,b,c]	31	74	Both groups (**MRND vs. SND**) showed a reduction in cervical flexion and extension.	⊕◯◯◯- [a,b,c] Very low	Critical
**E2.25. MRND vs. others (MRND vs. SND) (Follow-up: range 6 months to 1 year); (Assessed with: Cervical ROM (lateral flexion) - Inclinometer)**										
1	PCS[53]	very serious[a,b,c]	NA	serious[b,c]	serious[a,b,c]	16	55	MRND showed a significant reduction in cervical lateral flexion compared to SND	⊕◯◯◯- [a,b,c] Very low	Critical
**E2.26. MRND vs. others (MRND vs. SOND) (Follow-up: range 2 months post-surgery) ;(Assessed with: Cervical ROM (all cervical ROM)- Inclinometer)**										
1	PCS[52]	very serious[a,b,c]	NA	serious[a,b,c]	serious[a,b,c]	83	25	MRND showed a significant reduction in cervical ROM when compared to SOND	⊕◯◯◯-[a,b,c] Very low	Critical
**E2.27. MRND vs. others (MRND vs. SOND) (Follow-up: 12 months post-surgery) ;(Assessed with: Cervical ROM (all cervical ROM)- Inclinometer)**										
1	PCS[52]	very serious[a,b,c]	NA	serious[a,b,c]	serious[a,b,c]	83	25	All movements in both groups improved after 12 months post-surgery, except cervical rotation for the MRND group.	⊕◯◯◯-[a,b,c] Very low	Critical
**E2.28. SND vs. others (SND vs. non-surgical) (Follow-up: range 6 months to 12 years post-surgery); (Assessed with: Cervical ROM (all cervical ROM) - Goniometer and Tape measurement)**										
2	RCS [85] CS[71]	very serious[a,b]	serious[a,b]	serious[a,b]	serious[a,b]	114	100	SND did not have significant differences in all cervical ROM when compared with the non-surgical side.	⊕◯◯◯-[a,b] Very low	Critical
**E2.29. SND vs. others (SND level 2b spared bilaterally vs. spared unilaterally) (Follow-up: 21 days post-surgery); (Assessed with: Cervical ROM (flexion/extension and rotation)- Goniometer)**										
1	PCS[33]	very serious[a,c]	NA	serious[a,c]	serious[a,c]	25	16	Both groups showed a reduction of cervical ROMs (flexion/extension and rotation) after 21 days post-surgery. No significant differences between groups.	⊕◯◯◯-[a,c] Very low	Critical
**E2.30. SND vs. others (SND level 2b spared bilaterally vs. spared unilaterally) (Follow-up: 6 months post-surgery); (Assessed with: Cervical ROM (flexion/extension and rotation) - Goniometer)**										
1	PCS[33]	very serious[a,c]	NA	serious[a,c]	serious[a,c]	25	18	Both groups showed improvement of cervical ROM at long-term follow-up (6 months). No significant differences between groups.	⊕◯◯◯-[a,c] Very low	Critical
**E2.31. SND vs. others (SOND vs. SNB) (Follow-up: not reported) ;(Assessed with: Cervical ROM (all cervical ROM) - Inclinometer)**										

Certainty assessment						No of patients		Impact	Certainty	Importance
№ of studies	Study design	Risk of bias	Inconsistency	Indirectness	Imprecision	Intervention	Comparison			
1	CS[62]	very serious[a,c]	NA	serious[a,c]	serious[a,c]	25	24	Both groups have similar cervical ROM after the surgery. Neither group experienced a reduction in neck range of motion.	⊕◯◯◯-[a,c] Very low	Critical
**E2.32. MRND and SND with removed cervical root branches vs. preserved cervical root branches (Follow-up: more than 12 months post-surgery); (Assessed with: Cervical ROM (all cervical ROM)- Inclinometer)**										
1	RCS [84]	serious[b,c,g]	NA	serious[b,c]	serious[b,c]	29	24	Neck side flexion was significantly reduced in the MRND and SND with nerves removed when compared with those individuals who had preserved cervical root branches, even after more than 12 months of surgery.	⊕◯◯◯-[a,b,c,g] Very low	Critical
**JAW ROM**										
**E2.33. MRND with EBRT vs. SND with EBRT (Follow-up: 2 months post-surgery) ; (Assessed with: Mouth opening- Goniometer)**										
1	PCS[52]	very serious[a,b,c]	NA	serious[a,b,c]	serious[a,b,c]	83	25	Limited mouth opening was found in MRND with EBRT, two months after surgery when compared to SND with EBRT.	⊕◯◯◯-[a,b,c,] Very low	Critical

RCS: Retrospective cohort study; **PCS**: Prospective cohort study; **CS:** Cross-sectional study; **RCT:** Randomized controlled trial.

a. Serious or Critical risk of bias due to confounding, co-intervention, selections of participants, or measurement of outcomes; b. Different types of studies, comparison groups, type of HNC, surgery location, and follow-up duration; c. single study; d. Some studies did not mention the duration of follow-up; e. one study did not support the hypothesis. ;f.No comparison group; g. Moderate risk of bias.

Table S3: The overall quality of the evidence with the GRADE approach for **muscle strength** outcomes.

Certainty assessment						No of patients		Impact	Certainty	Importance
№ of studies	Study design	Risk of bias	Inconsistency	Indirectness	Imprecision	Intervention	Comparison			
**SHOULDER MUSCLE STRENGTH**										
**E3.1. RND vs. others (RND with sacrificed SAN vs. RND with preserved SAN) (Follow-up: range 2 to 7 years post-surgery); (Assessed with: Shoulder abductor -MMT)**										
1	CS[67]	very serious[a,c]	NA	serious[a,c]	serious[a,c]	18	35	RND with sacrificed SAN had worst shoulder abduction strength compared to the preserved SAN group.	⊕◯◯◯-[a,c] Very low	Critical
**E3.2. RND vs. others (RND vs. MRND) (Follow-up: 16 weeks post-surgery); (Assessed with: Shoulder abductor/flexor/elevator - MMT)**										
1	PCS[32]	very serious[a,b,c]	NA	serious[a,b,c]	serious[a,b,c]	11	14	Both interventions decreased shoulder abductors and flexors strength at 16-week periods. No differences between groups were found.	⊕◯◯◯-[a,b,c] Very low	Critical
**E3.3. RND vs. others (RND vs MRND/SND) (Follow-up: 6 months post-surgery); (Assessed with: Shoulder abductor/flexor/elevator - MMT)**										
1	PCS[41]	very serious[a,b,c]	NA	serious[a,b]	serious[a,b,c]	23	35	At 6 months of follow-up, both groups had reduced shoulder muscle strength (elevator, flexors, and abductor), but the RND group significantly had more weakness in shoulder abductors and elevator muscles.	⊕◯◯◯-[a,b,c] Very low	Critical
**E3.4. RND vs. Others (RND vs. SND) (Follow-up: range 16 weeks post-surgery); (assessed with: Shoulder abductor/flexor/ elevator - MMT)**										
1	PCS[32]	very serious[a,b,c]	NA	serious[a,b,c]	serious[a,b,c]	11	13	RND has significantly weaker muscles (abductors and flexors) at 16 weeks of post-surgery when compared with SND	⊕◯◯◯-[a,b,c] Very low	Critical
**E3.5. RND vs. Others (RND vs. SND) (Follow-up: range 6 months post-surgery); (assessed with: Shoulder abductor/flexor/ elevator - MMT)**										
1	PCS[41]	very serious[a,b,c]	NA	serious[a,b,c]	serious[a,b,c]	23	34	RND has significantly weaker muscles (abductors, flexors, and elevators) at 6 months of post-surgery when compared with SND	⊕◯◯◯-[a,b,c] Very low	Critical
**E3.6. MRND with PMMF vs. MRND without PMMF (Follow-up: range 3 months post-surgery); (Assessed with: Shoulder Flexor/extensor/abductor/adductor/int./ext. rotator -MMT)**										
1	PCS[34]	very serious[a,c]	NA	serious[a,b,c]	serious[a,b,c]	20	20	Both groups showed a reduction in all shoulder muscle strength after 3 months of post-surgery. No significant difference between the groups was observed.	⊕◯◯◯-[a,c] Very low	Critical
**E3.7. SND vs. Others (SND 2b dissected vs. SND 2b preserved) (Follow-up: less than 3 months post-surgery); (Assessed with: Shoulder flexor/abductor/elevator -Dynamometer)**										

Certainty assessment						No of patients		Impact	Certainty	Importance
№ of studies	Study design	Risk of bias	Inconsistency	Indirectness	Imprecision	Intervention	Comparison			
1	PCS[27]	very serious[a,b,c]	NA	serious[a,b,c]	very serious[a,b,c]	16	9	Both groups (**SND 2b dissected vs. SND 2b preserved)** significantly decreased shoulder flexors, abductors, and elevators muscle strength. No significant difference between groups was observed.	⊕◯◯◯-[a,b,c] Very low	Critical
**E3.8. SND vs. Others (SND level 2b spared bilaterally vs. SND level 2b spared unilaterally) (Follow-up: range 21 days post-surgery); (Assessed with: Scapular elevators, adduction depressors, and adductors - MMT)**										
1	PCS[33]	very serious[a,b,c]	NA	serious[a,b,c]	serious[a,b,c]	25	16	Both groups did not change any scapular muscle strength after both surgeries (**SND level 2b spared bilaterally vs. SND level 2b spared unilaterally)**	⊕◯◯◯-[a,b,c] Very low	Critical
**E3.9. SND vs. Others (SND level 2b spared bilaterally vs. SND level 2b spared unilaterally) (Follow-up: range 6 months post-surgery); (Assessed with: Scapular elevators, adduction depressors, and adductors - MMT)**										
1	PCS[33]	very serious[a,b,c]	NA	serious[a,b,c]	serious[a,b,c]	25	16	Both groups did not change any scapular muscle strength after both surgeries (**SND level 2b spared bilaterally vs. SND level 2b spared unilaterally)**	⊕◯◯◯-[a,b,c] Very low	Critical
**E3.10. SND vs. Others (SND-level II-V vs. SND -level II-IV) (Follow-up: range more than 1-year post-surgery); (Assessed with: Shoulder flexor/abductor-MMT)**										
1	RCS[82]	very serious[a,b,c]	NA	very serious[a,b,c]	serious[a,b,c]	20	20	SND with level II – V had weaker muscle strength when compared to SND with level II-IV. However, significant differences between the groups were not reported in the study.	⊕◯◯◯-[a,b,c] Very low	Critical
**E3.11. Mixed ND (with preserved SAN) alone (Follow-up: range 3 months post-surgery); (Assessed with: Trapezius, serratus anterior, infraspinatus/teres minor and supraspinatus - Dynamometer)**										
1	CS [55]	very serious[a,b,c,d]	NA	serious[a,b,c,f]	serious[a,b,c,d]	14	-	Mixed ND with preserved SAN can significantly decrease the strength of trapezius, serratus anterior, infraspinatus, supraspinatus muscles after surgery	⊕◯◯◯-[a,b,c,d] Very low	Critical
**NECK MUSCLE STRENGTH**										
**E3.12. SND vs. Others (SND-level 2b spared bilaterally vs. SND-level 2b spared unilaterally) (Follow-up: range 21 days post-surgery); (Assessed with: Cervical flexor/extensor - MMT)**										
1	PCS[33]	very serious[a,b,c]	NA	serious[a,b,c]	serious[a,b,c]	25	16	Cervical flexors and extensors strength was significantly reduced for both groups, with no differences between them.	⊕◯◯◯-[a,b,c] Very low	Critical
**E3.13. SND vs. Others (SND-level 2b preserved unilaterally vs. SND-level 2b preserved bilaterally) (Follow-up: range 6 months post-surgery); (Assessed with: Cervical flexor/extensor - MMT)**										

Certainty assessment						No of patients		Impact	Certainty	Importance
№ of studies	Study design	Risk of bias	Inconsistency	Indirectness	Imprecision	Intervention	Comparison			
1	PCS[33]	very serious[a,b,c]	NA	serious[a,b,c]	serious[a,b,c]	25	16	Cervical flexors and extensors strength was significantly reduced for both groups, with no differences between them	⊕◯◯◯-[a,b,c] Very low	Critical
**E3.14. SND vs. others (SND-preserved SAN vs. MRND-preserved SAN) (Follow-up: range 6 months post-surgery);(Assessed with: Cervical flexor/extensor - Dynamometer)**										
1	CS [76]	serious[b,c,e]	NA	serious[b,c]	serious[b,c]	37	20	Both groups decreased cervical and extensor muscle strength at 6-month follow-up	⊕◯◯◯-[b,c,e] Very low	Critical
**RESPIRATORY MUSCLE STRENGTH**										
**E3.15. Mixed ND -level I-IV dissection alone (Follow-up: range 48 hours to 1 month); (Assessed with: Respiratory Muscle strength - MIP)**										
1	PCS[44]	very serious[a,b,c,d]	NA	serious[a,b,c]	serious[a,b,c]	43	0	MIP and SNIP Maximum inspiratory pressure (MIP) and nasal inspiratory pressure (SNIP) decreased at 48 and 72 hours after the surgery. At 1 month, the respiratory strength returned to the pre-surgery level	⊕◯◯◯-[a,b,c,d] Very low	Critical

RS: Retrospective cohort study; **PS:** Prospective cohort study; **CS:** Cross-sectional study; **RCT**: Randomized controlled trial.

a. Serious or Critical risk of bias due to confounding, co-intervention, selections of participants, or measurement of outcomes; b. Different types of studies, comparison groups, type of HNC, surgery location, and follow-up duration; c. Single study; d.No comparison group; e. Moderate risk of bias

Table S4: The overall quality of the evidence with the grade approach for **disability questionnaire** outcomes.

Certainty assessment						No of patients		Impact	Certainty	Importance
№ of studies	Study design	Risk of bias	Inconsistency	Indirectness	Imprecision	Intervention	Comparison			
**SHOULDER DISABILITY**										
**E4.1. RND vs. others (RND vs. MRND) (Follow-up: range 1-day post-surgery); (Assessed with: Shoulder disability - SFPS)**										
1	PCS[45]	very serious[a,b,c]	NA	serious[a,b,c]	serious[a,b,c]	12	23	RND significantly have more shoulder disability than MRND after the surgery.	⊕◯◯◯-[a,b,c] Very low	Critical
**E4.2. RND vs. others (RND vs. MRND) (follow-up: range 6 months post-surgery); (Assessed with: Shoulder disability - CAS, SPADI)**										
2	RCS[87] , CS[65]	very serious[a,b,i]	very serious[a,e,i]	serious[a,b,e]	serious[a,b,e]	59	59	One study [87]reported that the RND group had the worst score in CAS. However, in another study [65], RND and MRND, both groups have worse scores in SPADI after more than 6 months post-surgery.	⊕◯◯◯-[a,b,e,i] Very low	Critical
**E4.3. RND vs. others (RND vs. MRND/SND) (Follow-up: range 6 months to 12 months); (Assessed with: Shoulder disability - SDQ)**										
2	CS [72] , RCS [88]	very serious[a,b,e]	very serious[a,b,e]	serious[a,b,e]	serious[a,b,e]	22	61	One study [88] reported that RND has a significantly lower SDQ score when compared to MRND/SND. Another study [72]did not find any significant differences between the ND groups, but RND had lower scores in SDQ when compared to MRND	⊕◯◯◯-[a,b,e] Very low	Critical
**E4.4. MRND vs. others (MRND (monitored vs. non-monitored) (Follow-up: range 6 months post-surgery); (Assessed with: Shoulder disability -CSM and SPADI)**										
1	PCS[54]	Very serious[b,c,h]	NA	serious[b,c]	serious[b,c]	20	20	Both groups have worse CSM and SPADI scores at 6-month follow-up. No differences between groups	⊕◯◯◯-[b,c,h] Very low	Critical
**E4.5. MRND vs. others (MRND vs. SND) (Follow-up: range 1 week to 3 months post-surgery); (Assessed with: Shoulder disability - SPADI)**										
1	PCS [26]	very serious[a,b,c,h]	NA	serious[a,b,c]	serious[a,b,c]	33	32	Both groups had significantly worse SPADI outcomes at 1 week, 1 month and 3 months post-surgery. MRND group had significantly worse shoulder disability than the SND	⊕◯◯◯-[a,b,c,h] Very low	Critical

Certainty assessment						No of patients		Impact	Certainty	Importance
№ of studies	Study design	Risk of bias	Inconsistency	Indirectness	Imprecision	Intervention	Comparison			
**SHOULDER DISABILITY**										
**E4.6. MRND vs. others (MRND vs. SND) (Follow-up: range more than 1-year post-surgery); (Assessed with: Shoulder disability - CMS and DASH)**										
3	CS [60], [66], [68]	very serious[a,b,h,i]	serious[a,b]	serious[a,b]	serious[a,b]	96	209	MRND significantly had a worse CMS score than SND. There were no significant differences in the DASH score. However, MRND showed more shoulder disability than SND	⨁◯◯◯-[a,b,h,i] Very low	Critical
**E4.7. MRND vs. others (MRND vs. SOND) (Follow-up: 1-day post-surgery); (Assessed with: Shoulder disability (UWQOL))**										
1	PCS[38]	very serious[a,b,c]	NA	serious[a,b,c]	serious[a,b,c]	160	162	MRND group had significantly worse shoulder disability than the SND.	⨁◯◯◯-[a,b,c] Very low	Critical
**E4.8. MRND vs. others (MRND vs. SOND) (Follow-up: More than 12 months post-surgery) ;(Assessed with: Shoulder disability (SDQ))**										
1	RCS [78]	very serious[a,b,c]	NA	serious[a,b,c]	serious[a,b,c]	51	65	MRND group had significantly worse shoulder disability than the SND	⨁◯◯◯-[a,b,c] Very low	Critical
**E4.9. MRND vs. others (MRND with PMMC vs. without PMMC) (Follow-up: range 3 months post-surgery) ; (Assessed with: Shoulder disability - SDQ)**										
1	PCS[34]	very serious[a,c]	NA	serious[a,c]	serious[a,c]	20	20	Both groups had lower scores in SDQ, indicating high shoulder dysfunctions. However, no differences between the groups were found.	⨁◯◯◯-[a,c] Very low	Critical
**E4.10. SND vs. others (SND alone) (Follow-up: range 1-month post-surgery) ;(Assessed with: Shoulder disability – SPADI, WORC, SDQ)**										
2	RCS[77] PCS[35]	very serious[a,b,f,i]	very serious[a,b,i]	serious[a,b]	serious[a,b]	144	-	SND had worse scores in SPADI, WORC, and SDQ	⨁◯◯◯-[a,b,f,i] Very low	Critical
**E4.11. SND vs others (SND alone) (Follow-up: range 6 months to 2 years post-surgery);(Assessed with: Shoulder disability – SPADI, UWQOL, WORC, SDQ, DASH)**										
4	RCS [77] PCS [31], [35], [43]	very serious[a,b,f,i]	serious[a,b,f,i]	serious[a,b]	serious[a,b]	229	-	SND had worse shoulder disability score after surgery (1 and 6 months)	⨁◯◯◯-[a,b,f,i] Very low	Critical

Certainty assessment						No of patients		Impact	Certainty	Importance
№ of studies	Study design	Risk of bias	Inconsistency	Indirectness	Imprecision	Intervention	Comparison			
**SHOULDER DISABILITY**										
**E4.12. SND vs. others (SND vs Non-surgery) (Follow-up: range 2 years post-surgery);(Assessed with: Shoulder disability – UWQOL)**										
1	RCS [89]	very serious[a,b,c]	NA	serious[a,b,c]	serious[a,b,c]	37	89	No significant differences were found between the groups; however, SND groups had worse HRQOL scores than the non-surgery group.	⨁◯◯◯-[a,b,c] Very low	Critical
**E4.13. SND vs. others (SND vs. FND) (Follow-up: more than 6 months post-surgery) ; (Assessed with: Shoulder disability - SPADI and GARS)**										
2	PCS [37], [48]	very serious[a,b]	very serious[a,b]	serious[a,b]	serious[a,b]	64	67	SND had significantly greater score in SPADI and GARS when compared to FND	⨁◯◯◯-[a,b] Very low	Critical
**E4.14. SND vs. others (SND vs. SND with radiotherapy/chemoradiation) (Follow-up: more than 6 months post-surgery) ; (Assessed with: Shoulder disability - CMS)**										
1	CS [63]	very serious[a,b,c]	NA	serious[a,b,c]	serious[a,b,c]	7	27	All groups had lower scores in the CMS	⨁◯◯◯-[a,b,c] Very low	Critical
**E4.15. SND vs. others (SND unilateral V vs. SND bilateral III-IV) (Follow-up: more than 6 months post-surgery); (Assessed with: Shoulder disability - UWQOL)**										
1	PCS [39]	very serious[a,c]	NA	serious[a,c]	serious[a,c]	181	39	Both groups had worse shoulder domain scores in UWQOL even after 6-month surgery. No differences observed between groups.	⨁◯◯◯-[a,c] Very low	Critical
**E4.16. SOND vs. others (SOND vs. SNB) (Follow-up: after surgery); (Assessed with: Shoulder disability - CMS)**										
1	CS [62]	very serious[a,b,c]	NA	serious[a,b,c]	serious[a,b,c]	25	24	SNB had a better CMS score compared to SOND after surgery.	⨁◯◯◯-[a,b,c] Very low	Critical
**E4.17. SOND vs. others (SOND vs. MISOND) (Follow-up: range 6 weeks post-surgery) ;(Assessed with: Shoulder disability - SPADI)**										
1	CS [61]	very serious[a,b,c]	NA	serious[a,b,c]	serious[a,b,c]	35	22	MISOND scored significantly better in the SPADI than the SOND group after 6 weeks of post-surgery.	⨁◯◯◯-[a,b,c] Very low	Critical
**E4.18. SOND vs. others (SOND vs. Extended SOND) (Follow-up: range more than 1-year post-surgery); (Assessed with: Shoulder disability - DASH)**										
1	RCS [80]	very serious[a,b,c]	NA	serious[a,b,c]	serious[a,b,c]	42	12	Both groups have minor shoulder disability evaluated with DASH after 1-year post-surgery.	⨁◯◯◯-[a,b,c] Very low	Critical

Certainty assessment						No of patients		Impact	Certainty	Importance
№ of studies	Study design	Risk of bias	Inconsistency	Indirectness	Imprecision	Intervention	Comparison			
**SHOULDER DISABILITY**										
**E4.19. SOND vs. others (SOND vs. Mixed ND) (Follow-up: range 1 month to 3 months post-surgery); (Assessed with: Shoulder disability - CMS and UWQOL)**										
1	RCT [90]	very serious[c,j]	NA	serious[c]	serious[c]	34	34	SOND had a better score in CMS and UWQOL - shoulder domain than the Mixed ND at 1- and 3-month post-surgery.	⊕◯◯◯-[c,j] Very low	Critical
**E4.20. Mixed ND vs. others (SND and MRND - preserved SAN alone) (Follow-up: range 3 months post-surgery); (Assessed with: Shoulder disability - CMS)**										
1	CS [55]	very serious[a,b,c,f]	NA	serious[a,b,c,f]	serious[a,b,c,f]	14	-	SND and MRND with preserved SAN had lower CMS scores after the surgery.	⊕◯◯◯-[a,b,c,f] Very low	Critical
**E4.21. Mixed ND vs. others (Mixed ND with PMMC vs Mixed ND without PMMC) (Follow-up: range 6 months post-surgery) ; (Assessed with: Shoulder disability - DASH and CMS)**										
2	PCS[36], [51]	very serious[a,b,h]	serious[a,b]	serious[b]	serious[b]	68	81	Both groups have higher DASH scores and low CMS scores, indicating high shoulder disability after surgery.	⊕◯◯◯-[a,b,h] Very low	Critical
**SHOULDER AND NECK DISABILITY**										
**E4.22. RND vs. FND (Follow-up: range 3 months to 9 months post-surgery); (Assessed with: Shoulder and neck disability -NDII)**										
1	PCS[47]	very serious[a,b,c]	NA	serious[a,b,c]	serious[a,b,c]	10	32	RND had a worse score in NDII when compared to FND post-surgery (3-9 months)	⊕◯◯◯-[a,b,c] Very low	Critical
**E4.23. MRND vs. SND (Follow-up: more than 1-year post-surgery) ; (Assessed with: Shoulder and neck disability - NDII)**										
2	CS [60], [66]	very serious[a,b,h]	serious[a,b]	serious[a,b]	serious[a,b]	77	195	MRND have lower scores in NDII when compared to SND after 1 year of surgery.	⊕◯◯◯-[a,b,h] Very low	Critical
**E4.24. SND (level 2a-4) vs. SND (level 2a-4 with 2b) (Follow-up: range 6 months post-surgery) ; (Assessed with: Shoulder and neck disability - NDII)**										
1	RCT [91]	very serious[b,c,j]	NA	serious[b,c,j]	serious[b,c,j]	15	15	Both groups have lower NDII scores. However, SND with level 2b scores significantly lower than SND without level 2b dissected.	⊕◯◯◯- [b,c,j] Very low	Critical

Certainty assessment						No of patients		Impact	Certainty	Importance
№ of studies	Study design	Risk of bias	Inconsistency	Indirectness	Imprecision	Intervention	Comparison			
**NECK DISABILITY**										
**E4.25. MRND vs. SND (Follow-up: more than 1-year post-surgery) ; (Assessed with: Neck disability - NDI)**										
1	CS [60]	very serious[b,c,h]	NA	serious[b,c]	serious[b,c]	21	16	There were no statistical differences between the groups. But MRND has slightly higher NDI (worse) compared to SND	⊕◯◯◯-[b,c,h] Very low	Critical
**E4.26. Mixed ND (spared CN XI) vs. without ND (Follow-up: range 1 year to 4 years post-surgery) ;(Assessed with: Neck disability – NPDS, NPNPQ)**										
1	PCS [50]	very serious[a,b,c]	NA	serious[a,b,c]	serious[a,b,c]	20	20	Mixed ND with spared CN XI had poorer scores in NPDS and NPNPQ than the non-neck dissection group.	⊕◯◯◯-[a,b,c] Very low	Critical

RCS: Retrospective cohort study; **PCS:** Prospective cohort study; **CS:** Cross-sectional study; **RCT:** Randomized controlled trial.

a. Serious or Critical risk of bias due to confounding, co-intervention, selections of participants, or measurement of outcomes; b. Different types of studies, comparison groups, type of HNC, surgery location, and follow-up duration; c. single study; d. Some studies did not mention the duration of follow-up, e. One study did not support the hypothesis; f.No comparison group; g. Evaluated different cervical ROM; h. Moderate risk of bias; i. Different outcome measures; j. some concern ROB
