# Supplementary material for: Musculoskeletal Impairments and Dysfunction in Individuals with Head and Neck Cancer Following Surgery with Neck Dissection—A Systematic Review
Source: Life (Basel). 2025 May 17;15(5):800. doi: 10.3390/life15050800 (PMC12112850; doi:10.3390/life15050800)
Supplement: Supplementary file 1 [file life-15-00800-s001.zip › Supplementary_Materials_F.pdf]

SUPPLEMENTARY MATERIALS F: Matrix table of the results.

Table S5: Matrix table of the results for the **pain** outcome.

**Groups**		**Study Design**	**Follow-up**	**Types of HNC**	**Outcomes**	**Summary**	**Quality of assessment (ROBINS [a] or ROB [b])**
**Intervention(s)**	**Comparison**	**PAIN**					
**RND**	**Others**	**SHOULDER PAIN**					
i. RND sacrificed SAN	MRND preserved SAN.	RCS[81]	Not reported	Mixed HNC	HRQOL shoulder domain	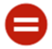	Critical [a]
		PCS[45]	6 weeks post-surgery	Mixed HNC	VAS	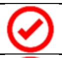	Critical [a]
ii. RND	SND	CS[69]	10 days of post-surgery	Mixed HNC	VAS	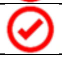	Critical [a]
**MRND**	**Others**						
i.MRND	SND	CS [69]	13.2 days of post-surgery	Mixed HNC	VAS	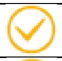	Critical [a] [69]
		CS [60]	More than 1 year of post-surgery			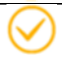	Moderate [a] [60]
ii. MRND	SOND	PCS[38]	More than 1 year of post-surgery	Oropharyngeal	UWQOL- Shoulder domain	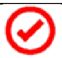	Serious [a]
**SND**	**Others**						
i.SND	Non-surgical side	CS[75]	After 2.6 months of post-surgery	Mixed HNC	VAS	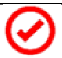	Moderate [a]
iia.SND with radiotherapy	SND without radiotherapy	RCS[85]	After more than 1 year of post-surgery (range, 0.5–9.1 years)	Mixed HNC	VAS	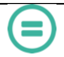	Critical [a]
iib.SND with sacrified cervical plexus	SND with preserved cervical plexus	PCS[49]	2 and 6 weeks of post-surgery	Mixed HNC	VAS		Critical [a]
iic.SND with EC	SND with HS	RCT [93]	Day 1 – 6 months of post-surgery	oropharyngeal [93]	VAS	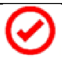	Some concern [b]
iii.SND	FND	PCS[37]	6 months of post-surgery (range 6-8 months)	Oral cavity	VAS	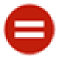	Critical [a]

iv. SOND	No comparison group	RCS[79]	more than 1 year (range 2.6 years) post-surgery	Mixed ND	VAS	!	Critical [a]
**Mixed ND: SND and MRND (preserved SAN)**	**No comparison group**	PCS[28]	6 months of post-surgery	Oropharyngeal	VAS	!	Critical [a]
**NECK PAIN**							
**RND (sacrificed SAN)**	**MRND (preserved SAN)**	RCS[81]	Not reported	Mixed HNC	HRQOL	⊜	Critical [a]
**MRND**	**SND**	PCS[53]	Shortly after the surgery	Mixed HNC	VAS	⊜	Moderate [a][60]<br>Critical [a] [53]
		CS[60]<br>PCS[53]	After 6 months [53] and more than 1.7 year of post-surgery[60]			✓	
**SND**	**Others**						
i. SND (with radiotherapy)	SND without radiotherapy)	RCS [85]	After more than 1 year of post-surgery(range: 0.5–9.1 years).	Mixed HNC	VAS	⊜	Critical [a]
ii. SND	Non-surgical group	PCS[53]	After surgery	Mixed HNC	VAS	✓	Critical [a]
			After 6 months of post-surgery			⊜	
**Mixed ND : SND and MRND (sacrificed cervical branches)**	**SND and MRND (preserved cervical branches)**	RCS [84]	After more than 1 year of post-surgery (range 1-2.8 years)	Mixed HNC	VAS	✓	Moderate [a]

Table S6: Matrix table of the results for the **range of motion** outcome.

**Groups**		**Study Design**	**Follow-up**	**Types of HNC**	**Outcomes**	**Summary**	**Quality of assessment (ROBINS [a] or ROB [b])**
**Intervention**	**Comparison**	**RANGE OF MOTION (ROM)**					
**RND**	**Others**	**SHOULDER ROM**					
i. RND	MRND	PCS [32]	16 weeks of post-surgery	Mixed HNC	Goniometer and Arm abduction test (ATT) score[70]	Shoulder abduction and flexion: 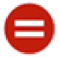	Critical [a][67], [69], [70], [72] Serious [a] [32], [41] , Moderate [a] [64]
		CS [64], [67], [69], [72] PCS[41]	6 to12 months [41], [69], [72] ; 2 to7 years of post-surgery[64], [67] and 1 to 23 years of post- surgery[70]	Oral carcinoma[67] , Mixed HNC [32], [41], [64], [69], [70], [72]		Shoulder abduction and flexion: 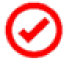	
ii. RND	SOND	PCS [32]	16 weeks	Mixed HNC	Goniometer	Shoulder abduction and flexion: 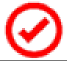	Serious [a] [32]
iii. RND	SND	CS[69], [72] PCS [41]	6 to 12 months of post-surgery	Mixed HNC	Goniometer	Shoulder abduction and flexion: 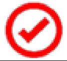	Critical [a] [69], [72] Serious [a][41]
**MRND**	**Others**						
i. MRND	Non-surgical side	CS[59]	6 to12 months of post-surgery	Mixed HNC	Goniometer	Shoulder abduction and flexion: 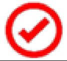	Serious [a]
ii. MRND	No comparison group	PCS[30]	1 and 6 months	Mixed HNC	Goniometer	Shoulder abduction and flexion: 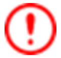	Serious [a]

iii. MRND	SND	PCS[26]	1 week and 1-month post-surgery	Mixed HNC	Goniometer	Shoulder abduction: ✔	Moderate [a][26]<br>Critical [a][29]
		PCS [26], [29]	6 months	Mixed HNC[26] and Oral [29]		Shoulder abduction: ✔ [26] ✓ [29]	
**MRND with PMMC**	**MRND without PMMC**	PCS[34]	3 and 6 months of post- surgery	Oral	Goniometer	Shoulder abduction and flexion: ⊜	Serious [a]
**SND**	**Others**						
i. SND	Non-surgical side	CS[75]	2.6 months of post-surgery	Mixed HNC	Goniometer and AAT score	Shoulder abduction and flexion: ✔	Moderate [a] [75]<br>Serious [a] [71]<br>Critical [a] [85]
		RCS [85]<br>CS [71]	6 months to 12 years of post-surgery	Mixed HNC [85]<br>Oral and Oropharynx carcinoma [71].		Shoulder abduction and flexion: ✔ [71] ⊜ [85]	
ii. SND	No comparison group	CS [56]	More than 6 months post-surgery	Oropharynx carcinoma	Goniometer and AAT score	Shoulder abduction: ❗	Critical
iiia. SND IIb	SND IIa	RCT [92]	6 weeks of post-surgery	Oral carcinoma [92].	Goniometer	Shoulder abduction: ⊜	Some concern [b] [91]<br>High risk [b] [92]
		RCT [91], [92]	4 to 6 months of post-surgery	Oral carcinoma [92].<br>Mixed HNC [91]		Shoulder abduction: [92]. ⊜<br>Shoulder abduction and external rotation: ✔ [91]	
iiib. SND IIb spared bilaterally	SND IIb spared unilaterally	PCS[33]	21 days of post-surgery	Mixed HNC	Goniometer	Shoulder abduction: ⊜	Serious [a]

			6 months of post-surgery			Shoulder abduction:	
iiic. SND IIb - level V dissection	SND IIb preserved	PCS[27]	3 months of post-surgery	Mixed HNC	Goniometer	Shoulder abduction:	Critical [a]
iv.SND (level V)	SND (level II - IV)	RCS [82]	Not reported	Mixed HNC	Goniometer	Shoulder abduction:	Critical [a]
v. SND	FND	PCS [48]	6 months of post-surgery	Laryngeal cancer	Goniometer	Shoulder abduction and flexion:	Serious [a]
**Mixed ND**	**Others**						
i. MRND and SND with cervical root branches removed	MRND and SND with preserved cervical root branches	PCS [42]<br>RCS [84]	6 to more than 12 months of post-surgery	Mixed HNC	Goniometer	Shoulder abduction:<br>[42] [84]	Critical [a] [42]<br>Moderate [a] [84]
ii. MRND and SND with preserved SAN	No comparison group	PCS[28]	Day 10 of post-surgery	Oropharyngeal cancer	Goniometer and AAT score	Shoulder abduction	Critical [a]
			6 months of post-surgery			Shoulder abduction	
iii. MRND and SND	No comparison group	PS[35]	1 month and 12 months post-surgery	Mixed HNC	Goniometer and AAT score	Shoulder abduction	Serious [a]
**CERVICAL ROM**							
**MRND**	**Others**						
i. MRND	SND	CS[76]	6 months to 5 years of post-surgery	Mixed HNC	Inclinometer	Flexion/Extension	Moderate [a]
		PCS[53]	6 months to 1 year of post-surgery			Lateral Flexion	Critical [a]

ii. MRND	SOND	PCS[52]	2 months of post-surgery	Mixed HNC	Inclinometer	All cervical ROM 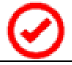	Critical[a]
			12 months of post-surgery			Rotation 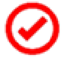	
**SND**	**Others**						
i. SND	Non-surgical side	RCS [85] CS[71]	6 months to 12 years of post-surgery	Oral and oropharyngeal cancer [71] and mixed HNC [85]	Goniometer[85] and tape measurement[71]	All cervical ROM: 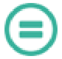	Critical[a] [85] Serious[a] [71]
ii. SND (level 2b spared bilaterally)	SND (level 2b spared unilaterally)	PCS[33]	Day 21 of post-surgery	Laryngeal carcinoma	Goniometer	Flexion, extension and rotation: 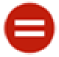	Serious[a]
			6 months of post-surgery			Flexion, extension and rotation: 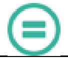	
iii. SOND	Sentinel node biopsy (SNB)	CS[62]	Not reported	Oral and oropharynx carcinoma	Inclinometer	All cervical ROM 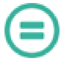	Critical[a]
**Mixed ND : MRND and SND with removed cervical root branches**	**MRND and SND preserved cervical root branches**	RCS [84]	More than 12 months of post-surgery	Mixed HNC	Inclinometer	Lateral neck flexion 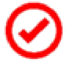	Moderate[a]
**JAW RANGE OF MOTION**							
**MRND with EBRT**	**SOND with EBRT**	PCS[52]	2 months	Mixed HNC	Ruler	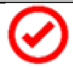	Critical[a]

Table S7: Matrix table of the results for the **muscle strength** outcome.

Groups		Study Design	Follow-up	Types of HNC	Outcome measure	Summary	Quality of assessment (ROBINS [a] or ROB [b])
**Intervention**	**Comparison**	**MUSCLES STRENGTH**					
**RND**	**Others**	**SHOULDER MUSCLES**					
i. RND with sacrificed SAN	RND with preserved SAN	CS[67]	2 to 7 years of post-surgery	Oral cancer	Manual Muscle Testing (MMT)	Shoulder Abductors: ✓	Critical [a]
ii. RND	MRND	PCS[32]	16 weeks of post-surgery	Mixed HNC	MMT	Shoulder abductors and flexors: =	Serious [a]
		PCS[41]	6 months of post-surgery			Shoulder abductors and elevators: ✓<br>Flexor: =	
iii. RND	SND	PCS[32]	16 weeks of post-surgery	Mixed HNC	MMT	Shoulder abductors and flexors: ✓	Serious [a]
		PCS[41]	6 months of post-surgery			Shoulder abductors, elevators and flexors: ✓	
**MRND with PMMF**	**MRND without PMMF**	PCS[34]	3 months post-surgery	Oral cancer	MMT	Shoulder flexors , abductors , extensors , internal and external rotators : =	Serious [a]

**SND**	**Others**						
i. SND (IIb dissected)	SND (IIb preserved)	PCS[27]	Less than 3 months of post-surgery	Mixed HNC	Dynamometer	Shoulder elevator, flexor and abductor :	Critical [a]
ii. SND (level 2b spared bilaterally)	SND (level 2b spared unilaterally)	PCS[33]	Day 21 post-surgery	Laryngeal	MMT	Scapular elevators, adduction depressors, and adductors	Serious [a]
			6 months post-surgery				
iii. SND (level II-V)	SND (level II-IV)	RCS[82]	More than 1-year post-surgery	Mixed HNC	MMT	Shoulder flexors and abductors :	Critical [a]
**Mixed ND (preserved SAN)**	**No comparison group**	CS [55]	3 months post-surgery	Mixed HNC	Dynamometer	Trapezius ,serratus anterior ,infraspinatus /teres minor and supraspinatus	Serious [a]
**NECK MUSCLE STRENGTH**							
**SND**	**Others**						
i. SND (level 2b spared bilaterally)	SND (level 2b spared uniaterally)	PCS[33]	21 days post-surgery	Mixed HNC	MMT	Neck flexors and extensors :	Serious [a]
			6 months post-surgery			Neck flexors and extensors :	
ii. SND (preserved SAN)	MRND (preserved SAN)	CS [76]	More than 6 months post-surgery	Mixed HNC	Dynamometer	Neck flexors and extensors :	Moderate [a]

RESPIRATORY MUSCLE STRENGTH							
**Mixed ND (RND , MRND , SND)**	**No comparison group**	PCS[44]	48 hours, 72 hours and 1-month post-surgery	Mixed HNC	Maximum inspiratory pressure (MIP) and nasal inspiratory pressure (SNIP)	48 and 72 hours : 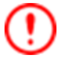 1 month : 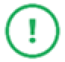	Critical [a]

Table S8: Matrix table of the results for the **disability questionnaire** outcome.

Groups		Study Design	Follow-up	Types of HNC	Outcome measure	Summary	Quality of assessment (ROBINS [a] or ROB [b])
**Intervention**	**Comparison**	**DISABILITY**					
**RND**	**Others**	**SHOULDER DISABILITY**					
i. RND	MRND	PCS[45]	After surgery [45]	Mixed HNC	Shoulder Function and Performance Score (SFPS)		Critical [a]
		RCS[87] , CS[65]	More than 6 months of surgery		Clinical Assessment Score (CAS) [87] , Shoulder Pain and Disability Index (SPADI) [65]	[87] [65]	Serious [a]
ii. RND	MRND/SND	CS [72] , RCS [88]	6 and 12 months of post-surgery	Mixed HNC	Shoulder Disability Questionnaire (SDQ)	[72] [88]	Critical [a]
**MRND**	**Others**						
i. MRND (monitored)	MRND (non-monitored)	PCS[54]	6 weeks and 6 months of post-surgery	Mixed HNC	Constant Murley Score (CSM) and SPADI		Moderate [a]
ii. MRND	SND	PCS [26]	1 week, 1 month and 3 months of post-surgery	Mixed HNC	SPADI		Moderate [a][26],[60] , Serious [a][66] and Critical [a][68]
		CS [60], [66], [68]	More than 12 months of post-surgery		CMS [66], [68] and DASH [60]	[66], [68] [60]	
iii. MRND	SOND	PCS[38]	After surgery	Oral cancer	University of Washington Quality-of-Life (UW-QOL)		Serious [a]
		RCS [78]	More than 1 year of post-surgery	Mixed HNC	SDQ		
iv. MRND with PMMC	MRND without PMMC	PCS[34]	3 months	Oral cancer	SDQ		Serious [a]

**SND**	**Others**						
i. SND	No comparison group	RCS[77]<br>PCS[35]	1 month	Oral cancer [77] and Mixed HNC[35]	SDQ[77], SPADI[77] and WORC[35]	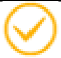	Serious [a] [35], [43], [77]<br>Critical [a] [31]
		RCS [77]<br>PCS [31], [35], [43]	6 months to 2 years post-surgery	Mixed HNC[35], nasopharyngeal carcinoma[31], tongue [43] and oral cancer[77].	SDQ[77]<br>SPADI[77]<br>UW-QOL [43],<br>Western Ontario Rotator Cuff (WORC) [35]<br>Disability of the arm, shoulder and hand (DASH) [31]	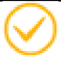<br>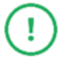 [43], (UWQOL)	
ii. SND	Non-surgery group	RCS [89]	2 years post-surgery	Mixed HNC	UW-QOL	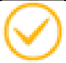	Serious [a]
ii. SND	FND	PCS [37], [48]	More than 6 months of post-surgery	Laryngeal [48] and tongue carcinoma [37]	SPADI[37], [48]<br>Groningen Activity Restriction Scale (GARS) [37]	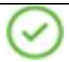	Serious [a] [48]<br>Critical [a] [37]
iii. SND	SND (radiotherapy and chemoradiation)	CS [63]	More than 6 months of post-surgery	Mixed HNC	CMS	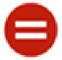	Critical [a]
iv. SND (unilateral level V	SND (bilateral level III-IV)	PCS [39]	More than 6 months of post-surgery	Oropharyngeal carcinoma	UWQOL	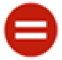	Critical [a]
**SOND**	**Others**						
i. SOND	SNB	CS [62]	After surgery	Mixed HNC	CMS	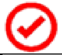	Serious [a]
ii. SOND	MISOND	CS [61]	6 weeks of post-surgery	Mixed HNC	SPADI	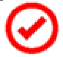	Serious [a]
iii. SOND	Extended SOND	RCS [80]	More than 1 year of post-surgery	Mixed HNC	DASH		Critical [a]
iv. SOND	Mixed ND	RCT [90]	1 and 3 months of post-surgery	Oral cancer	CMS and UWQOL	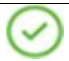	Some concern [b]

**Mixed ND**	**Others**						
i. SND and MRND (preserved SAN)	No comparison group	CS [55]	3 months of post-surgery	Mixed HNC	CMS		Critical [a]
ii. Mixed ND with PMMC	Mixed ND without PMMC	PCS[36], [51]	6 months of post-surgery	Oropharyngeal cancer [36] and mixed HNC[51]	DASH[36]<br>CMS [51]		Moderate [51]<br>Critical [36]
**SHOULDER AND NECK DISABILITY**							
**RND**	**FND**	PCS[47]	3 months and 9 months of post-surgery	Mixed HNC	Neck Dissection Impairment Index (NDII)		Serious [a]
**MRND**	**SND**	CS [60], [66]	More than 1 year of post-surgery	Mixed HNC	NDII		Serious [a][66]<br>Moderate [a] [60]
**SND (level 2a-4) with 2b**	**SND (level 2a-4)**	RCT [91]	6 months of post-surgery	Mixed HNC	NDII		Some concern [b]
**NECK DISABILITY**							
**MRND**	**SND**	CS [60]	More than 1-year post-surgery	Mixed HNC	Neck Disability Index (NDI)		Moderate [a]
**Mixed ND (spared CN XI)**	**Without ND**	PCS [50]	1-4 years of post-surgery	Laryngeal carcinoma	Neck Pain and Disability Scale (NPDS)<br>Northwick Park Neck Pain Questionnaire (NPNPQ)		Critical [a]

Abbreviation : Radical neck dissection (RND) , Modified radical neck dissection (MRND), Selective neck dissection (SND) , Functional neck dissection (FND), Neck dissection (ND), Pectoralis major myocutaneous (PMMC), Supraomohyoid Neck Dissection (SOND), Spinal accessory nerve (SAN), Cranial nerve (CN), external beam radiation therapy (EBRT) , Prospective cohort study(PCS), Retrospective cohort study (RCS) , Cross-sectional (CS), Randomized controlled trial (RCT), Minimally invasive supraomohyoid neck dissection (MISOND)

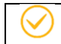	The intervention group experienced a negative outcome; however, no significant differences between the groups were reported, or group comparisons were not conducted.
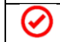	The intervention group experienced a negative outcome, with significant differences observed between the groups.
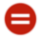	Both groups experienced negative outcomes. No significant differences between the groups were reported
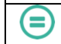	Neither group experienced a negative outcome.
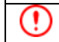	The intervention group experienced a negative outcome. No active comparison group existed in the study.
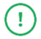	The intervention group did not experience negative outcomes. No active comparison group existed in the study.
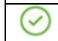	The intervention group achieved better outcomes than the comparison group, with significant differences between the groups.
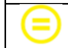	Both groups experienced minor/moderate disability, with no significant differences between them.
